# A Dipteran’s Novel Sucker Punch: Evolution of Arthropod Atypical Venom with a Neurotoxic Component in Robber Flies (Asilidae, Diptera)

**DOI:** 10.3390/toxins10010029

**Published:** 2018-01-05

**Authors:** Stephan Holger Drukewitz, Nico Fuhrmann, Eivind A. B. Undheim, Alexander Blanke, Julien Giribaldi, Rosanna Mary, Guillaume Laconde, Sébastien Dutertre, Björn Marcus von Reumont

**Affiliations:** 1 Institute for Biology, University of Leipzig, Talstr. 33, 04103 Leipzig, Germany; 2 Max Planck Institute for Evolutionary Biology, August-Thienemann-Str. 2, 24306 Plön, Germany; fuhrmann@evolbio.mpg.de; 3 Centre for Advanced Imaging, The University of Queensland, St. Lucia, Brisbane, QLD 4072, Australia; e.undheim@uq.edu.au; 4 Institute for Zoology, Biocenter, University of Cologne, Zuelpicher Str. 47b, 50674 Cologne, Germany; a.blanke@uni-koeln.de; 5 Medical and Biological Engineering Research Group, School of Engineering and Computer Science, University of Hull, Hull HU6 7RX, UK; 6 Institute for Biomolecules Max Mousseron, UMR 5247, University of Montpellier—CNRS, Place Eugène Bataillon, 34095 Montpellier CEDEX 5, France; julien.giribaldi@umontpellier.fr (J.G.); rosanna.mary@umontpellier.fr (R.M.); guillaume.laconde@umontpellier.fr (G.L.); sebastien.dutertre@umontpellier.fr (S.D.); 7 Department of Life Sciences, Natural History Museum, Cromwell Rd, London SW7 5BD, UK

**Keywords:** Asilidae, neurotoxins, cysteine inhibitor knot peptide, arthropod venom evolution, functional morphology, synchrotron micro computed tomography, Asilidin, This study provides the first comprehensive description of the venom system of two robber flies (Asilidae). We reveal a complex venom apparatus and an unusual, enzyme depleted venom with unique proteins, including also a new, neurotoxic ICK peptide.

## Abstract

Predatory robber flies (Diptera, Asilidae) have been suspected to be venomous due to their ability to overpower well-defended prey. However, details of their venom composition and toxin arsenal remained unknown. Here, we provide a detailed characterization of the venom system of robber flies through the application of comparative transcriptomics, proteomics and functional morphology. Our results reveal asilid venoms to be dominated by peptides and non-enzymatic proteins, and that the majority of components in the crude venom is represented by just ten toxin families, which we have named Asilidin1–10. Contrary to what might be expected for a liquid-feeding predator, the venoms of robber flies appear to be rich in novel peptides, rather than enzymes with a putative pre-digestive role. The novelty of these peptides suggests that the robber fly venom system evolved independently from hematophagous dipterans and other pancrustaceans. Indeed, six Asilidins match no other venom proteins, while three represent known examples of peptide scaffolds convergently recruited to a toxic function. Of these, members of Asilidin1 closely resemble cysteine inhibitor knot peptides (ICK), of which neurotoxic variants occur in cone snails, assassin bugs, scorpions and spiders. Synthesis of one of these putative ICKs, U-Asilidin_1_-Mar1a, followed by toxicity assays against an ecologically relevant prey model revealed that one of these likely plays a role as a neurotoxin involved in the immobilization of prey. Our results are fundamental to address these insights further and to understand processes that drive venom evolution in dipterans as well as other arthropods.

## 1. Introduction

Venoms are key adaptations that have evolved on numerous occasions in animal lineages to serve a range of ecological roles including defense, predation, communication and competition [1,2,3]. Venoms constitute complex cocktails of proteins, peptides, salts and different organic molecules, collectively referred to as toxins [1,4]. These toxins are expressed in venom glands and delivered via venom ducts to structures that finally inject the composed venom from the venom delivery system via a wound into the prey. Proteins and peptides usually comprise the main venom components, and these have evolved from ancestral molecules with basic physiological ”every-day” functions into highly potent and chemically stable toxins [2,5]. However, the processes that drive this functional transition are understood only fragmentary [6,7,8]. Moreover, the majority of all known toxins from animal venoms can be classified into a limited number of structural classes [9]. This extreme level of convergence of venoms implies that toxins can provide insights into fundamental processes of protein functional evolution and biochemical adaptations.

Understanding convergent evolution requires a broad taxonomic sampling to accurately identify adaptive traits through comparative evolutionary studies [10]. However, only a few venomous lineages have so far been studied in-depth. Although a number of animal lineages previously considered potentially venomous have been described in recent years [3,11,12,13,14], the taxonomic sampling remains fragmented, particularly among venomous insect lineages. One of these venomous insect groups are robber or assassin flies (Asilidae), which are a species-rich family within one of the largest and most diverse insect groups, the flies (Diptera) [15,16]. Assassin flies have a worldwide distribution (except Antarctica) and comprise more than 500 genera including over 7000 species [17].

In contrast to most other dipterans, asilids are also predatory in their adult life stage [17], and have been known since the 19th century [18,19] as major predators of other arthropods including flies, beetles, grasshoppers, dragonflies, hymenopterans, and even spiders [20,21,22,23]. Their ecological and economic impact as predators is illustrated by their ability to significantly affect grasshopper populations, while other species that feed on wasps and bees are known to take out entire beehives [24,25]. Asilids are adapted to a predatory lifestyle on the wing with their slender but robust body in combination with a heavily sclerotized proboscis and a needle-like hypopharynx, large eyes and good flight capabilities [26,27]. The predation on larger or defensive prey, which is almost instantly paralyzed, led to early suspicions that robber flies utilize some kind of venom to overpower venomous or larger prey [18,19,28].

Early experiments by Whitfield showed that grasshoppers attacked by robber flies indeed died dramatically faster compared to grasshoppers stabbed in a similar way with needles [28]. In the same study, Whitfield also described two separate gland systems consisting of a pair of smaller labial glands and a pair of thoracic glands for the asilid *Machimus atricapillus*. Kahan (1964) tested for the proteolytic activity of extracts from the thoracic and labial glands and compared these to the activity of the stomach content of *Promachus griseiventris* and the stomach only of *Philonicus dorsiger* by injection experiments. These experiments concluded that neurotoxic activity was present in gland extracts due their paralyzing effects on the legs of locusts and mice. However, although these early experiments demonstrated toxic effects of asilid venoms, the general composition of the venom and the mechanisms by which it is secreted has remained unknown [18,29].

In the present study, we apply a combined transcriptomic and proteomic approach to provide detailed insight into the composition of the venom of two common European asilid species (*Eutolmus rufibarbis* and *Machimus arthriticus*) (Figure S1 (Supplementary File 1)). We also use high-resolution synchrotron micro computed tomography (SRµCT) to provide a characterization of the morphology of the venom system of asilids. Our results reveal that asilid venoms are not the protease-dominated venoms expected from liquid-feeding lineages, but that they are instead peptide-rich cocktails originating in the thoracic glands and expelled through an elaborate high-speed venom delivery system. Furthermore, we show that at least one of these peptides indeed plays a neurotoxic role. This peptide toxin likely assumes a fold common to spider and cone snail neurotoxins, illustrating the value of virtually unknown venomous lineages in identifying molecular adaptations through evolution-based structure-function relationships. Our results thus provide the foundation for understanding venom evolution in not only flies but also other venomous groups of insects through comparative studies. The high novelty of the putative toxins in robber fly venoms also highlights their potential as sources of new therapeutic and agrochemical approaches [30,31].

## 2. Results

### 2.1. Assembled Transcripts and Numbers of Assigned Coding Regions

In order to identify both putative toxins and their ancestral “house-keeping” variants, transcriptomes of body and venom gland tissue were sequenced on Illumina HiSeq 2000 platforms for both species, with gland tissue on 1/3 and body tissue on 1/4 Illumina channel. The resulting libraries showed almost equal magnitudes of raw and processed read numbers between the two different tissue types (Table 1). The contig numbers of cleaned assemblies vary slightly between species but show a larger number of contig sequences for the thoracic gland of *Machimus arthriticus* compared to *Eutolmus rufibarbis*.

The expression levels of transcripts based on coding regions identified with Transdecoder were assessed with two different methods, the read mapper Segemehl [32] and the RNA quantification tool Kallisto [33]. To prevent over-interpretation of our data, only coding regions that show a TPM (transcripts per million kilobase) value larger or equal 1 were included in our subsequent analyses. Both methods found overall similar results, which shows the robustness of our analyses considering that both methods use different approaches (Supplementary Files 2–5). However, for our data interpretation we rely on Segemehl as an established and precise read mapper [34] (Material and Methods).

### 2.2. Transcriptomic and Proteomic Profiles Differ in Thoracic Glands

**Figure 1:**
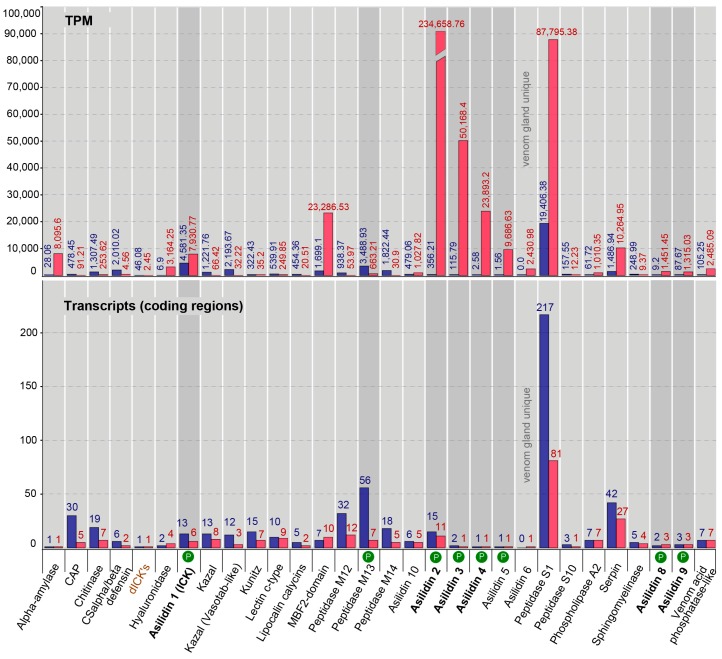
Comparative bar charts of 30 putative toxin protein classes from body and thoracic gland tissue from *Eutolmus rufibarbis*. Results from transcriptomics and proteomics (P) are summarized. The lower chart shows on the Y-axis the number of coding regions per protein class that passed the expression threshold. The relative expression level of each protein class in transcripts per million (TPM) in the putative venom gland (red) and in the body tissue (blue), is shown on the Y-axis in the upper part of the chart. The presence of the protein family in the proteome of the gland is marked with a white P in the green circle. Proteins that are present in transcriptomes and proteomes of both species are printed in bold, the two species unique protein classes are colored in brown.

**Figure 2:**
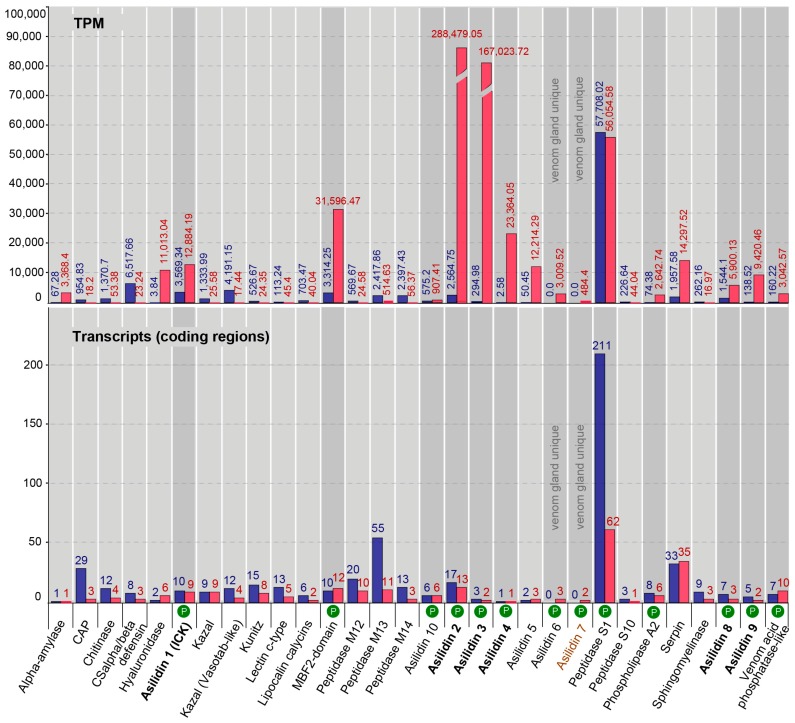
Comparative bar charts of 30 putative toxin protein classes from body and thoracic gland tissue from *Machimus arthriticus*. Results from transcriptomics and proteomics (P) are summarized. The lower chart shows on the Y-axis the number of coding regions per protein class that passed the expression threshold. The relative expression level of each protein class in transcripts per million (TPM) in the putative venom gland (red) and in the body tissue (blue), is shown on the Y-axis in the upper part of the chart. The presence of the protein family in the proteome of the gland is marked with a white P in the red circle. Proteins that are present in transcriptomes and proteomes of both species are printed in bold, the two species unique protein classes are colored in brown.

**Figure 3:**
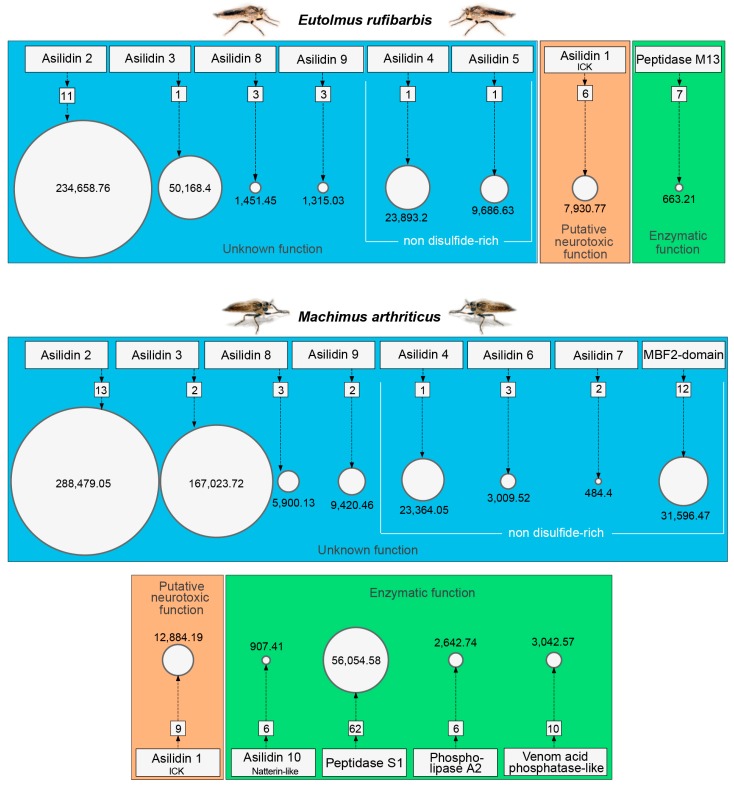
Bubble charts depicting the protein families secreted in the thoracic glands of *Eutolmus rufibarbis* and *Machimus arthriticus* and their expression levels. The number of assigned coding regions for each protein class is plotted in squares while related TPM values are plotted in circles. The size of the circle relates to the sum of all TPM values of all coding regions assigned to this protein class.

Our proteomic results revealed a major discrepancy between predicted venom components based on homology to other known toxins and relative expression levels of transcripts (transcriptomes) compared to the observed secreted proteins (proteomes) in the thoracic glands. For both species, the total numbers clearly differ between identified highly expressed or venom gland unique transcripts (16 for *E. rufibarbis* and 17 for *M. arthriticus*) and the number of finally secreted proteins in the crude venom cocktail. Surprisingly, the crude venoms of the two species also differed with regards to the proteomically detected putative toxin families. Only eight groups of secreted proteins were detected in the crude venom of *E. rufibarbis*, compared to 13 for *M. arthriticus* (Figure 1, Figure 2 and Figure 3, Figure S2 (Supplementary File 1)).

### 2.3. Similar Transcript Diversity but Different Expression Levels in Thoracic Gland and Body Tissue Transcriptomes

In total, we identified 31 putative venom protein classes that were expressed in the transcriptomes of the thoracic glands. 10 of these putative toxin families are novel, henceforth named Asilidin1–10 (Table 2), according to the rational nomenclature of centipede toxins [12,36]. Of these, 29 of the 31 proteins were expressed by both robber fly species. Both species expressed only one protein class that was uniquely expressed in the gland transcriptome but that was not identified in the other species: Double cysteine inhibitor knots (dICKs) were identified only in *E. rufibarbis* (but not in the venom proteome), while Asilidin7 was exclusive to *M. arthriticus*. However, both unique proteins are expressed at low levels, the dICKs with one transcript with 2.45 (TPM), the U_7_-Asilidin by two transcripts with 484.4 (TPM) (Figure 1 and Figure 2).

The relationship between the numbers of identified coding regions and their expression levels is very similar for both species and the thoracic gland and body tissue transcriptomes, respectively. The numbers of coding regions are generally higher for the body tissue transcriptomes. Exceptions for which the numbers of thoracic gland transcripts were higher are the hyaluronidase and the MBF2-domain-like proteins for both species and the venom acid phosphatase, Asilidin5, Asilidin6, Asilidin7 and serpin in *M. arthriticus*. For almost all identified putative venom proteins similar variants were also complementary recovered in the body tissue. Only one protein class without matching variants in the body tissue could be found in the glands of *E. rufibarbis* (Asilidin6, one transcript, TPM 484.4) while two protein classes were unique to the glands of *M. arthriticus* (Asilidin6 and Asilidin7).

The expression levels (TPM values) showed profound differences compared to the numbers of identified coding regions of putative toxins (Figure 1 and Figure 2). In contrast to the body tissue, 16 up-regulated protein classes for *E. rufibarbis*, and 17 higher expressed coding regions in *M. arthriticus* were identified for the thoracic gland (including Peptidase S1 which was slightly lower expressed compared to the body tissue from *M. arthriticus*). Major differences between gland transcripts of both species is the sequence of the three most highly expressed proteins (Figure 1 and Figure 2). Asilidin2 was the most highly expressed gland protein in both species with 234,658.76 (TPM) in *E. rufibarbis* and 288,479.05 (TPM) in *M. arthriticus*. Asilidin3 was the second most up-regulated protein in *M. arthriticus*, while it ranked third in *E. rufibarbis*. Peptidase S1, however, was the second highly up-regulated protein in *E. rufibarbis* while it was not present in *M. arthriticus*, (Figure 3). Except for peptidase S1 in *E. rufibarbis* all other proteins were also present in the crude venom of both species and supported by our proteomic data.

### 2.4. Anatomy of the Venom Delivery System

**Figure 4:**
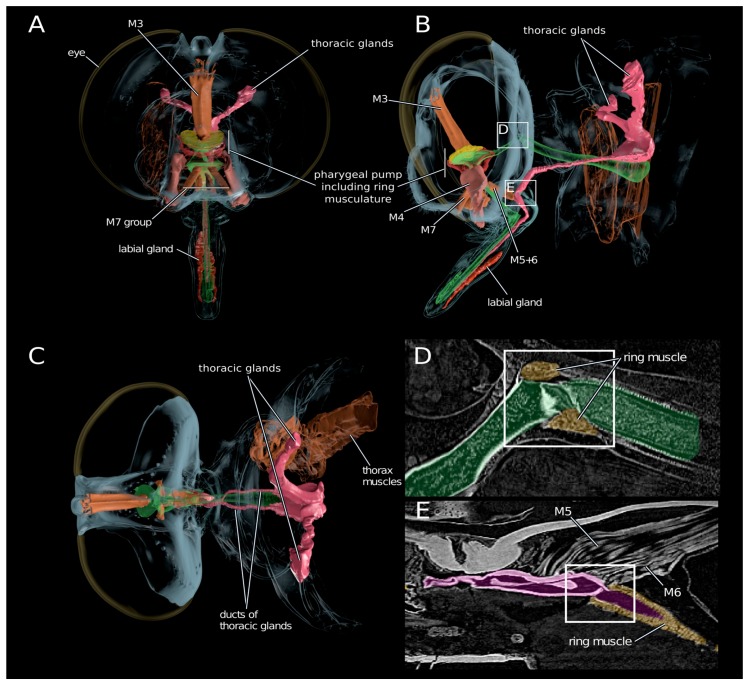
3D reconstruction of the gland and head morphology in *E. rufibarbis.* Reconstruction based on synchrotron micro-computed tomography data. (**A**) Frontal view; (**B**) Lateral view; (**C**) Dorsal view; (**D**,**E**) Images of the original scan data showing the digestive tract with the ring musculature to close the pharyngeal tract (**D**) and the musculature attaching at the salivary pump system (**E**). Note the “closed” position of the valve at the center of the white frame.

The thoracic gland system (magenta colored, Figure 4) is composed of a pair of elongated sac-like glands located in the dorsal parts of the first and second thoracic segments. Each gland opens into a separate duct (Figure 4C), which fuse ventrally of the food channel just before entering the head capsule. The single duct continues ventral of the food channel into a salivary pump composed of a non-return valve-like mechanism and two associated muscles (M5+6, Figure 4B) responsible for opening the valve. This salivary pump is attached to the base of the hypopharynx and consists of two plates, one for M6 attachment and another one to which muscle M5 attaches. The single duct continues further after the salivary pump and opens into the hypopharynx near the apex of the proboscis. The hypopharynx is indirectly moved by the strong paired expansor muscle M4 which is attached to lateral apophyses of the basipharynx on both sides. The pharyngeal pump is operated by a strong ring or sphincter muscle engulfing the pharynx and a dorsal expansor which attaches to the roof of the pharynx. The volume of the cibarium is controlled by the muscle group M7 (Figure 4).

Our results support descriptions by Whitfield (1925) that the labial glands (Figure 4) open into the lumen between the theca and the labium near the apex of the proboscis. They are therefore structurally separated from the hypopharynx at the tip of the proboscis where the duct of the thoracic glands opens into the lumen of the hypopharynx. Since the hypopharynx is the only structure entering the prey (Whitfield 1925) and an elaborate pumping apparatus is missing, we conclude that these labial glands are not part of the venom production system. Additionally, the structure of the labial glands and their location are variable within asilids [26]. It was suggested that they secret a lubricant that facilitates the mechanical sting process and their opening between the theca and the labium supports this idea.

### 2.5. Both Species Exhibit Similar Venom Proteomes

The venoms of both *E. rufibarbis* and *M. arthriticus* are dominated by a few highly expressed, novel proteins with unknown function. In *M. arthriticus* these are Asilidin2–4 and Asilidin6–10, while Asilidin2 and Asilidin3 show a similar pattern in *E. rufibarbis.* Asilidin2–5, Asilidin8 and Asilidin9 were also detected in the venom of *E. rufibarbis*, however showed lower relative expression than in *M. arthriticus* (Figure 1 and Figure 2). For both species two forms of cysteine-rich single von Willebrand factory type C (SVWFC) domain-containing peptides are expressed within the group without known function (Asilidin8 and Asilidin9).

A lower expressed integrant comprises proteins with enzymatic function. For *M. arthriticus* this fraction was composed of peptidase S1, phospholipase A2, venom acid phosphatase-like proteins and a low expressed, putative natterin-like protease known from a toadfish [37,38,39]. Despite its high expression level (third rank) peptidase S1 is not detected in the proteome of *E. rufibarbis*. The only detected enzymatic component in its gland system was a low expressed metalloprotease M13.

**Figure 5:**
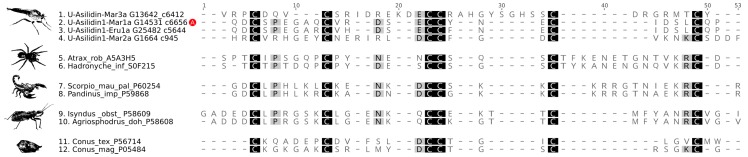
Sequence alignment of the mature Asilidin1 sequences identified in the proteome and transcriptome of the glands from *M. arthriticus* and *E. rufibarbis*. Mature peptide sequences of known and activity-tested ICK’s were included from venom of the cone snails *Conus textile* and *Conus magus*, the assassin bugs *Isyndus obscurus* and *Agriosphodrus dohrni*, the funnel web spiders *Hadronyche infensa* and *Atrax robustus* and the scorpions *Scorpio palmatus* and *Pandinus imperator*. The cysteine residue backbone shared by all sequences is highlighted in yellow. The red circle with a white A marks the activity tested, synthesized ICK sequence.

Putative neurotoxic peptides with a cysteine inhibitor knot (ICK)-like structure (Asilidin1) [40] constitute another integral venom part. These peptides were identified in both gland proteomes of *M. arthriticus* and *E. rufibarbis*, and exhibit a cysteine scaffold typical of ICK peptides, which constitute the bulk of the molecular diversity and ion channel modulating in spider venoms (Figure 5).

### 2.6. Neurotoxic Activity of U-Asilidin_1_-Mar1a

**Figure 6:**
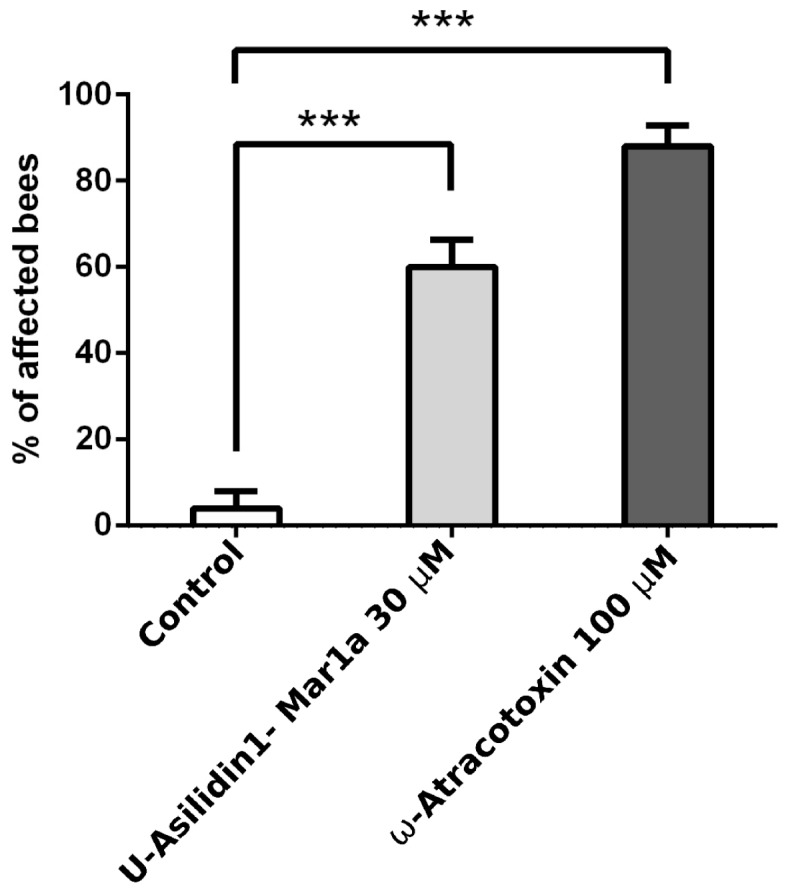
Neurotoxicity of Mar1a. Injection of 30 μM of Mar1a produced significant neurotoxic effects in bees (60% of injected bees were affected, see Supplementary File 6), compared to the negative control (MilliQ water, Supplementary File 6), including slow movements, disorientation and paralysis. The overall effect observed with Mar1a was comparable to our positive control, the potent insectidal spider toxin ω-atracotoxin (100 μM, >80% of bees were affected, Supplementary File 6). This bar graph represents the average of 5 experiments (5 bees per experiment), error bars represent S.E.M. and *** indicates the results were significantly different from control (*p* < 0.001).

In order to test the functional convergence between venom ICKs present in asilid and other venoms, the peptide corresponding to the mature peptide encoded by the highest expressed Asilidin1 transcript, U-Asilidin_1_-Mar1a (henceforth Mar1a) (Figure 6 and Figure S3 (Supplementary File 1)) was synthesized and injected into *Apis mellifera*. This revealed a clear neurotoxic effect on honeybees, including slow movements, disorientation and paralysis, similar to that of the positive control group treated with the potent insectidal spider toxin ω-atracotoxin from *Hadronyche versuta* [41,42] (Supplementary File 6).

## 3. Discussion

This study shows that robber flies (Asilidae) feature a complex venom delivery system and secrete venom from their thoracic venom glands. Like most venoms, asilid venoms consist of non-disulfide-rich peptides, disulfide-rich peptides novel proteins of unknown function, and enzymes. Contrary to our expectations for liquid-feeding predators, however, asilid venoms appear to be largely devoid of enzymatic proteins and instead consist of a large fraction of novel peptides and proteins. Moreover, we show that one of these peptides, Mar1a, produces neurotoxicity effects in the ecologically relevant prey species, the honey bee.

### 3.1. Highly Expressed Novel Proteins

Novel peptides and proteins with unknown function are by far most abundantly recovered by transcriptomics and proteomics in both species (Figure 3). This group comprises over 97% (*E. rufibarbis*) and 87% (*M. arthriticus*) of the TPM values assigned to all secreted putative toxins. Novel putative toxins are not unusual in proteomic/transcriptomic studies of poorly characterized venoms, such as in remipede crustaceans, where five unknown proteins accounted for ~15% of all FPKM values [43,44]. However, asilids seem to contain an unusually high percentage of unknown venom proteins. This is unexpected given that some dipterans such as *Drosophila* and *Aedes* are well studied model organisms of which several genomes have been sequenced and annotated. Without potential homologues outside the *E. rufibarbis* and *M. arthriticus* no speculation about the origin or putative function of these proteins seems feasible (Table 2 and Figures S4–S8 (Supplementary File 1)).

### 3.2. Asilid Venoms Contain Putative ICK Neurotoxins

While previous studies have observed neurotoxic activity of crude gland extracts [18,29,45], we here show that asilid venoms do indeed contain neurotoxic peptide toxins. This protein family, Asilidin1, is characterized by a cysteine pattern that is strongly suggestive of a cystine inhibitor knot (ICK) structural motif, which is defined by an antiparallel β sheet stabilized by a cystine knot. The cystine knot consists of a ring formed by two disulfide bonds and their intervening sections of the peptide backbone that is pierced by a disulfide bond that generally stabilizes the C-terminal region of the peptide. The ICK is probably the most widely recruited peptide fold in animal venoms, and ICK-like toxins are known from the venom of cone snails, scorpions, spiders and assassin bugs [9]. The neighbor network of Asilidin1 splits in to three distinct clades (Figure S3 (Supplementary File 1)). One clade includes U-Asilidin_1_-Mar2a, which is a unique sequence exclusively present in the thoracic glands with no matches in the body tissue. The other two clades also comprise identical body tissue sequences, although the expression values between gland sequence and body tissue sequence differ substantially. The most highly expressed peptides U-Asilidin_1_-Mar1a and U-Asilidin_1_-Eru1a are around 3000 times higher expressed in the thoracic glands compared to their respective body tissues. Activity test of a manually synthesized Mar1a protein showed motor effects in honey bees, which suggests Asilidin1 represents a family of neurotoxic ICK toxins (Figure 5 and Supplementary File 6).

### 3.3. Missing “Usual Suspect” Enzymes in Putative Asilid Venom?

Enzymes are known as important venom components in a vast number of venomous predators such as snakes, cephalopods, centipedes, assassin bugs, stinging insects and remipede crustaceans. Several classes of enzymatic proteins are often abundant in venoms, such as chitinases or serine proteinases, particularly in liquid-feeding arthropods where they likely play an important role in pre-digestion of prey [12,13,14,44]. Interestingly, the venom of *M. arthriticus* and *E. rufibarbis* differs from other liquid-feeding venomous insect predators by being largely devoid of enzymes. Although the transcriptome data of both species show high expression levels of M13 and S1 proteases as well as PLA2 and acid phosphatase (see Figure 1, Figure 2 and Figure 3), our proteomic analyses indicate that these enzymes constitute only minor components in the crude venom. This is in contrast to previous studies, which assumed that asilids secrete venom composed of proteolytic and neurotoxic activity [18,29,45]. Dipterans are generally known to digest orally by refluxing digestive enzymes from their gut to achieve extra oral digestion, a strategy applied in different variants also in other predatory arthropods [46,47]. Earlier studies showed that the stomach of the tested asilids shows higher proteolytic activity but also a larger range of pH-values in which enzymatic activity is observed [18]. Our proteomic results are in accordance with these findings.

### 3.4. Scenario for Envenomation of Prey by Asilids

**Figure 7:**
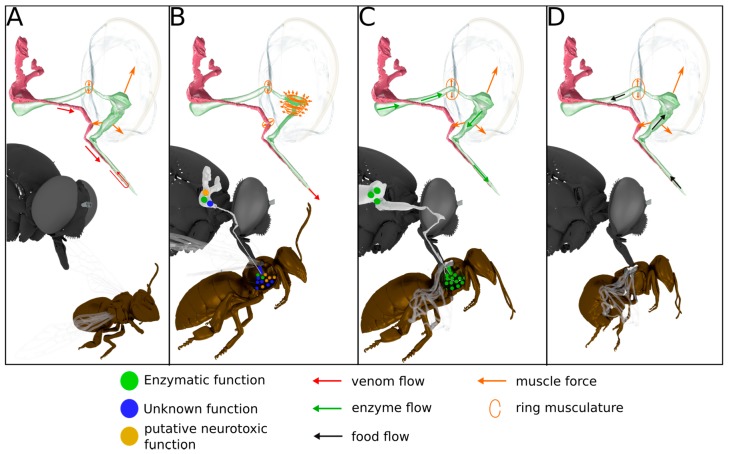
Envenomation and feeding process in asilids. Summary of the hypothesized feeding process for robber flies. (**A**) Closed valve of the digestive tract, open valve of the thoracic glands, increase of the pharyngeal pump volume leads to transport of the venom from the thoracic glands to the hypopharynx; (**B**) Closed valve of digestive tract, closed valve of the thoracic glands, decrease of the pharyngeal pump volume leads to powerful injection of the venom via the hypopharynx; (**C**) Open valve of the digestive tract, increase of the pharyngeal pump volume, transport of enzymes from the digestive tract to the hypopharynx and injection in the paralyzed prey; (**D**) Open valve of the digestive tract, increase of the pharyngeal pump volume, feeding on the liquefied prey contents.

Based on our proteomic and activity test result, and in combination with morphological data derived from SRµCT, we are now able to draw a more precise and partly new picture of the envenomation process in asilids. Envenomation begins with the transport of the secreted venom including the neurotoxic Mar1a into the lumen of the hypopharynx (Figure 7). This is on the one hand achieved by the salivary pump muscles but might be supported through an increase of the cibarial and pharyngeal lumen. Since the musculature directly inserting at the different parts of the gland system is minimal and only serves to control the non-return valve of the salivary pump, the larger volume changes of the cibarial pump are needed to effectively transport a larger fraction of venom into the prey with the first sting. Once the venom is loaded into the lumen of the hypopharynx, it can be injected in a second step against the hemolymph pressure of the prey through contraction of the ring musculature of the pharyngeal and cibarial pump. At the same time, the ring musculature of the salivary duct and the alimentary canal is contracted in order to prevent unwanted backflow. Literature data on the prey capture process indicates that prey is paralyzed within seconds and this could not be achieved with a passive inflow of saliva into the prey [18,28]. The prey’s inner compartments are then liquefied in a third step presumably by pumping stomach liquids through the proboscis into the lumen of the prey. The fourth and last step is the uptake of food. The liquefied body tissue is sucked up by contraction of M3 and M7 muscles which again increases the lumen of the cibarial and pharyngeal pump and thereby creates a negative pressure with respect to the lumen of the prey (Figure 4 and Figure 7).

### 3.5. Implications in the Context of Fly and Insect Venom Evolution

**Figure 8:**
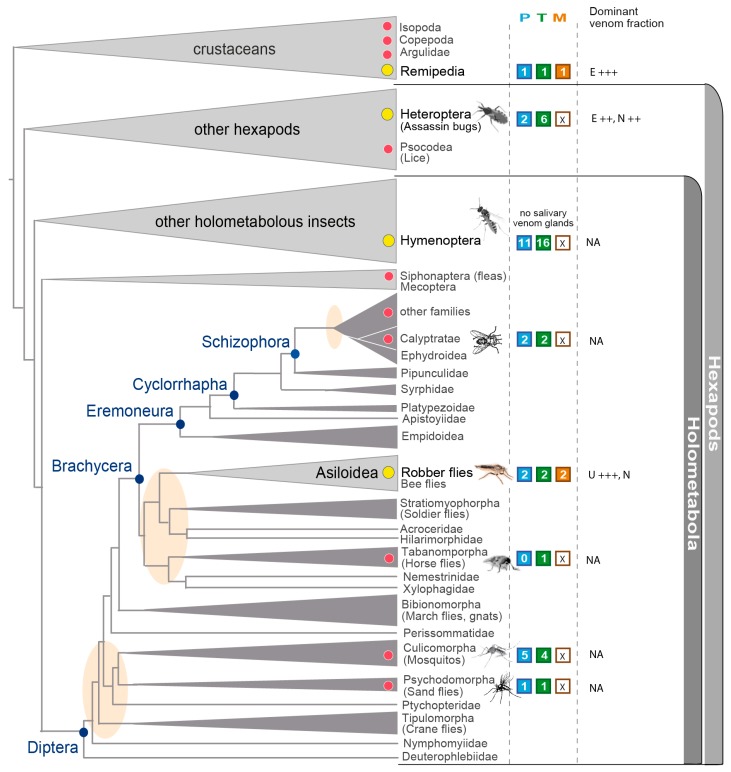
Current picture of venomics studies and phylogenetic implications for dipteran and insect venom evolution. The number of species that are covered by studies applying modern transcriptomic, proteomic and morphologic analyses are shown in the colored squares. Blue: Proteomics; Green: Transcriptomics; Brown: Reconstructions of the venom delivery system. Dominant venom fractions are roughly summarized with E: Enzymes; N: Neurotoxins; U: Unknown proteins. The plus sign indicates overexpressed fractions in the venom. Yellow circles indicate venomous predatory lineages, red circles hematophagous groups. Only studies based on Illumina transcriptome data and proteomic profiles of complete venom gland were included. See also a general overview on available salivary/venom gland transcriptomes of arthropods until 2014 in von Reumont et al., 2014 (Toxins) [4]. The tree is based on Wiegmann et al., 2011 and Misof et al., 2014 [16,55]. The branches do not reflect precise distances. Oval circles in beige indicate disputed branching events, blue dots represent major clades in Diptera.

Many dipteran groups have convergently evolved a hematophagous life style and are thus per definition also venomous. Due to their role as disease vectors and their impact on humans or live-stock, mosquitoes and tabanid species have received increased attention [48,49,50,51,52,53,54] (Figure 8). Surprisingly, however, even for these comparably well-studied groups, only a few species have been covered by recent—omics approaches or detailed morphological studies using modern technology (Figure 8) [4]. This situation hinders detailed comparative analyses of the evolution of venoms and the morphological dynamics of venom delivery systems within flies. Our study comprehensively describes the venom system of asilids, which, unlike most other dipterans, are a truly predatory lineage. Venom is not restricted to blood feeding females, as is the case in hematophagous dipterans, but occurs in both genders. Blood feeding evolved early in dipterans, long before the rapid radiation in the more basal groups of Brachycera to which asilids belong (Figure 8). The results from the present study support an independent evolution of asilid venom in which a separate suite of proteins and peptides compared to those of hematophagous lineages were functionally recruited as toxins. Reflecting this independent evolution of a predatory venom, the asilid the venom delivery system appears highly adapted to high-speed predation and envenomation that is facilitated by a derived muscle system.

Diptera is one of the three groups within insects besides hymenopterans and heteropterans that is known to feature venomous species, however, their venoms remain largely uncharacterized. Yet, in the quest to better understand venom evolution in insects in a larger framework, dipterans play an eminent role as the youngest insect group, having evolved in the ending Jurassic period around 150 million years ago [55]. Moreover, the venoms studied here indicate asilid venoms differ substantially from other venomous liquid feeding insect lineages. Future studies on a taxonomically wider sample may therefore provide insight into the key processes that govern toxin evolution [56].

## 4. Conclusions

This study provides a detailed description of the venom system of robber flies (Asilidae). Their venom is produced in the thoracic glands, and injected through a complex venom delivery system that agrees well with their agile, predatory lifestyle. Surprisingly, their venoms appear to be largely devoid of enzymatic proteins and instead consist of a large fraction of novel peptides and proteins. This high degree of molecular novelty suggests dipteran-specific groups of proteins and peptides were recruited as toxins into asilid venom. Moreover, we show that one of these peptides, Mar1a, produces neurotoxicity effects in honey bees, suggesting the other, novel peptide families may also harbor neurotoxic activities. Due to the attractiveness of peptides as easily synthesized compounds with a high degree of pharmacological potency and selectivity, asilid venoms therefore appear to be a good source of molecular tools and potential lead molecules for development into therapeutic and agrochemical products [30]. Our results thus provide the foundation for future studies to not just help understand mechanisms of toxin recruitment and evolution within and outside dipteran arthropods, but also mine this source of novel, bioactive tools and potential lead molecules.

## 5. Materials and Methods

### 5.1. Specimen Collection and Determination

In total 40 individuals of common robber fly species *Eutolmus rufibarbis* (25 specimens) and *Machimus arthriticus* (15 specimens) were collected in 2014 near Altenrath, Germany, (Figure S1 (Supplementary File 1)). Species were morphologically determined using the identification key by Fritz Geller-Grimm [57]. For each species one voucher sample was stored in 96% Ethanol. Additionally, barcodes of the mitochondrial cytochrome c oxidase subunit I (COI) were sequenced for all specimens except for two in Bouin solution sampled individuals (Supplementary Figure S9). DNA was extracted following standard procedure with the NucleoSpin^®^ Tissue Kit (MACHERY-NAGEL, Düren, Germany). PCR was performed with the primers LCO1490F and HCO2198R [57]. PCR products were cleaned with the NucleoSpin^®^ Gel and PCR Clean-up Kit (MACHERY-NAGEL, Düren, Germany). Sequencing was performed at GATC Biotech AG (Konstanz, Germany). Barcode sequences are accessible in GeneBank (NCBI) with accession numbers KY485001–KY485038.

### 5.2. Specimen Dissection and Sample Preservation

For transcriptome sequencing thoracic glands of 6 *Machimus arthriticus* and 10 *Eutolmus rufibarbis* specimens were immediately dissected on ice in RNAse free TBE buffer and water. From three of those individuals, for each species body tissue samples (muscle tissue) were preserved to analyze complementary paralog or ancestral variants of venom proteins from body tissue. All samples were stored in RNAlater at −4 °C. To preserve crude venom for proteomics analyses 8 glands from *Machimus arthriticus* and 12 glands of *Eutolmus rufibarbis* were dissected on ice and squeezed out in sterile proteinase inhibitor buffer. The proteinase inhibitor buffer was prepared following the manufacturer protocol (Complete Ultra, ROCHE, Mannheim, Germany). The venom extracts were then lyophilized and stored at −20 °C until proteomic analysis. Remaining parts of all dissected specimens were preserved as vouchers in 96% ethanol. Two specimens of the more frequent *Eutolmus rufibarbis* were stored in Bouin solution and critical point dried for later synchrotron-based micro-computer tomography (SRµCT) reconstruction.

### 5.3. RNA Extraction, Transcriptome Sequencing and Assembly

RNA-Extraction (Trizol method), construction of cDNA libraries (Illumina TruSeq kit, San Diego, CA, USA and sequencing was performed at the BGI Sequencing facility (Beijing, China), using the Illumina^®^ HiSeq 2000 platform with 100 bp paired end. Gland tissue samples were sequenced on one-third, body tissue samples on one-fourth Illumina lane. All data of venom gland and body tissue transcriptomes are accessible in GeneBank under the BioProject PRJNA361480, SRA accession numbers: SRR5185499, SRR5185498, SRR5185497, SRR5185496 and TSA entries: GFGA00000000, GFFZ00000000, GFZR00000000, GFZQ00000000.

Raw reads were pre-processed and quality checked before assembly. First, all raw reads were visually inspected to check for overall quality and for overexpressed sequences using FastQC v0.11.2 [58]. Afterwards, Trimmomatic v0.33 [59] was applied to exclude low quality reads below a phred value of 32 (sliding window size 4, HEADCROP 10, minimum length 50 bp). A modified and extended template file with known adapter and vector sequences was used to screen and to remove adapter and vector contaminations. Reads were assembled using Trinity v2.0.2 [35] applying standard settings, except for a transcript minimum length of 101.

### 5.4. Assessing Coding Regions and Expression Levels for Transcripts

Coding regions within the assembled transcripts were identified with TransDecoder v3.0.1 [60]. To include shorter putative toxins such as neurotoxins, the minimal open reading frame length was set to 40 amino acids. BlastP v.2.4 search against the UniProt database [61] and HMMscan v.3.1 [62] against the Pfam database [63] were performed to provide additional information for the identification of potential protein coding regions. In the case that several open reading frames per transcript are equally likely, TransDecoder retains all open reading frames with the same likelihood for this transcript.

To assess the expression level or abundance of the identified coding regions and transcripts, two slightly different approaches were chosen, first the read mapper Segemehl v.0.2.0 [32], and second the RNASeq quantification tool Kallisto v.0.43.1 [33]. Both approaches result in a normalized expression value for all transcripts. Segemehl was used to map the reads back to the coding region predicted with TransDecoder. In contrast to other read mappers, segemehl reports multiple hits for a read if several alignments are equally likely and fulfill the set alignment parameters. This strategy in combination with the restriction to map reads only for identified coding regions gives the most accurate approximation of expression levels for putative toxins. Segemehl was applied with 95 percent accuracy and the default setting for all other options.

After mapping, the number of mapped reads was normalized to account for the length of the coding region and for the number of overall mapped reads. All coding regions with a TPM smaller than 1 were excluded for subsequent analyses, similar to [64,65]. As an alternative approach to calculate the abundance of transcripts, the RNA-seq quantification program Kallisto was used (Table 1). As an input, the processed paired end libraries were provided. Kallisto calculates the normalized expression value TPM, which takes the library size and the effective length of a transcript into account. Is it important to note, that Kallisto uses an approximation of effective length of every transcript for the calculation of the TPM, while in the Segemehl approach the complete length of the coding region is used for length normalization. To minimize the false positive aligned reads, the bootstrap value was set to 100.

### 5.5. Identification of Venom Protein Classes and Putative Toxins via Transcriptomics

**Figure 9:**
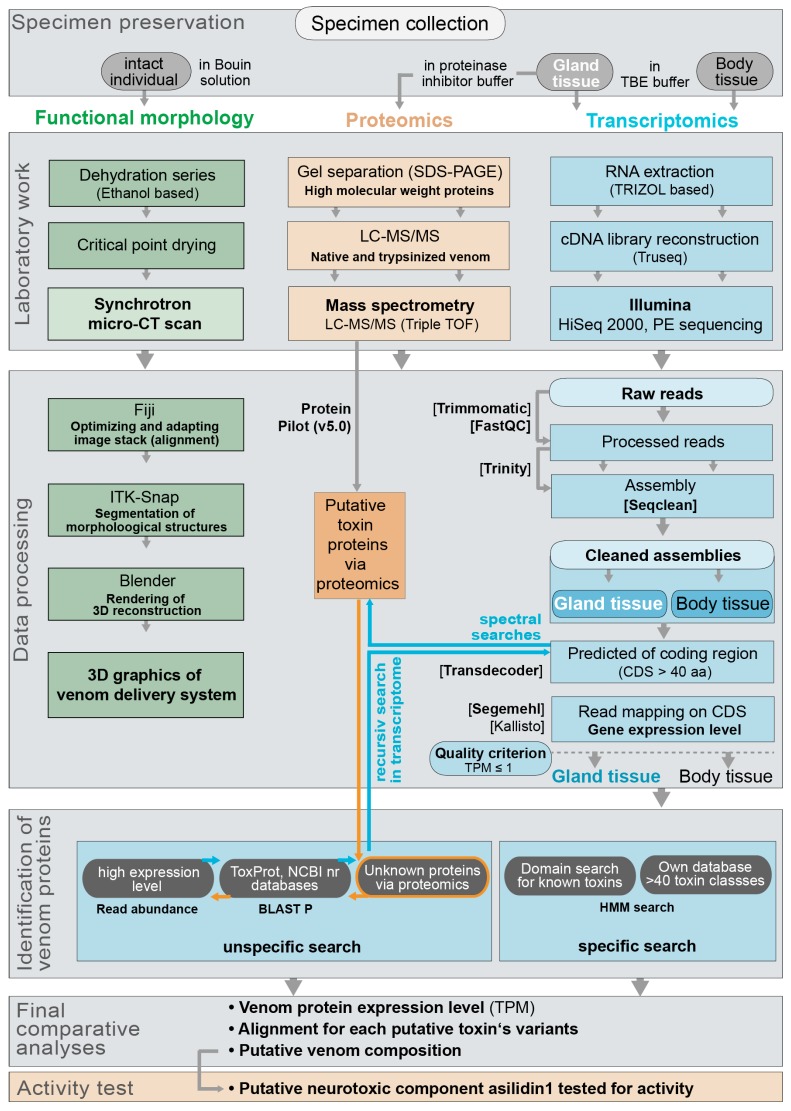
Workflow that was developed and applied in this study.

To search unspecific for protein families of putative toxins and noticeable high expressed proteins in the toracic glandsystem, BlastP searches (*e*-value 10^−4^) against the Toxprot Database [66] were carried out with the protein sequences of the coding regions. Additionally, hmmer 3 [62] was used to perform hmm searches with an own, customized and hand curated alignment database of over 40 known venom proteins (Supplementary Files 2–5). The alignments to train hmm models were compiled with sequences from the UniProt database, covering non-venomous and venomous species, following [3,13]. As a cut-off value a bit score of 20 was chosen, potential homologs with a lower bit score were excluded. Finally, the TPM values were linked to all identified coding regions that match venom protein classes or putative toxins for body tissue and putative venom gland tissue (Supplementary Files 2–5). Identified sequences were extracted by a customized python script and manually optimized and curated in Geneious R9. Finally, sequences were annotated with the InterProScan v.1.1.0 Plug-in Genieous R9 [67]. For all transcript sequences the presence of a signal peptide was tested using SignalP 4.1 [68]. This search strategy was performed for both venom gland and body tissue transcriptomes. Identical but also venom gland unique transcripts in body and venom gland tissue were identified using the CD-HIT-2D tool (sequence identity 0.99) of the CD-HIT software package v.4.6 [69] and additionally by visual inspection of alignments in Geneious R9 (Figure 9 and Supplementary File 7).

### 5.6. Sample and Data Processing for Proteome Analyses

Lyophilized venom in protease inhibitor was dissolved in water to a concentration of 1 mg/mL by repeated pipetting, before 20 µg protein was precipitated by addition of 5:1 volume ratio of −20 °C acetone, incubation for 1 h at −20 °C, and centrifugation at 20,000 rcf for 20 min at 0 °C. The acetone was then removed, the pellet washed (ice cold absolute ethanol), centrifuged, and the ethanol removed (pipetting and air drying). Protein was then dissolved to a concentration of 5 mg/mL in 4 M urea, 15% acetonitrile (ACN), 100 mM ammonium bicarbonate, before cystines were reduced by incubation with 5 mM dithiothreitol at 60 °C for 5 min and then alkylated with 10 mM iodoacetamide at 37 °C for 60 min. The reduced and alkylated sample was then digested by incubating with 20 ng/µL trypsin overnight at 37 °C in 2 M urea 10% ACN 100 mM ammonium bicarbonate. The digested sample was desalted using a C18 ZipTip (Thermo Fisher, Waltham, MA, USA) and dried prior to LC-MS/MS analysis using a vacuum centrifuge.

The digested protein was dissolved in 0.5% formic acid (FA) and 2 µg analyzed on an AB Sciex 5600 TripleTOF equipped with a Turbo-V source heated to 550 °C. Venom was fractionated on a Shimadzu (Kyoto, Japan) Nexera UHPLC with an Agilent Zorbax stable-bond C18 column (2.1 × 100 mm, 1.8 μm particle size, 300 Å pore size), using a flow rate of 180 µL/min and a gradient of 1–40% solvent B (90% ACN 0.1% FA) in 0.1% FA over 60 min. MS1 survey scans were acquired at 300–1800 *m*/*z* over 250 ms, and the 20 most intense ions with a charge of +2 to +5 and an intensity of at least 120 counts/s were selected for MS2. The unit mass precursor ion inclusion window mas ±0.7 Da, and isotopes within ±2 Da were excluded from MS2, which scans were acquired at 80–1400 *m*/*z* over 100 ms and optimized for high resolution.

For protein identification, MS/MS spectra were searched against the translated combined venom gland and body transcriptomes using ProteinPilot v5.0 (AB Sciex, Framingham, MA, USA). Searches were run as thorough identification searches, specifying urea denaturation, tryptic digestion and cysteine alkylation by iodoacetamide. Amino acid substitutions and biological modifications were allowed in order to identify potential post translational modifications and to account for chemical modifications due to experimental artefacts. Decoy-based false discovery rates (FDR) was estimated by ProteinPilot v5.0, and only protein identifications with a corresponding local FDR of <0.5% were considered significant. Spectra were also examined manually to further eliminate any false positives.

### 5.7. Chemical Synthesis of U-Asilidin_1_-Mar1a

All solvents used were of HPLC grade. DMF, DIEA, ACN, TIS, TFA, piperidine and all other reagents were obtained from Sigma-Aldrich (St. Louis, MO, USA) or Merck (Kenilworth, NJ, USA) and were used without further purification. Fmoc amino acid derivatives, HATU and 2-Chorotrityl chloride resin (100–200 Mesh, 1.44 mmol CL/g resin) were purchased from Iris Biotech (Marktredwitz, Germany). AmphiSpheres 20 HMP resin (0.6 mmol/g 75–150 µm) was purchased from Agilent Technologies (Les Ulis, France). Peptides were manually synthesized using Fmoc-based solid-phase peptide synthesis (SPPS). All Fmoc amino acids and HATU were dissolved in N,N-dimethylformamide (DMF) to reach 0.5 M. Fmoc deprotection was performed with 20% piperidine in DMF twice (1 min at room temperature), then the resin was washed three times with DMF. Each Fmoc-protected amino acid (5 eq) was coupled twice in the presence of HATU (5 eq) and DIEA (10 eq) in DMF at room temperature for two min. After completion of the coupling reaction, the resin was sequentially washed twice with DMF. Cleavage of peptide from the resin and removal of side-chain protecting groups were carried out using 10 mL of a solution containing TFA/triisopropylsilane/H_2_O (95:2.5:2.5, *v*/*v*/*v*) for 2 h 30 min at room temperature. Then, the resin was removed by filtration and washed three times with DCM. DCM and TFA were removed under vacuum and cold diethyl ether was added to precipitate the peptide, followed by two steps of centrifugation and removal of the supernatant. Crude peptide was purified by preparative RP-HPLC, and their purity were confirmed by LC/ESI-MS. Monoisotopic mass of folded Mar1a was confirmed by MALDI-HRMS analysis.

2-Cl-(Trt)-NHNH_2_ was prepared from 2-Chorotrityl chloride resin (100–200 Mesh, 1.44 mmol CL/g resin) [70]. Segment 1 of the Mar1a peptide was synthetized on 250 mg of 2-Cl-(Trt)-NHNH_2_ resin, whereas segment 2 of the Mar1a peptide was synthetized on 250 mg of HMPA-Pro-Fmoc resin. After cleavage from the resin and treatments, peptides were purified by preparative RP-HPLC. Elution fractions of the targeted peaks were pooled and lyophilized. The homogeneity of purified peptides was assessed by analytical RP-HPLC and ESI-MS confirmed the correct mass with an observed *m*/*z* of 819.5 Da for [M + 2H]^2+^ (calculated *m*/*z* 819.33 Da) for segment 1 and an observed *m*/*z* of 745.8 Da for [M + 2H]^2+^ (calculated *m*/*z* 745.77 Da), see Figure 1 activity. Overall, 24.4 mg of a white solid were obtained for the first segment (yield of 7.46%) and 12 mg for the second (yield = 5.37%) (Figure S10 (Supplementary File 1)).

Native Chemical Ligation between segment 1 and segment 2 was carried out with 9.83 mg (6 µmol) of segment 1 and 8.97 mg (6 µmol) of segment 2 [70]. After formation of a single major product, the peptide was purified by preparative RP-HPLC. Eluted fractions of the targeted mass were pooled and lyophilized. The homogeneity of purified peptide was assessed by analytical RP-HPLC and ESI-MS confirmed the correct mass with an observed *m*/*z* of 1033.6 Da for [M + 3H]^3+^ (calculated *m*/*z* 1033.12 Da). 4 mg (yield = 21.53%) of a white solid were obtained (Figure S11 (Supplementary File 1)).

Folding of linear Mar1a peptide (3 mg, 0.97 µmol, 1 eq) was carried out by stirring in the presence of GSH (5.96 mg, 19.39 µmol, 20 eq) and GSSG (1.19 mg, 1.94 µmol, 2 eq) into a solution of 0.1 M Tris-HCl (pH 8.0, 19.41 mL) at room temperature for 16 h. After formation of a single major product, the peptide was purified by preparative RP-HPLC. Elution fraction of the targeted peak was pooled and lyophilized. The homogeneity of purified peptide was assessed by analytical RP-HPLC. ESI-MS: observed *m*/*z* 1031 for [M + 3H]^3+^ (calcd *m*/*z* 1031.12). 1.58 mg (yield = 52.67%) of a white solid were obtained. MALDI-HRMS: observed *m*/*z* 3089.12 (monoisotopic mass) for [M + H]^+^ (calcd *m*/*z* 3089.14) (Figure S12 (Supplementary File 1)).

### 5.8. HPLC Purification and Activity Tests of U-Asilidin_1_-Mar1a 1a with Bioassay

Preparative RP-HPLC was run on a Gilson PLC 2250 Purification system (Villiers le bel, France) instrument using a preparative column (Waters DeltaPak C18 Radial-Pak Cartridge, 100 Å, 40 × 100 mm, 15 μm particle size, flow rate 50.0 mL/min). Buffer A was 0.1% TFA in water, and buffer B was 0.1% TFA in acetonitrile. Analytical analyses were carried out using a Chromolith (Fontenay sous Bois, France) Flash 25 × 4.6 mm C18 reversed-phase column. A flow rate of 3 mL/min and a gradient of 0–100% B over 2.5 min were used. Eluent A: water/0.1% HCO_2_H; eluent B: acetonitrile/0.1% FA. UV detection was performed at 214 nm.

Domestic honeybees (*Apis mellifera*) were caught at the hive entrance and placed at 4 °C prior to injection. 30 μM Mar1a, 100 μM ω-atracotoxin (positive control) or MilliQ water (negative control) were injected into the median ocellus. To perform the injection, the lens of the median ocellus was perforated with a pulled borosilicate-glass capillary. Using a second capillary, 1 μL of toxin or control solution was aspirated and 200 nL were injected per bee (5 individual per experiment). Following the injection, bees were placed in a petri dish containing a small cup filled with water + honey solution. Behavioral observations were recorded for 60 min and typical toxin effects included slow movements, disorientation and paralysis. The results were analyzed with Prism (GraphPad, San Diego, CA, USA) using a one-way analysis of variance (ANOVA) (Figure 6, Supplementary File 6).

### 5.9. Morphological Work and Data Processing

The anatomy of specimens was investigated using synchrotron micro-Computer Tomography (SRµCT) [66]. Prior to scanning, samples were critical point dried (Samdri-PVT-3D) and mounted on beamline-specific specimen holders. Generally, X-ray imaging has a high penetrating power and allows visualization of large specimens without the need for sectioning. SRµCT offers a true 3D spatial resolution of up to 1 μm with moderate resolving power of tissues and tissue interfaces. Specimens were scanned at the Swiss Light Source electron synchrotron accelerator (SLS, Villigen, Switzerland; Stampanoni et al., 2010). The SLS X-ray sources were optimized for high-density and spatial resolution (1–10 um) imaging with monochromatic X-rays. The raw data are available upon request from the corresponding author.

Segmentation and rendering of single structures was carried out using ITK-snap v3.6.0 [71] and Blender v2.78 [72]. Both software packages are distributed under the General Public License. The terminology for asilid musculature and other described structures is based on Owsley (1946).

## Figures and Tables

**Table 1:** Library size (numbers raw and processed reads), assembled contig sequences and numbers of identified coding regions for the analyzed species *Eutolmus rufibarbis* and *Machimus arthriticus*. Library size (processed reads) refers to the read numbers that survive the processing (trimming, adapter removing and filtering of quality scores) before they were assembled with Trinity [35]. The final contig numbers are given (contigs of cleaned assembly) for each transcriptome assembly after screening and trimming the contigs versus remaining adaptor and contaminating sequences (VecScreen), see also material and methods (Figure 9).

Species	Tissue	Library Size (Raw Reads)	Library Size (Processed Reads)	Contigs of Cleaned Assembly	Extracted Coding Regions	Coding Regions (TPM ≥ 1, Kallisto)	Coding Regions (TPM ≥ 1, Segemehl)
*Eutolmus rufibarbis*	Thoracic gland	108,632,880	87,187,856	56,640	33,218	16,049	15,523
	Body tissue	64,751,420	50,968,759	70,281	42,919	32,920	28,827
*Machimus arthriticus*	Thoracic gland	106,668,752	83,421,201	69,849	41,816	17,798	15,346
	Body tissue	64,651,716	44,208,096	67,504	42,784	34,478	30,629

**Table 2:** Novel proteins and peptides identified in the proteomic analyses of the thoracic glands of *E. rufibarbis* and *M. arthriticus*. Proteomic hits specify the number of transcripts matching the fragments from mass spectrometry for each peptide and protein. P-Distance-based networks are provided in Figures S3–S8 (Supplementary File 1) for proteins with more than two sequences.

Protein Family	Structural Fold	Scaffold	Number of Residues	Molecular Weight (Average)	Proteome Hits *E. rufibarbis*	Proteome Hits *M. arthriticus*
Asilidin1	ICK	Cx_3-6_Cx_5-9_CCx_3-10_Cx_4-6_C	51–65	6.2 kDa	1	3
Asilidin2	unknown	Cx_35_Cx_8-13_Cx_36-39_C	267–339	33.7 kDa	5	8
Asilidin3	unknown	Cx_7-8_Cx_22_Cx_14_C	88–104	11.36 kDa	1	2
Asilidin4	unknown	no cysteine scaffold	86	18.47 kDa	1	1
Asilidin5	unknown	no cysteine scaffold	273	30.2 kDa	1	0
Asilidin6	unknown	no cysteine scaffold, two P rich domains	115	13.12 kDa	0	2
Asilidin7	unknown	no cysteine scaffold	139	15.43 kDa	0	1
Asilidin8	SVWC-domain	Cx_16_Cx_4_Cx_10_Cx_7_Cx_14_CCx_5_C	101	11.29 kDa	1	1
Asilidin9	SVWC-domain	Cx_22_Cx_4_Cx_10_Cx_8_Cx_12_CCx_4_C	118–119	13.5 kDa	1	1
Asilidin10	Natterin-like	no cysteine scaffold, P rich domain	146–226	20.38 kDa	0	2

## Supplementary Materials

The following are available online at www.mdpi.com/2072-6651/10/1/29/s1, Supplementary File 1: Summary and description of the identified enzymatic components, with a short discussion of the putative functions in the robber fly venom. Figure S1: Pictures of the analyzed species and the habitat, where specimens were captured, Figure S2: SDS-PAGE gel of crude venom of *Machimus arthriticus* and *Eutolmus rufibarbis*, Figure S3: Neighbor Networks for the Asilidin1, Figure S4: Neighbor Networks for the Asilidin2, Figure S5: Neighbor Networks for the Asilidin3, Figure S6: Neighbor Networks for the Asilidin8, Figure S7: Neighbor Networks for the Asilidin9, Figure S8: Neighbor Networks for the Asilidin10, Figure S9: CO1 barcode alignment, Figure S10: Overall strategy for synthesis of U-Asilidin_1_-Mar1a, Figure S11: UV chromatogram of linear U-Asilidin_1_-Mar1a, Figure S12: UV chromatogram of folded U-Asilidin_1_-Mar1a, Supplementary File 2: Summarizing table of the identified classes of putative toxins for the thoracic gland system transcriptome of *Eutolmus rufibarbis*. The TPM values for each identified coding region are shown, the presence of a signal peptide and presence of proteomic data is marked, Supplementary File 3: Summarizing table of the identified classes of putative toxins for the body tissue transcriptome of *Eutolmus rufibarbis*. The TPM values for each identified coding region are shown, the presence of a signal peptide is marked, Supplementary File 4: Summarizing table of the identified classes of putative toxins for the thoracic gland system transcriptome of *Machimus arthriticus*. The TPM values for each identified coding region are shown, the presence of a signal peptide and presence of proteomic data is marked, Supplementary File 5: Summarizing table of the identified classes of putative toxins for the body tissue transcriptome of *Machimus arthriticus*. The TPM values for every identified coding region are shown, the presence of signal peptides is marked, Supplementary File 6: Video of observed effects of U-Asilidin_1_-Mar1a, ω-atracotoxin and negative control on honey bees based on the bioassay of the synthesized U-Asilidin_1_-Mar1a, Supplementary File 7: Protein alignments of all identified classes of putative toxins. For each class the coding regions identified in the thoracic gland system and the body tissue of both robber flies are aligned together with toxins known from other species. For some classes the alignments were cut to align only the domains, in these cases the name of the pruned alignment is marked with “cut”.
