# Supplementary material for: A Dipteran’s Novel Sucker Punch: Evolution of Arthropod Atypical Venom with a Neurotoxic Component in Robber Flies (Asilidae, Diptera)
Source: Toxins (Basel). 2018 Jan 5;10(1):29. doi: 10.3390/toxins10010029 (PMC5793116; doi:10.3390/toxins10010029)
Supplement: Supplementary file 1 [file toxins-10-00029-s001.zip › toxins-254261-supplementary/toxins-254261 supplementary file 2.pdf]

Protein Class	Contig ID	TPM - Segemehl	TPM - Kallisto	Signal Peptide	false positive
**Aegyptin**	no hit	0	0	X	
**Alpha-amylase**	Gene.160538::c53848_BT_Eu_ru_202_g1_i1::g.160538::m.160538	28.06	15.70	NO	
**Anophelin**	no hit	0	0	X	
**Apyrase**	Gene.25975::c5296_BT_Eu_ru_202_g1_i1::g.25975::m.25975	34.75	18.79	YES	only body tissue
	Gene.21888::c4723_BT_Eu_ru_202_g1_i1::g.21888::m.21888	5.87	2.99	YES	only body tissue
	Gene.8970::c2180_BT_Eu_ru_202_g1_i2::g.8970::m.8970	4.11	2.65	NO	only body tissue
	Gene.8964::c2180_BT_Eu_ru_202_g1_i1::g.8964::m.8964	4.26	0.00	NO	only body tissue
	Gene.37803::c6641_BT_Eu_ru_202_g1_i1::g.37803::m.37803	1.80	0.85	NO	only body tissue
	Gene.7814::c1896_BT_Eu_ru_202_g1_i1::g.7814::m.7814	1.59	2.22	NO	only body tissue
	Gene.118845::c12003_BT_Eu_ru_202_g1_i1::g.118845::m.118845	1.16	0.83	NO	only body tissue
**CAP**	Gene.142078::c36088_BT_Eu_ru_202_g1_i1::g.142078::m.142078	123.82	99.62	NO	
	Gene.29010::c5715_BT_Eu_ru_202_g1_i1::g.29010::m.29010	46.38	30.19	YES	
	Gene.68277::c8738_BT_Eu_ru_202_g1_i1::g.68277::m.68277	25.28	14.40	YES	
	Gene.68281::c8738_BT_Eu_ru_202_g1_i2::g.68281::m.68281	21.70	2.98	YES	
	Gene.24328::c5068_BT_Eu_ru_202_g1_i1::g.24328::m.24328	30.38	23.13	YES	
	Gene.86439::c9557_BT_Eu_ru_202_g1_i2::g.86439::m.86439	15.79	8.95	YES	
	Gene.86434::c9557_BT_Eu_ru_202_g1_i1::g.86434::m.86434	18.66	2.72	NO	
	Gene.86446::c9557_BT_Eu_ru_202_g1_i3::g.86446::m.86446	18.66	2.72	NO	
	Gene.86452::c9557_BT_Eu_ru_202_g1_i4::g.86452::m.86452	18.66	2.72	NO	
	Gene.17713::c4168_BT_Eu_ru_202_g1_i1::g.17713::m.17713	6.77	4.15	NO	
	Gene.51425::c7670_BT_Eu_ru_202_g1_i1::g.51425::m.51425	11.23	7.79	YES	
	Gene.61359::c8312_BT_Eu_ru_202_g1_i2::g.61359::m.61359	10.63	8.17	NO	
	Gene.14160::c3373_BT_Eu_ru_202_g1_i1::g.14160::m.14160	6.16	4.72	NO	
	Gene.164007::c55725_BT_Eu_ru_202_g1_i1::g.164007::m.164007	24.83	20.83	NO	
	Gene.24682::c5122_BT_Eu_ru_202_g1_i3::g.24682::m.24682	8.57	3.22	NO	
	Gene.41734::c6971_BT_Eu_ru_202_g1_i2::g.41734::m.41734	9.37	4.10	NO	
	Gene.24677::c5122_BT_Eu_ru_202_g1_i2::g.24677::m.24677	9.31	4.87	NO	
	Gene.41731::c6971_BT_Eu_ru_202_g1_i1::g.41731::m.41731	9.87	6.39	NO	
	Gene.51430::c7670_BT_Eu_ru_202_g2_i1::g.51430::m.51430	5.34	3.82	YES	
	Gene.61362::c8312_BT_Eu_ru_202_g1_i3::g.61362::m.61362	9.39	2.50	YES	
	Gene.5269::c1271_BT_Eu_ru_202_g1_i1::g.5269::m.5269	4.31	3.27	YES	
	Gene.16488::c3975_BT_Eu_ru_202_g1_i1::g.16488::m.16488	3.86	3.01	YES	
	Gene.1341::c294_BT_Eu_ru_202_g1_i1::g.1341::m.1341	20.87	82.26	NO	
	Gene.6394::c1542_BT_Eu_ru_202_g1_i1::g.6394::m.6394	3.38	1.99	YES	
	Gene.144484::c37151_BT_Eu_ru_202_g1_i1::g.144484::m.144484	3.35	2.71	NO	
	Gene.18178::c4228_BT_Eu_ru_202_g1_i2::g.18178::m.18178	1.79	1.37	YES	
	Gene.137298::c30369_BT_Eu_ru_202_g1_i1::g.137298::m.137298	1.59	1.05	NO	
	Gene.6399::c1542_BT_Eu_ru_202_g1_i2::g.6399::m.6399	2.78	1.30	NO	
	Gene.155148::c47202_BT_Eu_ru_202_g1_i1::g.155148::m.155148	1.21	0.82	NO	
	Gene.131222::c24156_BT_Eu_ru_202_g1_i1::g.131222::m.131222	4.52	8.29	NO	
**Chitinase**	Gene.160944::c53991_BT_Eu_ru_202_g1_i1::g.160944::m.160944	697.10	429.41	NO	
	Gene.239::c49_BT_Eu_ru_202_g1_i1::g.239::m.239	297.69	167.80	YES	
	Gene.11803::c2822_BT_Eu_ru_202_g1_i1::g.11803::m.11803	46.20	13.56	YES	
	Gene.11820::c2822_BT_Eu_ru_202_g1_i2::g.11820::m.11820	46.20	13.56	YES	
	Gene.34358::c6288_BT_Eu_ru_202_g1_i3::g.34358::m.34358	43.27	25.37	NO	
	Gene.34337::c6288_BT_Eu_ru_202_g1_i1::g.34337::m.34337	44.71	0.65	YES	
	Gene.34347::c6288_BT_Eu_ru_202_g1_i2::g.34347::m.34347	44.71	0.65	YES	
	Gene.36885::c6543_BT_Eu_ru_202_g1_i1::g.36885::m.36885	37.34	24.06	YES	
	Gene.97115::c9977_BT_Eu_ru_202_g2_i2::g.97115::m.97115	8.17	4.05	NO	
	Gene.97106::c9977_BT_Eu_ru_202_g2_i1::g.97106::m.97106	6.02	0.91	NO	
	Gene.42077::c7007_BT_Eu_ru_202_g1_i1::g.42077::m.42077	13.63	8.11	YES	
	Gene.54349::c7879_BT_Eu_ru_202_g1_i1::g.54349::m.54349	8.19	5.39	NO	
	Gene.20380::c4520_BT_Eu_ru_202_g1_i1::g.20380::m.20380	1.97	1.06	NO	
	Gene.20387::c4520_BT_Eu_ru_202_g1_i2::g.20387::m.20387	2.40	1.06	NO	
	Gene.97101::c9977_BT_Eu_ru_202_g1_i1::g.97101::m.97101	4.02	2.86	YES	
	Gene.137357::c30413_BT_Eu_ru_202_g1_i1::g.137357::m.137357	1.94	1.07	NO	
	Gene.142979::c36402_BT_Eu_ru_202_g1_i1::g.142979::m.142979	1.49	0.67	NO	
	Gene.121828::c14411_BT_Eu_ru_202_g1_i1::g.121828::m.121828	1.31	0.95	NO	
	Gene.143585::c36683_BT_Eu_ru_202_g1_i1::g.143585::m.143585	0.83	0.94	NO	
	Gene.120961::c13578_BT_Eu_ru_202_g1_i1::g.120961::m.120961	1.14	3.92	NO	
	Gene.121952::c14548_BT_Eu_ru_202_g1_i1::g.121952::m.121952	0.70	1.22	NO	
	Gene.124223::c17424_BT_Eu_ru_202_g1_i1::g.124223::m.124223	0.76	0.82	NO	
	Gene.150616::c43630_BT_Eu_ru_202_g1_i1::g.150616::m.150616	0.49	1.15	NO	
	Gene.128806::c21766_BT_Eu_ru_202_g1_i1::g.128806::m.128806	0.70	0.00	NO	
	Gene.125377::c19352_BT_Eu_ru_202_g1_i1::g.125377::m.125377	0.43	0.00	NO	
	Gene.167864::c59991_BT_Eu_ru_202_g1_i1::g.167864::m.167864	0.26	1.99	NO	
**Conoporin-like**	no hit	0	0	X	
**Csalpha/beta defensin**	Gene.7845::c1908_BT_Eu_ru_202_g1_i1::g.7845::m.7845	1890.74	4577.47	YES	
	Gene.23018::c4863_BT_Eu_ru_202_g1_i1::g.23018::m.23018	97.37	138.40	YES	
	Gene.23019::c4863_BT_Eu_ru_202_g1_i1::g.23019::m.23019	10.13	13.59	YES	
	Gene.165312::c56906_BT_Eu_ru_202_g1_i1::g.165312::m.165312	9.13	12.16	NO	
	Gene.128161::c21304_BT_Eu_ru_202_g1_i1::g.128161::m.128161	1.63	3.04	NO	
	Gene.129389::c22261_BT_Eu_ru_202_g1_i1::g.129389::m.129389	1.03	1.90	NO	
	Gene.121567::c14148_BT_Eu_ru_202_g1_i1::g.121567::m.121567	0.84	2.45	NO	
**Cystatin**	no hit	0	0	X	
**dICK's**	Gene.20180::c4485_BT_Eu_ru_202_g1_i2::g.20180::m.20180	46.08	40.85	YES	
**Disintegrine-like**	no hit	0	0	X	
**Echotoxin-like**	no hit	0	0	X	
**Gigantoxin-like**	no hit	0	0	X	
**Hyaluronidase**	Gene.87474::c9599_BT_Eu_ru_202_g1_i1::g.87474::m.87474	5.49	3.11	NO	
	Gene.132022::c25143_BT_Eu_ru_202_g1_i1::g.132022::m.132022	1.41	1.87	NO	
**ICK's (Asilidin1)**	Gene.161697::c54329_BT_Eu_ru_202_g1_i1::g.161697::m.161697	2444.80	1424.27	NO	
	Gene.21715::c4697_BT_Eu_ru_202_g1_i1::g.21715::m.21715	1264.43	4732.57	YES	
	Gene.93542::c9829_BT_Eu_ru_202_g1_i1::g.93542::m.93542	283.54	561.23	YES	

	Gene.152240::c45535_BT_Eu_ru_202_g1_i1::g.152240::m.152240	224.96	360.66	NO	
	Gene.39881::c6812_BT_Eu_ru_202_g1_i2::g.39881::m.39881	121.48	187.70	YES	
	Gene.20180::c4485_BT_Eu_ru_202_g1_i2::g.20180::m.20180	46.08	40.85	YES	
	Gene.39880::c6812_BT_Eu_ru_202_g1_i1::g.39880::m.39880	107.00	153.16	YES	
	Gene.1638::c383_BT_Eu_ru_202_g1_i1::g.1638::m.1638	41.46	74.42	YES	
	Gene.122087::c14689_BT_Eu_ru_202_g1_i1::g.122087::m.122087	36.06	60.63	NO	
	Gene.24204::c5054_BT_Eu_ru_202_g1_i1::g.24204::m.24204	31.67	74.15	YES	
	Gene.105934::c10281_BT_Eu_ru_202_g1_i4::g.105934::m.105934	18.94	46.23	NO	
	Gene.131101::c24008_BT_Eu_ru_202_g1_i1::g.131101::m.131101	3.57	24.69	NO	
	Gene.145664::c37908_BT_Eu_ru_202_g1_i1::g.145664::m.145664	2.15	6.30	NO	
	Gene.133178::c26839_BT_Eu_ru_202_g1_i1::g.133178::m.133178	1.29	6.33	NO	
**Kazal**	Gene.18128::c4219_BT_Eu_ru_202_g1_i1::g.18128::m.18128	955.03	2059.49	YES	
	Gene.160344::c53759_BT_Eu_ru_202_g1_i1::g.160344::m.160344	151.82	151.40	NO	
	Gene.73660::c9012_BT_Eu_ru_202_g1_i1::g.73660::m.73660	25.55	14.73	NO	excluded
	Gene.60::c14_BT_Eu_ru_202_g1_i2::g.60::m.60	43.69	20.87	YES	excluded
	Gene.54::c14_BT_Eu_ru_202_g1_i1::g.54::m.54	42.02	6.10	YES	excluded
	Gene.38877::c6728_BT_Eu_ru_202_g2_i1::g.38877::m.38877	9.21	5.29	NO	excluded
	Gene.119989::c12764_BT_Eu_ru_202_g1_i1::g.119989::m.119989	65.89	134.17	NO	
	Gene.153754::c46278_BT_Eu_ru_202_g1_i1::g.153754::m.153754	4.44	2.64	NO	
	Gene.77851::c9219_BT_Eu_ru_202_g1_i3::g.77851::m.77851	2.92	1.01	NO	excluded
	Gene.104004::c10221_BT_Eu_ru_202_g1_i2::g.104004::m.104004	9.76	4.66	NO	
	Gene.104009::c10221_BT_Eu_ru_202_g1_i4::g.104009::m.104009	9.76	4.66	NO	
	Gene.77840::c9219_BT_Eu_ru_202_g1_i2::g.77840::m.77840	3.00	0.88	NO	excluded
	Gene.104007::c10221_BT_Eu_ru_202_g1_i3::g.104007::m.104007	8.58	2.85	NO	
	Gene.38488::c6702_BT_Eu_ru_202_g1_i1::g.38488::m.38488	4.06	2.14	NO	
	Gene.38494::c6702_BT_Eu_ru_202_g1_i2::g.38494::m.38494	3.64	0.68	NO	
	Gene.161553::c54262_BT_Eu_ru_202_g1_i1::g.161553::m.161553	1.33	1.12	NO	excluded
	Gene.51196::c7651_BT_Eu_ru_202_g1_i2::g.51196::m.51196	3.07	3.33	NO	excluded
	Gene.51194::c7651_BT_Eu_ru_202_g1_i1::g.51194::m.51194	3.21	0.00	YES	excluded
	Gene.142522::c36239_BT_Eu_ru_202_g1_i1::g.142522::m.142522	3.72	9.25	NO	
	Gene.119945::c12736_BT_Eu_ru_202_g1_i1::g.119945::m.119945	1.58	1.73	NO	
	Gene.21472::c4674_BT_Eu_ru_202_g1_i1::g.21472::m.21472	2.13	5.42	YES	
	Gene.121155::c13763_BT_Eu_ru_202_g1_i1::g.121155::m.121155	1.54	4.42	NO	
	Gene.131547::c24540_BT_Eu_ru_202_g1_i1::g.131547::m.131547	1.00	1.18	NO	
	Gene.134151::c28539_BT_Eu_ru_202_g1_i1::g.134151::m.134151	0.96	4.31	NO	
	Gene.132595::c25933_BT_Eu_ru_202_g1_i1::g.132595::m.132595	1.41	7.20	NO	
	Gene.149143::c41436_BT_Eu_ru_202_g1_i1::g.149143::m.149143	0.70	1.31	NO	
	Gene.166132::c57797_BT_Eu_ru_202_g1_i1::g.166132::m.166132	0.59	0.73	NO	
	Gene.124573::c17955_BT_Eu_ru_202_g1_i1::g.124573::m.124573	0.41	0.97	NO	
	Gene.130853::c23721_BT_Eu_ru_202_g1_i1::g.130853::m.130853	0.38	0.79	NO	
	Gene.150391::c43220_BT_Eu_ru_202_g1_i1::g.150391::m.150391	0.24	0.00	NO	
**Kazal (vasotab-like)**	Gene.27039::c5453_BT_Eu_ru_202_g1_i1::g.27039::m.27039	459.25	295.38	YES	
	Gene.27307::c5494_BT_Eu_ru_202_g1_i1::g.27307::m.27307	989.21	2483.01	YES	
	Gene.1621::c376_BT_Eu_ru_202_g1_i2::g.1621::m.1621	75.42	32.86	YES	excluded
	Gene.1608::c376_BT_Eu_ru_202_g1_i1::g.1608::m.1608	73.78	9.96	YES	excluded
	Gene.116928::c11046_BT_Eu_ru_202_g1_i1::g.116928::m.116928	268.79	221.45	NO	
	Gene.119724::c12575_BT_Eu_ru_202_g1_i1::g.119724::m.119724	258.96	464.41	NO	
	Gene.73991::c9035_BT_Eu_ru_202_g1_i1::g.73991::m.73991	105.07	80.74	YES	
	Gene.83158::c9419_BT_Eu_ru_202_g1_i1::g.83158::m.83158	19.53	3.93	YES	
	Gene.83168::c9419_BT_Eu_ru_202_g1_i3::g.83168::m.83168	17.10	5.88	YES	
	Gene.26194::c5324_BT_Eu_ru_202_g1_i1::g.26194::m.26194	20.40	18.32	NO	
	Gene.127974::c21185_BT_Eu_ru_202_g1_i1::g.127974::m.127974	30.61	73.03	NO	
	Gene.83162::c9419_BT_Eu_ru_202_g1_i2::g.83162::m.83162	19.50	6.46	NO	
	Gene.1339::c293_BT_Eu_ru_202_g1_i1::g.1339::m.1339	3.67	4.55	YES	
	Gene.119945::c12736_BT_Eu_ru_202_g1_i1::g.119945::m.119945	1.58	1.73	NO	
	Gene.131547::c24540_BT_Eu_ru_202_g1_i1::g.131547::m.131547	1.00	1.18	NO	
	Gene.27331::c5499_BT_Eu_ru_202_g2_i1::g.27331::m.27331	0.90	1.86	YES	
	Gene.138131::c31103_BT_Eu_ru_202_g1_i1::g.138131::m.138131	0.74	1.85	NO	
**Kunitz**	Gene.100222::c10088_BT_Eu_ru_202_g1_i1::g.100222::m.100222	62.67	35.18	NO	
	Gene.108787::c10380_BT_Eu_ru_202_g1_i1::g.108787::m.108787	16.83	8.72	NO	
	Gene.108822::c10380_BT_Eu_ru_202_g1_i2::g.108822::m.108822	15.78	0.43	NO	
	Gene.83719::c9444_BT_Eu_ru_202_g1_i2::g.83719::m.83719	23.11	12.70	NO	excluded
	Gene.83672::c9444_BT_Eu_ru_202_g1_i1::g.83672::m.83672	21.88	0.40	NO	excluded
	Gene.58792::c8135_BT_Eu_ru_202_g1_i3::g.58792::m.58792	19.81	10.23	NO	
	Gene.58781::c8135_BT_Eu_ru_202_g1_i2::g.58781::m.58781	19.66	0.00	NO	
	Gene.63943::c8462_BT_Eu_ru_202_g1_i3::g.63943::m.63943	4.51	2.18	NO	
	Gene.63891::c8462_BT_Eu_ru_202_g1_i1::g.63891::m.63891	4.38	0.25	NO	
	Gene.63918::c8462_BT_Eu_ru_202_g1_i2::g.63918::m.63918	4.46	0.00	NO	
	Gene.143192::c36491_BT_Eu_ru_202_g1_i1::g.143192::m.143192	56.97	85.46	NO	
	Gene.3414::c866_BT_Eu_ru_202_g1_i1::g.3414::m.3414	48.97	76.95	YES	
	Gene.145823::c38016_BT_Eu_ru_202_g1_i1::g.145823::m.145823	34.30	46.65	NO	
	Gene.18876::c4298_BT_Eu_ru_202_g1_i1::g.18876::m.18876	15.10	10.96	NO	
	Gene.156979::c48848_BT_Eu_ru_202_g1_i1::g.156979::m.156979	11.22	19.23	NO	
	Gene.126709::c20527_BT_Eu_ru_202_g1_i1::g.126709::m.126709	4.72	5.29	NO	
	Gene.7602::c1818_BT_Eu_ru_202_g1_i1::g.7602::m.7602	3.07	4.84	YES	
	Gene.152895::c45821_BT_Eu_ru_202_g1_i1::g.152895::m.152895	0.51	0.00	NO	
	Gene.140458::c33756_BT_Eu_ru_202_g1_i1::g.140458::m.140458	0.57	2.61	NO	
**Lectin c-type**	Gene.23041::c4869_BT_Eu_ru_202_g1_i1::g.23041::m.23041	430.71	303.27	YES	
	Gene.52516::c7754_BT_Eu_ru_202_g1_i3::g.52516::m.52516	26.32	13.84	YES	
	Gene.32805::c6140_BT_Eu_ru_202_g1_i1::g.32805::m.32805	12.07	4.75	NO	excluded
	Gene.32820::c6140_BT_Eu_ru_202_g1_i2::g.32820::m.32820	12.02	1.20	NO	excluded
	Gene.10507::c2495_BT_Eu_ru_202_g1_i1::g.10507::m.10507	25.83	22.96	YES	
	Gene.22191::c4762_BT_Eu_ru_202_g1_i1::g.22191::m.22191	7.74	3.65	NO	
	Gene.22194::c4762_BT_Eu_ru_202_g1_i2::g.22194::m.22194	7.72	1.05	NO	
	Gene.145637::c37888_BT_Eu_ru_202_g1_i1::g.145637::m.145637	21.38	17.12	NO	
	Gene.45117::c7223_BT_Eu_ru_202_g2_i1::g.45117::m.45117	7.03	3.94	NO	excluded
	Gene.15947::c3813_BT_Eu_ru_202_g1_i1::g.15947::m.15947	9.06	6.70	YES	
	Gene.15951::c3813_BT_Eu_ru_202_g1_i2::g.15951::m.15951	7.97	2.70	YES	
	Gene.127797::c21075_BT_Eu_ru_202_g1_i1::g.127797::m.127797	1.84	1.14	NO	
	Gene.22361::c4783_BT_Eu_ru_202_g1_i1::g.22361::m.22361	1.31	0.73	NO	excluded
	Gene.165742::c57351_BT_Eu_ru_202_g1_i1::g.165742::m.165742	1.34	0.77	NO	
**Lipocalin calycins**	Gene.17947::c4193_BT_Eu_ru_202_g1_i1::g.17947::m.17947	261.36	212.66	YES	
	Gene.10611::c2536_BT_Eu_ru_202_g1_i1::g.10611::m.10611	177.93	122.10	YES	
	Gene.143062::c36440_BT_Eu_ru_202_g1_i1::g.143062::m.143062	11.78	10.54	NO	
	Gene.1206::c260_BT_Eu_ru_202_g1_i1::g.1206::m.1206	1.75	1.68	YES	
	Gene.127831::c21094_BT_Eu_ru_202_g1_i1::g.127831::m.127831	1.55	1.08	NO	
**Maxadilan-like**	no hit	0	0	X	
**MBF2-domain**	Gene.36405::c6479_BT_Eu_ru_202_g1_i1::g.36405::m.36405	944.88	980.39	YES	
	Gene.153899::c46349_BT_Eu_ru_202_g1_i1::g.153899::m.153899	474.26	528.63	NO	

	Gene.134364::c28620_BT_Eu_ru_202_g1_i1::g.134364::m.134364	115.58	122.93	NO
	Gene.137891::c30881_BT_Eu_ru_202_g1_i1::g.137891::m.137891	61.64	54.13	NO
	Gene.127065::c20713_BT_Eu_ru_202_g1_i1::g.127065::m.127065	63.27	83.74	NO
	Gene.144921::c37410_BT_Eu_ru_202_g1_i1::g.144921::m.144921	33.10	49.49	NO
	Gene.155280::c47297_BT_Eu_ru_202_g1_i1::g.155280::m.155280	6.36	7.25	NO
**Melittin-like**	no hit	0	0	X
**Natterin-like (Asilidin10)**	Gene.31937::c6042_BT_Eu_ru_202_g1_i1::g.31937::m.31937	260.64	198.66	NO
	Gene.134183::c28558_BT_Eu_ru_202_g1_i1::g.134183::m.134183	116.57	80.13	NO
	Gene.48255::c7442_BT_Eu_ru_202_g1_i1::g.48255::m.48255	47.22	34.07	NO
	Gene.6341::c1532_BT_Eu_ru_202_g1_i2::g.6341::m.6341	22.58	5.83	NO
	Gene.6338::c1532_BT_Eu_ru_202_g1_i1::g.6338::m.6338	28.92	21.84	NO
	Gene.36870::c6540_BT_Eu_ru_202_g1_i2::g.36870::m.36870	3.13	2.83	NO
**Neurokinin-like**	no hit	0	0	X
**Nitrophorin-like**	no hit	0	0	X
**Novel protein (Asilidin2)**	Gene.11205::c2697_BT_Eu_ru_202_g1_i1::g.11205::m.11205	155.89	101.16	NO
	Gene.58349::c8110_BT_Eu_ru_202_g1_i1::g.58349::m.58349	111.60	77.43	YES
	Gene.50653::c7611_BT_Eu_ru_202_g1_i1::g.50653::m.50653	21.47	15.11	YES
	Gene.64818::c8521_BT_Eu_ru_202_g1_i1::g.64818::m.64818	18.57	11.77	YES
	Gene.11204::c2697_BT_Eu_ru_202_g1_i1::g.11204::m.11204	10.07	6.56	YES
	Gene.101340::c10126_BT_Eu_ru_202_g1_i1::g.101340::m.101340	9.51	6.09	NO
	Gene.106804::c10309_BT_Eu_ru_202_g1_i1::g.106804::m.106804	4.42	2.87	NO
	Gene.81563::c9358_BT_Eu_ru_202_g1_i1::g.81563::m.81563	5.49	3.02	YES
	Gene.127319::c20833_BT_Eu_ru_202_g1_i1::g.127319::m.127319	3.96	2.61	NO
	Gene.81448::c9352_BT_Eu_ru_202_g1_i1::g.81448::m.81448	3.38	2.15	YES
	Gene.81459::c9352_BT_Eu_ru_202_g3_i2::g.81459::m.81459	3.08	1.52	NO
	Gene.83601::c9439_BT_Eu_ru_202_g1_i2::g.83601::m.83601	2.99	2.03	YES
	Gene.83593::c9439_BT_Eu_ru_202_g1_i1::g.83593::m.83593	2.39	1.19	YES
	Gene.6366::c1537_BT_Eu_ru_202_g1_i1::g.6366::m.6366	1.57	0.83	NO
	Gene.128156::c21301_BT_Eu_ru_202_g1_i1::g.128156::m.128156	1.82	1.10	NO
**Novel protein (Asilidin3)**	Gene.141974::c36063_BT_Eu_ru_202_g1_i1::g.141974::m.141974	107.33	141.93	NO
	Gene.47305::c7377_BT_Eu_ru_202_g1_i1::g.47305::m.47305	8.46	7.37	YES
**Novel protein (Asilidin4)**	Gene.30032::c5847_BT_Eu_ru_202_g1_i1::g.30032::m.30032	2.58	5.68	YES
**Novel protein (Asilidin5)**	Gene.10676::c2552_BT_Eu_ru_202_g1_i1::g.10676::m.10676	1.56	1.32	NO
**Novel protein (Asilidin6)**	no hit	0	0	X
**Novel protein (Asilidin7)**	no hit	0	0	X
**Peptidase M12**	Gene.6965::c1644_BT_Eu_ru_202_g1_i1::g.6965::m.6965	399.30	269.12	YES
	Gene.99159::c10056_BT_Eu_ru_202_g1_i4::g.99159::m.99159	14.97	4.63	NO
	Gene.99111::c10056_BT_Eu_ru_202_g1_i1::g.99111::m.99111	14.46	1.83	NO
	Gene.100790::c10108_BT_Eu_ru_202_g1_i3::g.100790::m.100790	11.90	3.19	NO
	Gene.100773::c10108_BT_Eu_ru_202_g1_i2::g.100773::m.100773	12.13	3.42	NO
	Gene.92491::c9799_BT_Eu_ru_202_g1_i2::g.92491::m.92491	19.03	0.00	NO
	Gene.92552::c9799_BT_Eu_ru_202_g1_i8::g.92552::m.92552	19.03	0.00	NO
	Gene.92537::c9799_BT_Eu_ru_202_g1_i7::g.92537::m.92537	19.47	0.79	YES
	Gene.92564::c9799_BT_Eu_ru_202_g1_i9::g.92564::m.92564	18.64	1.58	NO
	Gene.54391::c7885_BT_Eu_ru_202_g1_i1::g.54391::m.54391	12.12	6.59	NO
	Gene.90464::c9714_BT_Eu_ru_202_g1_i1::g.90464::m.90464	53.79	27.97	NO
	Gene.92509::c9799_BT_Eu_ru_202_g1_i4::g.92509::m.92509	21.04	3.89	NO
	Gene.92576::c9799_BT_Eu_ru_202_g1_i10::g.92576::m.92576	21.36	0.00	NO
	Gene.99131::c10056_BT_Eu_ru_202_g1_i2::g.99131::m.99131	11.80	0.00	NO
	Gene.105558::c10269_BT_Eu_ru_202_g2_i1::g.105558::m.105558	11.79	6.39	NO
	Gene.100759::c10108_BT_Eu_ru_202_g1_i1::g.100759::m.100759	10.79	0.57	NO
	Gene.92525::c9799_BT_Eu_ru_202_g1_i6::g.92525::m.92525	18.93	2.16	NO
	Gene.92478::c9799_BT_Eu_ru_202_g1_i1::g.92478::m.92478	19.12	1.88	NO
	Gene.92502::c9799_BT_Eu_ru_202_g1_i3::g.92502::m.92502	19.12	1.88	NO
	Gene.92477::c9799_BT_Eu_ru_202_g1_i1::g.92477::m.92477	17.00	1.51	NO
	Gene.92524::c9799_BT_Eu_ru_202_g1_i6::g.92524::m.92524	17.85	3.92	NO
	Gene.90467::c9714_BT_Eu_ru_202_g1_i2::g.90467::m.90467	46.12	0.00	NO
	Gene.90469::c9714_BT_Eu_ru_202_g1_i3::g.90469::m.90469	46.12	0.00	NO
	Gene.90472::c9714_BT_Eu_ru_202_g1_i4::g.90472::m.90472	45.77	10.02	NO
	Gene.63553::c8441_BT_Eu_ru_202_g1_i5::g.63553::m.63553	6.08	0.21	NO
	Gene.49148::c7523_BT_Eu_ru_202_g1_i1::g.49148::m.49148	3.84	2.09	YES
	Gene.72647::c8958_BT_Eu_ru_202_g1_i1::g.72647::m.72647	5.10	2.72	YES
	Gene.85558::c9516_BT_Eu_ru_202_g1_i1::g.85558::m.85558	3.48	1.83	NO
	Gene.63496::c8441_BT_Eu_ru_202_g1_i1::g.63496::m.63496	4.99	0.80	NO
	Gene.63512::c8441_BT_Eu_ru_202_g1_i2::g.63512::m.63512	4.99	0.80	NO
	Gene.63529::c8441_BT_Eu_ru_202_g1_i3::g.63529::m.63529	4.99	0.80	NO
	Gene.32210::c6072_BT_Eu_ru_202_g1_i1::g.32210::m.32210	3.26	18.63	NO
	Gene.149978::c42610_BT_Eu_ru_202_g1_i1::g.149978::m.149978	0.47	0.79	NO
	Gene.166082::c57729_BT_Eu_ru_202_g1_i1::g.166082::m.166082	0.47	0.00	NO
	Gene.156932::c48814_BT_Eu_ru_202_g1_i1::g.156932::m.156932	0.56	2.40	NO
	Gene.140944::c34478_BT_Eu_ru_202_g1_i1::g.140944::m.140944	0.38	1.75	NO
**Peptidase M13**	Gene.86165::c9547_BT_Eu_ru_202_g1_i1::g.86165::m.86165	1731.75	995.14	NO
	Gene.86169::c9547_BT_Eu_ru_202_g2_i1::g.86169::m.86169	1035.26	980.11	YES
	Gene.108607::c10374_BT_Eu_ru_202_g1_i1::g.108607::m.108607	108.97	62.31	NO
	Gene.142273::c36158_BT_Eu_ru_202_g1_i1::g.142273::m.142273	80.02	43.98	NO
	Gene.143124::c36469_BT_Eu_ru_202_g1_i1::g.143124::m.143124	59.71	34.31	NO
	Gene.126367::c20418_BT_Eu_ru_202_g1_i1::g.126367::m.126367	50.95	31.26	NO
	Gene.112876::c10484_BT_Eu_ru_202_g1_i1::g.112876::m.112876	35.52	19.39	NO
	Gene.42633::c7061_BT_Eu_ru_202_g1_i1::g.42633::m.42633	52.95	31.67	NO
	Gene.57632::c8072_BT_Eu_ru_202_g1_i1::g.57632::m.57632	41.87	26.46	NO
	Gene.33434::c6200_BT_Eu_ru_202_g1_i2::g.33434::m.33434	22.36	10.11	NO
	Gene.33423::c6200_BT_Eu_ru_202_g1_i1::g.33423::m.33423	21.64	2.92	NO
	Gene.134741::c28778_BT_Eu_ru_202_g1_i1::g.134741::m.134741	19.82	11.19	NO
	Gene.112740::c10480_BT_Eu_ru_202_g1_i1::g.112740::m.112740	19.95	11.26	NO
	Gene.37736::c6634_BT_Eu_ru_202_g1_i1::g.37736::m.37736	18.98	11.17	YES
	Gene.87170::c9581_BT_Eu_ru_202_g1_i1::g.87170::m.87170	10.32	5.71	YES
	Gene.24389::c5080_BT_Eu_ru_202_g1_i1::g.24389::m.24389	8.59	2.32	NO
	Gene.24395::c5080_BT_Eu_ru_202_g1_i2::g.24395::m.24395	8.59	2.32	NO
	Gene.143229::c36510_BT_Eu_ru_202_g1_i1::g.143229::m.143229	9.66	5.99	NO

	Gene.22466::c4795_BT_Eu_ru_202_g1_i1::g.22466::m.22466	10.32	6.14	NO	
	Gene.151519::c45290_BT_Eu_ru_202_g1_i1::g.151519::m.151519	8.05	4.23	NO	
	Gene.16835::c4067_BT_Eu_ru_202_g1_i1::g.16835::m.16835	23.84	16.85	YES	
	Gene.33309::c6191_BT_Eu_ru_202_g1_i1::g.33309::m.33309	5.67	3.29	YES	
	Gene.14379::c3421_BT_Eu_ru_202_g1_i1::g.14379::m.14379	6.52	3.92	NO	
	Gene.24087::c5037_BT_Eu_ru_202_g1_i1::g.24087::m.24087	5.40	3.42	YES	
	Gene.109792::c10403_BT_Eu_ru_202_g1_i1::g.109792::m.109792	4.05	2.47	NO	
	Gene.112741::c10480_BT_Eu_ru_202_g1_i1::g.112741::m.112741	22.64	26.56	NO	
	Gene.12313::c2910_BT_Eu_ru_202_g1_i2::g.12313::m.12313	3.10	1.68	YES	
	Gene.5006::c1201_BT_Eu_ru_202_g1_i1::g.5006::m.5006	2.59	1.53	NO	
	Gene.109798::c10403_BT_Eu_ru_202_g2_i1::g.109798::m.109798	2.88	1.44	NO	
	Gene.142066::c36082_BT_Eu_ru_202_g1_i1::g.142066::m.142066	2.71	1.70	NO	
	Gene.117255::c11176_BT_Eu_ru_202_g1_i1::g.117255::m.117255	2.38	1.53	NO	
	Gene.20868::c4591_BT_Eu_ru_202_g1_i1::g.20868::m.20868	1.90	1.17	NO	
	Gene.4552::c1057_BT_Eu_ru_202_g1_i1::g.4552::m.4552	5.23	3.53	NO	
	Gene.126531::c20465_BT_Eu_ru_202_g1_i1::g.126531::m.126531	1.92	0.99	NO	
	Gene.56303::c8008_BT_Eu_ru_202_g1_i1::g.56303::m.56303	2.45	1.58	NO	
	Gene.120000::c12771_BT_Eu_ru_202_g1_i1::g.120000::m.120000	2.47	1.62	NO	
	Gene.162148::c54544_BT_Eu_ru_202_g1_i1::g.162148::m.162148	1.90	1.41	NO	
	Gene.4553::c1057_BT_Eu_ru_202_g1_i1::g.4553::m.4553	4.09	3.97	NO	
	Gene.128951::c21889_BT_Eu_ru_202_g1_i1::g.128951::m.128951	2.61	1.98	NO	
	Gene.154511::c46721_BT_Eu_ru_202_g1_i1::g.154511::m.154511	1.71	0.93	NO	
	Gene.120001::c12771_BT_Eu_ru_202_g1_i1::g.120001::m.120001	3.49	3.93	NO	
	Gene.126010::c20265_BT_Eu_ru_202_g1_i1::g.126010::m.126010	1.41	0.91	NO	
	Gene.2037::c474_BT_Eu_ru_202_g1_i1::g.2037::m.2037	1.77	0.86	NO	
	Gene.126854::c20581_BT_Eu_ru_202_g1_i1::g.126854::m.126854	1.34	0.71	NO	
	Gene.109803::c10403_BT_Eu_ru_202_g1_i2::g.109803::m.109803	2.68	1.42	NO	
	Gene.117351::c11208_BT_Eu_ru_202_g1_i1::g.117351::m.117351	1.33	1.02	NO	
	Gene.109801::c10403_BT_Eu_ru_202_g2_i2::g.109801::m.109801	2.46	1.32	NO	
	Gene.117424::c11247_BT_Eu_ru_202_g1_i1::g.117424::m.117424	1.73	0.98	NO	
	Gene.127646::c20999_BT_Eu_ru_202_g1_i1::g.127646::m.127646	1.41	0.93	NO	
	Gene.67::c15_BT_Eu_ru_202_g1_i1::g.67::m.67	1.17	0.82	NO	
	Gene.147075::c39117_BT_Eu_ru_202_g1_i1::g.147075::m.147075	1.64	0.99	NO	
	Gene.162654::c54832_BT_Eu_ru_202_g1_i1::g.162654::m.162654	1.49	1.56	NO	
	Gene.4554::c1057_BT_Eu_ru_202_g1_i1::g.4554::m.4554	2.30	0.92	NO	
	Gene.163631::c55458_BT_Eu_ru_202_g1_i1::g.163631::m.163631	1.05	0.54	NO	
	Gene.141178::c34886_BT_Eu_ru_202_g1_i1::g.141178::m.141178	1.26	0.75	NO	
	Gene.5032::c1208_BT_Eu_ru_202_g1_i1::g.5032::m.5032	0.95	1.16	YES	
	Gene.116220::c10772_BT_Eu_ru_202_g1_i1::g.116220::m.116220	0.96	1.19	NO	
	Gene.138231::c31214_BT_Eu_ru_202_g1_i1::g.138231::m.138231	1.05	0.93	NO	
	Gene.136608::c29798_BT_Eu_ru_202_g1_i1::g.136608::m.136608	0.52	0.98	NO	
	Gene.147335::c39397_BT_Eu_ru_202_g1_i1::g.147335::m.147335	0.70	1.59	NO	
	Gene.120580::c13241_BT_Eu_ru_202_g1_i1::g.120580::m.120580	0.58	1.32	NO	
	Gene.122511::c15171_BT_Eu_ru_202_g1_i1::g.122511::m.122511	0.54	0.74	NO	
	Gene.129497::c22349_BT_Eu_ru_202_g1_i1::g.129497::m.129497	0.69	1.78	NO	
	Gene.133810::c28059_BT_Eu_ru_202_g1_i1::g.133810::m.133810	0.73	0.00	NO	
	Gene.135954::c29397_BT_Eu_ru_202_g1_i1::g.135954::m.135954	0.60	0.63	NO	
	Gene.147945::c40018_BT_Eu_ru_202_g1_i1::g.147945::m.147945	0.70	1.86	NO	
	Gene.131115::c24020_BT_Eu_ru_202_g1_i1::g.131115::m.131115	0.71	2.92	NO	
	Gene.132928::c26412_BT_Eu_ru_202_g1_i1::g.132928::m.132928	0.89	2.70	NO	
	Gene.141203::c34937_BT_Eu_ru_202_g1_i1::g.141203::m.141203	0.66	0.00	NO	
	Gene.123892::c16918_BT_Eu_ru_202_g1_i1::g.123892::m.123892	0.70	0.00	NO	
	Gene.131761::c24802_BT_Eu_ru_202_g1_i1::g.131761::m.131761	0.42	1.23	NO	
	Gene.141470::c35432_BT_Eu_ru_202_g1_i1::g.141470::m.141470	0.60	4.66	NO	
	Gene.160182::c53593_BT_Eu_ru_202_g1_i1::g.160182::m.160182	0.74	0.00	NO	
	Gene.145366::c37708_BT_Eu_ru_202_g1_i1::g.145366::m.145366	0.44	0.00	NO	
	Gene.147527::c39608_BT_Eu_ru_202_g1_i1::g.147527::m.147527	0.51	0.00	NO	
	Gene.132156::c25347_BT_Eu_ru_202_g1_i1::g.132156::m.132156	0.35	2.76	NO	
	Gene.166801::c58564_BT_Eu_ru_202_g1_i1::g.166801::m.166801	0.29	5.07	NO	
**Peptidase M14**	Gene.151432::c45264_BT_Eu_ru_202_g1_i1::g.151432::m.151432	801.42	484.53	NO	
	Gene.126185::c20337_BT_Eu_ru_202_g1_i1::g.126185::m.126185	338.25	194.62	NO	
	Gene.125932::c20241_BT_Eu_ru_202_g1_i1::g.125932::m.125932	125.57	75.55	NO	
	Gene.44827::c7204_BT_Eu_ru_202_g2_i2::g.44827::m.44827	29.52	0.39	YES	
	Gene.44817::c7204_BT_Eu_ru_202_g2_i1::g.44817::m.44817	28.24	11.72	YES	
	Gene.44835::c7204_BT_Eu_ru_202_g2_i3::g.44835::m.44835	29.58	1.27	YES	
	Gene.93762::c9845_BT_Eu_ru_202_g2_i1::g.93762::m.93762	101.44	35.94	YES	
	Gene.93768::c9845_BT_Eu_ru_202_g2_i2::g.93768::m.93768	101.44	35.94	YES	
	Gene.93760::c9845_BT_Eu_ru_202_g1_i1::g.93760::m.93760	104.59	64.46	NO	
	Gene.44846::c7204_BT_Eu_ru_202_g2_i5::g.44846::m.44846	41.28	18.83	YES	
	Gene.11984::c2859_BT_Eu_ru_202_g1_i1::g.11984::m.11984	36.84	22.63	NO	
	Gene.116370::c10828_BT_Eu_ru_202_g1_i1::g.116370::m.116370	41.31	29.29	NO	
	Gene.161025::c54021_BT_Eu_ru_202_g1_i1::g.161025::m.161025	12.19	6.80	NO	
	Gene.44841::c7204_BT_Eu_ru_202_g2_i4::g.44841::m.44841	13.33	1.33	YES	
	Gene.40121::c6833_BT_Eu_ru_202_g1_i3::g.40121::m.40121	4.70	1.08	YES	
	Gene.40117::c6833_BT_Eu_ru_202_g1_i2::g.40117::m.40117	4.20	0.88	YES	
	Gene.40112::c6833_BT_Eu_ru_202_g1_i1::g.40112::m.40112	4.49	0.62	YES	
	Gene.162109::c54521_BT_Eu_ru_202_g1_i1::g.162109::m.162109	4.05	2.81	NO	
	Gene.137700::c30704_BT_Eu_ru_202_g1_i1::g.137700::m.137700	0.71	0.00	NO	
	Gene.122691::c15383_BT_Eu_ru_202_g1_i1::g.122691::m.122691	0.83	0.66	NO	
	Gene.154952::c47058_BT_Eu_ru_202_g1_i1::g.154952::m.154952	0.72	0.43	NO	
	Gene.121304::c13918_BT_Eu_ru_202_g1_i1::g.121304::m.121304	0.53	1.15	NO	
	Gene.146822::c38846_BT_Eu_ru_202_g1_i1::g.146822::m.146822	0.67	1.24	NO	
	Gene.158738::c51017_BT_Eu_ru_202_g1_i1::g.158738::m.158738	0.46	0.98	NO	
	Gene.118410::c11747_BT_Eu_ru_202_g1_i1::g.118410::m.118410	0.59	1.46	NO	
**Peptidase S10**	Gene.56023::c7990_BT_Eu_ru_202_g1_i1::g.56023::m.56023	110.34	64.26	YES	
	Gene.126554::c20475_BT_Eu_ru_202_g1_i1::g.126554::m.126554	40.65	24.81	NO	
	Gene.74343::c9062_BT_Eu_ru_202_g1_i1::g.74343::m.74343	6.56	3.09	YES	
**Peptidase S1**	Gene.1164::c246_BT_Eu_ru_202_g1_i1::g.1164::m.1164	2110.08	1305.22	YES	
	Gene.77973::c9226_BT_Eu_ru_202_g1_i1::g.77973::m.77973	1734.55	1128.15	YES	
	Gene.44555::c7186_BT_Eu_ru_202_g1_i1::g.44555::m.44555	1347.79	836.98	YES	
	Gene.27841::c5556_BT_Eu_ru_202_g1_i1::g.27841::m.27841	1132.26	732.20	YES	
	Gene.161299::c54149_BT_Eu_ru_202_g1_i1::g.161299::m.161299	1094.87	704.45	NO	
	Gene.30986::c5960_BT_Eu_ru_202_g1_i1::g.30986::m.30986	898.09	596.47	YES	
	Gene.45971::c7278_BT_Eu_ru_202_g1_i1::g.45971::m.45971	903.05	589.55	YES	
	Gene.71847::c8913_BT_Eu_ru_202_g1_i1::g.71847::m.71847	675.81	448.89	NO	
	Gene.21632::c4688_BT_Eu_ru_202_g1_i1::g.21632::m.21632	710.49	459.02	YES	
	Gene.84017::c9455_BT_Eu_ru_202_g1_i1::g.84017::m.84017	63.95	23.59	YES	excluded
	Gene.70893::c8871_BT_Eu_ru_202_g1_i1::g.70893::m.70893	474.83	381.77	YES	
	Gene.154678::c46846_BT_Eu_ru_202_g1_i1::g.154678::m.154678	415.61	275.85	NO	
	Gene.84040::c9455_BT_Eu_ru_202_g1_i2::g.84040::m.84040	75.32	17.37	YES	excluded
	Gene.36925::c6548_BT_Eu_ru_202_g1_i1::g.36925::m.36925	339.41	226.89	YES	
	Gene.51851::c7708_BT_Eu_ru_202_g1_i1::g.51851::m.51851	213.68	132.44	YES	
	Gene.79666::c9289_BT_Eu_ru_202_g1_i1::g.79666::m.79666	320.02	179.24	YES	
	Gene.68770::c8765_BT_Eu_ru_202_g2_i2::g.68770::m.68770	174.60	62.73	YES	
	Gene.79670::c9289_BT_Eu_ru_202_g1_i2::g.79670::m.79670	239.15	52.56	YES	
	Gene.68759::c8765_BT_Eu_ru_202_g2_i1::g.68759::m.68759	166.91	45.85	YES	
	Gene.19914::c4449_BT_Eu_ru_202_g1_i1::g.19914::m.19914	167.49	105.77	YES	

	Gene.35606::c6400_BT_Eu_ru_202_g1_i1::g.35606::m.35606	203.90	131.52	YES	
	Gene.134496::c28673_BT_Eu_ru_202_g1_i1::g.134496::m.134496	215.02	144.38	NO	
	Gene.88825::c9654_BT_Eu_ru_202_g1_i1::g.88825::m.88825	202.01	135.65	YES	
	Gene.56692::c8029_BT_Eu_ru_202_g1_i1::g.56692::m.56692	184.58	116.94	YES	
	Gene.33906::c6234_BT_Eu_ru_202_g1_i1::g.33906::m.33906	164.13	104.82	NO	
	Gene.8758::c2145_BT_Eu_ru_202_g2_i1::g.8758::m.8758	170.32	110.98	YES	
	Gene.38453::c6697_BT_Eu_ru_202_g1_i1::g.38453::m.38453	169.72	115.57	YES	
	Gene.40524::c6874_BT_Eu_ru_202_g1_i1::g.40524::m.40524	35.10	13.13	NO	excluded
	Gene.41446::c6944_BT_Eu_ru_202_g1_i1::g.41446::m.41446	150.58	100.36	YES	
	Gene.160590::c53866_BT_Eu_ru_202_g1_i1::g.160590::m.160590	131.45	85.14	NO	
	Gene.58755::c8133_BT_Eu_ru_202_g1_i1::g.58755::m.58755	123.13	77.08	NO	
	Gene.84335::c9468_BT_Eu_ru_202_g1_i1::g.84335::m.84335	79.36	24.58	NO	
	Gene.84341::c9468_BT_Eu_ru_202_g1_i2::g.84341::m.84341	79.36	24.58	NO	
	Gene.84348::c9468_BT_Eu_ru_202_g1_i3::g.84348::m.84348	79.37	0.00	NO	
	Gene.84355::c9468_BT_Eu_ru_202_g1_i4::g.84355::m.84355	79.37	0.00	NO	
	Gene.81799::c9367_BT_Eu_ru_202_g1_i1::g.81799::m.81799	111.42	70.21	YES	
	Gene.15817::c3781_BT_Eu_ru_202_g1_i1::g.15817::m.15817	17.44	9.53	NO	excluded
	Gene.125984::c20252_BT_Eu_ru_202_g1_i1::g.125984::m.125984	108.34	66.45	NO	
	Gene.60623::c8272_BT_Eu_ru_202_g1_i2::g.60623::m.60623	79.20	42.70	YES	
	Gene.21811::c4712_BT_Eu_ru_202_g1_i1::g.21811::m.21811	103.44	66.80	YES	
	Gene.46212::c7294_BT_Eu_ru_202_g1_i1::g.46212::m.46212	69.12	45.62	YES	
	Gene.8757::c2145_BT_Eu_ru_202_g2_i1::g.8757::m.8757	94.91	62.92	YES	
	Gene.36583::c6505_BT_Eu_ru_202_g1_i1::g.36583::m.36583	94.66	61.48	YES	
	Gene.67547::c8693_BT_Eu_ru_202_g1_i1::g.67547::m.67547	69.35	46.74	YES	
	Gene.60619::c8272_BT_Eu_ru_202_g1_i1::g.60619::m.60619	66.33	36.32	YES	
	Gene.48972::c7511_BT_Eu_ru_202_g1_i1::g.48972::m.48972	86.96	60.23	NO	
	Gene.83624::c9441_BT_Eu_ru_202_g3_i1::g.83624::m.83624	45.34	29.31	YES	
	Gene.40536::c6874_BT_Eu_ru_202_g1_i2::g.40536::m.40536	57.63	24.31	NO	
	Gene.34860::c6333_BT_Eu_ru_202_g1_i1::g.34860::m.34860	73.31	46.93	YES	
	Gene.56698::c8029_BT_Eu_ru_202_g2_i1::g.56698::m.56698	77.14	48.89	YES	
	Gene.72687::c8962_BT_Eu_ru_202_g1_i1::g.72687::m.72687	75.85	50.07	YES	
	Gene.139587::c32623_BT_Eu_ru_202_g1_i1::g.139587::m.139587	83.68	51.86	NO	
	Gene.117739::c11393_BT_Eu_ru_202_g1_i1::g.117739::m.117739	49.04	32.68	NO	
	Gene.72179::c8934_BT_Eu_ru_202_g2_i1::g.72179::m.72179	37.26	23.05	YES	
	Gene.114375::c10524_BT_Eu_ru_202_g1_i2::g.114375::m.114375	55.88	8.92	NO	
	Gene.114400::c10524_BT_Eu_ru_202_g1_i6::g.114400::m.114400	55.16	18.80	NO	
	Gene.2708::c658_BT_Eu_ru_202_g1_i2::g.2708::m.2708	30.01	9.28	YES	
	Gene.126406::c20430_BT_Eu_ru_202_g1_i1::g.126406::m.126406	61.29	40.37	NO	
	Gene.91044::c9739_BT_Eu_ru_202_g1_i1::g.91044::m.91044	72.72	52.00	YES	
	Gene.113485::c10499_BT_Eu_ru_202_g1_i1::g.113485::m.113485	7.64	3.90	NO	excluded
	Gene.34163::c6268_BT_Eu_ru_202_g1_i1::g.34163::m.34163	60.20	41.02	YES	
	Gene.15892::c3796_BT_Eu_ru_202_g1_i1::g.15892::m.15892	47.88	27.86	YES	
	Gene.114382::c10524_BT_Eu_ru_202_g1_i3::g.114382::m.114382	58.79	19.95	NO	
	Gene.16139::c3881_BT_Eu_ru_202_g1_i1::g.16139::m.16139	57.53	39.87	YES	
	Gene.145570::c37840_BT_Eu_ru_202_g1_i1::g.145570::m.145570	48.69	34.12	NO	
	Gene.114393::c10524_BT_Eu_ru_202_g1_i5::g.114393::m.114393	56.02	1.43	NO	
	Gene.48858::c7498_BT_Eu_ru_202_g1_i1::g.48858::m.48858	66.26	50.19	NO	
	Gene.114406::c10524_BT_Eu_ru_202_g1_i7::g.114406::m.114406	57.45	2.73	NO	
	Gene.117552::c11323_BT_Eu_ru_202_g1_i1::g.117552::m.117552	44.58	29.35	NO	
	Gene.14311::c3408_BT_Eu_ru_202_g1_i1::g.14311::m.14311	39.09	27.42	YES	
	Gene.60095::c8241_BT_Eu_ru_202_g1_i1::g.60095::m.60095	41.04	26.79	YES	
	Gene.69264::c8790_BT_Eu_ru_202_g1_i1::g.69264::m.69264	29.14	19.16	YES	
	Gene.37431::c6603_BT_Eu_ru_202_g1_i1::g.37431::m.37431	40.90	26.57	YES	
	Gene.116614::c10907_BT_Eu_ru_202_g1_i1::g.116614::m.116614	36.99	24.89	NO	
	Gene.19630::c4411_BT_Eu_ru_202_g1_i1::g.19630::m.19630	36.23	25.65	YES	
	Gene.152447::c45613_BT_Eu_ru_202_g1_i1::g.152447::m.152447	34.05	21.55	NO	
	Gene.58708::c8128_BT_Eu_ru_202_g1_i1::g.58708::m.58708	23.55	15.07	YES	
	Gene.14194::c3384_BT_Eu_ru_202_g1_i2::g.14194::m.14194	15.67	9.10	YES	
	Gene.115368::c10547_BT_Eu_ru_202_g1_i4::g.115368::m.115368	31.02	20.41	NO	
	Gene.161313::c54150_BT_Eu_ru_202_g1_i1::g.161313::m.161313	32.10	20.93	NO	
	Gene.91010::c9738_BT_Eu_ru_202_g1_i1::g.91010::m.91010	19.41	7.20	YES	
	Gene.115376::c10547_BT_Eu_ru_202_g1_i7::g.115376::m.115376	29.04	15.24	NO	
	Gene.42609::c7057_BT_Eu_ru_202_g1_i1::g.42609::m.42609	32.71	20.04	YES	
	Gene.142808::c36336_BT_Eu_ru_202_g1_i1::g.142808::m.142808	30.00	19.02	NO	
	Gene.145963::c38114_BT_Eu_ru_202_g1_i1::g.145963::m.145963	21.65	15.00	NO	
	Gene.114412::c10524_BT_Eu_ru_202_g1_i8::g.114412::m.114412	53.58	25.76	NO	
	Gene.146050::c38183_BT_Eu_ru_202_g1_i1::g.146050::m.146050	25.95	16.73	NO	
	Gene.151736::c45342_BT_Eu_ru_202_g1_i1::g.151736::m.151736	23.18	15.26	NO	
	Gene.130610::c23430_BT_Eu_ru_202_g1_i1::g.130610::m.130610	24.71	15.99	NO	
	Gene.26986::c5444_BT_Eu_ru_202_g1_i1::g.26986::m.26986	24.47	14.91	YES	
	Gene.154358::c46637_BT_Eu_ru_202_g1_i1::g.154358::m.154358	18.51	12.08	NO	
	Gene.157749::c49727_BT_Eu_ru_202_g1_i1::g.157749::m.157749	23.47	14.26	NO	
	Gene.114407::c10524_BT_Eu_ru_202_g1_i7::g.114407::m.114407	62.05	81.89	NO	
	Gene.40591::c6882_BT_Eu_ru_202_g1_i2::g.40591::m.40591	21.08	13.09	YES	
	Gene.36495::c6491_BT_Eu_ru_202_g1_i1::g.36495::m.36495	27.07	20.20	NO	
	Gene.115363::c10547_BT_Eu_ru_202_g1_i2::g.115363::m.115363	22.55	13.37	NO	
	Gene.23002::c4858_BT_Eu_ru_202_g1_i1::g.23002::m.23002	19.41	13.44	NO	
	Gene.91030::c9738_BT_Eu_ru_202_g1_i6::g.91030::m.91030	17.47	5.32	YES	
	Gene.144667::c37260_BT_Eu_ru_202_g1_i1::g.144667::m.144667	19.71	13.48	NO	
	Gene.32351::c6088_BT_Eu_ru_202_g1_i1::g.32351::m.32351	19.22	10.93	YES	
	Gene.11268::c2718_BT_Eu_ru_202_g1_i1::g.11268::m.11268	8.44	4.99	YES	
	Gene.50116::c7580_BT_Eu_ru_202_g1_i2::g.50116::m.50116	19.08	12.57	YES	
	Gene.16455::c3970_BT_Eu_ru_202_g1_i2::g.16455::m.16455	17.10	10.29	YES	
	Gene.23608::c4977_BT_Eu_ru_202_g1_i1::g.23608::m.23608	5.45	3.33	NO	excluded
	Gene.37271::c6581_BT_Eu_ru_202_g1_i1::g.37271::m.37271	18.97	12.22	YES	
	Gene.91013::c9738_BT_Eu_ru_202_g1_i2::g.91013::m.91013	18.19	0.24	YES	
	Gene.91019::c9738_BT_Eu_ru_202_g1_i3::g.91019::m.91019	18.19	0.24	YES	
	Gene.97941::c10009_BT_Eu_ru_202_g2_i2::g.97941::m.97941	6.76	4.28	YES	
	Gene.114414::c10524_BT_Eu_ru_202_g1_i8::g.114414::m.114414	45.42	0.00	NO	
	Gene.7185::c1676_BT_Eu_ru_202_g1_i1::g.7185::m.7185	17.61	12.11	YES	
	Gene.84461::c9476_BT_Eu_ru_202_g1_i1::g.84461::m.84461	17.87	11.36	YES	
	Gene.58803::c8136_BT_Eu_ru_202_g1_i1::g.58803::m.58803	16.79	11.42	NO	
	Gene.83642::c9441_BT_Eu_ru_202_g2_i3::g.83642::m.83642	9.92	6.47	YES	
	Gene.40587::c6882_BT_Eu_ru_202_g1_i1::g.40587::m.40587	22.01	1.16	YES	
	Gene.114388::c10524_BT_Eu_ru_202_g1_i4::g.114388::m.114388	28.79	14.38	NO	
	Gene.1080::c226_BT_Eu_ru_202_g1_i1::g.1080::m.1080	15.12	11.11	YES	
	Gene.83612::c9441_BT_Eu_ru_202_g1_i1::g.83612::m.83612	9.21	2.26	YES	
	Gene.83648::c9441_BT_Eu_ru_202_g1_i3::g.83648::m.83648	9.21	2.26	YES	
	Gene.5808::c1428_BT_Eu_ru_202_g1_i1::g.5808::m.5808	58.50	95.63	YES	
	Gene.126058::c20280_BT_Eu_ru_202_g1_i1::g.126058::m.126058	14.28	9.75	NO	
	Gene.56442::c8017_BT_Eu_ru_202_g1_i1::g.56442::m.56442	12.09	8.75	YES	
	Gene.113750::c10506_BT_Eu_ru_202_g1_i2::g.113750::m.113750	14.48	6.09	NO	
	Gene.113740::c10506_BT_Eu_ru_202_g1_i1::g.113740::m.113740	13.95	4.16	NO	
	Gene.69269::c8790_BT_Eu_ru_202_g1_i2::g.69269::m.69269	30.37	12.01	NO	
	Gene.27821::c5552_BT_Eu_ru_202_g1_i1::g.27821::m.27821	46.26	62.52	YES	
	Gene.129390::c22264_BT_Eu_ru_202_g1_i1::g.129390::m.129390	27.03	31.46	NO	
	Gene.114394::c10524_BT_Eu_ru_202_g1_i5::g.114394::m.114394	35.23	14.84	NO	
	Gene.5230::c1260_BT_Eu_ru_202_g1_i1::g.5230::m.5230	11.82	7.33	YES	
	Gene.91039::c9738_BT_Eu_ru_202_g1_i7::g.91039::m.91039	16.96	5.01	NO	
	Gene.93875::c9850_BT_Eu_ru_202_g1_i1::g.93875::m.93875	10.50	7.25	NO	
	Gene.115360::c10547_BT_Eu_ru_202_g1_i1::g.115360::m.115360	22.84	9.42	NO	
	Gene.9118::c2199_BT_Eu_ru_202_g1_i1::g.9118::m.9118	4.61	1.62	NO	
	Gene.83631::c9441_BT_Eu_ru_202_g1_i2::g.83631::m.83631	8.93	2.79	NO	

	Gene.114373::c10524_BT_Eu_ru_202_g1_i1::g.114373::m.114373	36.97	13.46	NO	
	Gene.9127::c2199_BT_Eu_ru_202_g1_i2::g.9127::m.9127	4.54	0.95	NO	
	Gene.67552::c8693_BT_Eu_ru_202_g2_i2::g.67552::m.67552	11.45	8.52	NO	
	Gene.6273::c1513_BT_Eu_ru_202_g1_i2::g.6273::m.6273	7.38	4.96	YES	
	Gene.115380::c10547_BT_Eu_ru_202_g1_i8::g.115380::m.115380	22.03	11.29	NO	
	Gene.21751::c4705_BT_Eu_ru_202_g1_i2::g.21751::m.21751	8.68	5.97	YES	
	Gene.134225::c28571_BT_Eu_ru_202_g1_i1::g.134225::m.134225	8.11	5.82	NO	
	Gene.146473::c38519_BT_Eu_ru_202_g1_i1::g.146473::m.146473	44.08	67.34	NO	
	Gene.19155::c4343_BT_Eu_ru_202_g1_i1::g.19155::m.19155	8.95	6.47	NO	
	Gene.16452::c3970_BT_Eu_ru_202_g1_i1::g.16452::m.16452	15.65	1.50	NO	
	Gene.69268::c8790_BT_Eu_ru_202_g1_i2::g.69268::m.69268	17.44	0.79	NO	
	Gene.50112::c7580_BT_Eu_ru_202_g1_i1::g.50112::m.50112	18.14	0.00	NO	
	Gene.91014::c9738_BT_Eu_ru_202_g1_i2::g.91014::m.91014	20.21	3.06	NO	
	Gene.91028::c9738_BT_Eu_ru_202_g1_i5::g.91028::m.91028	20.21	3.06	NO	
	Gene.106529::c10296_BT_Eu_ru_202_g1_i2::g.106529::m.106529	14.91	6.74	NO	
	Gene.106534::c10296_BT_Eu_ru_202_g1_i3::g.106534::m.106534	14.91	6.74	NO	
	Gene.118873::c12019_BT_Eu_ru_202_g1_i1::g.118873::m.118873	8.23	5.11	NO	
	Gene.2470::c593_BT_Eu_ru_202_g1_i1::g.2470::m.2470	22.10	17.71	NO	
	Gene.30241::c5889_BT_Eu_ru_202_g1_i1::g.30241::m.30241	7.85	5.04	YES	
	Gene.34325::c6286_BT_Eu_ru_202_g1_i1::g.34325::m.34325	7.67	4.92	YES	
	Gene.106530::c10296_BT_Eu_ru_202_g1_i2::g.106530::m.106530	14.37	13.08	NO	
	Gene.16385::c3949_BT_Eu_ru_202_g1_i1::g.16385::m.16385	7.34	5.23	YES	
	Gene.51668::c7696_BT_Eu_ru_202_g1_i1::g.51668::m.51668	7.37	4.43	YES	
	Gene.16453::c3970_BT_Eu_ru_202_g1_i1::g.16453::m.16453	13.17	1.27	YES	
	Gene.34333::c6286_BT_Eu_ru_202_g1_i3::g.34333::m.34333	4.31	1.40	YES	
	Gene.115365::c10547_BT_Eu_ru_202_g1_i3::g.115365::m.115365	13.59	7.52	NO	
	Gene.145897::c38070_BT_Eu_ru_202_g1_i1::g.145897::m.145897	3.63	2.26	NO	
	Gene.36546::c6500_BT_Eu_ru_202_g1_i1::g.36546::m.36546	6.47	3.88	NO	
	Gene.91026::c9738_BT_Eu_ru_202_g1_i4::g.91026::m.91026	17.08	1.82	NO	
	Gene.34329::c6286_BT_Eu_ru_202_g1_i2::g.34329::m.34329	9.23	6.98	NO	
	Gene.15486::c3681_BT_Eu_ru_202_g1_i1::g.15486::m.15486	5.75	5.27	NO	
	Gene.165360::c56952_BT_Eu_ru_202_g1_i1::g.165360::m.165360	4.29	2.77	NO	
	Gene.126160::c20326_BT_Eu_ru_202_g1_i1::g.126160::m.126160	3.24	2.08	NO	
	Gene.32881::c6144_BT_Eu_ru_202_g1_i1::g.32881::m.32881	4.49	3.47	YES	
	Gene.4253::c1005_BT_Eu_ru_202_g1_i1::g.4253::m.4253	3.05	2.24	YES	
	Gene.118261::c11656_BT_Eu_ru_202_g1_i1::g.118261::m.118261	4.04	2.53	NO	
	Gene.114887::c10538_BT_Eu_ru_202_g1_i3::g.114887::m.114887	4.47	6.28	NO	
	Gene.20503::c4538_BT_Eu_ru_202_g1_i1::g.20503::m.20503	2.51	1.47	YES	
	Gene.23952::c5018_BT_Eu_ru_202_g1_i2::g.23952::m.23952	2.58	1.90	YES	
	Gene.13302::c3145_BT_Eu_ru_202_g1_i2::g.13302::m.13302	4.33	3.91	NO	
	Gene.119498::c12415_BT_Eu_ru_202_g1_i1::g.119498::m.119498	3.24	2.01	NO	
	Gene.6560::c1575_BT_Eu_ru_202_g1_i1::g.6560::m.6560	3.49	1.95	YES	
	Gene.21190::c4641_BT_Eu_ru_202_g1_i1::g.21190::m.21190	2.90	1.04	NO	
	Gene.21748::c4705_BT_Eu_ru_202_g1_i1::g.21748::m.21748	6.54	0.00	YES	
	Gene.21749::c4705_BT_Eu_ru_202_g1_i1::g.21749::m.21749	6.35	0.00	NO	
	Gene.114892::c10538_BT_Eu_ru_202_g1_i4::g.114892::m.114892	3.33	3.77	NO	
	Gene.21193::c4641_BT_Eu_ru_202_g1_i2::g.21193::m.21193	3.11	1.44	NO	
	Gene.23946::c5018_BT_Eu_ru_202_g1_i1::g.23946::m.23946	2.91	0.00	NO	
	Gene.13300::c3145_BT_Eu_ru_202_g1_i1::g.13300::m.13300	3.90	0.91	NO	
	Gene.91042::c9738_BT_Eu_ru_202_g1_i8::g.91042::m.91042	8.83	8.31	NO	
	Gene.119066::c12112_BT_Eu_ru_202_g1_i1::g.119066::m.119066	1.53	0.94	NO	
	Gene.135639::c29207_BT_Eu_ru_202_g1_i1::g.135639::m.135639	1.63	0.90	NO	
	Gene.33334::c6194_BT_Eu_ru_202_g1_i1::g.33334::m.33334	5.99	11.30	NO	
	Gene.114885::c10538_BT_Eu_ru_202_g1_i2::g.114885::m.114885	2.50	0.88	NO	
	Gene.143224::c36509_BT_Eu_ru_202_g1_i1::g.143224::m.143224	1.87	1.54	NO	
	Gene.2104::c496_BT_Eu_ru_202_g1_i1::g.2104::m.2104	2.08	1.79	NO	
	Gene.137866::c30863_BT_Eu_ru_202_g1_i1::g.137866::m.137866	1.86	0.90	NO	
	Gene.145921::c38086_BT_Eu_ru_202_g1_i1::g.145921::m.145921	1.47	1.31	NO	
	Gene.151744::c45345_BT_Eu_ru_202_g1_i1::g.151744::m.151744	1.22	0.65	NO	
	Gene.67551::c8693_BT_Eu_ru_202_g2_i1::g.67551::m.67551	7.97	7.27	NO	
	Gene.7216::c1687_BT_Eu_ru_202_g1_i1::g.7216::m.7216	1.73	1.18	NO	
	Gene.9763::c2291_BT_Eu_ru_202_g1_i1::g.9763::m.9763	1.28	1.08	YES	
	Gene.2106::c496_BT_Eu_ru_202_g1_i2::g.2106::m.2106	1.99	0.00	NO	
	Gene.34724::c6320_BT_Eu_ru_202_g1_i2::g.34724::m.34724	2.31	1.07	NO	
	Gene.50864::c7632_BT_Eu_ru_202_g1_i2::g.50864::m.50864	2.36	1.87	NO	
	Gene.115372::c10547_BT_Eu_ru_202_g1_i5::g.115372::m.115372	4.71	10.23	NO	
	Gene.144101::c36940_BT_Eu_ru_202_g1_i1::g.144101::m.144101	1.20	0.68	NO	
	Gene.148111::c40197_BT_Eu_ru_202_g1_i1::g.148111::m.148111	1.22	0.61	NO	
	Gene.163607::c55446_BT_Eu_ru_202_g1_i1::g.163607::m.163607	1.11	0.77	NO	
	Gene.129899::c22733_BT_Eu_ru_202_g1_i1::g.129899::m.129899	2.31	3.46	NO	
	Gene.131697::c24719_BT_Eu_ru_202_g1_i1::g.131697::m.131697	1.18	0.51	NO	
	Gene.34723::c6320_BT_Eu_ru_202_g1_i1::g.34723::m.34723	2.13	0.39	YES	
	Gene.155855::c47767_BT_Eu_ru_202_g1_i1::g.155855::m.155855	1.16	0.46	NO	
	Gene.120227::c12932_BT_Eu_ru_202_g1_i1::g.120227::m.120227	0.85	0.71	NO	
	Gene.15661::c3733_BT_Eu_ru_202_g1_i2::g.15661::m.15661	1.24	0.00	NO	
	Gene.133972::c28482_BT_Eu_ru_202_g1_i1::g.133972::m.133972	1.28	1.21	NO	
	Gene.164754::c56367_BT_Eu_ru_202_g1_i1::g.164754::m.164754	1.14	0.71	NO	
	Gene.50865::c7632_BT_Eu_ru_202_g1_i2::g.50865::m.50865	1.66	1.15	NO	
	Gene.153258::c46022_BT_Eu_ru_202_g1_i1::g.153258::m.153258	0.77	0.54	NO	
	Gene.157053::c48948_BT_Eu_ru_202_g1_i1::g.157053::m.157053	0.87	0.45	NO	
	Gene.114898::c10538_BT_Eu_ru_202_g1_i5::g.114898::m.114898	1.61	0.00	NO	
	Gene.42287::c7034_BT_Eu_ru_202_g1_i1::g.42287::m.42287	2.27	1.71	NO	
	Gene.3189::c803_BT_Eu_ru_202_g1_i1::g.3189::m.3189	1.70	1.52	NO	
	Gene.150125::c42822_BT_Eu_ru_202_g1_i1::g.150125::m.150125	3.22	8.40	NO	
	Gene.162612::c54807_BT_Eu_ru_202_g1_i1::g.162612::m.162612	1.42	1.44	NO	
	Gene.114884::c10538_BT_Eu_ru_202_g1_i1::g.114884::m.114884	2.07	0.00	NO	
	Gene.15655::c3733_BT_Eu_ru_202_g1_i1::g.15655::m.15655	1.33	2.53	NO	
	Gene.157208::c49112_BT_Eu_ru_202_g1_i1::g.157208::m.157208	1.00	1.40	NO	
	Gene.12987::c3063_BT_Eu_ru_202_g1_i1::g.12987::m.12987	1.16	2.27	YES	
	Gene.130495::c23314_BT_Eu_ru_202_g1_i1::g.130495::m.130495	0.77	0.79	NO	
	Gene.144256::c37025_BT_Eu_ru_202_g1_i1::g.144256::m.144256	0.90	1.32	NO	
	Gene.146820::c38845_BT_Eu_ru_202_g1_i1::g.146820::m.146820	0.94	0.57	NO	
	Gene.122030::c14632_BT_Eu_ru_202_g1_i1::g.122030::m.122030	0.76	1.30	NO	
	Gene.128845::c21792_BT_Eu_ru_202_g1_i1::g.128845::m.128845	0.84	0.52	NO	
	Gene.138480::c31421_BT_Eu_ru_202_g1_i1::g.138480::m.138480	0.92	0.31	NO	
	Gene.151797::c45360_BT_Eu_ru_202_g1_i1::g.151797::m.151797	0.90	1.69	NO	
	Gene.166115::c57775_BT_Eu_ru_202_g1_i1::g.166115::m.166115	0.72	0.89	NO	
	Gene.4962::c1184_BT_Eu_ru_202_g1_i1::g.4962::m.4962	1.00	1.47	NO	
	Gene.137196::c30282_BT_Eu_ru_202_g1_i1::g.137196::m.137196	1.02	0.52	NO	
	Gene.138650::c31577_BT_Eu_ru_202_g1_i1::g.138650::m.138650	0.78	0.90	NO	
	Gene.140207::c33407_BT_Eu_ru_202_g1_i1::g.140207::m.140207	0.75	0.28	NO	
	Gene.162808::c54916_BT_Eu_ru_202_g1_i1::g.162808::m.162808	1.10	2.45	NO	
	Gene.83621::c9441_BT_Eu_ru_202_g2_i1::g.83621::m.83621	1.87	0.00	NO	
	Gene.118377::c11730_BT_Eu_ru_202_g1_i1::g.118377::m.118377	0.92	2.46	NO	
	Gene.139319::c32318_BT_Eu_ru_202_g1_i1::g.139319::m.139319	0.61	0.44	NO	
	Gene.133948::c28469_BT_Eu_ru_202_g1_i1::g.133948::m.133948	0.61	1.03	NO	
	Gene.129561::c22398_BT_Eu_ru_202_g1_i1::g.129561::m.129561	0.75	0.93	NO	
	Gene.131295::c24228_BT_Eu_ru_202_g1_i1::g.131295::m.131295	0.82	1.15	NO	
	Gene.143226::c36509_BT_Eu_ru_202_g1_i1::g.143226::m.143226	0.83	0.00	NO	
	Gene.149698::c42192_BT_Eu_ru_202_g1_i1::g.149698::m.149698	0.59	0.86	NO	
	Gene.120077::c12838_BT_Eu_ru_202_g1_i1::g.120077::m.120077	0.57	0.00	NO	
	Gene.132399::c25648_BT_Eu_ru_202_g1_i1::g.132399::m.132399	0.79	0.68	NO	
	Gene.115722::c10615_BT_Eu_ru_202_g1_i1::g.115722::m.115722	0.76	2.47	NO	

	Gene.131022::c23904_BT_Eu_ru_202_g1_i1::g.131022::m.131022	0.93	2.76	NO	
	Gene.131989::c25114_BT_Eu_ru_202_g1_i1::g.131989::m.131989	0.66	1.72	NO	
	Gene.134686::c28757_BT_Eu_ru_202_g1_i1::g.134686::m.134686	0.69	1.27	NO	
	Gene.139806::c32873_BT_Eu_ru_202_g1_i1::g.139806::m.139806	0.67	1.18	NO	
	Gene.159454::c52138_BT_Eu_ru_202_g1_i1::g.159454::m.159454	0.67	1.18	NO	
	Gene.163715::c55500_BT_Eu_ru_202_g1_i1::g.163715::m.163715	0.80	0.00	NO	
	Gene.166924::c58703_BT_Eu_ru_202_g1_i1::g.166924::m.166924	0.47	0.58	NO	
	Gene.42288::c7034_BT_Eu_ru_202_g1_i2::g.42288::m.42288	1.05	3.29	NO	
	Gene.121926::c14518_BT_Eu_ru_202_g1_i1::g.121926::m.121926	0.56	2.16	NO	
	Gene.132312::c25532_BT_Eu_ru_202_g1_i1::g.132312::m.132312	0.81	4.14	NO	
	Gene.132591::c25923_BT_Eu_ru_202_g1_i1::g.132591::m.132591	0.49	0.41	NO	
	Gene.155736::c47665_BT_Eu_ru_202_g1_i1::g.155736::m.155736	0.57	1.72	NO	
	Gene.130327::c23136_BT_Eu_ru_202_g1_i1::g.130327::m.130327	0.62	0.00	NO	
	Gene.134195::c28561_BT_Eu_ru_202_g1_i1::g.134195::m.134195	0.51	0.61	NO	
	Gene.163397::c55273_BT_Eu_ru_202_g1_i1::g.163397::m.163397	0.43	0.86	NO	
	Gene.165245::c56843_BT_Eu_ru_202_g1_i1::g.165245::m.165245	0.44	0.00	NO	
	Gene.124197::c17388_BT_Eu_ru_202_g1_i1::g.124197::m.124197	0.59	1.46	NO	
	Gene.140251::c33458_BT_Eu_ru_202_g1_i1::g.140251::m.140251	0.67	0.00	NO	
	Gene.153751::c46275_BT_Eu_ru_202_g1_i1::g.153751::m.153751	0.74	2.70	NO	
	Gene.159059::c51469_BT_Eu_ru_202_g1_i1::g.159059::m.159059	0.58	1.38	NO	
	Gene.167517::c59466_BT_Eu_ru_202_g1_i1::g.167517::m.167517	0.49	0.89	NO	
	Gene.121290::c13899_BT_Eu_ru_202_g1_i1::g.121290::m.121290	0.41	2.04	NO	
	Gene.123780::c16730_BT_Eu_ru_202_g1_i1::g.123780::m.123780	0.39	1.78	NO	
	Gene.125447::c19516_BT_Eu_ru_202_g1_i1::g.125447::m.125447	0.58	10.14	NO	
	Gene.129207::c22104_BT_Eu_ru_202_g1_i1::g.129207::m.129207	0.51	1.87	NO	
	Gene.139468::c32476_BT_Eu_ru_202_g1_i1::g.139468::m.139468	0.59	0.00	NO	
	Gene.140373::c33640_BT_Eu_ru_202_g1_i1::g.140373::m.140373	0.40	0.93	NO	
	Gene.140471::c33776_BT_Eu_ru_202_g1_i1::g.140471::m.140471	0.46	1.38	NO	
	Gene.143665::c36719_BT_Eu_ru_202_g1_i1::g.143665::m.143665	0.44	1.18	NO	
	Gene.145757::c37970_BT_Eu_ru_202_g1_i1::g.145757::m.145757	0.47	1.46	NO	
	Gene.149135::c41430_BT_Eu_ru_202_g1_i1::g.149135::m.149135	0.45	2.61	NO	
	Gene.149221::c41529_BT_Eu_ru_202_g1_i1::g.149221::m.149221	0.42	0.00	NO	
	Gene.158880::c51213_BT_Eu_ru_202_g1_i1::g.158880::m.158880	0.36	0.00	NO	
	Gene.167050::c58867_BT_Eu_ru_202_g1_i1::g.167050::m.167050	0.56	0.00	NO	
	Gene.124720::c18226_BT_Eu_ru_202_g1_i1::g.124720::m.124720	0.37	0.00	NO	
	Gene.130674::c23504_BT_Eu_ru_202_g1_i1::g.130674::m.130674	0.35	1.46	NO	
	Gene.137405::c30454_BT_Eu_ru_202_g1_i1::g.137405::m.137405	0.38	3.50	NO	
	Gene.141672::c35879_BT_Eu_ru_202_g1_i1::g.141672::m.141672	0.36	0.00	NO	
	Gene.148114::c40198_BT_Eu_ru_202_g1_i1::g.148114::m.148114	0.38	0.00	NO	
	Gene.157903::c49925_BT_Eu_ru_202_g1_i1::g.157903::m.157903	0.40	2.10	NO	
	Gene.166150::c57816_BT_Eu_ru_202_g1_i1::g.166150::m.166150	0.39	1.99	NO	
	Gene.140669::c34065_BT_Eu_ru_202_g1_i1::g.140669::m.140669	0.28	0.00	NO	
	Gene.4964::c1184_BT_Eu_ru_202_g1_i2::g.4964::m.4964	0.14	0.00	NO	
**Phospholipase A2**	Gene.21162::c4635_BT_Eu_ru_202_g1_i1::g.21162::m.21162	13.46	10.87	NO	
	Gene.118557::c11832_BT_Eu_ru_202_g1_i1::g.118557::m.118557	15.96	11.68	NO	
	Gene.13732::c3278_BT_Eu_ru_202_g1_i1::g.13732::m.13732	13.10	9.37	NO	
	Gene.53896::c7848_BT_Eu_ru_202_g1_i1::g.53896::m.53896	6.02	0.38	YES	
	Gene.36007::c6436_BT_Eu_ru_202_g1_i1::g.36007::m.36007	6.64	6.03	YES	
	Gene.53905::c7848_BT_Eu_ru_202_g1_i2::g.53905::m.53905	4.49	3.67	NO	
	Gene.8870::c2162_BT_Eu_ru_202_g1_i1::g.8870::m.8870	2.04	1.21	YES	
	Gene.148845::c41066_BT_Eu_ru_202_g1_i1::g.148845::m.148845	0.65	0.37	NO	
**Pimplin**	Gene.15431::c3670_BT_Eu_ru_202_g1_i1::g.15431::m.15431	4.70	2.57	NO	excluded
	Gene.147588::c39679_BT_Eu_ru_202_g1_i1::g.147588::m.147588	0.89	0.51	NO	
**Serpin**	Gene.117173::c11138_BT_Eu_ru_202_g1_i1::g.117173::m.117173	132.77	87.84	NO	
	Gene.74626::c9077_BT_Eu_ru_202_g1_i1::g.74626::m.74626	124.97	66.53	YES	
	Gene.53257::c7797_BT_Eu_ru_202_g1_i1::g.53257::m.53257	124.34	83.54	YES	
	Gene.47147::c7365_BT_Eu_ru_202_g1_i3::g.47147::m.47147	65.37	27.21	YES	
	Gene.50941::c7638_BT_Eu_ru_202_g1_i2::g.50941::m.50941	78.74	17.66	NO	
	Gene.47158::c7365_BT_Eu_ru_202_g1_i4::g.47158::m.47158	62.95	4.78	YES	
	Gene.50951::c7638_BT_Eu_ru_202_g1_i5::g.50951::m.50951	87.13	6.52	YES	
	Gene.50949::c7638_BT_Eu_ru_202_g1_i4::g.50949::m.50949	86.50	15.00	YES	
	Gene.50938::c7638_BT_Eu_ru_202_g1_i1::g.50938::m.50938	86.61	10.92	YES	
	Gene.50945::c7638_BT_Eu_ru_202_g1_i3::g.50945::m.50945	86.09	4.65	YES	
	Gene.153730::c46265_BT_Eu_ru_202_g1_i1::g.153730::m.153730	66.70	44.47	NO	
	Gene.47139::c7365_BT_Eu_ru_202_g1_i2::g.47139::m.47139	63.26	12.14	YES	
	Gene.143683::c36728_BT_Eu_ru_202_g1_i1::g.143683::m.143683	51.04	31.39	NO	
	Gene.87745::c9609_BT_Eu_ru_202_g1_i1::g.87745::m.87745	20.23	11.68	YES	
	Gene.9330::c2227_BT_Eu_ru_202_g1_i1::g.9330::m.9330	30.34	20.17	YES	
	Gene.49370::c7535_BT_Eu_ru_202_g1_i1::g.49370::m.49370	34.05	19.67	NO	
	Gene.47133::c7365_BT_Eu_ru_202_g1_i1::g.47133::m.47133	56.91	11.28	NO	
	Gene.49369::c7535_BT_Eu_ru_202_g1_i1::g.49369::m.49369	20.13	6.19	YES	
	Gene.49377::c7535_BT_Eu_ru_202_g1_i2::g.49377::m.49377	20.13	6.19	YES	
	Gene.27785::c5547_BT_Eu_ru_202_g1_i1::g.27785::m.27785	17.86	11.97	NO	
	Gene.4319::c1024_BT_Eu_ru_202_g1_i1::g.4319::m.4319	15.77	9.98	NO	
	Gene.49378::c7535_BT_Eu_ru_202_g1_i2::g.49378::m.49378	26.23	5.54	NO	
	Gene.71064::c8881_BT_Eu_ru_202_g1_i2::g.71064::m.71064	11.58	7.55	NO	
	Gene.6529::c1569_BT_Eu_ru_202_g1_i1::g.6529::m.6529	11.90	9.07	NO	
	Gene.8835::c2154_BT_Eu_ru_202_g1_i2::g.8835::m.8835	11.08	8.00	YES	
	Gene.28797::c5677_BT_Eu_ru_202_g1_i1::g.28797::m.28797	5.11	3.05	YES	
	Gene.8831::c2154_BT_Eu_ru_202_g1_i1::g.8831::m.8831	11.06	0.00	YES	
	Gene.2489::c596_BT_Eu_ru_202_g1_i1::g.2489::m.2489	6.51	4.47	YES	
	Gene.49136::c7520_BT_Eu_ru_202_g1_i1::g.49136::m.49136	5.02	3.01	YES	
	Gene.49371::c7535_BT_Eu_ru_202_g1_i1::g.49371::m.49371	18.83	17.75	NO	
	Gene.17451::c4135_BT_Eu_ru_202_g2_i1::g.17451::m.17451	2.83	1.51	NO	
	Gene.50947::c7638_BT_Eu_ru_202_g1_i3::g.50947::m.50947	8.59	6.90	NO	
	Gene.27790::c5547_BT_Eu_ru_202_g1_i2::g.27790::m.27790	6.51	3.18	NO	
	Gene.2496::c596_BT_Eu_ru_202_g1_i2::g.2496::m.2496	4.45	0.00	NO	
	Gene.158215::c50315_BT_Eu_ru_202_g1_i1::g.158215::m.158215	4.30	8.44	NO	
	Gene.2633::c636_BT_Eu_ru_202_g1_i1::g.2633::m.2633	5.08	20.45	NO	
	Gene.1383::c309_BT_Eu_ru_202_g1_i2::g.1383::m.1383	3.83	9.67	NO	
	Gene.15132::c3598_BT_Eu_ru_202_g1_i1::g.15132::m.15132	3.77	12.25	NO	
	Gene.17448::c4135_BT_Eu_ru_202_g1_i1::g.17448::m.17448	1.70	3.17	NO	
	Gene.16594::c4019_BT_Eu_ru_202_g1_i1::g.16594::m.16594	2.71	6.58	NO	
	Gene.16595::c4019_BT_Eu_ru_202_g1_i2::g.16595::m.16595	2.71	6.58	NO	
	Gene.17459::c4135_BT_Eu_ru_202_g1_i2::g.17459::m.17459	0.97	0.00	NO	
	Gene.10602::c2529_BT_Eu_ru_202_g1_i1::g.10602::m.10602	1.23	8.23	NO	
	Gene.157982::c50027_BT_Eu_ru_202_g1_i1::g.157982::m.157982	0.77	0.00	NO	
	Gene.1381::c309_BT_Eu_ru_202_g1_i1::g.1381::m.1381	0.57	0.00	NO	
	Gene.123282::c16109_BT_Eu_ru_202_g1_i1::g.123282::m.123282	0.41	0.97	NO	
	Gene.123405::c16238_BT_Eu_ru_202_g1_i1::g.123405::m.123405	0.46	0.00	NO	
**Sphingomyelinase**	Gene.10413::c2468_BT_Eu_ru_202_g1_i1::g.10413::m.10413	173.43	98.23	NO	
	Gene.73130::c8983_BT_Eu_ru_202_g1_i1::g.73130::m.73130	25.66	13.87	YES	
	Gene.96649::c9956_BT_Eu_ru_202_g1_i1::g.96649::m.96649	21.53	11.79	YES	
	Gene.14533::c3451_BT_Eu_ru_202_g1_i1::g.14533::m.14533	25.38	16.60	YES	
	Gene.10716::c2561_BT_Eu_ru_202_g1_i1::g.10716::m.10716	3.00	1.65	NO	

**SVWC-domain 1 (Asilidin8)**	Gene.118129::c11590_BT_Eu_ru_202_g1_i1::g.118129::m.118129	4.63	6.47	NO	
	Gene.128250::c21369_BT_Eu_ru_202_g1_i1::g.128250::m.128250	4.57	7.73	NO	
**SVWC-domain 2 (Asilidin9)**	Gene.160399::c53785_BT_Eu_ru_202_g1_i1::g.160399::m.160399	33.40	33.55	NO	
	Gene.152506::c45649_BT_Eu_ru_202_g1_i1::g.152506::m.152506	28.25	33.24	NO	
	Gene.119101::c12130_BT_Eu_ru_202_g1_i1::g.119101::m.119101	26.01	33.58	NO	
**Venom acid phosphatase-like**	Gene.52327::c7742_BT_Eu_ru_202_g1_i1::g.52327::m.52327	21.84	15.09	YES	
	Gene.94951::c9898_BT_Eu_ru_202_g1_i2::g.94951::m.94951	20.40	5.54	NO	
	Gene.94957::c9898_BT_Eu_ru_202_g1_i3::g.94957::m.94957	20.40	5.54	NO	
	Gene.94945::c9898_BT_Eu_ru_202_g1_i1::g.94945::m.94945	18.72	2.20	NO	
	Gene.94944::c9898_BT_Eu_ru_202_g1_i1::g.94944::m.94944	16.11	0.99	NO	
	Gene.91784::c9775_BT_Eu_ru_202_g2_i1::g.91784::m.91784	5.96	3.58	NO	
	Gene.32451::c6099_BT_Eu_ru_202_g1_i1::g.32451::m.32451	1.82	1.30	YES	
