# Supplementary material for: A Dipteran’s Novel Sucker Punch: Evolution of Arthropod Atypical Venom with a Neurotoxic Component in Robber Flies (Asilidae, Diptera)
Source: Toxins (Basel). 2018 Jan 5;10(1):29. doi: 10.3390/toxins10010029 (PMC5793116; doi:10.3390/toxins10010029)
Supplement: Supplementary file 1 [file toxins-10-00029-s001.zip › toxins-254261-supplementary/toxins-254261 supplementary file 3.pdf]

Protein Class	Contig ID	TPM - Segemehl	TPM - Kallisto	Signal Peptide	Protein Hit	Protein Name	false positive
**Aegyptin**	no hit	0	0	X	X		
**Alpha-amylase**	Gene.32383::c6358_g1_i1_VG_Eu_ru_204::g.32383::m.32383	8095.60	4447.21	YES	NO		
**Anophelin**	no hit	0	0	X	X		
**Apyrase**	Gene.94938::c23568_g1_i1_VG_Eu_ru_204::g.94938::m.94938	0.81	0.47	NO	NO		
	Gene.88055::c16877_g1_i1_VG_Eu_ru_204::g.88055::m.88055	0.52	0.27	NO	NO		
	Gene.99726::c27472_g1_i1_VG_Eu_ru_204::g.99726::m.99726	0.39	0.18	NO	NO		
	Gene.90958::c18849_g1_i1_VG_Eu_ru_204::g.90958::m.90958	0.20	0.21	NO	NO		
	Gene.114344::c41461_g1_i1_VG_Eu_ru_204::g.114344::m.114344	0.22	0.98	NO	NO		
	Gene.94413::c23299_g1_i1_VG_Eu_ru_204::g.94413::m.94413	0.21	0.57	NO	NO		
**CAP**	Gene.56292::c8081_g1_i1_VG_Eu_ru_204::g.56292::m.56292	20.08	11.32	NO	NO		
	Gene.25434::c5637_g1_i1_VG_Eu_ru_204::g.25434::m.25434	33.29	21.55	YES	NO		
	Gene.56304::c8081_g1_i2_VG_Eu_ru_204::g.56304::m.56304	17.79	0.28	NO	NO		
	Gene.120487::c47127_g1_i1_VG_Eu_ru_204::g.120487::m.120487	18.52	10.26	NO	NO		
	Gene.30179::c6142_g1_i2_VG_Eu_ru_204::g.30179::m.30179	1.53	1.16	YES	NO		
	Gene.30174::c6142_g1_i1_VG_Eu_ru_204::g.30174::m.30174	0.98	0.00	YES	NO		
	Gene.119506::c46271_g1_i1_VG_Eu_ru_204::g.119506::m.119506	0.35	0.66	NO	NO		
	Gene.114899::c42170_g1_i1_VG_Eu_ru_204::g.114899::m.114899	0.26	0.00	NO	NO		
**Chitinase**	Gene.50699::c7760_g1_i1_VG_Eu_ru_204::g.50699::m.50699	218.71	111.70	YES	NO		
	Gene.33640::c6465_g1_i1_VG_Eu_ru_204::g.33640::m.33640	2.89	0.93	NO	NO		
	Gene.74512::c8891_g1_i1_VG_Eu_ru_204::g.74512::m.74512	9.46	5.20	YES	NO		
	Gene.119146::c45986_g1_i1_VG_Eu_ru_204::g.119146::m.119146	8.94	4.70	NO	NO		
	Gene.58313::c8192_g1_i1_VG_Eu_ru_204::g.58313::m.58313	4.75	2.74	NO	NO		
	Gene.1064::c337_g1_i1_VG_Eu_ru_204::g.1064::m.1064	7.56	4.64	NO	NO		
	Gene.7116::c2115_g1_i1_VG_Eu_ru_204::g.7116::m.7116	0.96	0.64	NO	NO		
	Gene.105109::c32613_g1_i1_VG_Eu_ru_204::g.105109::m.105109	0.83	0.48	NO	NO		
	Gene.118790::c45742_g1_i1_VG_Eu_ru_204::g.118790::m.118790	0.59	0.54	NO	NO		
	Gene.106754::c34301_g1_i1_VG_Eu_ru_204::g.106754::m.106754	1.31	1.11	NO	NO		
	Gene.117565::c45001_g1_i1_VG_Eu_ru_204::g.117565::m.117565	0.45	0.54	NO	NO		
	Gene.116959::c44720_g1_i1_VG_Eu_ru_204::g.116959::m.116959	0.35	0.23	NO	NO		
	Gene.11852::c3557_g1_i1_VG_Eu_ru_204::g.11852::m.11852	0.39	0.21	NO	NO		
	Gene.91913::c19802_g1_i1_VG_Eu_ru_204::g.91913::m.91913	0.52	0.94	YES	NO		
	Gene.93109::c21215_g1_i1_VG_Eu_ru_204::g.93109::m.93109	0.20	0.57	NO	NO		
	Gene.113432::c40431_g1_i1_VG_Eu_ru_204::g.113432::m.113432	0.31	1.19	NO	NO		
	Gene.122395::c49222_g1_i1_VG_Eu_ru_204::g.122395::m.122395	0.23	0.44	NO	NO		
	Gene.117154::c44827_g1_i1_VG_Eu_ru_204::g.117154::m.117154	0.25	1.91	NO	NO		
	Gene.116091::c44044_g1_i1_VG_Eu_ru_204::g.116091::m.116091	0.15	0.00	NO	NO		
**Conoporin-like**	no hit	0	0	X	X		
**Csalpha/beta defensin**	Gene.23259::c5354_g1_i1_VG_Eu_ru_204::g.23259::m.23259	3.45	3.81	NO	NO		
	Gene.918::c290_g1_i1_VG_Eu_ru_204::g.918::m.918	1.11	1.99	NO	NO		
	Gene.101988::c30522_g1_i1_VG_Eu_ru_204::g.101988::m.101988	0.77	1.31	NO	NO		
**Cystatin**	no hit	0	0	X	X		
**dICKS's**	Gene.14819::c4098_g1_i1_VG_Eu_ru_204::g.14819::m.14819	2.45	1.57	YES	NO		
**Disintegrine-like**	no hit	0	0	X	X		
**Echotoxin-like**	no hit	0	0	X	X		
**Gigantoxin-like**	no hit	0	0	X			
**Hyaluronidase**	Gene.43820::c7298_g1_i1_VG_Eu_ru_204::g.43820::m.43820	782.44	60.57	NO	NO		
	Gene.43838::c7298_g1_i3_VG_Eu_ru_204::g.43838::m.43838	782.44	60.57	NO	NO		
	Gene.43831::c7298_g1_i2_VG_Eu_ru_204::g.43831::m.43831	799.69	156.29	YES	NO		
	Gene.43850::c7298_g1_i4_VG_Eu_ru_204::g.43850::m.43850	799.69	156.29	YES	NO		
	Gene.86419::c15398_g1_i1_VG_Eu_ru_204::g.86419::m.86419	0.28	0.70	NO	NO		
**ICK's (Asilidin1)**	Gene.25482::c5644_g1_i1_VG_Eu_ru_204::g.25482::m.25482	7165.69	10985.00	NO	YES	U-Asilidin2-Eru1a	
	Gene.6636::c1937_g1_i1_VG_Eu_ru_204::g.6636::m.6636	736.05	2538.30	YES	NO		
	Gene.82712::c11039_g1_i1_VG_Eu_ru_204::g.82712::m.82712	11.97	22.97	YES	NO		
	Gene.25481::c5644_g1_i1_VG_Eu_ru_204::g.25481::m.25481	8.75	10.67	NO	NO		
	Gene.31380::c6259_g1_i1_VG_Eu_ru_204::g.31380::m.31380	6.37	28.06	YES	NO		
	Gene.14819::c4098_g1_i1_VG_Eu_ru_204::g.14819::m.14819	2.45	1.57	YES	NO		
	Gene.24414::c5508_g1_i1_VG_Eu_ru_204::g.24414::m.24414	1.94	5.86	YES	NO		
	Gene.9809::c2929_g1_i1_VG_Eu_ru_204::g.9809::m.9809	0.84	1.39	YES	NO		
	Gene.2787::c847_g1_i1_VG_Eu_ru_204::g.2787::m.2787	0.40	1.22	YES	NO		
	Gene.112880::c39870_g1_i1_VG_Eu_ru_204::g.112880::m.112880	0.38	1.01	NO	NO		
	Gene.122557::c49431_g1_i1_VG_Eu_ru_204::g.122557::m.122557	0.11	0.51	NO	NO		
**Kazal**	Gene.64171::c8487_g1_i4_VG_Eu_ru_204::g.64171::m.64171	14.30	10.16	YES	NO		
	Gene.64169::c8487_g1_i3_VG_Eu_ru_204::g.64169::m.64169	11.35	0.76	YES	NO		
	Gene.64177::c8487_g1_i5_VG_Eu_ru_204::g.64177::m.64177	11.66	0.59	YES	NO		
	Gene.64166::c8487_g1_i1_VG_Eu_ru_204::g.64166::m.64166	12.26	1.13	YES	NO		
	Gene.121387::c48013_g1_i1_VG_Eu_ru_204::g.121387::m.121387	6.27	7.55	NO	NO		
	Gene.10788::c3201_g1_i1_VG_Eu_ru_204::g.10788::m.10788	4.33	3.00	YES	NO		
	Gene.2778::c838_g1_i1_VG_Eu_ru_204::g.2778::m.2778	5.20	6.08	NO	NO		
	Gene.109049::c37346_g1_i1_VG_Eu_ru_204::g.109049::m.109049	0.77	0.36	NO	NO		
	Gene.2219::c705_g1_i1_VG_Eu_ru_204::g.2219::m.2219	0.53	0.31	NO	NO		
	Gene.5756::c1772_g1_i1_VG_Eu_ru_204::g.5756::m.5756	0.68	0.46	NO	NO		
	Gene.104297::c31944_g1_i1_VG_Eu_ru_204::g.104297::m.104297	0.59	0.25	NO	NO		
	Gene.90025::c18099_g1_i1_VG_Eu_ru_204::g.90025::m.90025	1.04	1.81	YES	NO		
	Gene.87647::c16666_g1_i1_VG_Eu_ru_204::g.87647::m.87647	0.25	0.22	NO	NO		
	Gene.112380::c39391_g1_i1_VG_Eu_ru_204::g.112380::m.112380	0.32	0.51	NO	NO		
	Gene.93420::c21625_g1_i1_VG_Eu_ru_204::g.93420::m.93420	0.18	0.42	NO	NO		
	Gene.105292::c32803_g1_i1_VG_Eu_ru_204::g.105292::m.105292	0.18	1.25	NO	NO		
	Gene.82765::c11072_g1_i1_VG_Eu_ru_204::g.82765::m.82765	0.18	0.00	NO	NO		
**Kazal (vasotab-like)**	Gene.26724::c5793_g1_i1_VG_Eu_ru_204::g.26724::m.26724	25.21	49.94	YES	NO		
	Gene.120711::c47332_g1_i1_VG_Eu_ru_204::g.120711::m.120711	5.29	8.56	NO	NO		
	Gene.2219::c705_g1_i1_VG_Eu_ru_204::g.2219::m.2219	0.53	0.31	NO	NO		
	Gene.89427::c17662_g1_i1_VG_Eu_ru_204::g.89427::m.89427	0.86	0.62	NO	NO		
	Gene.103638::c31458_g1_i1_VG_Eu_ru_204::g.103638::m.103638	1.72	3.68	NO	NO		
**Kunitz**	Gene.109565::c37539_g1_i1_VG_Eu_ru_204::g.109565::m.109565	13.32	9.81	NO	NO		
	Gene.13684::c3920_g1_i1_VG_Eu_ru_204::g.13684::m.13684	10.41	7.24	YES	NO		
	Gene.51541::c7810_g1_i1_VG_Eu_ru_204::g.51541::m.51541	1.25	0.56	NO	NO		
	Gene.51552::c7810_g1_i2_VG_Eu_ru_204::g.51552::m.51552	1.18	0.16	NO	NO		
	Gene.33831::c6485_g1_i2_VG_Eu_ru_204::g.33831::m.33831	2.84	2.13	YES	NO		
	Gene.80039::c9339_g1_i1_VG_Eu_ru_204::g.80039::m.80039	3.88	3.82	YES	NO		
	Gene.33829::c6485_g1_i1_VG_Eu_ru_204::g.33829::m.33829	2.33	0.00	YES	NO		
	Gene.84166::c12408_g1_i1_VG_Eu_ru_204::g.84166::m.84166	0.63	0.42	NO	NO		
	Gene.102284::c30672_g1_i1_VG_Eu_ru_204::g.102284::m.102284	0.88	1.52	NO	NO		
	Gene.85170::c13653_g1_i1_VG_Eu_ru_204::g.85170::m.85170	0.54	0.88	YES	NO		
	Gene.121027::c47646_g1_i1_VG_Eu_ru_204::g.121027::m.121027	0.53	0.26	NO	NO		

	Gene.98032::c25729_g1_i1_VG_Eu_ru_204::g.98032::m.98032	0.46	0.92	YES	NO		
	Gene.84542::c12869_g1_i1_VG_Eu_ru_204::g.84542::m.84542	0.41	0.82	NO	NO		
	Gene.84800::c13191_g1_i1_VG_Eu_ru_204::g.84800::m.84800	0.32	0.00	NO	NO		
	Gene.93932::c22414_g1_i1_VG_Eu_ru_204::g.93932::m.93932	0.22	0.73	NO	NO		
	Gene.115912::c43738_g1_i1_VG_Eu_ru_204::g.115912::m.115912	0.29	0.00	NO	NO		
	Gene.83789::c12013_g1_i1_VG_Eu_ru_204::g.83789::m.83789	0.17	0.00	YES	NO		
**Lectin c-type**	Gene.54596::c7978_g2_i1_VG_Eu_ru_204::g.54596::m.54596	102.91	49.58	YES	NO		
	Gene.54625::c7978_g2_i2_VG_Eu_ru_204::g.54625::m.54625	97.33	6.08	YES	NO		
	Gene.60435::c8303_g1_i1_VG_Eu_ru_204::g.60435::m.60435	8.58	4.45	NO	NO		excluded
	Gene.24029::c5462_g1_i1_VG_Eu_ru_204::g.24029::m.24029	31.57	19.81	YES	NO		
	Gene.67911::c8661_g1_i1_VG_Eu_ru_204::g.67911::m.67911	2.64	0.93	NO	NO		
	Gene.67931::c8661_g1_i4_VG_Eu_ru_204::g.67931::m.67931	5.35	1.25	NO	NO		
	Gene.67923::c8661_g1_i2_VG_Eu_ru_204::g.67923::m.67923	3.94	0.49	NO	NO		
	Gene.117399::c44930_g1_i1_VG_Eu_ru_204::g.117399::m.117399	2.49	2.01	NO	NO		
	Gene.35394::c6607_g1_i1_VG_Eu_ru_204::g.35394::m.35394	2.15	1.73	YES	NO		
	Gene.79379::c9050_g1_i1_VG_Eu_ru_204::g.79379::m.79379	1.46	1.07	YES	NO		
	Gene.96956::c24817_g1_i1_VG_Eu_ru_204::g.96956::m.96956	0.26	0.15	NO	NO		
	Gene.100146::c28002_g1_i1_VG_Eu_ru_204::g.100146::m.100146	0.38	1.01	NO	NO		
**Lipocalin calycins**	Gene.95508::c23892_g1_i1_VG_Eu_ru_204::g.95508::m.95508	14.63	9.38	YES	NO		
	Gene.22429::c5248_g1_i1_VG_Eu_ru_204::g.22429::m.22429	5.88	4.06	YES	NO		
	Gene.93268::c21423_g1_i1_VG_Eu_ru_204::g.93268::m.93268	0.59	0.60	NO	NO		
	Gene.123561::c50908_g1_i1_VG_Eu_ru_204::g.123561::m.123561	0.33	0.29	NO	NO		
	Gene.1618::c565_g1_i1_VG_Eu_ru_204::g.1618::m.1618	0.26	0.13	YES	NO		
**Maxadilan-like**	no hit	0	0	X	X		
**MBF2-domain**	Gene.4165::c1212_g1_i1_VG_Eu_ru_204::g.4165::m.4165	9127.06	8716.38	YES	NO		
	Gene.4074::c1175_g6_i1_VG_Eu_ru_204::g.4074::m.4074	8346.96	10513.20	YES	NO		
	Gene.116489::c44479_g1_i1_VG_Eu_ru_204::g.116489::m.116489	3665.55	3129.84	NO	NO		
	Gene.36327::c6704_g1_i1_VG_Eu_ru_204::g.36327::m.36327	765.45	774.40	YES	NO		
	Gene.23269::c5357_g1_i1_VG_Eu_ru_204::g.23269::m.23269	400.44	233.46	YES	NO		
	Gene.23275::c5357_g1_i2_VG_Eu_ru_204::g.23275::m.23275	400.44	233.46	YES	NO		
	Gene.3256::c983_g1_i1_VG_Eu_ru_204::g.3256::m.3256	326.15	377.80	YES	NO		
	Gene.29774::c6107_g1_i1_VG_Eu_ru_204::g.29774::m.29774	128.84	133.63	YES	NO		
	Gene.17514::c4493_g1_i1_VG_Eu_ru_204::g.17514::m.17514	104.48	85.74	NO	NO		
	Gene.102744::c30916_g1_i1_VG_Eu_ru_204::g.102744::m.102744	21.14	35.10	NO	NO		
**Melittin-like**	no hit	0	0	X	X		
**Mu agatoxin-like**	no hit	0	0	X	X		
**Natterin-like (Asilidin10)**	Gene.8863::c2616_g1_i1_VG_Eu_ru_204::g.8863::m.8863	360.27	232.50	NO	NO		
	Gene.38911::c6949_g1_i1_VG_Eu_ru_204::g.38911::m.38911	207.42	152.24	NO	NO		
	Gene.50334::c7734_g1_i1_VG_Eu_ru_204::g.50334::m.50334	173.35	104.28	NO	NO		
	Gene.40271::c7041_g1_i2_VG_Eu_ru_204::g.40271::m.40271	124.30	33.19	NO	NO		
	Gene.40265::c7041_g1_i1_VG_Eu_ru_204::g.40265::m.40265	162.48	111.80	NO	NO		
**Neurokinin-like**	no hit	0	0	X	X		
**Nitrophorin-like**	no hit	0	0	X	X		
**Novel protein 1 (Asilidin2)**	Gene.94447::c23387_g1_i1_VG_Eu_ru_204::g.94447::m.94447	51106.96	31665.80	YES	YES	U-Asilidin2-Eru1a	
	Gene.46103::c7461_g2_i2_VG_Eu_ru_204::g.46103::m.46103	37141.21	20682.20	YES	YES	U-Asilidin2-Eru3a	
	Gene.46097::c7461_g2_i1_VG_Eu_ru_204::g.46097::m.46097	36258.27	53.86	YES	YES	U-Asilidin2-Eru3b	
	Gene.79411::c9061_g1_i1_VG_Eu_ru_204::g.79411::m.79411	31252.23	17001.90	NO	YES	U-Asilidin2-Eru2a	
	Gene.9035::c2674_g3_i1_VG_Eu_ru_204::g.9035::m.9035	33909.37	19163.10	YES	YES	U-Asilidin2-Eru4a	
	Gene.8959::c2651_g5_i1_VG_Eu_ru_204::g.8959::m.8959	26367.31	15797.80	YES	NO		
	Gene.2314::c726_g1_i1_VG_Eu_ru_204::g.2314::m.2314	14338.19	8246.11	YES	NO		
	Gene.37863::c6854_g2_i1_VG_Eu_ru_204::g.37863::m.37863	4196.43	2561.53	YES	NO		
	Gene.37864::c6854_g2_i1_VG_Eu_ru_204::g.37864::m.37864	81.59	62.05	YES	NO		
	Gene.23725::c5413_g1_i1_VG_Eu_ru_204::g.23725::m.23725	5.55	3.41	YES	NO		
	Gene.115324::c42846_g1_i1_VG_Eu_ru_204::g.115324::m.115324	1.64	1.63	NO	NO		
**Novel protein (Asilidin3)**	Gene.14234::c4012_g1_i1_VG_Eu_ru_204::g.14234::m.14234	50168.40	65478.30	YES	YES	U-Asilidin3-Eru1a	
**Novel protein (Asilidin4)**	Gene.8837::c2610_g3_i1_VG_Eu_ru_204::g.8837::m.8837	23893.20	39114.10	YES	YES	U-Asilidin4-Eru1a	
**Novel protein (Asilidin5)**	Gene.79357::c9042_g2_i1_VG_Eu_ru_204::g.79357::m.79357	9686.63	5586.70	YES	YES	U-Asilidin5-Eru1a	
**Novel protein (Asilidin6)**	Gene.87115::c16440_g1_i1_VG_Eu_ru_204::g.87115::m.87115	2430.98	1642.03	NO	NO		
**Novel protein (Asilidin7)**	no hit	0	0	X	X		
**Peptidase M12**	Gene.62234::c8406_g1_i2_VG_Eu_ru_204::g.62234::m.62234	3.25	0.55	NO	NO		
	Gene.62264::c8406_g1_i5_VG_Eu_ru_204::g.62264::m.62264	3.91	0.00	NO	NO		
	Gene.62287::c8406_g1_i9_VG_Eu_ru_204::g.62287::m.62287	3.88	0.89	NO	NO		
	Gene.33027::c6414_g1_i3_VG_Eu_ru_204::g.33027::m.33027	6.93	1.51	YES	NO		
	Gene.33023::c6414_g1_i1_VG_Eu_ru_204::g.33023::m.33023	7.16	1.39	YES	NO		
	Gene.62283::c8406_g1_i8_VG_Eu_ru_204::g.62283::m.62283	4.07	0.21	NO	NO		
	Gene.62245::c8406_g1_i3_VG_Eu_ru_204::g.62245::m.62245	4.13	0.40	NO	NO		
	Gene.62252::c8406_g1_i4_VG_Eu_ru_204::g.62252::m.62252	4.13	0.40	NO	NO		
	Gene.33025::c6414_g1_i2_VG_Eu_ru_204::g.33025::m.33025	6.38	0.47	NO	NO		
	Gene.4544::c1347_g1_i1_VG_Eu_ru_204::g.4544::m.4544	1.75	0.29	YES	NO		
	Gene.62253::c8406_g1_i4_VG_Eu_ru_204::g.62253::m.62253	2.70	0.21	NO	NO		
	Gene.33031::c6414_g1_i4_VG_Eu_ru_204::g.33031::m.33031	5.69	1.01	NO	NO		
	Gene.8652::c2549_g1_i1_VG_Eu_ru_204::g.8652::m.8652	0.85	0.47	NO	NO		
	Gene.8687::c2562_g1_i1_VG_Eu_ru_204::g.8687::m.8687	0.93	0.61	NO	NO		
	Gene.109216::c37411_g1_i1_VG_Eu_ru_204::g.109216::m.109216	0.89	0.61	NO	NO		
	Gene.90532::c18508_g1_i1_VG_Eu_ru_204::g.90532::m.90532	0.56	0.32	NO	NO		
	Gene.79316::c9026_g1_i1_VG_Eu_ru_204::g.79316::m.79316	0.54	0.37	NO	NO		
	Gene.87237::c16483_g1_i1_VG_Eu_ru_204::g.87237::m.87237	0.36	0.32	NO	NO		
	Gene.98469::c26123_g1_i1_VG_Eu_ru_204::g.98469::m.98469	0.61	0.55	NO	NO		
	Gene.80337::c9481_g1_i1_VG_Eu_ru_204::g.80337::m.80337	0.46	0.43	NO	NO		
	Gene.100718::c28800_g1_i1_VG_Eu_ru_204::g.100718::m.100718	0.36	0.18	NO	NO		
**Peptidase M13**	Gene.29383::c6071_g1_i1_VG_Eu_ru_204::g.29383::m.29383	639.21	370.67	NO	NO		
	Gene.36051::c6668_g1_i1_VG_Eu_ru_204::g.36051::m.36051	9.59	4.86	YES	NO		
	Gene.49552::c7682_g1_i1_VG_Eu_ru_204::g.49552::m.49552	4.33	2.26	NO	NO		
	Gene.2935::c905_g1_i1_VG_Eu_ru_204::g.2935::m.2935	2.49	1.29	NO	NO		
	Gene.49558::c7682_g1_i2_VG_Eu_ru_204::g.49558::m.49558	4.30	0.00	NO	NO		
	Gene.38883::c6946_g1_i1_VG_Eu_ru_204::g.38883::m.38883	1.97	0.97	YES	NO		
	Gene.108990::c37328_g1_i1_VG_Eu_ru_204::g.108990::m.108990	1.32	0.64	NO	YES		
	Gene.3150::c959_g1_i1_VG_Eu_ru_204::g.3150::m.3150	0.97	0.71	NO	NO		
	Gene.96173::c24285_g1_i1_VG_Eu_ru_204::g.96173::m.96173	0.91	0.57	NO	NO		
	Gene.2603::c783_g1_i1_VG_Eu_ru_204::g.2603::m.2603	0.72	0.76	NO	NO		
	Gene.88372::c17029_g1_i1_VG_Eu_ru_204::g.88372::m.88372	0.47	0.24	NO	NO		
	Gene.87819::c16751_g1_i1_VG_Eu_ru_204::g.87819::m.87819	0.56	0.35	NO	NO		
	Gene.87689::c16691_g1_i1_VG_Eu_ru_204::g.87689::m.87689	0.35	0.21	NO	NO		
	Gene.105187::c32686_g1_i1_VG_Eu_ru_204::g.105187::m.105187	0.32	0.23	NO	NO		
	Gene.81956::c10469_g1_i1_VG_Eu_ru_204::g.81956::m.81956	0.31	0.16	NO	NO		

	Gene.102833::c30968_g1_i1_VG_Eu_ru_204::g.102833::m.102833	0.37	0.64	NO	NO		
	Gene.112632::c39626_g1_i1_VG_Eu_ru_204::g.112632::m.112632	0.61	0.44	NO	NO		
	Gene.91180::c19045_g1_i1_VG_Eu_ru_204::g.91180::m.91180	0.29	0.28	NO	NO		
	Gene.120319::c46978_g1_i1_VG_Eu_ru_204::g.120319::m.120319	0.51	0.67	NO	NO		
	Gene.91779::c19672_g1_i1_VG_Eu_ru_204::g.91779::m.91779	0.32	0.17	NO	NO		
	Gene.94757::c23493_g1_i1_VG_Eu_ru_204::g.94757::m.94757	0.34	0.78	YES	NO		
	Gene.114621::c41775_g1_i1_VG_Eu_ru_204::g.114621::m.114621	0.31	0.48	NO	NO		
	Gene.95299::c23766_g1_i1_VG_Eu_ru_204::g.95299::m.95299	0.36	0.85	NO	NO		
	Gene.119241::c46054_g1_i1_VG_Eu_ru_204::g.119241::m.119241	0.36	0.85	NO	NO		
	Gene.93841::c22248_g1_i1_VG_Eu_ru_204::g.93841::m.93841	0.30	0.73	NO	NO		
	Gene.98848::c26516_g1_i1_VG_Eu_ru_204::g.98848::m.98848	0.29	0.22	YES	NO		
	Gene.105589::c33079_g1_i1_VG_Eu_ru_204::g.105589::m.105589	0.32	0.88	NO	NO		
	Gene.85610::c14196_g1_i1_VG_Eu_ru_204::g.85610::m.85610	0.35	1.02	NO	NO		
	Gene.85960::c14656_g1_i1_VG_Eu_ru_204::g.85960::m.85960	0.21	0.43	NO	NO		
	Gene.84233::c12486_g1_i1_VG_Eu_ru_204::g.84233::m.84233	0.26	0.59	NO	NO		
	Gene.92338::c20266_g1_i1_VG_Eu_ru_204::g.92338::m.92338	0.30	0.90	NO	NO		
	Gene.121950::c48667_g1_i1_VG_Eu_ru_204::g.121950::m.121950	0.24	0.46	NO	NO		
	Gene.109930::c37706_g1_i1_VG_Eu_ru_204::g.109930::m.109930	0.24	0.00	NO	NO		
	Gene.110290::c37919_g1_i1_VG_Eu_ru_204::g.110290::m.110290	0.21	0.59	NO	NO		
	Gene.120563::c47203_g1_i1_VG_Eu_ru_204::g.120563::m.120563	0.25	1.91	NO	NO		
	Gene.122947::c49936_g1_i1_VG_Eu_ru_204::g.122947::m.122947	0.19	0.00	NO	NO		
	Gene.92848::c20908_g1_i1_VG_Eu_ru_204::g.92848::m.92848	0.19	0.95	NO	NO		
	Gene.120512::c47158_g1_i1_VG_Eu_ru_204::g.120512::m.120512	0.16	1.32	NO	NO		
**Peptidase M14**	Gene.16298::c4327_g1_i1_VG_Eu_ru_204::g.16298::m.16298	14.90	7.69	YES	NO		
	Gene.62949::c8433_g1_i1_VG_Eu_ru_204::g.62949::m.62949	7.59	4.31	NO	NO		
	Gene.29528::c6083_g1_i1_VG_Eu_ru_204::g.29528::m.29528	4.21	2.41	YES	NO		
	Gene.4616::c1376_g1_i1_VG_Eu_ru_204::g.4616::m.4616	2.43	1.48	YES	NO		
	Gene.18179::c4601_g1_i1_VG_Eu_ru_204::g.18179::m.18179	1.76	0.95	YES	NO		
	Gene.81090::c9875_g1_i1_VG_Eu_ru_204::g.81090::m.81090	0.54	0.29	NO	NO		
	Gene.111980::c39040_g1_i1_VG_Eu_ru_204::g.111980::m.111980	0.49	0.36	NO	NO		
	Gene.99548::c27280_g1_i1_VG_Eu_ru_204::g.99548::m.99548	0.40	0.19	NO	NO		
	Gene.85633::c14214_g1_i1_VG_Eu_ru_204::g.85633::m.85633	0.30	0.00	NO	NO		
	Gene.114071::c41151_g1_i1_VG_Eu_ru_204::g.114071::m.114071	0.25	0.24	NO	NO		
	Gene.94033::c22571_g1_i1_VG_Eu_ru_204::g.94033::m.94033	0.22	0.71	NO	NO		
	Gene.100219::c28096_g1_i1_VG_Eu_ru_204::g.100219::m.100219	0.25	0.00	NO	NO		
**Peptidase S10**	Gene.7190::c2146_g1_i1_VG_Eu_ru_204::g.7190::m.7190	12.23	7.15	YES	NO		
**Peptidase S1**	Gene.108685::c37210_g2_i1_VG_Eu_ru_204::g.108685::m.108685	27580.99	16772.00	NO	NO		
	Gene.3533::c1049_g1_i2_VG_Eu_ru_204::g.3533::m.3533	17647.83	7357.31	NO	NO		
	Gene.3527::c1049_g1_i1_VG_Eu_ru_204::g.3527::m.3527	18589.80	3203.29	YES	NO		
	Gene.94691::c23464_g2_i1_VG_Eu_ru_204::g.94691::m.94691	13282.56	7946.86	NO	NO		
	Gene.116445::c44470_g1_i1_VG_Eu_ru_204::g.116445::m.116445	5299.94	3315.12	NO	NO		
	Gene.32897::c6401_g1_i1_VG_Eu_ru_204::g.32897::m.32897	2284.21	1488.10	YES	NO		
	Gene.94708::c23473_g1_i1_VG_Eu_ru_204::g.94708::m.94708	1263.94	752.92	YES	NO		
	Gene.28201::c5950_g1_i1_VG_Eu_ru_204::g.28201::m.28201	719.69	373.18	YES	NO		
	Gene.66344::c8591_g1_i1_VG_Eu_ru_204::g.66344::m.66344	101.90	60.98	YES	NO		
	Gene.95279::c23747_g1_i1_VG_Eu_ru_204::g.95279::m.95279	109.17	67.60	YES	NO		
	Gene.48270::c7594_g1_i1_VG_Eu_ru_204::g.48270::m.48270	74.85	45.16	NO	NO		
	Gene.3713::c1081_g1_i1_VG_Eu_ru_204::g.3713::m.3713	63.41	36.66	YES	NO		
	Gene.44622::c7344_g1_i1_VG_Eu_ru_204::g.44622::m.44622	72.51	53.63	NO	NO		
	Gene.12417::c3682_g1_i1_VG_Eu_ru_204::g.12417::m.12417	39.63	23.26	YES	NO		
	Gene.6726::c1959_g1_i1_VG_Eu_ru_204::g.6726::m.6726	50.65	31.79	YES	NO		
	Gene.112040::c39094_g1_i1_VG_Eu_ru_204::g.112040::m.112040	47.72	27.75	NO	NO		
	Gene.1536::c522_g1_i1_VG_Eu_ru_204::g.1536::m.1536	38.74	23.11	YES	NO		
	Gene.94695::c23464_g5_i1_VG_Eu_ru_204::g.94695::m.94695	38.99	25.64	YES	NO		
	Gene.103488::c31358_g1_i1_VG_Eu_ru_204::g.103488::m.103488	34.49	21.62	NO	NO		
	Gene.64671::c8511_g1_i1_VG_Eu_ru_204::g.64671::m.64671	21.84	6.98	NO	NO		
	Gene.64677::c8511_g1_i2_VG_Eu_ru_204::g.64677::m.64677	20.91	6.03	NO	NO		
	Gene.117404::c44932_g1_i1_VG_Eu_ru_204::g.117404::m.117404	16.15	9.82	NO	NO		
	Gene.33673::c6467_g1_i1_VG_Eu_ru_204::g.33673::m.33673	24.19	13.80	YES	NO		
	Gene.28690::c5995_g1_i1_VG_Eu_ru_204::g.28690::m.28690	23.86	15.04	NO	NO		
	Gene.14211::c4007_g1_i1_VG_Eu_ru_204::g.14211::m.14211	21.94	13.17	YES	NO		
	Gene.51445::c7798_g1_i3_VG_Eu_ru_204::g.51445::m.51445	13.30	5.83	YES	NO		
	Gene.51430::c7798_g1_i1_VG_Eu_ru_204::g.51430::m.51430	13.00	1.76	YES	NO		
	Gene.47327::c7525_g1_i1_VG_Eu_ru_204::g.47327::m.47327	16.24	10.51	YES	NO		
	Gene.97431::c25195_g1_i1_VG_Eu_ru_204::g.97431::m.97431	15.42	9.33	YES	NO		
	Gene.17551::c4500_g1_i1_VG_Eu_ru_204::g.17551::m.17551	18.13	10.64	YES	NO		
	Gene.102485::c30773_g1_i1_VG_Eu_ru_204::g.102485::m.102485	15.48	8.21	NO	NO		
	Gene.102234::c30650_g1_i1_VG_Eu_ru_204::g.102234::m.102234	15.35	8.79	NO	NO		
	Gene.48562::c7612_g1_i1_VG_Eu_ru_204::g.48562::m.48562	10.20	6.07	YES	NO		
	Gene.58657::c8212_g1_i3_VG_Eu_ru_204::g.58657::m.58657	9.77	4.30	NO	NO		
	Gene.1344::c453_g1_i1_VG_Eu_ru_204::g.1344::m.1344	11.02	7.78	YES	NO		
	Gene.73391::c8859_g1_i1_VG_Eu_ru_204::g.73391::m.73391	10.92	8.43	YES	NO		
	Gene.51438::c7798_g1_i2_VG_Eu_ru_204::g.51438::m.51438	11.88	0.00	NO	NO		
	Gene.46185::c7467_g1_i1_VG_Eu_ru_204::g.46185::m.46185	9.82	6.31	YES	NO		
	Gene.35896::c6652_g1_i1_VG_Eu_ru_204::g.35896::m.35896	6.51	3.41	YES	NO		
	Gene.907::c285_g1_i1_VG_Eu_ru_204::g.907::m.907	8.72	5.66	YES	NO		
	Gene.3941::c1136_g1_i1_VG_Eu_ru_204::g.3941::m.3941	8.50	5.04	YES	NO		
	Gene.12247::c3643_g1_i1_VG_Eu_ru_204::g.12247::m.12247	7.27	4.05	YES	NO		
	Gene.103441::c31324_g1_i1_VG_Eu_ru_204::g.103441::m.103441	7.67	5.16	NO	NO		
	Gene.6510::c1904_g1_i1_VG_Eu_ru_204::g.6510::m.6510	9.67	5.69	YES	NO		
	Gene.51355::c7793_g2_i1_VG_Eu_ru_204::g.51355::m.51355	6.81	3.97	YES	NO		
	Gene.58652::c8212_g1_i1_VG_Eu_ru_204::g.58652::m.58652	8.24	1.59	NO	NO		
	Gene.49862::c7700_g1_i1_VG_Eu_ru_204::g.49862::m.49862	6.52	4.33	YES	NO		
	Gene.17400::c4475_g1_i1_VG_Eu_ru_204::g.17400::m.17400	4.16	2.50	YES	NO		
	Gene.20016::c4911_g1_i1_VG_Eu_ru_204::g.20016::m.20016	5.43	3.42	NO	NO		
	Gene.14840::c4103_g1_i2_VG_Eu_ru_204::g.14840::m.14840	5.29	3.55	YES	NO		
	Gene.52576::c7883_g1_i2_VG_Eu_ru_204::g.52576::m.52576	4.61	2.88	YES	NO		
	Gene.58653::c8212_g1_i1_VG_Eu_ru_204::g.58653::m.58653	8.45	0.96	NO	NO		
	Gene.10283::c3071_g1_i1_VG_Eu_ru_204::g.10283::m.10283	4.48	2.22	YES	NO		
	Gene.118633::c45639_g1_i1_VG_Eu_ru_204::g.118633::m.118633	2.42	1.39	NO	NO		
	Gene.52574::c7883_g1_i1_VG_Eu_ru_204::g.52574::m.52574	3.48	2.19	YES	NO		
	Gene.47849::c7566_g1_i1_VG_Eu_ru_204::g.47849::m.47849	3.71	1.35	NO	NO		
	Gene.47856::c7566_g1_i2_VG_Eu_ru_204::g.47856::m.47856	3.71	1.35	NO	NO		
	Gene.108756::c37236_g1_i1_VG_Eu_ru_204::g.108756::m.108756	1.13	0.57	NO	NO		excluded
	Gene.18064::c4584_g1_i1_VG_Eu_ru_204::g.18064::m.18064	1.52	0.67	YES	NO		
	Gene.97988::c25688_g1_i1_VG_Eu_ru_204::g.97988::m.97988	3.39	2.20	YES	NO		
	Gene.3898::c1126_g1_i1_VG_Eu_ru_204::g.3898::m.3898	2.65	1.46	YES	NO		
	Gene.43795::c7293_g1_i1_VG_Eu_ru_204::g.43795::m.43795	2.84	1.71	YES	NO		
	Gene.55647::c8044_g1_i1_VG_Eu_ru_204::g.55647::m.55647	2.16	1.33	YES	NO		
	Gene.90400::c18414_g1_i1_VG_Eu_ru_204::g.90400::m.90400	1.59	0.89	NO	NO		
	Gene.87056::c16423_g1_i1_VG_Eu_ru_204::g.87056::m.87056	1.63	1.12	YES	NO		
	Gene.1768::c607_g1_i1_VG_Eu_ru_204::g.1768::m.1768	1.64	0.98	YES	NO		
	Gene.13593::c3896_g1_i1_VG_Eu_ru_204::g.13593::m.13593	1.73	1.56	YES	NO		
	Gene.10989::c3276_g1_i1_VG_Eu_ru_204::g.10989::m.10989	1.69	0.84	YES	NO		
	Gene.10894::c3243_g1_i1_VG_Eu_ru_204::g.10894::m.10894	1.07	0.50	YES	NO		
	Gene.4004::c1152_g1_i1_VG_Eu_ru_204::g.4004::m.4004	1.80	1.08	YES	NO		
	Gene.96884::c24774_g1_i1_VG_Eu_ru_204::g.96884::m.96884	1.66	0.78	YES	NO		
	Gene.15060::c4147_g1_i1_VG_Eu_ru_204::g.15060::m.15060	0.74	0.39	NO	NO		
	Gene.43816::c7297_g1_i1_VG_Eu_ru_204::g.43816::m.43816	1.17	0.60	YES	NO		
	Gene.1394::c478_g1_i1_VG_Eu_ru_204::g.1394::m.1394	0.99	0.58	YES	NO		
	Gene.94601::c23431_g1_i1_VG_Eu_ru_204::g.94601::m.94601	1.04	0.55	YES	NO		
	Gene.82355::c10772_g1_i1_VG_Eu_ru_204::g.82355::m.82355	1.99	1.96	NO	NO		
	Gene.11436::c3441_g1_i2_VG_Eu_ru_204::g.11436::m.11436	1.24	0.53	NO	NO		
	Gene.2303::c724_g1_i2_VG_Eu_ru_204::g.2303::m.2303	0.98	0.52	NO	NO		
	Gene.9674::c2893_g1_i1_VG_Eu_ru_204::g.9674::m.9674	0.62	0.35	YES	NO		
	Gene.11434::c3441_g1_i1_VG_Eu_ru_204::g.11434::m.11434	1.18	0.30	NO	NO		
	Gene.89826::c17948_g1_i1_VG_Eu_ru_204::g.89826::m.89826	0.68	0.50	YES	NO		
	Gene.14833::c4101_g1_i1_VG_Eu_ru_204::g.14833::m.14833	1.33	1.29	NO	NO		
	Gene.14962::c4123_g1_i1_VG_Eu_ru_204::g.14962::m.14962	1.04	0.65	NO	NO		
	Gene.117139::c44822_g1_i1_VG_Eu_ru_204::g.117139::m.117139	0.79	0.36	NO	NO		
	Gene.35815::c6645_g2_i1_VG_Eu_ru_204::g.35815::m.35815	0.78	0.19	NO	NO		
	Gene.82211::c10654_g1_i1_VG_Eu_ru_204::g.82211::m.82211	0.84	0.56	NO	NO		
	Gene.7337::c2196_g1_i1_VG_Eu_ru_204::g.7337::m.7337	0.69	0.47	NO	NO		

	Gene.145::c57_g1_i1_VG_Eu_ru_204::g.145::m.145	1.02	0.89	NO	NO		
	Gene.2299::c724_g1_i1_VG_Eu_ru_204::g.2299::m.2299	1.04	0.00	NO	NO		
	Gene.117462::c44953_g1_i1_VG_Eu_ru_204::g.117462::m.117462	0.66	0.51	NO	NO		
	Gene.3147::c957_g1_i1_VG_Eu_ru_204::g.3147::m.3147	0.58	0.47	YES	NO		
	Gene.35821::c6645_g4_i1_VG_Eu_ru_204::g.35821::m.35821	0.46	0.34	NO	NO		
	Gene.95685::c23992_g1_i1_VG_Eu_ru_204::g.95685::m.95685	0.85	0.72	NO	NO		
	Gene.81289::c10000_g1_i1_VG_Eu_ru_204::g.81289::m.81289	0.65	0.62	NO	NO		
	Gene.73394::c8859_g1_i2_VG_Eu_ru_204::g.73394::m.73394	1.49	2.12	NO	NO		
	Gene.4766::c1421_g1_i1_VG_Eu_ru_204::g.4766::m.4766	0.55	0.36	NO	NO		
	Gene.95761::c24039_g1_i1_VG_Eu_ru_204::g.95761::m.95761	0.38	0.14	NO	NO		
	Gene.116799::c44628_g1_i1_VG_Eu_ru_204::g.116799::m.116799	0.90	0.86	NO	NO		
	Gene.121265::c47878_g1_i1_VG_Eu_ru_204::g.121265::m.121265	0.73	0.70	NO	NO		
	Gene.97637::c25385_g1_i1_VG_Eu_ru_204::g.97637::m.97637	0.47	0.30	NO	NO		
	Gene.4918::c1501_g1_i2_VG_Eu_ru_204::g.4918::m.4918	0.56	0.55	NO	NO		
	Gene.106239::c33759_g1_i1_VG_Eu_ru_204::g.106239::m.106239	0.48	0.17	NO	NO		
	Gene.119970::c46654_g1_i1_VG_Eu_ru_204::g.119970::m.119970	0.59	0.24	NO	NO		
	Gene.7323::c2189_g1_i1_VG_Eu_ru_204::g.7323::m.7323	1.39	3.78	NO	NO		
	Gene.120396::c47046_g1_i1_VG_Eu_ru_204::g.120396::m.120396	0.66	0.74	NO	NO		
	Gene.120642::c47275_g1_i1_VG_Eu_ru_204::g.120642::m.120642	0.32	0.17	NO	NO		
	Gene.97650::c25399_g1_i1_VG_Eu_ru_204::g.97650::m.97650	0.80	0.68	NO	NO		
	Gene.102550::c30805_g1_i1_VG_Eu_ru_204::g.102550::m.102550	0.79	0.66	NO	NO		
	Gene.119120::c45971_g1_i1_VG_Eu_ru_204::g.119120::m.119120	0.37	0.36	NO	NO		
	Gene.119508::c46281_g1_i1_VG_Eu_ru_204::g.119508::m.119508	0.45	0.38	NO	NO		
	Gene.90955::c18847_g1_i1_VG_Eu_ru_204::g.90955::m.90955	0.58	0.63	NO	NO		
	Gene.110118::c37831_g1_i1_VG_Eu_ru_204::g.110118::m.110118	0.42	0.36	NO	NO		
	Gene.80835::c9730_g1_i1_VG_Eu_ru_204::g.80835::m.80835	0.59	0.70	YES	NO		
	Gene.4906::c1491_g1_i1_VG_Eu_ru_204::g.4906::m.4906	0.43	0.34	NO	NO		
	Gene.97893::c25621_g1_i1_VG_Eu_ru_204::g.97893::m.97893	0.39	0.14	NO	NO		
	Gene.106149::c33652_g1_i1_VG_Eu_ru_204::g.106149::m.106149	0.37	0.39	NO	NO		
	Gene.112466::c39454_g1_i1_VG_Eu_ru_204::g.112466::m.112466	0.38	0.30	NO	NO		
	Gene.118149::c45319_g1_i1_VG_Eu_ru_204::g.118149::m.118149	0.38	0.53	NO	NO		
	Gene.83346::c11587_g1_i1_VG_Eu_ru_204::g.83346::m.83346	0.51	0.74	NO	NO		
	Gene.89857::c17967_g1_i1_VG_Eu_ru_204::g.89857::m.89857	0.30	0.31	NO	NO		
	Gene.88075::c16885_g1_i1_VG_Eu_ru_204::g.88075::m.88075	0.32	0.24	YES	NO		
	Gene.92612::c20616_g1_i1_VG_Eu_ru_204::g.92612::m.92612	0.27	0.17	NO	NO		
	Gene.105420::c32922_g1_i1_VG_Eu_ru_204::g.105420::m.105420	0.40	0.46	NO	NO		
	Gene.115371::c42909_g1_i1_VG_Eu_ru_204::g.115371::m.115371	0.51	0.78	NO	NO		
	Gene.90498::c18481_g1_i1_VG_Eu_ru_204::g.90498::m.90498	0.39	0.63	NO	NO		
	Gene.98314::c25968_g1_i1_VG_Eu_ru_204::g.98314::m.98314	0.38	0.35	NO	NO		
	Gene.99154::c26826_g1_i1_VG_Eu_ru_204::g.99154::m.99154	0.39	0.50	NO	NO		
	Gene.79262::c9007_g1_i1_VG_Eu_ru_204::g.79262::m.79262	0.32	0.26	NO	NO		
	Gene.79951::c9290_g1_i1_VG_Eu_ru_204::g.79951::m.79951	0.28	0.20	NO	NO		
	Gene.98580::c26233_g1_i1_VG_Eu_ru_204::g.98580::m.98580	0.26	0.28	NO	NO		
	Gene.99752::c27498_g1_i1_VG_Eu_ru_204::g.99752::m.99752	0.39	1.01	NO	NO		
	Gene.100661::c28715_g1_i1_VG_Eu_ru_204::g.100661::m.100661	0.44	1.33	NO	NO		
	Gene.113587::c40598_g1_i1_VG_Eu_ru_204::g.113587::m.113587	0.29	0.17	NO	NO		
	Gene.90273::c18318_g1_i1_VG_Eu_ru_204::g.90273::m.90273	0.33	0.63	YES	NO		
	Gene.98592::c26240_g1_i1_VG_Eu_ru_204::g.98592::m.98592	0.39	0.17	NO	NO		
	Gene.108846::c37266_g1_i1_VG_Eu_ru_204::g.108846::m.108846	0.26	0.23	NO	NO		
	Gene.83811::c12031_g1_i1_VG_Eu_ru_204::g.83811::m.83811	0.38	0.83	NO	NO		
	Gene.93713::c22047_g1_i1_VG_Eu_ru_204::g.93713::m.93713	0.36	0.37	NO	NO		
	Gene.93820::c22204_g1_i1_VG_Eu_ru_204::g.93820::m.93820	0.29	0.35	NO	NO		
	Gene.115224::c42673_g1_i1_VG_Eu_ru_204::g.115224::m.115224	0.30	0.24	NO	NO		
	Gene.122052::c48780_g1_i1_VG_Eu_ru_204::g.122052::m.122052	0.36	0.87	NO	NO		
	Gene.100064::c27883_g1_i1_VG_Eu_ru_204::g.100064::m.100064	0.34	0.39	NO	NO		
	Gene.104965::c32493_g1_i1_VG_Eu_ru_204::g.104965::m.104965	0.44	1.14	NO	NO		
	Gene.106248::c33768_g1_i1_VG_Eu_ru_204::g.106248::m.106248	0.48	0.97	NO	NO		
	Gene.4917::c1501_g1_i1_VG_Eu_ru_204::g.4917::m.4917	0.31	0.00	NO	NO		
	Gene.12065::c3602_g1_i1_VG_Eu_ru_204::g.12065::m.12065	0.46	0.00	NO	NO		
	Gene.111517::c38723_g1_i1_VG_Eu_ru_204::g.111517::m.111517	0.32	0.64	NO	NO		
	Gene.81702::c10281_g1_i1_VG_Eu_ru_204::g.81702::m.81702	0.23	0.00	NO	NO		
	Gene.84415::c12712_g1_i1_VG_Eu_ru_204::g.84415::m.84415	0.25	0.00	NO	NO		
	Gene.92196::c20095_g1_i1_VG_Eu_ru_204::g.92196::m.92196	0.34	0.35	NO	NO		
	Gene.93027::c21130_g1_i1_VG_Eu_ru_204::g.93027::m.93027	0.24	0.30	NO	NO		
	Gene.115276::c42765_g1_i1_VG_Eu_ru_204::g.115276::m.115276	0.29	0.46	NO	NO		
	Gene.89771::c17902_g1_i1_VG_Eu_ru_204::g.89771::m.89771	0.32	0.40	NO	NO		
	Gene.91170::c19038_g1_i1_VG_Eu_ru_204::g.91170::m.91170	0.32	0.00	NO	NO		
	Gene.100118::c27964_g1_i1_VG_Eu_ru_204::g.100118::m.100118	0.27	0.52	NO	NO		
	Gene.103867::c31621_g1_i1_VG_Eu_ru_204::g.103867::m.103867	0.28	0.59	NO	NO		
	Gene.104508::c32119_g1_i1_VG_Eu_ru_204::g.104508::m.104508	0.26	0.24	NO	NO		
	Gene.107848::c35700_g1_i1_VG_Eu_ru_204::g.107848::m.107848	0.20	0.13	NO	NO		
	Gene.108303::c36479_g1_i1_VG_Eu_ru_204::g.108303::m.108303	0.29	0.32	NO	NO		
	Gene.113063::c40049_g1_i1_VG_Eu_ru_204::g.113063::m.113063	0.27	0.27	NO	NO		
	Gene.113083::c40092_g1_i1_VG_Eu_ru_204::g.113083::m.113083	0.30	0.70	NO	NO		
	Gene.121517::c48164_g1_i1_VG_Eu_ru_204::g.121517::m.121517	0.35	0.51	NO	NO		
	Gene.122264::c49045_g1_i1_VG_Eu_ru_204::g.122264::m.122264	0.37	1.19	NO	NO		
	Gene.122714::c49647_g1_i1_VG_Eu_ru_204::g.122714::m.122714	0.25	0.21	NO	NO		
	Gene.86260::c15144_g1_i1_VG_Eu_ru_204::g.86260::m.86260	0.17	0.25	NO	NO		
	Gene.105349::c32844_g1_i1_VG_Eu_ru_204::g.105349::m.105349	0.26	1.09	NO	NO		
	Gene.113079::c40080_g1_i1_VG_Eu_ru_204::g.113079::m.113079	0.21	0.20	NO	NO		
	Gene.85964::c14669_g1_i1_VG_Eu_ru_204::g.85964::m.85964	0.23	0.44	NO	NO		
	Gene.92483::c20456_g1_i1_VG_Eu_ru_204::g.92483::m.92483	0.25	1.62	NO	NO		
	Gene.97936::c25646_g1_i1_VG_Eu_ru_204::g.97936::m.97936	0.28	1.50	NO	NO		
	Gene.106440::c33973_g1_i1_VG_Eu_ru_204::g.106440::m.106440	0.29	0.79	NO	NO		
	Gene.115020::c42353_g1_i1_VG_Eu_ru_204::g.115020::m.115020	0.20	0.31	NO	NO		
	Gene.80798::c9709_g1_i1_VG_Eu_ru_204::g.80798::m.80798	0.25	0.51	NO	NO		
	Gene.85064::c13513_g1_i1_VG_Eu_ru_204::g.85064::m.85064	0.20	0.00	NO	NO		
	Gene.85836::c14473_g1_i1_VG_Eu_ru_204::g.85836::m.85836	0.22	0.00	NO	NO		
	Gene.86097::c14848_g1_i1_VG_Eu_ru_204::g.86097::m.86097	0.18	0.79	NO	NO		
	Gene.88059::c16878_g1_i1_VG_Eu_ru_204::g.88059::m.88059	0.18	0.38	NO	NO		
	Gene.102148::c30598_g1_i1_VG_Eu_ru_204::g.102148::m.102148	0.20	0.00	NO	NO		
	Gene.107798::c35638_g1_i1_VG_Eu_ru_204::g.107798::m.107798	0.25	0.95	NO	NO		
	Gene.113118::c40129_g1_i1_VG_Eu_ru_204::g.113118::m.113118	0.26	2.03	NO	NO		
	Gene.115964::c43820_g1_i1_VG_Eu_ru_204::g.115964::m.115964	0.24	0.00	NO	NO		
	Gene.116186::c44277_g1_i1_VG_Eu_ru_204::g.116186::m.116186	0.27	2.29	NO	NO		
	Gene.116858::c44665_g1_i1_VG_Eu_ru_204::g.116858::m.116858	0.19	0.44	NO	NO		
	Gene.121410::c48038_g1_i1_VG_Eu_ru_204::g.121410::m.121410	0.21	0.63	NO	NO		
	Gene.121782::c48493_g1_i1_VG_Eu_ru_204::g.121782::m.121782	0.20	0.00	NO	NO		
	Gene.93440::c21650_g1_i1_VG_Eu_ru_204::g.93440::m.93440	0.15	1.70	NO	NO		
	Gene.97210::c25004_g1_i1_VG_Eu_ru_204::g.97210::m.97210	0.18	0.00	NO	NO		
	Gene.113377::c40386_g1_i1_VG_Eu_ru_204::g.113377::m.113377	0.20	2.29	NO	NO		
	Gene.114168::c41277_g1_i1_VG_Eu_ru_204::g.114168::m.114168	0.17	2.25	NO	NO		
	Gene.115061::c42420_g1_i1_VG_Eu_ru_204::g.115061::m.115061	0.16	1.19	NO	NO		
	Gene.87674::c16683_g1_i1_VG_Eu_ru_204::g.87674::m.87674	0.12	0.90	NO	NO		
	Gene.94707::c23471_g1_i1_VG_Eu_ru_204::g.94707::m.94707	0.13	0.00	NO	NO		
	Gene.35819::c6645_g2_i2_VG_Eu_ru_204::g.35819::m.35819	0.00	0.00	NO	NO		
**Phospholipase A2**	Gene.4975::c1525_g1_i1_VG_Eu_ru_204::g.4975::m.4975	924.34	606.83	YES	NO		
	Gene.22840::c5296_g1_i3_VG_Eu_ru_204::g.22840::m.22840	31.24	12.60	NO	NO		
	Gene.22838::c5296_g1_i2_VG_Eu_ru_204::g.22838::m.22838	36.72	15.71	NO	NO		
	Gene.45279::c7390_g1_i1_VG_Eu_ru_204::g.45279::m.45279	4.10	2.38	YES	NO		
	Gene.16727::c4387_g1_i1_VG_Eu_ru_204::g.16727::m.16727	4.87	1.58	NO	NO		
	Gene.16731::c4387_g1_i2_VG_Eu_ru_204::g.16731::m.16731	4.87	1.58	NO	NO		
	Gene.31394::c6262_g1_i1_VG_Eu_ru_204::g.31394::m.31394	4.21	2.58	NO	NO		
	Gene.82786::c11093_g1_i1_VG_Eu_ru_204::g.82786::m.82786	0.26	0.00	NO	NO		
**Pimplin**	Gene.59797::c8273_g2_i1_VG_Eu_ru_204::g.59797::m.59797	17670.95	13270.00	NO	NO		excluded
	Gene.59801::c8273_g2_i2_VG_Eu_ru_204::g.59801::m.59801	16121.13	8559.65	NO	NO		excluded
	Gene.15272::c4180_g1_i1_VG_Eu_ru_204::g.15272::m.15272	1.60	0.52	NO	NO		excluded
	Gene.15289::c4180_g1_i3_VG_Eu_ru_204::g.15289::m.15289	1.60	0.52	NO	NO		excluded
	Gene.4686::c1390_g1_i1_VG_Eu_ru_204::g.4686::m.4686	0.29	0.22	NO	NO		
**Serpin**	Gene.17422::c4479_g2_i1_VG_Eu_ru_204::g.17422::m.17422	9620.39	5305.42	YES	NO		
	Gene.62059::c8395_g1_i2_VG_Eu_ru_204::g.62059::m.62059	165.54	85.83	YES	NO		
	Gene.44484::c7332_g1_i1_VG_Eu_ru_204::g.44484::m.44484	87.61	33.91	YES	NO		
	Gene.44486::c7332_g1_i2_VG_Eu_ru_204::g.44486::m.44486	87.16	16.35	YES	NO		

	Gene.62065::c8395_g1_i3_VG_Eu_ru_204::g.62065::m.62065	75.13	10.86	NO	NO		
	Gene.17425::c4479_g3_i1_VG_Eu_ru_204::g.17425::m.17425	72.52	51.85	NO	NO		
	Gene.109053::c37347_g1_i1_VG_Eu_ru_204::g.109053::m.109053	30.36	16.49	NO	NO		
	Gene.62056::c8395_g1_i1_VG_Eu_ru_204::g.62056::m.62056	28.55	14.21	NO	NO		
	Gene.82466::c10855_g1_i1_VG_Eu_ru_204::g.82466::m.82466	13.02	7.66	YES	NO		
	Gene.12721::c3734_g1_i1_VG_Eu_ru_204::g.12721::m.12721	12.45	7.10	YES	NO		
	Gene.82659::c11006_g1_i1_VG_Eu_ru_204::g.82659::m.82659	9.47	5.06	YES	NO		
	Gene.54137::c7959_g1_i1_VG_Eu_ru_204::g.54137::m.54137	5.92	1.44	YES	NO		
	Gene.54143::c7959_g1_i2_VG_Eu_ru_204::g.54143::m.54143	5.92	1.44	YES	NO		
	Gene.51961::c7834_g1_i1_VG_Eu_ru_204::g.51961::m.51961	2.89	0.78	YES	NO		
	Gene.51972::c7834_g1_i3_VG_Eu_ru_204::g.51972::m.51972	2.89	0.78	YES	NO		
	Gene.14956::c4122_g1_i1_VG_Eu_ru_204::g.14956::m.14956	3.79	2.23	YES	NO		
	Gene.51966::c7834_g1_i2_VG_Eu_ru_204::g.51966::m.51966	2.60	0.00	YES	NO		
	Gene.35548::c6620_g1_i1_VG_Eu_ru_204::g.35548::m.35548	13.52	83.52	NO	NO		
	Gene.51967::c7834_g1_i2_VG_Eu_ru_204::g.51967::m.51967	2.92	0.03	NO	NO		
	Gene.11367::c3418_g2_i1_VG_Eu_ru_204::g.11367::m.11367	3.17	2.77	NO	NO		
	Gene.11362::c3418_g1_i1_VG_Eu_ru_204::g.11362::m.11362	3.17	1.96	NO	NO		
	Gene.119789::c46500_g1_i1_VG_Eu_ru_204::g.119789::m.119789	7.90	17.23	NO	NO		
	Gene.81245::c9973_g1_i1_VG_Eu_ru_204::g.81245::m.81245	0.92	0.53	NO	NO		
	Gene.6934::c2033_g1_i1_VG_Eu_ru_204::g.6934::m.6934	0.60	0.23	NO	NO		
	Gene.98401::c26057_g1_i1_VG_Eu_ru_204::g.98401::m.98401	2.66	4.82	NO	NO		
	Gene.111600::c38764_g1_i1_VG_Eu_ru_204::g.111600::m.111600	0.61	0.54	NO	NO		
	Gene.108702::c37214_g1_i1_VG_Eu_ru_204::g.108702::m.108702	0.49	0.30	NO	NO		
	Gene.109935::c37708_g1_i1_VG_Eu_ru_204::g.109935::m.109935	1.71	6.30	NO	NO		
	Gene.12838::c3751_g1_i1_VG_Eu_ru_204::g.12838::m.12838	1.23	7.36	NO	NO		
	Gene.7857::c2369_g1_i1_VG_Eu_ru_204::g.7857::m.7857	1.20	8.32	NO	NO		
	Gene.13774::c3940_g1_i1_VG_Eu_ru_204::g.13774::m.13774	1.25	9.93	NO	NO		
	Gene.117269::c44879_g1_i1_VG_Eu_ru_204::g.117269::m.117269	0.40	0.10	NO	NO		
	Gene.105075::c32584_g1_i1_VG_Eu_ru_204::g.105075::m.105075	0.38	1.02	NO	NO		
	Gene.5258::c1627_g1_i1_VG_Eu_ru_204::g.5258::m.5258	0.60	2.65	NO	NO		
	Gene.85457::c14004_g1_i1_VG_Eu_ru_204::g.85457::m.85457	0.40	1.53	NO	NO		
	Gene.96615::c24587_g1_i1_VG_Eu_ru_204::g.96615::m.96615	0.25	0.00	NO	NO		
	Gene.101193::c29545_g1_i1_VG_Eu_ru_204::g.101193::m.101193	0.25	1.53	NO	NO		
	Gene.112639::c39631_g1_i1_VG_Eu_ru_204::g.112639::m.112639	0.16	0.31	NO	NO		
	Gene.114211::c41331_g1_i1_VG_Eu_ru_204::g.114211::m.114211	0.23	0.79	NO	NO		
**Sphingomyelinase**	Gene.28959::c6029_g1_i1_VG_Eu_ru_204::g.28959::m.28959	2.03	1.18	YES	NO		
	Gene.28975::c6029_g1_i2_VG_Eu_ru_204::g.28975::m.28975	2.02	0.00	YES	NO		
	Gene.14951::c4121_g1_i2_VG_Eu_ru_204::g.14951::m.14951	2.97	1.69	YES	NO		
	Gene.14947::c4121_g1_i1_VG_Eu_ru_204::g.14947::m.14947	2.34	0.00	YES	NO		
	Gene.119599::c46351_g1_i1_VG_Eu_ru_204::g.119599::m.119599	0.75	0.52	NO	NO		
	Gene.80852::c9741_g1_i1_VG_Eu_ru_204::g.80852::m.80852	0.86	0.38	NO	NO		
**SVWC-domain 1 (Asilidin8)**	Gene.4808::c1438_g1_i1_VG_Eu_ru_204::g.4808::m.4808	1443.99	1093.16	NO	YES	U-Asilidin8-Eru1a	
	Gene.3809::c1109_g1_i2_VG_Eu_ru_204::g.3809::m.3809	3.85	4.20	YES	NO		
	Gene.99960::c27749_g1_i1_VG_Eu_ru_204::g.99960::m.99960	3.62	4.28	YES	NO		
**SVWC-domain 2 (Asilidin9)**	Gene.34546::c6542_g1_i1_VG_Eu_ru_204::g.34546::m.34546	781.60	529.17	NO	YES	U-Asilidin9-Eru1a	
	Gene.34549::c6542_g1_i2_VG_Eu_ru_204::g.34549::m.34549	532.03	106.81	YES	NO		
	Gene.103013::c31070_g1_i1_VG_Eu_ru_204::g.103013::m.103013	1.40	1.23	NO	NO		
**Venom acid phosphatase-like**	Gene.61465::c8353_g2_i1_VG_Eu_ru_204::g.61465::m.61465	2379.59	1386.51	YES	NO		
	Gene.61466::c8353_g2_i1_VG_Eu_ru_204::g.61466::m.61466	59.30	45.27	NO	NO		
	Gene.15759::c4249_g1_i1_VG_Eu_ru_204::g.15759::m.15759	9.95	5.32	NO	NO		
	Gene.61467::c8353_g2_i1_VG_Eu_ru_204::g.61467::m.61467	21.32	17.96	NO	NO		
	Gene.109467::c37501_g1_i1_VG_Eu_ru_204::g.109467::m.109467	6.23	3.67	NO	NO		
	Gene.58545::c8208_g1_i2_VG_Eu_ru_204::g.58545::m.58545	4.64	2.48	NO	NO		
	Gene.58536::c8208_g1_i1_VG_Eu_ru_204::g.58536::m.58536	4.05	0.08	NO	NO		
