# Supplementary material for: A Dipteran’s Novel Sucker Punch: Evolution of Arthropod Atypical Venom with a Neurotoxic Component in Robber Flies (Asilidae, Diptera)
Source: Toxins (Basel). 2018 Jan 5;10(1):29. doi: 10.3390/toxins10010029 (PMC5793116; doi:10.3390/toxins10010029)
Supplement: Supplementary file 1 [file toxins-10-00029-s001.zip › toxins-254261-supplementary/toxins-254261 supplementary file 4.pdf]

Protein Class	Contig ID	TPM - Segemehl	TPM - Kallisto	Signal Peptide	false positive
**Aegyptin**	Gene.35590::c8084_BT_Ma_ar_201_g1_i3::g.35590::m.35590	26.87	18.80	NO	only body tissue
**Alpha-amylase**	Gene.9814::c3546_BT_Ma_ar_201_g1_i1::g.9814::m.9814	67.28	37.91	YES	
**Anophelin**	no hit	0	0	X	
**Apyrase**	Gene.7895::c2798_BT_Ma_ar_201_g1_i1::g.7895::m.7895	21.59	11.43	YES	only body tissue
	Gene.51259::c9670_BT_Ma_ar_201_g1_i2::g.51259::m.51259	14.43	6.41	NO	only body tissue
	Gene.106645::c12823_BT_Ma_ar_201_g1_i2::g.106645::m.106645	16.35	9.05	NO	only body tissue
	Gene.106641::c12823_BT_Ma_ar_201_g1_i1::g.106641::m.106641	15.97	0.00	NO	only body tissue
	Gene.51253::c9670_BT_Ma_ar_201_g1_i1::g.51253::m.51253	14.59	2.72	NO	only body tissue
	Gene.106652::c12823_BT_Ma_ar_201_g1_i3::g.106652::m.106652	9.14	3.95	NO	only body tissue
	Gene.106655::c12823_BT_Ma_ar_201_g1_i4::g.106655::m.106655	8.34	2.63	NO	only body tissue
	Gene.154379::c55460_BT_Ma_ar_201_g1_i1::g.154379::m.154379	1.78	1.38	NO	only body tissue
	Gene.148940::c49510_BT_Ma_ar_201_g1_i1::g.148940::m.148940	1.79	1.04	NO	only body tissue
	Gene.111936::c14279_BT_Ma_ar_201_g1_i1::g.111936::m.111936	1.55	1.07	NO	only body tissue
**CAP**	Gene.32398::c7699_BT_Ma_ar_201_g1_i1::g.32398::m.32398	283.74	195.85	YES	
	Gene.65498::c10811_BT_Ma_ar_201_g1_i1::g.65498::m.65498	194.01	159.23	NO	
	Gene.44599::c9047_BT_Ma_ar_201_g1_i1::g.44599::m.44599	48.28	39.76	YES	
	Gene.69514::c11120_BT_Ma_ar_201_g1_i2::g.69514::m.69514	29.93	15.67	YES	
	Gene.60081::c10434_BT_Ma_ar_201_g1_i1::g.60081::m.60081	32.19	22.04	YES	
	Gene.45568::c9142_BT_Ma_ar_201_g1_i1::g.45568::m.45568	25.34	11.66	YES	
	Gene.17547::c5350_BT_Ma_ar_201_g1_i1::g.17547::m.17547	34.76	11.00	NO	
	Gene.44268::c9020_BT_Ma_ar_201_g1_i2::g.44268::m.44268	33.17	24.79	YES	
	Gene.17550::c5350_BT_Ma_ar_201_g1_i2::g.17550::m.17550	36.95	14.76	NO	
	Gene.35207::c8036_BT_Ma_ar_201_g1_i1::g.35207::m.35207	29.86	36.95	NO	
	Gene.45575::c9142_BT_Ma_ar_201_g1_i2::g.45575::m.45575	25.30	9.91	NO	
	Gene.69522::c11120_BT_Ma_ar_201_g1_i4::g.69522::m.69522	28.59	0.25	NO	
	Gene.33726::c7866_BT_Ma_ar_201_g1_i1::g.33726::m.33726	7.26	5.22	NO	
	Gene.69511::c11120_BT_Ma_ar_201_g1_i1::g.69511::m.69511	26.07	2.54	NO	
	Gene.69518::c11120_BT_Ma_ar_201_g1_i3::g.69518::m.69518	26.07	2.54	NO	
	Gene.45576::c9142_BT_Ma_ar_201_g1_i2::g.45576::m.45576	20.02	6.02	YES	
	Gene.25302::c6709_BT_Ma_ar_201_g1_i1::g.25302::m.25302	10.29	8.36	NO	
	Gene.44263::c9020_BT_Ma_ar_201_g1_i1::g.44263::m.44263	20.86	17.63	NO	
	Gene.35124::c8028_BT_Ma_ar_201_g1_i1::g.35124::m.35124	9.09	9.57	NO	
	Gene.30113::c7405_BT_Ma_ar_201_g1_i1::g.30113::m.30113	4.51	3.10	YES	
	Gene.10594::c3877_BT_Ma_ar_201_g1_i1::g.10594::m.10594	3.92	3.51	YES	
	Gene.35878::c8121_BT_Ma_ar_201_g1_i2::g.35878::m.35878	3.99	2.33	YES	
	Gene.35876::c8121_BT_Ma_ar_201_g1_i1::g.35876::m.35876	4.15	1.89	NO	
	Gene.35885::c8121_BT_Ma_ar_201_g1_i4::g.35885::m.35885	3.79	0.45	YES	
	Gene.109742::c12982_BT_Ma_ar_201_g1_i1::g.109742::m.109742	1.83	1.56	NO	
	Gene.35879::c8121_BT_Ma_ar_201_g1_i2::g.35879::m.35879	3.82	1.31	NO	
	Gene.35886::c8121_BT_Ma_ar_201_g1_i4::g.35886::m.35886	3.82	1.31	NO	
	Gene.119744::c22063_BT_Ma_ar_201_g1_i1::g.119744::m.119744	1.84	1.66	NO	
	Gene.117277::c19556_BT_Ma_ar_201_g1_i1::g.117277::m.117277	1.37	5.48	NO	
**Chitinase**	Gene.68467::c11045_BT_Ma_ar_201_g1_i1::g.68467::m.68467	866.74	532.55	YES	
	Gene.126092::c28349_BT_Ma_ar_201_g1_i1::g.126092::m.126092	380.80	219.38	NO	
	Gene.61049::c10500_BT_Ma_ar_201_g1_i1::g.61049::m.61049	81.08	49.87	YES	
	Gene.21292::c6130_BT_Ma_ar_201_g1_i2::g.21292::m.21292	13.29	8.32	NO	
	Gene.71611::c11272_BT_Ma_ar_201_g1_i1::g.71611::m.71611	9.28	5.64	YES	
	Gene.42602::c8854_BT_Ma_ar_201_g1_i1::g.42602::m.42602	5.94	4.39	YES	
	Gene.6621::c2322_BT_Ma_ar_201_g1_i1::g.6621::m.6621	3.06	1.47	NO	
	Gene.34774::c7976_BT_Ma_ar_201_g1_i1::g.34774::m.34774	3.48	1.78	NO	
	Gene.10135::c3691_BT_Ma_ar_201_g1_i1::g.10135::m.10135	2.68	1.35	YES	
	Gene.1980::c719_BT_Ma_ar_201_g1_i2::g.1980::m.1980	1.57	0.49	NO	
	Gene.1978::c719_BT_Ma_ar_201_g1_i1::g.1978::m.1978	1.72	0.90	NO	
	Gene.112317::c14520_BT_Ma_ar_201_g1_i1::g.112317::m.112317	1.07	1.07	NO	
	Gene.158491::c59413_BT_Ma_ar_201_g1_i1::g.158491::m.158491	0.78	0.80	NO	
	Gene.123002::c24503_BT_Ma_ar_201_g1_i1::g.123002::m.123002	0.73	0.68	NO	
	Gene.114454::c16306_BT_Ma_ar_201_g1_i1::g.114454::m.114454	0.49	1.61	NO	
	Gene.156890::c57547_BT_Ma_ar_201_g1_i1::g.156890::m.156890	0.82	10.30	NO	
	Gene.125405::c27564_BT_Ma_ar_201_g1_i1::g.125405::m.125405	0.58	4.82	NO	
	Gene.141591::c42844_BT_Ma_ar_201_g1_i1::g.141591::m.141591	0.39	0.00	NO	
**Conoporin-like**	no hit	0	0	X	
**Csalpha/beta defensin**	Gene.126225::c28400_BT_Ma_ar_201_g1_i1::g.126225::m.126225	5810.80	13218.60	NO	
	Gene.18244::c5472_BT_Ma_ar_201_g1_i1::g.18244::m.18244	612.87	508.65	NO	
	Gene.13246::c4500_BT_Ma_ar_201_g1_i1::g.13246::m.13246	33.84	58.88	YES	
	Gene.38644::c8442_BT_Ma_ar_201_g1_i1::g.38644::m.38644	20.91	7.75	YES	
	Gene.38650::c8442_BT_Ma_ar_201_g1_i2::g.38650::m.38650	20.91	7.75	YES	
	Gene.8269::c2974_BT_Ma_ar_201_g1_i1::g.8269::m.8269	10.05	17.75	YES	
	Gene.112706::c14796_BT_Ma_ar_201_g1_i1::g.112706::m.112706	4.84	9.13	NO	
	Gene.4719::c1678_BT_Ma_ar_201_g1_i1::g.4719::m.4719	3.44	11.19	YES	
	Gene.146578::c47347_BT_Ma_ar_201_g1_i1::g.146578::m.146578	0.42	0.94	NO	
**Cystatin**	no hit	0	0	X	
**dICK's**	Gene.122719::c24236_BT_Ma_ar_201_g1_i1::g.122719::m.122719	73.93	68.95	NO	
	Gene.113514::c15452_BT_Ma_ar_201_g1_i1::g.113514::m.113514	1.58	1.03	NO	
**Disintegrine-like**	no hit	0	0	X	
**Echotoxin-like**	no hit	0	0	X	
**Gigantoxin-like**	no hit	0	0	X	

**Hyaluronidase**	Gene.13911::c4644_BT_Ma_ar_201_g1_i1::g.13911::m.13911	2.23	1.41	NO	
	Gene.1639::c579_BT_Ma_ar_201_g1_i1::g.1639::m.1639	1.62	3.29	YES	
**ICK's (Asilidin1)**	Gene.16040::c5071_BT_Ma_ar_201_g1_i1::g.16040::m.16040	2326.43	7803.10	YES	
	Gene.12953::c4441_BT_Ma_ar_201_g1_i1::g.12953::m.12953	479.35	1604.17	YES	
	Gene.128574::c29838_BT_Ma_ar_201_g1_i1::g.128574::m.128574	419.49	456.09	NO	
	Gene.2374::c871_BT_Ma_ar_201_g1_i1::g.2374::m.2374	170.09	194.77	YES	
	Gene.122719::c24236_BT_Ma_ar_201_g1_i1::g.122719::m.122719	73.93	68.95	NO	
	Gene.135785::c37503_BT_Ma_ar_201_g1_i1::g.135785::m.135785	125.60	134.72	NO	
	Gene.112576::c14699_BT_Ma_ar_201_g1_i1::g.112576::m.112576	20.09	71.40	NO	
	Gene.4789::c1710_BT_Ma_ar_201_g1_i1::g.4789::m.4789	12.65	25.53	YES	
	Gene.47439::c9321_BT_Ma_ar_201_g2_i1::g.47439::m.47439	6.26	8.22	NO	
	Gene.124527::c26203_BT_Ma_ar_201_g1_i1::g.124527::m.124527	6.14	8.75	NO	
	Gene.7265::c2557_BT_Ma_ar_201_g1_i1::g.7265::m.7265	3.24	3.87	NO	
	Gene.113514::c15452_BT_Ma_ar_201_g1_i1::g.113514::m.113514	1.58	1.03	NO	
	Gene.117389::c19695_BT_Ma_ar_201_g1_i1::g.117389::m.117389	0.69	0.47	NO	
	Gene.132650::c33743_BT_Ma_ar_201_g1_i1::g.132650::m.132650	0.68	1.99	NO	
**Kazal**	Gene.119253::c21798_BT_Ma_ar_201_g1_i1::g.119253::m.119253	962.17	2577.36	NO	
	Gene.45586::c9144_BT_Ma_ar_201_g1_i1::g.45586::m.45586	172.27	213.35	YES	
	Gene.60316::c10447_BT_Ma_ar_201_g1_i1::g.60316::m.60316	44.96	28.08	YES	excluded
	Gene.95966::c12490_BT_Ma_ar_201_g1_i1::g.95966::m.95966	17.14	5.35	NO	excluded
	Gene.95973::c12490_BT_Ma_ar_201_g1_i2::g.95973::m.95973	17.05	2.29	NO	excluded
	Gene.95981::c12490_BT_Ma_ar_201_g1_i3::g.95981::m.95981	17.05	2.29	NO	excluded
	Gene.110960::c13633_BT_Ma_ar_201_g1_i1::g.110960::m.110960	124.24	238.93	NO	
	Gene.77823::c11638_BT_Ma_ar_201_g1_i1::g.77823::m.77823	8.55	4.93	YES	excluded
	Gene.33349::c7815_BT_Ma_ar_201_g1_i1::g.33349::m.33349	8.67	4.76	NO	excluded
	Gene.33342::c7813_BT_Ma_ar_201_g1_i1::g.33342::m.33342	29.78	27.23	YES	
	Gene.9490::c3418_BT_Ma_ar_201_g1_i1::g.9490::m.9490	4.97	3.15	NO	excluded
	Gene.122601::c24133_BT_Ma_ar_201_g1_i1::g.122601::m.122601	15.34	34.33	NO	
	Gene.13259::c4505_BT_Ma_ar_201_g1_i1::g.13259::m.13259	21.20	49.84	NO	
	Gene.2123::c786_BT_Ma_ar_201_g1_i1::g.2123::m.2123	2.11	0.96	NO	excluded
	Gene.122644::c24170_BT_Ma_ar_201_g1_i1::g.122644::m.122644	2.44	1.61	NO	
	Gene.41579::c8751_BT_Ma_ar_201_g1_i1::g.41579::m.41579	5.52	3.02	NO	
	Gene.153697::c55024_BT_Ma_ar_201_g1_i1::g.153697::m.153697	1.87	1.39	NO	excluded
	Gene.146547::c47332_BT_Ma_ar_201_g1_i1::g.146547::m.146547	1.04	0.51	NO	
	Gene.5220::c1883_BT_Ma_ar_201_g1_i1::g.5220::m.5220	0.81	0.87	YES	
	Gene.141617::c42878_BT_Ma_ar_201_g1_i1::g.141617::m.141617	1.00	4.54	NO	
**Kazal (vasotab-like)**	Gene.13474::c4555_BT_Ma_ar_201_g1_i1::g.13474::m.13474	2766.76	1977.82	YES	
	Gene.41010::c8683_BT_Ma_ar_201_g1_i1::g.41010::m.41010	190.55	82.07	YES	excluded
	Gene.41028::c8683_BT_Ma_ar_201_g1_i2::g.41028::m.41028	184.44	17.79	YES	excluded
	Gene.53030::c9832_BT_Ma_ar_201_g1_i1::g.53030::m.53030	432.14	382.44	YES	
	Gene.13787::c4617_BT_Ma_ar_201_g1_i1::g.13787::m.13787	373.55	291.47	YES	
	Gene.29335::c7299_BT_Ma_ar_201_g1_i1::g.29335::m.29335	331.64	890.92	YES	
	Gene.128714::c29944_BT_Ma_ar_201_g1_i1::g.128714::m.128714	205.08	389.38	NO	
	Gene.28504::c7196_BT_Ma_ar_201_g1_i2::g.28504::m.28504	14.00	7.70	YES	
	Gene.67548::c10971_BT_Ma_ar_201_g3_i1::g.67548::m.67548	40.30	90.01	YES	
	Gene.41582::c8751_BT_Ma_ar_201_g1_i2::g.41582::m.41582	7.88	5.09	NO	
	Gene.28501::c7196_BT_Ma_ar_201_g1_i1::g.28501::m.28501	10.05	0.00	NO	
	Gene.155793::c56607_BT_Ma_ar_201_g1_i1::g.155793::m.155793	2.46	1.71	NO	
	Gene.143089::c45249_BT_Ma_ar_201_g1_i1::g.143089::m.143089	4.99	5.34	NO	
	Gene.149466::c50074_BT_Ma_ar_201_g1_i1::g.149466::m.149466	2.31	4.47	NO	
**Kunitz**	Gene.89151::c12217_BT_Ma_ar_201_g1_i1::g.89151::m.89151	126.42	71.77	YES	
	Gene.71522::c11261_BT_Ma_ar_201_g1_i1::g.71522::m.71522	35.56	17.76	NO	
	Gene.18759::c5598_BT_Ma_ar_201_g1_i1::g.18759::m.18759	168.97	297.21	YES	
	Gene.61876::c10561_BT_Ma_ar_201_g1_i1::g.61876::m.61876	5.51	1.56	YES	
	Gene.61901::c10561_BT_Ma_ar_201_g1_i2::g.61901::m.61901	5.51	1.56	YES	
	Gene.154614::c55630_BT_Ma_ar_201_g1_i1::g.154614::m.154614	87.47	95.37	NO	
	Gene.97366::c12536_BT_Ma_ar_201_g1_i1::g.97366::m.97366	4.76	1.90	NO	
	Gene.105517::c12789_BT_Ma_ar_201_g1_i1::g.105517::m.105517	19.42	1.24	NO	excluded
	Gene.97403::c12536_BT_Ma_ar_201_g1_i3::g.97403::m.97403	4.97	0.13	NO	
	Gene.97422::c12536_BT_Ma_ar_201_g1_i4::g.97422::m.97422	4.88	0.00	NO	
	Gene.97389::c12536_BT_Ma_ar_201_g1_i2::g.97389::m.97389	5.22	1.25	NO	excluded
	Gene.17651::c5368_BT_Ma_ar_201_g1_i1::g.17651::m.17651	57.04	76.71	NO	
	Gene.9688::c3499_BT_Ma_ar_201_g1_i1::g.9688::m.9688	12.15	14.25	YES	
	Gene.5434::c1981_BT_Ma_ar_201_g1_i1::g.5434::m.5434	5.76	10.68	YES	
	Gene.8747::c3175_BT_Ma_ar_201_g1_i1::g.8747::m.8747	4.48	6.59	YES	
	Gene.88336::c12186_BT_Ma_ar_201_g1_i1::g.88336::m.88336	2.11	3.87	YES	
	Gene.123331::c24834_BT_Ma_ar_201_g1_i1::g.123331::m.123331	0.97	1.04	NO	
	Gene.123332::c24834_BT_Ma_ar_201_g1_i1::g.123332::m.123332	1.00	0.68	NO	
	Gene.114104::c15956_BT_Ma_ar_201_g1_i1::g.114104::m.114104	1.08	5.12	NO	
**Lectin c-type**	Gene.36026::c8138_BT_Ma_ar_201_g1_i1::g.36026::m.36026	14.07	7.01	YES	excluded
	Gene.105760::c12795_BT_Ma_ar_201_g1_i4::g.105760::m.105760	5.76	0.23	NO	
	Gene.105734::c12795_BT_Ma_ar_201_g1_i2::g.105734::m.105734	5.67	1.53	NO	
	Gene.11911::c4239_BT_Ma_ar_201_g1_i1::g.11911::m.11911	46.50	29.17	YES	
	Gene.105749::c12795_BT_Ma_ar_201_g1_i3::g.105749::m.105749	6.60	0.58	NO	
	Gene.105775::c12795_BT_Ma_ar_201_g1_i5::g.105775::m.105775	6.90	0.42	NO	
	Gene.102428::c12697_BT_Ma_ar_201_g1_i1::g.102428::m.102428	14.33	8.13	NO	
	Gene.105726::c12795_BT_Ma_ar_201_g1_i1::g.105726::m.105726	7.66	1.23	NO	
	Gene.102444::c12697_BT_Ma_ar_201_g1_i2::g.102444::m.102444	9.46	2.25	NO	
	Gene.14425::c4754_BT_Ma_ar_201_g1_i1::g.14425::m.14425	2.98	2.65	YES	
	Gene.130433::c31420_BT_Ma_ar_201_g1_i1::g.130433::m.130433	3.25	2.93	NO	
	Gene.114463::c16318_BT_Ma_ar_201_g1_i1::g.114463::m.114463	1.46	0.80	NO	
	Gene.126170::c28379_BT_Ma_ar_201_g1_i1::g.126170::m.126170	1.63	1.05	NO	
	Gene.123368::c24872_BT_Ma_ar_201_g1_i1::g.123368::m.123368	1.05	0.47	NO	
	Gene.155118::c56036_BT_Ma_ar_201_g1_i1::g.155118::m.155118	0.84	0.00	NO	
**Lipocalin calycins**	Gene.53973::c9931_BT_Ma_ar_201_g1_i1::g.53973::m.53973	304.44	188.94	YES	
	Gene.6370::c2249_BT_Ma_ar_201_g1_i1::g.6370::m.6370	361.04	283.45	YES	
	Gene.84615::c11973_BT_Ma_ar_201_g1_i1::g.84615::m.84615	16.65	10.91	YES	

	Gene.120380::c22458_BT_Ma_ar_201_g1_i1::g.120380::m.120380	9.81	3.87	NO	
	Gene.120384::c22458_BT_Ma_ar_201_g1_i2::g.120384::m.120384	9.81	3.87	NO	
	Gene.7610::c2677_BT_Ma_ar_201_g1_i2::g.7610::m.7610	1.71	1.34	NO	
**Maxadilan-like**	no hit	0	0	X	
**MBF2-domain**	Gene.41350::c8726_BT_Ma_ar_201_g1_i1::g.41350::m.41350	2306.14	2338.62	YES	
	Gene.21832::c6201_BT_Ma_ar_201_g1_i1::g.21832::m.21832	501.79	544.99	YES	
	Gene.24349::c6571_BT_Ma_ar_201_g1_i1::g.24349::m.24349	170.09	197.61	YES	
	Gene.127315::c29022_BT_Ma_ar_201_g1_i1::g.127315::m.127315	105.96	97.85	NO	
	Gene.130793::c31762_BT_Ma_ar_201_g1_i1::g.130793::m.130793	85.75	84.30	NO	
	Gene.25112::c6680_BT_Ma_ar_201_g1_i1::g.25112::m.25112	47.81	58.77	YES	
	Gene.48115::c9376_BT_Ma_ar_201_g1_i1::g.48115::m.48115	30.77	13.06	YES	
	Gene.48118::c9376_BT_Ma_ar_201_g1_i2::g.48118::m.48118	30.77	13.06	YES	
	Gene.48121::c9376_BT_Ma_ar_201_g1_i3::g.48121::m.48121	30.77	13.06	YES	
	Gene.147264::c47889_BT_Ma_ar_201_g1_i1::g.147264::m.147264	4.39	6.30	NO	
**Melittin-like**	no hit	0	0	X	
**Natterin-like (Asilidin10)**	Gene.18175::c5457_BT_Ma_ar_201_g1_i1::g.18175::m.18175	392.69	263.15	NO	
	Gene.49004::c9464_BT_Ma_ar_201_g1_i1::g.49004::m.49004	92.48	63.43	NO	
	Gene.33996::c7899_BT_Ma_ar_201_g1_i1::g.33996::m.33996	29.50	20.63	NO	
	Gene.34984::c8003_BT_Ma_ar_201_g1_i1::g.34984::m.34984	23.65	6.76	NO	
	Gene.34989::c8003_BT_Ma_ar_201_g1_i2::g.34989::m.34989	29.56	19.48	NO	
	Gene.42290::c8827_BT_Ma_ar_201_g1_i1::g.42290::m.42290	7.33	4.12	NO	
**Neurokinin-like**	no hit	0	0	X	
**Nitrophorin-like**	no hit	0	0	X	
**Novel protein (Asilidin2)**	Gene.21554::c6170_BT_Ma_ar_201_g1_i1::g.21554::m.21554	1777.28	1329.58	YES	
	Gene.41502::c8742_BT_Ma_ar_201_g1_i1::g.41502::m.41502	271.73	84.57	YES	
	Gene.41503::c8742_BT_Ma_ar_201_g1_i2::g.41503::m.41503	271.73	84.57	YES	
	Gene.79913::c11744_BT_Ma_ar_201_g1_i1::g.79913::m.79913	92.44	53.75	YES	
	Gene.71032::c11227_BT_Ma_ar_201_g1_i1::g.71032::m.71032	74.50	48.46	YES	
	Gene.92596::c12356_BT_Ma_ar_201_g1_i2::g.92596::m.92596	23.20	11.52	NO	
	Gene.92590::c12356_BT_Ma_ar_201_g1_i1::g.92590::m.92590	16.91	7.60	YES	
	Gene.81282::c11818_BT_Ma_ar_201_g1_i1::g.81282::m.81282	9.71	7.44	YES	
	Gene.110503::c13360_BT_Ma_ar_201_g1_i1::g.110503::m.110503	4.41	2.31	NO	
	Gene.39630::c8538_BT_Ma_ar_201_g1_i1::g.39630::m.39630	5.11	2.59	NO	
	Gene.41214::c8703_BT_Ma_ar_201_g1_i1::g.41214::m.41214	3.56	2.02	YES	
	Gene.14287::c4723_BT_Ma_ar_201_g1_i1::g.14287::m.14287	3.60	2.09	NO	
	Gene.66422::c10881_BT_Ma_ar_201_g1_i2::g.66422::m.66422	3.71	1.78	NO	
	Gene.121292::c23081_BT_Ma_ar_201_g1_i1::g.121292::m.121292	1.70	1.12	NO	
	Gene.66419::c10881_BT_Ma_ar_201_g1_i1::g.66419::m.66419	1.79	2.09	NO	
	Gene.145216::c46377_BT_Ma_ar_201_g1_i1::g.145216::m.145216	1.33	1.44	NO	
	Gene.81288::c11818_BT_Ma_ar_201_g2_i1::g.81288::m.81288	2.04	3.19	NO	
**Novel protein (Asilidin3)**	Gene.2992::c1157_BT_Ma_ar_201_g1_i2::g.2992::m.2992	158.34	139.39	YES	
	Gene.2991::c1157_BT_Ma_ar_201_g1_i1::g.2991::m.2991	127.21	19.23	YES	
	Gene.27157::c7006_BT_Ma_ar_201_g1_i1::g.27157::m.27157	9.43	13.51	YES	
**Novel protein (Asilidin4)**	Gene.114720::c16546_BT_Ma_ar_201_g1_i1::g.114720::m.114720	2.58	3.47	NO	
**Novel protein (Asilidin5)**	Gene.47615::c9335_BT_Ma_ar_201_g1_i1::g.47615::m.47615	48.75	31.18	YES	
	Gene.131739::c32726_BT_Ma_ar_201_g1_i1::g.131739::m.131739	1.70	1.75	NO	
**Novel protein (Asilidin6)**	no hit	0	0	X	
**Novel protein (Asilidin7)**	no hit	0	0	X	
**Peptidase M12**	Gene.153229::c54758_BT_Ma_ar_201_g1_i1::g.153229::m.153229	307.34	202.47	NO	
	Gene.8639::c3136_BT_Ma_ar_201_g1_i1::g.8639::m.8639	109.69	75.56	NO	
	Gene.44509::c9040_BT_Ma_ar_201_g1_i1::g.44509::m.44509	13.58	5.80	NO	
	Gene.44521::c9040_BT_Ma_ar_201_g1_i2::g.44521::m.44521	15.00	2.57	NO	
	Gene.106137::c12808_BT_Ma_ar_201_g1_i1::g.106137::m.106137	8.14	3.07	NO	
	Gene.31561::c7589_BT_Ma_ar_201_g1_i2::g.31561::m.31561	9.49	4.60	YES	
	Gene.31579::c7589_BT_Ma_ar_201_g1_i3::g.31579::m.31579	9.53	0.84	YES	
	Gene.106154::c12808_BT_Ma_ar_201_g1_i2::g.106154::m.106154	8.23	2.09	NO	
	Gene.70649::c11202_BT_Ma_ar_201_g1_i1::g.70649::m.70649	7.55	4.62	YES	
	Gene.64398::c10735_BT_Ma_ar_201_g1_i1::g.64398::m.64398	8.04	4.14	NO	
	Gene.53853::c9912_BT_Ma_ar_201_g1_i3::g.53853::m.53853	23.84	14.15	NO	
	Gene.46302::c9209_BT_Ma_ar_201_g1_i2::g.46302::m.46302	7.19	2.01	YES	
	Gene.15108::c4897_BT_Ma_ar_201_g1_i1::g.15108::m.15108	13.32	8.95	NO	
	Gene.46290::c9209_BT_Ma_ar_201_g1_i1::g.46290::m.46290	7.03	2.39	YES	
	Gene.46291::c9209_BT_Ma_ar_201_g1_i1::g.46291::m.46291	6.95	0.32	NO	
	Gene.64399::c10735_BT_Ma_ar_201_g1_i1::g.64399::m.64399	4.04	2.87	YES	
	Gene.155335::c56229_BT_Ma_ar_201_g1_i1::g.155335::m.155335	2.06	1.15	NO	
	Gene.16454::c5136_BT_Ma_ar_201_g1_i1::g.16454::m.16454	4.83	4.18	NO	
	Gene.1768::c631_BT_Ma_ar_201_g1_i1::g.1768::m.1768	1.92	1.75	NO	
	Gene.1775::c631_BT_Ma_ar_201_g1_i2::g.1775::m.1775	1.89	0.00	NO	
	Gene.146852::c47565_BT_Ma_ar_201_g1_i1::g.146852::m.146852	0.84	0.66	NO	
	Gene.148272::c48824_BT_Ma_ar_201_g1_i1::g.148272::m.148272	0.64	1.53	NO	
	Gene.154913::c55875_BT_Ma_ar_201_g1_i1::g.154913::m.154913	0.33	3.43	NO	
**Peptidase M13**	Gene.54034::c9940_BT_Ma_ar_201_g1_i1::g.54034::m.54034	587.73	295.55	YES	
	Gene.18379::c5498_BT_Ma_ar_201_g2_i1::g.18379::m.18379	310.52	181.55	YES	

	Gene.84024::c11945_BT_Ma_ar_201_g1_i1::g.84024::m.84024	293.26	177.35	YES	
	Gene.68065::c11012_BT_Ma_ar_201_g1_i1::g.68065::m.68065	225.99	124.31	YES	
	Gene.4012::c1436_BT_Ma_ar_201_g1_i1::g.4012::m.4012	117.60	65.20	YES	
	Gene.69748::c11133_BT_Ma_ar_201_g1_i1::g.69748::m.69748	126.00	76.12	NO	
	Gene.38384::c8413_BT_Ma_ar_201_g1_i1::g.38384::m.38384	94.76	52.19	YES	
	Gene.76312::c11556_BT_Ma_ar_201_g1_i1::g.76312::m.76312	60.44	34.94	YES	
	Gene.38638::c8440_BT_Ma_ar_201_g1_i1::g.38638::m.38638	60.74	35.92	YES	
	Gene.48796::c9439_BT_Ma_ar_201_g1_i1::g.48796::m.48796	48.22	27.95	NO	
	Gene.77963::c11648_BT_Ma_ar_201_g1_i1::g.77963::m.77963	51.97	32.82	YES	
	Gene.75638::c11520_BT_Ma_ar_201_g1_i3::g.75638::m.75638	35.81	0.00	NO	
	Gene.75626::c11520_BT_Ma_ar_201_g1_i2::g.75626::m.75626	36.58	16.27	NO	
	Gene.96967::c12522_BT_Ma_ar_201_g1_i1::g.96967::m.96967	39.80	21.58	NO	
	Gene.75650::c11520_BT_Ma_ar_201_g1_i4::g.75650::m.75650	34.93	3.96	NO	
	Gene.107618::c12854_BT_Ma_ar_201_g1_i1::g.107618::m.107618	39.30	21.72	NO	
	Gene.69969::c11150_BT_Ma_ar_201_g1_i1::g.69969::m.69969	24.04	13.71	YES	
	Gene.84905::c11991_BT_Ma_ar_201_g1_i1::g.84905::m.84905	27.44	17.16	NO	
	Gene.91320::c12307_BT_Ma_ar_201_g2_i1::g.91320::m.91320	15.14	4.62	YES	
	Gene.91326::c12307_BT_Ma_ar_201_g2_i2::g.91326::m.91326	15.14	4.62	YES	
	Gene.107619::c12854_BT_Ma_ar_201_g1_i1::g.107619::m.107619	12.46	7.08	NO	
	Gene.22833::c6363_BT_Ma_ar_201_g1_i1::g.22833::m.22833	20.93	12.06	NO	
	Gene.10945::c4030_BT_Ma_ar_201_g1_i1::g.10945::m.10945	9.99	5.83	YES	
	Gene.53784::c9906_BT_Ma_ar_201_g1_i1::g.53784::m.53784	6.66	3.86	YES	
	Gene.14458::c4765_BT_Ma_ar_201_g1_i1::g.14458::m.14458	6.63	4.19	YES	
	Gene.22834::c6363_BT_Ma_ar_201_g1_i1::g.22834::m.22834	18.75	12.35	YES	
	Gene.2324::c850_BT_Ma_ar_201_g1_i1::g.2324::m.2324	6.85	4.11	YES	
	Gene.112510::c14665_BT_Ma_ar_201_g1_i1::g.112510::m.112510	4.41	2.46	NO	
	Gene.65479::c10809_BT_Ma_ar_201_g1_i1::g.65479::m.65479	5.81	3.28	NO	
	Gene.24526::c6585_BT_Ma_ar_201_g1_i1::g.24526::m.24526	11.89	8.60	NO	
	Gene.24525::c6585_BT_Ma_ar_201_g1_i1::g.24525::m.24525	8.22	5.70	NO	
	Gene.14191::c4700_BT_Ma_ar_201_g1_i1::g.14191::m.14191	4.17	2.19	YES	
	Gene.1592::c564_BT_Ma_ar_201_g1_i1::g.1592::m.1592	3.64	2.65	NO	
	Gene.154853::c55826_BT_Ma_ar_201_g1_i1::g.154853::m.154853	4.61	2.59	NO	
	Gene.135048::c37085_BT_Ma_ar_201_g1_i1::g.135048::m.135048	3.38	2.22	NO	
	Gene.56192::c10119_BT_Ma_ar_201_g1_i1::g.56192::m.56192	3.64	2.31	NO	
	Gene.9414::c3380_BT_Ma_ar_201_g1_i1::g.9414::m.9414	2.56	1.45	NO	
	Gene.2444::c897_BT_Ma_ar_201_g1_i1::g.2444::m.2444	4.08	1.53	NO	
	Gene.29329::c7298_BT_Ma_ar_201_g1_i1::g.29329::m.29329	2.62	1.36	NO	
	Gene.144185::c45755_BT_Ma_ar_201_g1_i1::g.144185::m.144185	2.06	1.52	NO	
	Gene.7226::c2546_BT_Ma_ar_201_g1_i1::g.7226::m.7226	3.38	2.36	NO	
	Gene.143100::c45255_BT_Ma_ar_201_g1_i1::g.143100::m.143100	2.32	1.43	NO	
	Gene.110408::c13303_BT_Ma_ar_201_g1_i1::g.110408::m.110408	2.75	2.32	NO	
	Gene.13848::c4631_BT_Ma_ar_201_g1_i1::g.13848::m.13848	2.04	1.36	NO	
	Gene.6448::c2266_BT_Ma_ar_201_g1_i1::g.6448::m.6448	1.57	1.26	YES	
	Gene.5627::c2055_BT_Ma_ar_201_g1_i1::g.5627::m.5627	3.24	3.07	NO	
	Gene.110544::c13380_BT_Ma_ar_201_g1_i1::g.110544::m.110544	1.67	1.25	NO	
	Gene.136003::c37619_BT_Ma_ar_201_g1_i1::g.136003::m.136003	1.52	1.18	NO	
	Gene.137406::c38594_BT_Ma_ar_201_g1_i1::g.137406::m.137406	1.93	1.66	NO	
	Gene.5500::c2015_BT_Ma_ar_201_g1_i1::g.5500::m.5500	2.16	3.08	YES	
	Gene.111073::c13710_BT_Ma_ar_201_g1_i1::g.111073::m.111073	1.75	2.80	NO	
	Gene.127269::c28992_BT_Ma_ar_201_g1_i1::g.127269::m.127269	0.89	1.31	NO	
	Gene.144195::c45762_BT_Ma_ar_201_g1_i1::g.144195::m.144195	1.22	1.60	NO	
	Gene.156401::c57085_BT_Ma_ar_201_g1_i1::g.156401::m.156401	1.11	0.55	NO	
	Gene.130751::c31715_BT_Ma_ar_201_g1_i1::g.130751::m.130751	1.30	1.64	NO	
	Gene.122676::c24190_BT_Ma_ar_201_g1_i1::g.122676::m.122676	0.95	1.49	NO	
	Gene.111858::c14226_BT_Ma_ar_201_g1_i1::g.111858::m.111858	0.94	3.50	NO	
	Gene.114764::c16592_BT_Ma_ar_201_g1_i1::g.114764::m.114764	0.66	1.29	NO	
	Gene.116757::c18896_BT_Ma_ar_201_g1_i1::g.116757::m.116757	0.68	0.90	NO	
	Gene.123642::c25177_BT_Ma_ar_201_g1_i1::g.123642::m.123642	0.97	2.05	NO	
	Gene.138033::c39058_BT_Ma_ar_201_g1_i1::g.138033::m.138033	0.73	1.04	NO	
	Gene.115531::c17414_BT_Ma_ar_201_g1_i1::g.115531::m.115531	0.85	2.95	NO	
	Gene.128437::c29764_BT_Ma_ar_201_g1_i1::g.128437::m.128437	0.60	0.00	NO	
	Gene.10426::c3807_BT_Ma_ar_201_g1_i1::g.10426::m.10426	1.15	0.00	NO	
	Gene.139184::c40079_BT_Ma_ar_201_g1_i1::g.139184::m.139184	0.68	0.00	NO	
	Gene.150379::c51166_BT_Ma_ar_201_g1_i1::g.150379::m.150379	0.50	1.58	NO	
	Gene.122052::c23656_BT_Ma_ar_201_g1_i1::g.122052::m.122052	0.47	5.94	NO	
**Peptidase M14**	Gene.25661::c6761_BT_Ma_ar_201_g1_i1::g.25661::m.25661	1834.10	1066.49	YES	
	Gene.58257::c10278_BT_Ma_ar_201_g2_i2::g.58257::m.58257	44.59	21.90	YES	
	Gene.78424::c11673_BT_Ma_ar_201_g1_i1::g.78424::m.78424	143.20	85.26	YES	
	Gene.47589::c9333_BT_Ma_ar_201_g1_i1::g.47589::m.47589	49.18	29.30	YES	
	Gene.58251::c10278_BT_Ma_ar_201_g2_i1::g.58251::m.58251	49.08	15.70	YES	
	Gene.118692::c21531_BT_Ma_ar_201_g1_i1::g.118692::m.118692	62.07	37.27	NO	
	Gene.82558::c11878_BT_Ma_ar_201_g1_i1::g.82558::m.82558	47.35	30.60	YES	
	Gene.34707::c7971_BT_Ma_ar_201_g1_i1::g.34707::m.34707	34.28	20.35	YES	
	Gene.34712::c7971_BT_Ma_ar_201_g1_i2::g.34712::m.34712	34.19	0.00	YES	
	Gene.1034::c381_BT_Ma_ar_201_g1_i1::g.1034::m.1034	35.00	21.44	YES	
	Gene.75785::c11528_BT_Ma_ar_201_g1_i1::g.75785::m.75785	26.71	16.79	YES	
	Gene.66298::c10874_BT_Ma_ar_201_g2_i1::g.66298::m.66298	19.40	11.66	YES	
	Gene.66303::c10874_BT_Ma_ar_201_g2_i2::g.66303::m.66303	18.29	1.69	YES	
	Gene.123504::c25045_BT_Ma_ar_201_g1_i1::g.123504::m.123504	0.84	0.67	NO	
	Gene.135067::c37093_BT_Ma_ar_201_g1_i1::g.135067::m.135067	0.83	0.90	NO	
	Gene.150434::c51228_BT_Ma_ar_201_g1_i1::g.150434::m.150434	0.50	1.22	NO	
	Gene.158112::c58889_BT_Ma_ar_201_g1_i1::g.158112::m.158112	0.99	1.50	NO	
**Peptidase S10**	Gene.50758::c9627_BT_Ma_ar_201_g1_i1::g.50758::m.50758	149.69	88.26	YES	
	Gene.21138::c6101_BT_Ma_ar_201_g1_i1::g.21138::m.21138	61.47	34.22	YES	
	Gene.36746::c8222_BT_Ma_ar_201_g1_i1::g.36746::m.36746	15.48	6.97	NO	
	Gene.133717::c35213_BT_Ma_ar_201_g1_i1::g.133717::m.133717	0.57	0.00	NO	
**Peptidase S1**	Gene.152462::c54419_BT_Ma_ar_201_g1_i1::g.152462::m.152462	7914.57	4919.74	NO	
	Gene.152306::c54353_BT_Ma_ar_201_g1_i1::g.152306::m.152306	7288.54	4670.59	NO	
	Gene.11221::c4095_BT_Ma_ar_201_g1_i1::g.11221::m.11221	5323.59	3413.49	NO	
	Gene.125784::c28228_BT_Ma_ar_201_g1_i1::g.125784::m.125784	3796.84	2422.30	NO	
	Gene.31723::c7605_BT_Ma_ar_201_g1_i1::g.31723::m.31723	3463.24	2189.28	YES	
	Gene.152645::c54485_BT_Ma_ar_201_g1_i1::g.152645::m.152645	3005.74	1920.04	NO	
	Gene.71704::c11277_BT_Ma_ar_201_g1_i1::g.71704::m.71704	2275.17	1760.78	NO	
	Gene.119215::c21790_BT_Ma_ar_201_g1_i1::g.119215::m.119215	1861.46	1202.55	NO	
	Gene.90915::c12282_BT_Ma_ar_201_g2_i1::g.90915::m.90915	1767.71	1150.41	NO	

	Gene.39674::c8547_BT_Ma_ar_201_g1_i1::g.39674::m.39674	1099.47	703.93	YES	
	Gene.32758::c7751_BT_Ma_ar_201_g1_i1::g.32758::m.32758	979.63	585.07	YES	
	Gene.143661::c45514_BT_Ma_ar_201_g1_i1::g.143661::m.143661	932.57	607.21	NO	
	Gene.107740::c12857_BT_Ma_ar_201_g1_i1::g.107740::m.107740	127.88	66.49	NO	excluded
	Gene.11283::c4108_BT_Ma_ar_201_g1_i1::g.11283::m.11283	836.56	532.58	YES	
	Gene.109593::c12932_BT_Ma_ar_201_g1_i1::g.109593::m.109593	422.51	235.34	NO	
	Gene.34883::c7986_BT_Ma_ar_201_g1_i1::g.34883::m.34883	653.79	424.66	NO	
	Gene.57001::c10198_BT_Ma_ar_201_g1_i1::g.57001::m.57001	645.66	401.09	NO	
	Gene.32764::c7751_BT_Ma_ar_201_g2_i1::g.32764::m.32764	622.33	411.32	YES	
	Gene.24084::c6538_BT_Ma_ar_201_g1_i1::g.24084::m.24084	586.42	388.29	YES	
	Gene.120282::c22396_BT_Ma_ar_201_g1_i1::g.120282::m.120282	516.80	316.22	NO	
	Gene.62395::c10598_BT_Ma_ar_201_g1_i1::g.62395::m.62395	452.92	293.14	YES	
	Gene.24216::c6555_BT_Ma_ar_201_g1_i1::g.24216::m.24216	440.47	266.85	NO	
	Gene.154170::c55325_BT_Ma_ar_201_g1_i1::g.154170::m.154170	458.66	295.05	NO	
	Gene.152920::c54605_BT_Ma_ar_201_g1_i1::g.152920::m.152920	440.03	275.24	NO	
	Gene.90911::c12282_BT_Ma_ar_201_g1_i1::g.90911::m.90911	1646.33	2104.14	NO	
	Gene.22661::c6331_BT_Ma_ar_201_g1_i1::g.22661::m.22661	448.50	278.68	YES	
	Gene.32624::c7725_BT_Ma_ar_201_g1_i1::g.32624::m.32624	386.97	245.06	YES	
	Gene.90917::c12282_BT_Ma_ar_201_g2_i2::g.90917::m.90917	690.44	273.26	NO	
	Gene.110168::c13179_BT_Ma_ar_201_g1_i1::g.110168::m.110168	357.09	222.77	NO	
	Gene.32461::c7707_BT_Ma_ar_201_g1_i1::g.32461::m.32461	345.03	195.39	YES	
	Gene.31486::c7576_BT_Ma_ar_201_g1_i1::g.31486::m.31486	315.57	197.40	NO	
	Gene.100235::c12629_BT_Ma_ar_201_g1_i2::g.100235::m.100235	65.31	30.14	NO	excluded
	Gene.98967::c12593_BT_Ma_ar_201_g3_i2::g.98967::m.98967	250.36	91.44	YES	
	Gene.98972::c12593_BT_Ma_ar_201_g3_i3::g.98972::m.98972	248.51	45.06	YES	
	Gene.42848::c8878_BT_Ma_ar_201_g1_i1::g.42848::m.42848	278.77	181.87	YES	
	Gene.32466::c7707_BT_Ma_ar_201_g1_i2::g.32466::m.32466	303.43	183.35	NO	
	Gene.63340::c10666_BT_Ma_ar_201_g1_i1::g.63340::m.63340	136.43	76.90	YES	
	Gene.135078::c37100_BT_Ma_ar_201_g1_i1::g.135078::m.135078	213.91	136.85	NO	
	Gene.144221::c45781_BT_Ma_ar_201_g1_i1::g.144221::m.144221	214.97	142.00	NO	
	Gene.3751::c1373_BT_Ma_ar_201_g1_i1::g.3751::m.3751	207.76	124.82	YES	
	Gene.11749::c4200_BT_Ma_ar_201_g1_i1::g.11749::m.11749	197.10	125.77	YES	
	Gene.73210::c11375_BT_Ma_ar_201_g1_i1::g.73210::m.73210	226.56	151.68	NO	
	Gene.135671::c37431_BT_Ma_ar_201_g1_i1::g.135671::m.135671	160.14	97.57	NO	
	Gene.52859::c9814_BT_Ma_ar_201_g1_i1::g.52859::m.52859	114.71	75.03	YES	
	Gene.60585::c10460_BT_Ma_ar_201_g1_i2::g.60585::m.60585	98.57	35.68	YES	
	Gene.60579::c10460_BT_Ma_ar_201_g1_i1::g.60579::m.60579	99.04	31.46	YES	
	Gene.839::c291_BT_Ma_ar_201_g1_i1::g.839::m.839	135.83	81.71	YES	
	Gene.51593::c9700_BT_Ma_ar_201_g1_i1::g.51593::m.51593	142.70	101.10	NO	
	Gene.16588::c5162_BT_Ma_ar_201_g1_i1::g.16588::m.16588	133.64	80.55	YES	
	Gene.71603::c11271_BT_Ma_ar_201_g1_i1::g.71603::m.71603	124.90	27.58	YES	
	Gene.71606::c11271_BT_Ma_ar_201_g1_i2::g.71606::m.71606	124.90	27.58	YES	
	Gene.71609::c11271_BT_Ma_ar_201_g1_i3::g.71609::m.71609	124.90	27.58	YES	
	Gene.100232::c12629_BT_Ma_ar_201_g1_i1::g.100232::m.100232	88.76	26.36	NO	
	Gene.98983::c12593_BT_Ma_ar_201_g3_i5::g.98983::m.98983	204.47	28.04	NO	
	Gene.126477::c28512_BT_Ma_ar_201_g1_i1::g.126477::m.126477	112.71	74.24	NO	
	Gene.73215::c11375_BT_Ma_ar_201_g2_i1::g.73215::m.73215	194.91	140.27	YES	
	Gene.28195::c7164_BT_Ma_ar_201_g1_i1::g.28195::m.28195	77.33	46.87	YES	
	Gene.23631::c6475_BT_Ma_ar_201_g1_i1::g.23631::m.23631	118.61	79.02	YES	
	Gene.36876::c8237_BT_Ma_ar_201_g1_i1::g.36876::m.36876	106.65	68.07	YES	
	Gene.98960::c12593_BT_Ma_ar_201_g3_i1::g.98960::m.98960	203.47	0.00	NO	
	Gene.40317::c8605_BT_Ma_ar_201_g1_i1::g.40317::m.40317	84.68	58.03	YES	
	Gene.53855::c9913_BT_Ma_ar_201_g1_i1::g.53855::m.53855	86.22	64.18	YES	
	Gene.50386::c9593_BT_Ma_ar_201_g1_i1::g.50386::m.50386	54.30	35.69	YES	
	Gene.37245::c8282_BT_Ma_ar_201_g1_i1::g.37245::m.37245	74.59	50.15	YES	
	Gene.53109::c9842_BT_Ma_ar_201_g1_i1::g.53109::m.53109	53.60	34.36	YES	
	Gene.56263::c10129_BT_Ma_ar_201_g1_i1::g.56263::m.56263	41.79	12.38	YES	
	Gene.56272::c10129_BT_Ma_ar_201_g1_i2::g.56272::m.56272	41.79	12.38	YES	
	Gene.57105::c10204_BT_Ma_ar_201_g1_i3::g.57105::m.57105	71.78	39.65	YES	
	Gene.52477::c9783_BT_Ma_ar_201_g1_i1::g.52477::m.52477	69.91	42.21	YES	
	Gene.3446::c1298_BT_Ma_ar_201_g1_i1::g.3446::m.3446	69.62	45.86	YES	
	Gene.3977::c1433_BT_Ma_ar_201_g1_i1::g.3977::m.3977	58.81	42.43	YES	
	Gene.98984::c12593_BT_Ma_ar_201_g3_i5::g.98984::m.98984	170.59	7.08	NO	
	Gene.31874::c7626_BT_Ma_ar_201_g1_i1::g.31874::m.31874	59.93	39.49	YES	
	Gene.26444::c6870_BT_Ma_ar_201_g1_i1::g.26444::m.26444	42.46	25.78	YES	
	Gene.37853::c8362_BT_Ma_ar_201_g1_i1::g.37853::m.37853	48.90	30.53	NO	
	Gene.127579::c29170_BT_Ma_ar_201_g1_i1::g.127579::m.127579	51.79	34.71	NO	
	Gene.119812::c22097_BT_Ma_ar_201_g1_i1::g.119812::m.119812	52.88	32.65	NO	
	Gene.154767::c55759_BT_Ma_ar_201_g1_i1::g.154767::m.154767	84.91	55.95	NO	
	Gene.62906::c10639_BT_Ma_ar_201_g1_i1::g.62906::m.62906	43.44	28.54	YES	
	Gene.18111::c5439_BT_Ma_ar_201_g1_i1::g.18111::m.18111	56.60	39.63	YES	
	Gene.22820::c6362_BT_Ma_ar_201_g1_i1::g.22820::m.22820	42.35	23.57	YES	
	Gene.31487::c7576_BT_Ma_ar_201_g1_i1::g.31487::m.31487	44.68	28.62	YES	
	Gene.14130::c4687_BT_Ma_ar_201_g1_i1::g.14130::m.14130	43.10	31.05	YES	
	Gene.1841::c657_BT_Ma_ar_201_g1_i1::g.1841::m.1841	37.51	26.07	YES	
	Gene.69260::c11105_BT_Ma_ar_201_g1_i1::g.69260::m.69260	24.55	12.93	YES	
	Gene.80775::c11795_BT_Ma_ar_201_g1_i1::g.80775::m.80775	23.06	15.26	YES	
	Gene.57099::c10204_BT_Ma_ar_201_g1_i1::g.57099::m.57099	67.73	9.54	YES	
	Gene.120468::c22509_BT_Ma_ar_201_g1_i1::g.120468::m.120468	88.25	76.91	NO	
	Gene.113790::c15687_BT_Ma_ar_201_g1_i1::g.113790::m.113790	40.54	26.75	NO	
	Gene.98961::c12593_BT_Ma_ar_201_g3_i1::g.98961::m.98961	105.22	18.58	NO	
	Gene.10067::c3661_BT_Ma_ar_201_g1_i1::g.10067::m.10067	31.66	23.26	YES	
	Gene.34339::c7931_BT_Ma_ar_201_g1_i1::g.34339::m.34339	34.28	24.74	YES	
	Gene.119484::c21924_BT_Ma_ar_201_g1_i1::g.119484::m.119484	34.26	21.30	NO	
	Gene.3487::c1316_BT_Ma_ar_201_g1_i1::g.3487::m.3487	22.25	14.87	YES	
	Gene.110218::c13209_BT_Ma_ar_201_g1_i1::g.110218::m.110218	30.69	20.09	NO	
	Gene.119984::c22193_BT_Ma_ar_201_g1_i1::g.119984::m.119984	31.17	20.61	NO	
	Gene.24620::c6602_BT_Ma_ar_201_g1_i1::g.24620::m.24620	23.93	13.32	YES	
	Gene.145092::c46298_BT_Ma_ar_201_g1_i1::g.145092::m.145092	19.88	13.36	NO	
	Gene.18588::c5543_BT_Ma_ar_201_g1_i1::g.18588::m.18588	29.13	17.59	YES	
	Gene.26977::c6964_BT_Ma_ar_201_g1_i1::g.26977::m.26977	25.39	17.41	YES	
	Gene.44577::c9043_BT_Ma_ar_201_g1_i1::g.44577::m.44577	19.95	13.44	YES	
	Gene.22534::c6307_BT_Ma_ar_201_g1_i1::g.22534::m.22534	26.69	14.44	YES	
	Gene.60760::c10476_BT_Ma_ar_201_g1_i2::g.60760::m.60760	26.53	16.56	YES	
	Gene.72221::c11310_BT_Ma_ar_201_g1_i1::g.72221::m.72221	9.65	5.60	YES	
	Gene.57100::c10204_BT_Ma_ar_201_g1_i1::g.57100::m.57100	55.60	5.13	NO	
	Gene.57102::c10204_BT_Ma_ar_201_g1_i2::g.57102::m.57102	55.60	5.13	NO	
	Gene.26431::c6867_BT_Ma_ar_201_g1_i1::g.26431::m.26431	24.33	14.34	YES	
	Gene.143693::c45529_BT_Ma_ar_201_g1_i1::g.143693::m.143693	23.55	15.89	NO	
	Gene.126865::c28724_BT_Ma_ar_201_g1_i1::g.126865::m.126865	10.02	5.02	NO	
	Gene.119366::c21861_BT_Ma_ar_201_g1_i1::g.119366::m.119366	19.85	13.96	NO	

	Gene.35942::c8128_BT_Ma_ar_201_g1_i1::g.35942::m.35942	5.13	2.58	NO	excluded
	Gene.154508::c55563_BT_Ma_ar_201_g1_i1::g.154508::m.154508	32.74	18.10	NO	
	Gene.8750::c3176_BT_Ma_ar_201_g1_i1::g.8750::m.8750	38.55	24.34	NO	
	Gene.57103::c10204_BT_Ma_ar_201_g1_i2::g.57103::m.57103	56.59	10.98	YES	
	Gene.17231::c5286_BT_Ma_ar_201_g1_i2::g.17231::m.17231	17.08	9.42	NO	excluded
	Gene.67817::c10991_BT_Ma_ar_201_g1_i1::g.67817::m.67817	17.89	11.03	YES	
	Gene.23270::c6433_BT_Ma_ar_201_g1_i1::g.23270::m.23270	17.61	11.80	YES	
	Gene.51831::c9714_BT_Ma_ar_201_g1_i1::g.51831::m.51831	14.66	7.39	YES	
	Gene.67913::c10996_BT_Ma_ar_201_g1_i1::g.67913::m.67913	10.93	7.49	YES	
	Gene.41835::c8781_BT_Ma_ar_201_g1_i1::g.41835::m.41835	15.81	11.50	YES	
	Gene.17227::c5286_BT_Ma_ar_201_g1_i1::g.17227::m.17227	16.65	2.36	NO	excluded
	Gene.62273::c10584_BT_Ma_ar_201_g1_i3::g.62273::m.62273	11.89	2.23	YES	
	Gene.62263::c10584_BT_Ma_ar_201_g1_i2::g.62263::m.62263	11.49	3.86	NO	
	Gene.62251::c10584_BT_Ma_ar_201_g1_i1::g.62251::m.62251	14.13	3.86	NO	
	Gene.77369::c11612_BT_Ma_ar_201_g1_i1::g.77369::m.77369	10.39	7.05	YES	
	Gene.56829::c10183_BT_Ma_ar_201_g1_i1::g.56829::m.56829	10.76	6.99	YES	
	Gene.13155::c4477_BT_Ma_ar_201_g1_i1::g.13155::m.13155	11.29	8.04	YES	
	Gene.18708::c5579_BT_Ma_ar_201_g1_i1::g.18708::m.18708	33.05	34.11	NO	
	Gene.67820::c10991_BT_Ma_ar_201_g2_i1::g.67820::m.67820	16.81	14.70	YES	
	Gene.55821::c10084_BT_Ma_ar_201_g1_i2::g.55821::m.55821	5.38	2.62	YES	
	Gene.98958::c12593_BT_Ma_ar_201_g2_i1::g.98958::m.98958	18.15	8.76	NO	
	Gene.673::c216_BT_Ma_ar_201_g1_i2::g.673::m.673	5.97	3.50	NO	
	Gene.16248::c5102_BT_Ma_ar_201_g1_i1::g.16248::m.16248	9.16	5.39	NO	
	Gene.39616::c8536_BT_Ma_ar_201_g1_i2::g.39616::m.39616	5.71	3.98	YES	
	Gene.54307::c9964_BT_Ma_ar_201_g1_i1::g.54307::m.54307	8.14	5.64	NO	
	Gene.55810::c10084_BT_Ma_ar_201_g1_i1::g.55810::m.55810	4.65	1.27	YES	
	Gene.29593::c7338_BT_Ma_ar_201_g1_i1::g.29593::m.29593	6.77	5.28	YES	
	Gene.128566::c29834_BT_Ma_ar_201_g1_i1::g.128566::m.128566	7.22	5.47	NO	
	Gene.55813::c10084_BT_Ma_ar_201_g2_i1::g.55813::m.55813	3.71	2.10	YES	
	Gene.51834::c9714_BT_Ma_ar_201_g1_i2::g.51834::m.51834	13.98	6.16	YES	
	Gene.22453::c6291_BT_Ma_ar_201_g1_i1::g.22453::m.22453	4.47	3.09	YES	
	Gene.3295::c1255_BT_Ma_ar_201_g1_i2::g.3295::m.3295	4.76	3.28	NO	
	Gene.12182::c4294_BT_Ma_ar_201_g1_i1::g.12182::m.12182	4.10	2.30	YES	
	Gene.18709::c5579_BT_Ma_ar_201_g1_i2::g.18709::m.18709	28.81	0.00	NO	
	Gene.26194::c6824_BT_Ma_ar_201_g1_i1::g.26194::m.26194	4.51	1.34	NO	
	Gene.18124::c5442_BT_Ma_ar_201_g1_i1::g.18124::m.18124	3.97	2.71	YES	
	Gene.119365::c21858_BT_Ma_ar_201_g1_i1::g.119365::m.119365	24.81	30.19	NO	
	Gene.55825::c10084_BT_Ma_ar_201_g3_i1::g.55825::m.55825	3.08	1.70	YES	
	Gene.12102::c4277_BT_Ma_ar_201_g1_i2::g.12102::m.12102	4.52	2.58	NO	
	Gene.119306::c21826_BT_Ma_ar_201_g1_i1::g.119306::m.119306	4.84	4.14	NO	
	Gene.51835::c9714_BT_Ma_ar_201_g1_i2::g.51835::m.51835	10.28	1.64	NO	
	Gene.6445::c2265_BT_Ma_ar_201_g1_i1::g.6445::m.6445	4.06	2.42	YES	
	Gene.12096::c4277_BT_Ma_ar_201_g1_i1::g.12096::m.12096	4.76	1.12	NO	
	Gene.41464::c8738_BT_Ma_ar_201_g1_i1::g.41464::m.41464	7.90	5.65	NO	
	Gene.51165::c9659_BT_Ma_ar_201_g1_i1::g.51165::m.51165	2.70	1.35	YES	
	Gene.149454::c50055_BT_Ma_ar_201_g1_i1::g.149454::m.149454	23.04	53.74	NO	
	Gene.157304::c57956_BT_Ma_ar_201_g1_i1::g.157304::m.157304	14.14	24.96	NO	
	Gene.153843::c55121_BT_Ma_ar_201_g1_i1::g.153843::m.153843	3.69	2.09	NO	
	Gene.1490::c545_BT_Ma_ar_201_g1_i1::g.1490::m.1490	3.43	2.80	NO	
	Gene.33687::c7857_BT_Ma_ar_201_g1_i1::g.33687::m.33687	3.35	1.67	YES	
	Gene.124705::c26460_BT_Ma_ar_201_g1_i1::g.124705::m.124705	10.63	6.32	NO	
	Gene.39613::c8536_BT_Ma_ar_201_g1_i1::g.39613::m.39613	6.56	1.37	NO	
	Gene.40561::c8634_BT_Ma_ar_201_g1_i1::g.40561::m.40561	2.67	2.04	NO	
	Gene.15488::c4986_BT_Ma_ar_201_g1_i2::g.15488::m.15488	2.09	0.99	YES	
	Gene.123597::c25138_BT_Ma_ar_201_g1_i1::g.123597::m.123597	10.02	10.81	NO	
	Gene.1995::c723_BT_Ma_ar_201_g1_i1::g.1995::m.1995	2.06	1.30	NO	
	Gene.3293::c1255_BT_Ma_ar_201_g1_i1::g.3293::m.3293	3.32	0.00	NO	
	Gene.10790::c3950_BT_Ma_ar_201_g1_i1::g.10790::m.10790	3.22	3.71	NO	
	Gene.121059::c22897_BT_Ma_ar_201_g1_i1::g.121059::m.121059	4.09	2.14	NO	
	Gene.1436::c528_BT_Ma_ar_201_g1_i1::g.1436::m.1436	1.91	1.20	YES	
	Gene.137313::c38531_BT_Ma_ar_201_g1_i1::g.137313::m.137313	1.93	1.59	NO	
	Gene.119922::c22165_BT_Ma_ar_201_g1_i1::g.119922::m.119922	3.56	4.81	NO	
	Gene.153744::c55051_BT_Ma_ar_201_g1_i1::g.153744::m.153744	1.66	1.24	NO	
	Gene.156195::c56923_BT_Ma_ar_201_g1_i1::g.156195::m.156195	1.79	1.63	NO	
	Gene.145017::c46255_BT_Ma_ar_201_g1_i1::g.145017::m.145017	5.98	21.84	NO	
	Gene.159071::c60342_BT_Ma_ar_201_g1_i1::g.159071::m.159071	4.73	9.22	NO	
	Gene.113279::c15250_BT_Ma_ar_201_g1_i1::g.113279::m.113279	1.86	1.87	NO	
	Gene.119324::c21833_BT_Ma_ar_201_g1_i1::g.119324::m.119324	2.81	6.16	NO	
	Gene.126400::c28481_BT_Ma_ar_201_g1_i1::g.126400::m.126400	1.51	0.96	NO	
	Gene.128053::c29469_BT_Ma_ar_201_g1_i1::g.128053::m.128053	1.29	1.29	NO	
	Gene.155391::c56261_BT_Ma_ar_201_g1_i1::g.155391::m.155391	1.11	0.57	NO	
	Gene.134787::c36968_BT_Ma_ar_201_g1_i1::g.134787::m.134787	1.45	1.32	NO	
	Gene.143418::c45395_BT_Ma_ar_201_g1_i1::g.143418::m.143418	1.44	0.78	NO	
	Gene.155860::c56665_BT_Ma_ar_201_g1_i1::g.155860::m.155860	1.05	0.90	NO	
	Gene.122735::c24250_BT_Ma_ar_201_g1_i1::g.122735::m.122735	1.17	0.83	NO	
	Gene.131575::c32545_BT_Ma_ar_201_g1_i1::g.131575::m.131575	1.71	1.70	NO	
	Gene.9480::c3417_BT_Ma_ar_201_g1_i1::g.9480::m.9480	1.61	1.64	NO	
	Gene.9486::c3417_BT_Ma_ar_201_g1_i2::g.9486::m.9486	1.61	1.64	NO	
	Gene.119325::c21833_BT_Ma_ar_201_g1_i1::g.119325::m.119325	1.89	4.83	NO	
	Gene.10661::c3899_BT_Ma_ar_201_g1_i1::g.10661::m.10661	1.28	1.51	NO	
	Gene.114145::c16000_BT_Ma_ar_201_g1_i1::g.114145::m.114145	1.23	1.28	NO	
	Gene.6462::c2270_BT_Ma_ar_201_g1_i1::g.6462::m.6462	1.18	0.77	NO	
	Gene.130107::c31106_BT_Ma_ar_201_g1_i1::g.130107::m.130107	1.33	0.63	NO	
	Gene.154627::c55644_BT_Ma_ar_201_g1_i1::g.154627::m.154627	1.31	1.56	NO	
	Gene.158634::c59619_BT_Ma_ar_201_g1_i1::g.158634::m.158634	1.77	3.19	NO	
	Gene.120696::c22671_BT_Ma_ar_201_g1_i1::g.120696::m.120696	1.54	2.61	NO	
	Gene.133502::c34879_BT_Ma_ar_201_g1_i1::g.133502::m.133502	1.36	0.80	NO	
	Gene.154399::c55485_BT_Ma_ar_201_g1_i1::g.154399::m.154399	1.36	1.61	NO	
	Gene.111655::c14095_BT_Ma_ar_201_g1_i1::g.111655::m.111655	0.98	0.96	NO	
	Gene.9212::c3304_BT_Ma_ar_201_g1_i1::g.9212::m.9212	1.07	0.86	NO	
	Gene.122172::c23753_BT_Ma_ar_201_g1_i1::g.122172::m.122172	1.36	3.56	NO	
	Gene.109512::c12903_BT_Ma_ar_201_g1_i1::g.109512::m.109512	1.31	1.61	NO	
	Gene.146857::c47569_BT_Ma_ar_201_g1_i1::g.146857::m.146857	0.89	1.40	NO	
	Gene.152953::c54624_BT_Ma_ar_201_g1_i1::g.152953::m.152953	1.39	2.11	NO	
	Gene.156219::c56949_BT_Ma_ar_201_g1_i1::g.156219::m.156219	1.09	1.26	NO	
	Gene.158335::c59201_BT_Ma_ar_201_g1_i1::g.158335::m.158335	1.06	1.18	NO	
	Gene.141997::c43361_BT_Ma_ar_201_g1_i1::g.141997::m.141997	1.18	3.40	NO	
	Gene.130412::c31398_BT_Ma_ar_201_g1_i1::g.130412::m.130412	1.20	2.11	NO	
	Gene.133251::c34529_BT_Ma_ar_201_g1_i1::g.133251::m.133251	1.98	10.96	NO	
	Gene.137986::c39026_BT_Ma_ar_201_g1_i1::g.137986::m.137986	0.92	1.57	NO	
	Gene.146528::c47313_BT_Ma_ar_201_g1_i1::g.146528::m.146528	0.82	1.27	NO	

	Gene.147327::c47936_BT_Ma_ar_201_g1_i1::g.147327::m.147327	0.97	2.20	NO	
	Gene.124025::c25602_BT_Ma_ar_201_g1_i1::g.124025::m.124025	0.81	1.08	NO	
	Gene.137069::c38349_BT_Ma_ar_201_g1_i1::g.137069::m.137069	0.76	0.97	NO	
	Gene.110477::c13341_BT_Ma_ar_201_g1_i1::g.110477::m.110477	0.85	0.94	NO	
	Gene.113310::c15272_BT_Ma_ar_201_g1_i1::g.113310::m.113310	1.36	3.00	NO	
	Gene.114597::c16436_BT_Ma_ar_201_g1_i1::g.114597::m.114597	0.82	2.20	NO	
	Gene.116837::c19009_BT_Ma_ar_201_g1_i1::g.116837::m.116837	0.79	0.82	NO	
	Gene.142097::c43512_BT_Ma_ar_201_g1_i1::g.142097::m.142097	0.82	0.44	NO	
	Gene.117195::c19440_BT_Ma_ar_201_g1_i1::g.117195::m.117195	0.85	1.17	NO	
	Gene.138146::c39156_BT_Ma_ar_201_g1_i1::g.138146::m.138146	1.04	1.89	NO	
	Gene.149616::c50233_BT_Ma_ar_201_g1_i1::g.149616::m.149616	1.09	3.22	NO	
	Gene.112958::c14992_BT_Ma_ar_201_g1_i1::g.112958::m.112958	0.76	1.70	NO	
	Gene.113894::c15791_BT_Ma_ar_201_g1_i1::g.113894::m.113894	0.57	0.32	NO	
	Gene.134294::c36182_BT_Ma_ar_201_g1_i1::g.134294::m.134294	0.80	1.28	NO	
	Gene.146351::c47192_BT_Ma_ar_201_g1_i1::g.146351::m.146351	1.09	1.42	NO	
	Gene.15487::c4986_BT_Ma_ar_201_g1_i1::g.15487::m.15487	0.80	0.00	NO	
	Gene.158813::c59871_BT_Ma_ar_201_g1_i1::g.158813::m.158813	1.16	1.68	NO	
	Gene.137596::c38731_BT_Ma_ar_201_g1_i1::g.137596::m.137596	0.97	4.27	NO	
	Gene.140516::c41485_BT_Ma_ar_201_g1_i1::g.140516::m.140516	0.54	0.72	NO	
	Gene.157641::c58310_BT_Ma_ar_201_g1_i1::g.157641::m.157641	0.72	1.97	NO	
	Gene.125096::c27059_BT_Ma_ar_201_g1_i1::g.125096::m.125096	0.81	0.00	NO	
	Gene.132617::c33701_BT_Ma_ar_201_g1_i1::g.132617::m.132617	1.07	0.00	NO	
	Gene.133874::c35490_BT_Ma_ar_201_g1_i1::g.133874::m.133874	0.56	1.53	NO	
	Gene.141222::c42331_BT_Ma_ar_201_g1_i1::g.141222::m.141222	1.09	2.97	NO	
	Gene.147082::c47749_BT_Ma_ar_201_g1_i1::g.147082::m.147082	0.71	1.75	NO	
	Gene.150965::c52010_BT_Ma_ar_201_g1_i1::g.150965::m.150965	0.74	1.97	NO	
	Gene.154986::c55936_BT_Ma_ar_201_g1_i1::g.154986::m.154986	1.02	4.82	NO	
	Gene.114371::c16221_BT_Ma_ar_201_g1_i1::g.114371::m.114371	0.59	2.43	NO	
	Gene.124673::c26406_BT_Ma_ar_201_g1_i1::g.124673::m.124673	0.50	1.10	NO	
	Gene.125945::c28291_BT_Ma_ar_201_g1_i1::g.125945::m.125945	1.01	0.00	NO	
	Gene.133089::c34325_BT_Ma_ar_201_g1_i1::g.133089::m.133089	0.59	2.43	NO	
	Gene.138344::c39317_BT_Ma_ar_201_g1_i1::g.138344::m.138344	0.72	1.35	NO	
	Gene.151941::c53598_BT_Ma_ar_201_g1_i1::g.151941::m.151941	0.65	2.05	NO	
	Gene.156474::c57138_BT_Ma_ar_201_g1_i1::g.156474::m.156474	0.80	1.88	NO	
	Gene.158262::c59097_BT_Ma_ar_201_g1_i1::g.158262::m.158262	0.58	0.00	NO	
	Gene.111601::c14064_BT_Ma_ar_201_g1_i1::g.111601::m.111601	0.60	0.00	NO	
	Gene.116311::c18279_BT_Ma_ar_201_g1_i1::g.116311::m.116311	0.75	5.11	NO	
	Gene.122591::c24117_BT_Ma_ar_201_g1_i1::g.122591::m.122591	0.55	0.00	NO	
	Gene.125403::c27559_BT_Ma_ar_201_g1_i1::g.125403::m.125403	0.59	1.28	NO	
	Gene.126349::c28450_BT_Ma_ar_201_g1_i1::g.126349::m.126349	0.64	5.04	NO	
	Gene.128266::c29630_BT_Ma_ar_201_g1_i1::g.128266::m.128266	0.59	3.85	NO	
	Gene.131259::c32224_BT_Ma_ar_201_g1_i1::g.131259::m.131259	0.59	0.00	NO	
	Gene.135506::c37319_BT_Ma_ar_201_g1_i1::g.135506::m.135506	0.71	2.27	NO	
	Gene.147050::c47723_BT_Ma_ar_201_g1_i1::g.147050::m.147050	0.62	1.50	NO	
	Gene.149245::c49820_BT_Ma_ar_201_g1_i1::g.149245::m.149245	0.63	1.58	NO	
	Gene.151173::c52292_BT_Ma_ar_201_g1_i1::g.151173::m.151173	0.64	0.00	NO	
	Gene.159205::c60618_BT_Ma_ar_201_g1_i1::g.159205::m.159205	0.60	1.35	NO	
	Gene.117452::c19777_BT_Ma_ar_201_g1_i1::g.117452::m.117452	0.44	0.00	NO	
	Gene.671::c216_BT_Ma_ar_201_g1_i1::g.671::m.671	0.64	18.65	NO	
	Gene.134266::c36126_BT_Ma_ar_201_g1_i1::g.134266::m.134266	0.48	1.35	NO	
	Gene.141252::c42369_BT_Ma_ar_201_g1_i1::g.141252::m.141252	0.54	0.00	NO	
	Gene.149203::c49776_BT_Ma_ar_201_g1_i1::g.149203::m.149203	0.50	0.00	NO	
	Gene.117199::c19446_BT_Ma_ar_201_g1_i1::g.117199::m.117199	0.34	0.00	NO	
	Gene.128409::c29745_BT_Ma_ar_201_g1_i1::g.128409::m.128409	0.17	7.33	NO	
**Phospholipase A2**	Gene.8694::c3153_BT_Ma_ar_201_g1_i1::g.8694::m.8694	23.12	16.70	NO	
	Gene.14173::c4695_BT_Ma_ar_201_g1_i1::g.14173::m.14173	24.69	16.79	YES	
	Gene.64466::c10740_BT_Ma_ar_201_g1_i2::g.64466::m.64466	8.62	5.08	YES	
	Gene.28407::c7179_BT_Ma_ar_201_g1_i1::g.28407::m.28407	5.79	3.82	NO	
	Gene.16555::c5152_BT_Ma_ar_201_g1_i1::g.16555::m.16555	3.90	3.19	NO	
	Gene.30::c29_BT_Ma_ar_201_g1_i1::g.30::m.30	2.06	1.30	NO	
	Gene.64460::c10740_BT_Ma_ar_201_g1_i1::g.64460::m.64460	3.98	1.32	NO	
	Gene.137709::c38815_BT_Ma_ar_201_g1_i1::g.137709::m.137709	2.22	1.81	NO	
	Gene.141097::c42185_BT_Ma_ar_201_g1_i1::g.141097::m.141097	0.50	7.33	NO	
**Pimplin**	Gene.153232::c54761_BT_Ma_ar_201_g1_i1::g.153232::m.153232	3.09	2.23	NO	excluded
	Gene.127906::c29389_BT_Ma_ar_201_g1_i1::g.127906::m.127906	1.93	1.94	NO	excluded
**Serpin**	Gene.58837::c10333_BT_Ma_ar_201_g1_i2::g.58837::m.58837	414.34	190.71	YES	
	Gene.58834::c10333_BT_Ma_ar_201_g1_i1::g.58834::m.58834	400.99	61.36	YES	
	Gene.58675::c10321_BT_Ma_ar_201_g1_i1::g.58675::m.58675	265.68	180.00	YES	
	Gene.118611::c21505_BT_Ma_ar_201_g1_i1::g.118611::m.118611	146.46	95.84	NO	
	Gene.22988::c6390_BT_Ma_ar_201_g1_i1::g.22988::m.22988	107.58	59.42	NO	
	Gene.54731::c10000_BT_Ma_ar_201_g1_i1::g.54731::m.54731	95.06	59.91	YES	
	Gene.144723::c46061_BT_Ma_ar_201_g1_i1::g.144723::m.144723	61.22	40.39	NO	
	Gene.15285::c4927_BT_Ma_ar_201_g1_i1::g.15285::m.15285	35.37	18.57	YES	
	Gene.15294::c4927_BT_Ma_ar_201_g1_i2::g.15294::m.15294	34.97	3.61	YES	
	Gene.16505::c5144_BT_Ma_ar_201_g1_i1::g.16505::m.16505	52.37	32.11	YES	
	Gene.3730::c1369_BT_Ma_ar_201_g1_i1::g.3730::m.3730	39.52	25.16	NO	
	Gene.38185::c8389_BT_Ma_ar_201_g1_i1::g.38185::m.38185	36.68	27.16	NO	
	Gene.53241::c9853_BT_Ma_ar_201_g1_i1::g.53241::m.53241	35.20	22.20	YES	
	Gene.53435::c9873_BT_Ma_ar_201_g1_i1::g.53435::m.53435	33.64	22.46	YES	
	Gene.77171::c11601_BT_Ma_ar_201_g1_i1::g.77171::m.77171	18.34	10.83	YES	
	Gene.85340::c12015_BT_Ma_ar_201_g1_i1::g.85340::m.85340	23.26	16.77	NO	
	Gene.4898::c1762_BT_Ma_ar_201_g1_i1::g.4898::m.4898	22.77	15.80	YES	
	Gene.4856::c1737_BT_Ma_ar_201_g1_i1::g.4856::m.4856	44.71	32.72	NO	
	Gene.56324::c10131_BT_Ma_ar_201_g1_i1::g.56324::m.56324	12.42	6.92	NO	
	Gene.62902::c10638_BT_Ma_ar_201_g1_i1::g.62902::m.62902	10.16	4.75	NO	
	Gene.62904::c10638_BT_Ma_ar_201_g1_i2::g.62904::m.62904	9.09	1.42	NO	
	Gene.128031::c29451_BT_Ma_ar_201_g1_i1::g.128031::m.128031	7.64	4.89	NO	
	Gene.56328::c10131_BT_Ma_ar_201_g1_i2::g.56328::m.56328	7.99	1.68	NO	
	Gene.25731::c6773_BT_Ma_ar_201_g1_i1::g.25731::m.25731	8.14	44.53	NO	
	Gene.9684::c3498_BT_Ma_ar_201_g1_i1::g.9684::m.9684	5.01	3.53	NO	
	Gene.9686::c3498_BT_Ma_ar_201_g1_i2::g.9686::m.9686	5.47	8.24	NO	
	Gene.154294::c55412_BT_Ma_ar_201_g1_i1::g.154294::m.154294	7.11	11.96	NO	
	Gene.127011::c28805_BT_Ma_ar_201_g1_i1::g.127011::m.127011	1.58	1.04	NO	
	Gene.12678::c4392_BT_Ma_ar_201_g1_i1::g.12678::m.12678	5.38	42.74	NO	
	Gene.7646::c2693_BT_Ma_ar_201_g1_i1::g.7646::m.7646	4.07	16.03	NO	

	Gene.120116::c22267_BT_Ma_ar_201_g1_i1::g.120116::m.120116	1.20	1.05	NO	
	Gene.2430::c891_BT_Ma_ar_201_g1_i1::g.2430::m.2430	2.23	10.63	NO	
	Gene.147289::c47907_BT_Ma_ar_201_g1_i1::g.147289::m.147289	1.91	1.99	NO	
	Gene.5423::c1979_BT_Ma_ar_201_g1_i1::g.5423::m.5423	0.61	1.42	YES	
**Sphingomyelinase**	Gene.74976::c11487_BT_Ma_ar_201_g1_i1::g.74976::m.74976	72.60	40.38	YES	
	Gene.88773::c12201_BT_Ma_ar_201_g1_i2::g.88773::m.88773	32.48	18.89	NO	
	Gene.44686::c9056_BT_Ma_ar_201_g1_i2::g.44686::m.44686	26.49	3.88	YES	
	Gene.44697::c9056_BT_Ma_ar_201_g1_i3::g.44697::m.44697	26.49	3.88	YES	
	Gene.44713::c9056_BT_Ma_ar_201_g1_i5::g.44713::m.44713	26.49	3.88	YES	
	Gene.44674::c9056_BT_Ma_ar_201_g1_i1::g.44674::m.44674	25.61	3.37	YES	
	Gene.73701::c11407_BT_Ma_ar_201_g1_i1::g.73701::m.73701	15.81	9.62	YES	
	Gene.88779::c12201_BT_Ma_ar_201_g1_i3::g.88779::m.88779	18.10	5.34	NO	
	Gene.88782::c12201_BT_Ma_ar_201_g1_i4::g.88782::m.88782	18.10	5.34	NO	
**SVWC-domain 1 (Asilidin8)**	Gene.24709::c6613_BT_Ma_ar_201_g1_i1::g.24709::m.24709	648.70	758.92	YES	
	Gene.24713::c6613_BT_Ma_ar_201_g1_i2::g.24713::m.24713	648.70	758.92	YES	
	Gene.58952::c10343_BT_Ma_ar_201_g2_i1::g.58952::m.58952	216.29	334.54	YES	
	Gene.112586::c14704_BT_Ma_ar_201_g1_i1::g.112586::m.112586	16.19	16.14	NO	
	Gene.29303::c7294_BT_Ma_ar_201_g1_i2::g.29303::m.29303	10.44	15.71	YES	
	Gene.23018::c6396_BT_Ma_ar_201_g1_i3::g.23018::m.23018	2.63	5.19	NO	
	Gene.140587::c41569_BT_Ma_ar_201_g1_i1::g.140587::m.140587	1.15	2.15	NO	
**SVWC-domain 2 (Asilidin9)**	Gene.38622::c8439_BT_Ma_ar_201_g1_i1::g.38622::m.38622	45.56	28.41	YES	
	Gene.38630::c8439_BT_Ma_ar_201_g1_i2::g.38630::m.38630	45.56	28.41	YES	
	Gene.33189::c7791_BT_Ma_ar_201_g1_i1::g.33189::m.33189	35.20	34.60	YES	
	Gene.4113::c1462_BT_Ma_ar_201_g1_i1::g.4113::m.4113	9.47	8.45	YES	
	Gene.12475::c4361_BT_Ma_ar_201_g1_i1::g.12475::m.12475	2.74	2.64	YES	
**Venom acid phosphatase-like**	Gene.75102::c11491_BT_Ma_ar_201_g2_i1::g.75102::m.75102	53.77	33.34	YES	
	Gene.50677::c9620_BT_Ma_ar_201_g1_i1::g.50677::m.50677	33.83	23.52	NO	
	Gene.70606::c11198_BT_Ma_ar_201_g1_i1::g.70606::m.70606	28.22	8.45	NO	
	Gene.70612::c11198_BT_Ma_ar_201_g1_i2::g.70612::m.70612	28.22	8.45	NO	
	Gene.42899::c8888_BT_Ma_ar_201_g1_i1::g.42899::m.42899	13.62	7.11	YES	
	Gene.120657::c22646_BT_Ma_ar_201_g1_i1::g.120657::m.120657	1.41	0.45	NO	
	Gene.138450::c39425_BT_Ma_ar_201_g1_i1::g.138450::m.138450	1.14	2.41	NO	
