# Supplementary material for: A Dipteran’s Novel Sucker Punch: Evolution of Arthropod Atypical Venom with a Neurotoxic Component in Robber Flies (Asilidae, Diptera)
Source: Toxins (Basel). 2018 Jan 5;10(1):29. doi: 10.3390/toxins10010029 (PMC5793116; doi:10.3390/toxins10010029)
Supplement: Supplementary file 1 [file toxins-10-00029-s001.zip › toxins-254261-supplementary/toxins-254261 supplementary file 5.pdf]

Protein Class	Contig ID	TPM - Segemehl	TPM - Kallisto	Signal Peptide	Protein Hit	Protein Name	false positive
**Aegyptin**	no hit	0	0	X	X		
**Alpha-amylase**	Gene.55345::c11530_g5_i1_VG_Ma_ar_203::g.55345::m.55345	3368.40	1964.30	YES	NO		
**Anophelin**	no hit	0	0	X	X		
**Apyrase**	Gene.124024::c57686_g1_i1_VG_Ma_ar_203::g.124024::m.124024	0.73	0.31	NO	NO		
	Gene.123955::c57638_g1_i1_VG_Ma_ar_203::g.123955::m.123955	0.48	0.27	NO	NO		
	Gene.84819::c16839_g1_i1_VG_Ma_ar_203::g.84819::m.84819	0.40	0.82	NO	NO		
	Gene.121725::c55358_g1_i1_VG_Ma_ar_203::g.121725::m.121725	0.29	0.46	NO	NO		
	Gene.124970::c58408_g1_i1_VG_Ma_ar_203::g.124970::m.124970	0.43	0.00	NO	NO		
	Gene.101276::c33896_g1_i1_VG_Ma_ar_203::g.101276::m.101276	0.21	0.00	NO	NO		
**CAP**	Gene.20169::c7787_g1_i1_VG_Ma_ar_203::g.20169::m.20169	9.93	5.28	YES	NO		
	Gene.28460::c9053_g1_i1_VG_Ma_ar_203::g.28460::m.28460	5.47	3.43	YES	NO		
	Gene.20913::c7931_g1_i1_VG_Ma_ar_203::g.20913::m.20913	2.80	1.39	NO	NO		
	Gene.103759::c36572_g1_i1_VG_Ma_ar_203::g.103759::m.103759	0.21	0.00	NO	NO		
**Chitinase**	Gene.17914::c7381_g1_i1_VG_Ma_ar_203::g.17914::m.17914	36.23	16.99	YES	NO		
	Gene.45193::c10777_g1_i1_VG_Ma_ar_203::g.45193::m.45193	13.59	6.97	NO	NO		
	Gene.244::c123_g1_i1_VG_Ma_ar_203::g.244::m.244	1.79	0.83	YES	NO		
	Gene.101530::c34136_g1_i1_VG_Ma_ar_203::g.101530::m.101530	1.78	4.11	NO	NO		
	Gene.80874::c13493_g1_i1_VG_Ma_ar_203::g.80874::m.80874	0.40	0.33	YES	NO		
	Gene.98145::c31275_g1_i1_VG_Ma_ar_203::g.98145::m.98145	0.92	0.45	NO	NO		
	Gene.91863::c24312_g1_i1_VG_Ma_ar_203::g.91863::m.91863	0.25	0.05	NO	NO		
	Gene.97246::c30619_g1_i1_VG_Ma_ar_203::g.97246::m.97246	0.29	0.42	NO	NO		
	Gene.107463::c40636_g1_i1_VG_Ma_ar_203::g.107463::m.107463	0.34	0.38	NO	NO		
	Gene.89624::c22433_g1_i1_VG_Ma_ar_203::g.89624::m.89624	0.23	0.00	NO	NO		
	Gene.97972::c31143_g1_i1_VG_Ma_ar_203::g.97972::m.97972	0.22	0.51	NO	NO		
	Gene.93877::c26287_g1_i1_VG_Ma_ar_203::g.93877::m.93877	0.22	0.26	NO	NO		
	Gene.116667::c49942_g1_i1_VG_Ma_ar_203::g.116667::m.116667	0.27	1.63	NO	NO		
	Gene.130459::c64248_g1_i1_VG_Ma_ar_203::g.130459::m.130459	0.30	2.26	NO	NO		
	Gene.88261::c21310_g1_i1_VG_Ma_ar_203::g.88261::m.88261	0.19	1.44	NO	NO		
	Gene.91476::c23944_g1_i1_VG_Ma_ar_203::g.91476::m.91476	0.13	0.68	NO	NO		
**Conoporin-like**	no hit	0	0	X	X		
**Csalpha/beta defensin**	Gene.11823::c5912_g1_i1_VG_Ma_ar_203::g.11823::m.11823	18.25	30.07	YES	NO		
	Gene.20857::c7918_g1_i1_VG_Ma_ar_203::g.20857::m.20857	3.77	8.18	YES	NO		
	Gene.1068::c616_g1_i1_VG_Ma_ar_203::g.1068::m.1068	1.22	0.79	NO	NO		
	Gene.5942::c3498_g1_i1_VG_Ma_ar_203::g.5942::m.5942	0.54	0.65	YES	NO		
	Gene.107433::c40613_g1_i1_VG_Ma_ar_203::g.107433::m.107433	0.49	0.87	NO	NO		
**Cystatin**	no hit	0	0	X	X		
**dICK'S**	no hit	0	0	X	X		
**Disintegrine-like**	no hit	0	0	X	X		
**Echotoxin-like**	no hit	0	0	X	X		
**Gigantoxin-like**	no hit	0	0	X	X		
**Hyaluronidase**	Gene.28757::c9090_g2_i1_VG_Ma_ar_203::g.28757::m.28757	3656.19	1457.11	YES	NO		
	Gene.28764::c9090_g2_i1_VG_Ma_ar_203::g.28764::m.28764	3619.17	261.50	YES	NO		
	Gene.28756::c9090_g1_i2_VG_Ma_ar_203::g.28756::m.28756	3388.29	2693.45	NO	NO		
	Gene.28753::c9090_g1_i1_VG_Ma_ar_203::g.28753::m.28753	143.92	192.92	NO	NO		
	Gene.3918::c2263_g1_i1_VG_Ma_ar_203::g.3918::m.3918	113.30	78.63	NO	NO		
	Gene.107592::c40726_g1_i1_VG_Ma_ar_203::g.107592::m.107592	92.16	71.71	NO	NO		
**ICK's (Asilidin1)**	Gene.14531::c6656_g1_i1_VG_Ma_ar_203::g.14531::m.14531	10503.09	34056.90	YES	YES	U-Asilidin1-Mar1a	
	Gene.1664::c945_g1_i1_VG_Ma_ar_203::g.1664::m.1664	2183.45	6137.03	YES	YES	U-Asilidin1-Mar2a	
	Gene.107024::c40279_g1_i1_VG_Ma_ar_203::g.107024::m.107024	158.90	494.40	NO	NO		
	Gene.13642::c6412_g1_i1_VG_Ma_ar_203::g.13642::m.13642	14.90	34.12	YES	YES	U-Asilidin1-Mar3a	
	Gene.16201::c7031_g1_i1_VG_Ma_ar_203::g.16201::m.16201	13.20	15.05	YES	NO		
	Gene.13643::c6412_g1_i2_VG_Ma_ar_203::g.13643::m.13643	5.89	4.83	YES	NO		
	Gene.10845::c5521_g1_i1_VG_Ma_ar_203::g.10845::m.10845	2.39	12.93	YES	NO		
	Gene.2190::c1225_g1_i1_VG_Ma_ar_203::g.2190::m.2190	1.27	0.88	YES	NO		
	Gene.15197::c6797_g1_i2_VG_Ma_ar_203::g.15197::m.15197	1.11	1.24	YES	NO		
	Gene.116437::c49732_g1_i1_VG_Ma_ar_203::g.116437::m.116437	0.74	1.02	NO	NO		
	Gene.110937::c43784_g1_i1_VG_Ma_ar_203::g.110937::m.110937	0.43	1.34	NO	NO		
	Gene.109832::c42699_g1_i1_VG_Ma_ar_203::g.109832::m.109832	0.36	1.21	NO	NO		
	Gene.8508::c4716_g1_i1_VG_Ma_ar_203::g.8508::m.8508	0.44	3.16	NO	NO		
**Kazal**	Gene.18749::c7531_g1_i2_VG_Ma_ar_203::g.18749::m.18749	1.95	1.01	YES	NO		
	Gene.18522::c7488_g1_i1_VG_Ma_ar_203::g.18522::m.18522	12.51	42.71	YES	NO		
	Gene.18740::c7531_g1_i1_VG_Ma_ar_203::g.18740::m.18740	1.75	0.09	YES	NO		
	Gene.15959::c6979_g1_i2_VG_Ma_ar_203::g.15959::m.15959	5.61	7.53	NO	NO		
	Gene.2803::c1568_g1_i2_VG_Ma_ar_203::g.2803::m.2803	1.22	0.76	NO	NO		excluded
	Gene.15957::c6979_g1_i1_VG_Ma_ar_203::g.15957::m.15957	4.55	4.12	NO	NO		
	Gene.89253::c22127_g1_i1_VG_Ma_ar_203::g.89253::m.89253	4.39	8.42	YES	NO		
	Gene.105938::c39487_g1_i1_VG_Ma_ar_203::g.105938::m.105938	0.69	0.33	NO	NO		
	Gene.21338::c8009_g1_i2_VG_Ma_ar_203::g.21338::m.21338	2.12	2.00	YES	NO		
	Gene.21333::c8009_g1_i1_VG_Ma_ar_203::g.21333::m.21333	1.80	1.20	YES	NO		
	Gene.7042::c4196_g1_i1_VG_Ma_ar_203::g.7042::m.7042	1.40	1.04	YES	NO		
	Gene.97792::c31014_g1_i1_VG_Ma_ar_203::g.97792::m.97792	0.60	0.45	NO	NO		
	Gene.88427::c21559_g1_i1_VG_Ma_ar_203::g.88427::m.88427	2.00	2.33	NO	NO		
	Gene.114606::c48259_g1_i1_VG_Ma_ar_203::g.114606::m.114606	1.15	1.48	NO	NO		
	Gene.2801::c1568_g1_i1_VG_Ma_ar_203::g.2801::m.2801	0.81	0.00	NO	NO		
	Gene.99070::c31951_g1_i1_VG_Ma_ar_203::g.99070::m.99070	0.45	0.26	YES	NO		
	Gene.106536::c39905_g1_i1_VG_Ma_ar_203::g.106536::m.106536	0.44	0.18	NO	NO		
	Gene.97136::c30550_g1_i1_VG_Ma_ar_203::g.97136::m.97136	0.43	0.29	NO	NO		
	Gene.9729::c5148_g1_i1_VG_Ma_ar_203::g.9729::m.9729	0.43	0.77	NO	NO		
	Gene.88545::c21635_g1_i1_VG_Ma_ar_203::g.88545::m.88545	0.36	0.33	NO	NO		
	Gene.101601::c34199_g1_i1_VG_Ma_ar_203::g.101601::m.101601	0.32	0.55	NO	NO		
	Gene.124742::c58235_g1_i1_VG_Ma_ar_203::g.124742::m.124742	0.25	1.36	NO	NO		
	Gene.111723::c44675_g1_i1_VG_Ma_ar_203::g.111723::m.111723	0.21	0.93	NO	NO		
	Gene.108168::c41174_g1_i1_VG_Ma_ar_203::g.108168::m.108168	0.08	1.20	NO	NO		
**Kazal (vasotab-like)**	Gene.18522::c7488_g1_i1_VG_Ma_ar_203::g.18522::m.18522	12.51	42.71	YES	NO		
	Gene.2803::c1568_g1_i2_VG_Ma_ar_203::g.2803::m.2803	1.22	0.76	NO	NO		excluded
	Gene.105938::c39487_g1_i1_VG_Ma_ar_203::g.105938::m.105938	0.69	0.33	NO	NO		
	Gene.14165::c6545_g1_i1_VG_Ma_ar_203::g.14165::m.14165	1.96	1.19	YES	NO		
	Gene.14172::c6545_g1_i2_VG_Ma_ar_203::g.14172::m.14172	1.83	0.00	YES	NO		
	Gene.114606::c48259_g1_i1_VG_Ma_ar_203::g.114606::m.114606	1.15	1.48	NO	NO		
	Gene.2801::c1568_g1_i1_VG_Ma_ar_203::g.2801::m.2801	0.81	0.00	NO	NO		
	Gene.99070::c31951_g1_i1_VG_Ma_ar_203::g.99070::m.99070	0.45	0.26	YES	NO		
	Gene.14168::c6545_g2_i1_VG_Ma_ar_203::g.14168::m.14168	0.61	1.63	YES	NO		
	Gene.97136::c30550_g1_i1_VG_Ma_ar_203::g.97136::m.97136	0.43	0.29	NO	NO		
	Gene.88545::c21635_g1_i1_VG_Ma_ar_203::g.88545::m.88545	0.36	0.33	NO	NO		
	Gene.108168::c41174_g1_i1_VG_Ma_ar_203::g.108168::m.108168	0.08	1.20	NO	NO		

**Kunitz**	Gene.21219::c7992_g1_i1_VG_Ma_ar_203::g.21219::m.21219	11.54	7.36	YES	NO		
	Gene.10407::c5358_g1_i1_VG_Ma_ar_203::g.10407::m.10407	1.16	0.51	NO	NO		excluded
	Gene.113889::c47766_g1_i1_VG_Ma_ar_203::g.113889::m.113889	1.45	0.70	NO	NO		
	Gene.16390::c7075_g1_i1_VG_Ma_ar_203::g.16390::m.16390	3.72	4.28	YES	NO		
	Gene.12590::c6122_g1_i1_VG_Ma_ar_203::g.12590::m.12590	2.18	1.82	YES	NO		
	Gene.16393::c7075_g1_i2_VG_Ma_ar_203::g.16393::m.16393	2.02	2.39	YES	NO		
	Gene.117936::c51081_g1_i1_VG_Ma_ar_203::g.117936::m.117936	1.06	0.57	NO	NO		
	Gene.80995::c13579_g1_i1_VG_Ma_ar_203::g.80995::m.80995	0.50	0.36	NO	NO		
	Gene.11874::c5925_g1_i2_VG_Ma_ar_203::g.11874::m.11874	1.25	1.21	YES	NO		
	Gene.11871::c5925_g1_i1_VG_Ma_ar_203::g.11871::m.11871	1.13	0.86	YES	NO		
	Gene.98155::c31287_g1_i1_VG_Ma_ar_203::g.98155::m.98155	0.39	0.26	YES	NO		
	Gene.99576::c32365_g1_i1_VG_Ma_ar_203::g.99576::m.99576	0.46	0.67	NO	NO		
	Gene.5595::c3272_g1_i1_VG_Ma_ar_203::g.5595::m.5595	0.45	0.40	NO	NO		
	Gene.21222::c7992_g2_i1_VG_Ma_ar_203::g.21222::m.21222	0.51	0.47	YES	NO		
	Gene.84339::c16397_g1_i1_VG_Ma_ar_203::g.84339::m.84339	0.37	0.74	YES	NO		
	Gene.114882::c48469_g1_i1_VG_Ma_ar_203::g.114882::m.114882	0.26	0.41	NO	NO		
	Gene.127770::c61022_g1_i1_VG_Ma_ar_203::g.127770::m.127770	0.32	0.00	NO	NO		
	Gene.128733::c62005_g1_i1_VG_Ma_ar_203::g.128733::m.128733	0.26	0.36	NO	NO		
**Lectin c-type**	Gene.72719::c12442_g1_i2_VG_Ma_ar_203::g.72719::m.72719	12.69	4.73	YES	NO		
	Gene.72706::c12442_g1_i1_VG_Ma_ar_203::g.72706::m.72706	11.50	1.96	YES	NO		
	Gene.74293::c12497_g1_i1_VG_Ma_ar_203::g.74293::m.74293	3.92	1.74	NO	NO		
	Gene.46843::c10911_g1_i1_VG_Ma_ar_203::g.46843::m.46843	5.55	2.57	YES	NO		excluded
	Gene.34389::c9787_g1_i1_VG_Ma_ar_203::g.34389::m.34389	12.00	7.41	NO	NO		
	Gene.34398::c9787_g1_i2_VG_Ma_ar_203::g.34398::m.34398	5.29	2.68	YES	NO		
	Gene.76981::c12591_g2_i3_VG_Ma_ar_203::g.76981::m.76981	0.86	0.52	NO	NO		
	Gene.124466::c58021_g1_i1_VG_Ma_ar_203::g.124466::m.124466	0.76	0.45	NO	NO		
	Gene.3196::c1832_g1_i1_VG_Ma_ar_203::g.3196::m.3196	0.40	0.25	NO	NO		
	Gene.123236::c57107_g1_i1_VG_Ma_ar_203::g.123236::m.123236	0.51	0.53	NO	NO		
	Gene.85764::c17880_g1_i1_VG_Ma_ar_203::g.85764::m.85764	0.32	0.19	NO	NO		
	Gene.112921::c46119_g1_i1_VG_Ma_ar_203::g.112921::m.112921	0.41	0.22	NO	NO		
	Gene.86347::c18532_g1_i1_VG_Ma_ar_203::g.86347::m.86347	0.31	1.06	NO	NO		
**Lipocalin calycins**	Gene.23605::c8362_g1_i1_VG_Ma_ar_203::g.23605::m.23605	34.32	18.76	YES	NO		
	Gene.37491::c10076_g1_i1_VG_Ma_ar_203::g.37491::m.37491	5.72	4.38	YES	NO		
**Maxadilan-like**	no hit	0	0	X	X		
**MBF2-domain**	Gene.22058::c8127_g1_i1_VG_Ma_ar_203::g.22058::m.22058	13382.33	14289.60	YES	YES		
	Gene.22705::c8233_g1_i1_VG_Ma_ar_203::g.22705::m.22705	7311.64	5886.31	YES	YES		
	Gene.30540::c9325_g1_i1_VG_Ma_ar_203::g.30540::m.30540	6483.05	6001.45	YES	YES		
	Gene.14157::c6543_g1_i1_VG_Ma_ar_203::g.14157::m.14157	2603.90	2619.80	YES	YES		
	Gene.24708::c8530_g2_i1_VG_Ma_ar_203::g.24708::m.24708	1543.09	1549.42	YES	YES		
	Gene.27218::c8887_g1_i1_VG_Ma_ar_203::g.27218::m.27218	119.03	130.91	YES	NO		
	Gene.23193::c8307_g1_i1_VG_Ma_ar_203::g.23193::m.23193	62.12	67.70	YES	NO		
	Gene.24703::c8530_g1_i1_VG_Ma_ar_203::g.24703::m.24703	60.56	51.86	YES	NO		
	Gene.14801::c6717_g1_i1_VG_Ma_ar_203::g.14801::m.14801	11.38	9.60	YES	NO		
	Gene.17235::c7246_g1_i4_VG_Ma_ar_203::g.17235::m.17235	11.92	24.34	YES	NO		
	Gene.97150::c30562_g1_i1_VG_Ma_ar_203::g.97150::m.97150	3.85	3.53	NO	NO		
	Gene.14803::c6717_g1_i2_VG_Ma_ar_203::g.14803::m.14803	3.59	3.17	YES	NO		
**Melittin-like**	no hit	0	0	X	X		
**Natterin-like (Asilidin10)**	Gene.27187::c8882_g1_i2_VG_Ma_ar_203::g.27187::m.27187	287.11	78.66	NO	YES	U-Asilidin10-Mar1a	
	Gene.27181::c8882_g1_i1_VG_Ma_ar_203::g.27181::m.27181	371.25	220.64	NO	YES	U-Asilidin10-Mar1b	
	Gene.39255::c10244_g1_i1_VG_Ma_ar_203::g.39255::m.39255	144.22	82.67	NO	NO		
	Gene.31149::c9406_g3_i1_VG_Ma_ar_203::g.31149::m.31149	62.21	44.73	NO	NO		
	Gene.30691::c9348_g2_i1_VG_Ma_ar_203::g.30691::m.30691	41.02	21.44	NO	NO		
	Gene.21548::c8046_g1_i1_VG_Ma_ar_203::g.21548::m.21548	1.60	0.60	NO	NO		
**Neurokinin-like**	no hit	0	0	X	X		
**Nitrophorin-like**	no hit	0	0	X	X		
**Novel protein (Asilidin2)**	Gene.39461::c10270_g1_i1_VG_Ma_ar_203::g.39461::m.39461	89677.87	50391.90	YES	YES	U-Asilidin2-Mar1a	
	Gene.15717::c6926_g2_i2_VG_Ma_ar_203::g.15717::m.15717	63481.01	33897.70	YES	YES	U-Asilidin2-Mar6a	
	Gene.27859::c8967_g2_i1_VG_Ma_ar_203::g.27859::m.27859	52155.36	29204.60	YES	YES	U-Asilidin2-Mar2a	
	Gene.27205::c8885_g1_i1_VG_Ma_ar_203::g.27205::m.27205	32431.17	17831.50	YES	YES	U-Asilidin2-Mar4a	
	Gene.36480::c9987_g1_i1_VG_Ma_ar_203::g.36480::m.36480	23928.14	13207.10	YES	YES	U-Asilidin2-Mar3a	
	Gene.27405::c8910_g1_i1_VG_Ma_ar_203::g.27405::m.27405	15245.66	8564.66	YES	YES	U-Asilidin2-Mar5a	
	Gene.31085::c9400_g3_i1_VG_Ma_ar_203::g.31085::m.31085	6280.55	3774.55	YES	YES	U-Asilidin2-Mar8a	
	Gene.35004::c9845_g2_i1_VG_Ma_ar_203::g.35004::m.35004	3485.47	3784.43	YES	YES	U-Asilidin2-Mar7a	
	Gene.35009::c9845_g2_i2_VG_Ma_ar_203::g.35009::m.35009	1746.77	750.04	YES	NO		
	Gene.16974::c7198_g1_i1_VG_Ma_ar_203::g.16974::m.16974	40.99	28.94	YES	NO		
	Gene.123596::c57367_g1_i1_VG_Ma_ar_203::g.123596::m.123596	3.51	2.49	NO	NO		
	Gene.11712::c5868_g1_i1_VG_Ma_ar_203::g.11712::m.11712	1.31	0.67	YES	NO		
	Gene.81909::c14296_g1_i1_VG_Ma_ar_203::g.81909::m.81909	1.25	0.59	YES	NO		
**Novel protein (Asilidin3)**	Gene.12999::c6237_g1_i1_VG_Ma_ar_203::g.12999::m.12999	102696.52	118265.00	YES	YES	U-Asilidin3-Mar1a	
	Gene.19005::c7574_g1_i1_VG_Ma_ar_203::g.19005::m.19005	64327.20	68838.30	YES	YES	U-Asilidin3-Mar1b	
**Novel protein (Asilidin4)**	Gene.28195::c9018_g3_i1_VG_Ma_ar_203::g.28195::m.28195	23364.05	25950.30	NO	YES	U-Asilidin4-Mar1a	
**Novel protein (Asilidin5)**	Gene.33857::c9725_g1_i1_VG_Ma_ar_203::g.33857::m.33857	7486.13	3753.84	YES	NO		
	Gene.105450::c39128_g1_i1_VG_Ma_ar_203::g.105450::m.105450	2819.97	2391.12	NO	NO		
	Gene.80917::c13524_g1_i1_VG_Ma_ar_203::g.80917::m.80917	1908.19	2264.93	NO	NO		
**Novel protein (Asilidin6)**	Gene.2154::c1207_g1_i2_VG_Ma_ar_203::g.2154::m.2154	1320.41	820.05	NO	YES	U-Asilidin6-Mar1a	
	Gene.2148::c1207_g1_i1_VG_Ma_ar_203::g.2148::m.2148	1420.80	202.38	NO	YES	U-Asilidin6-Mar1b	
	Gene.89382::c22237_g1_i1_VG_Ma_ar_203::g.89382::m.89382	268.31	225.25	NO	NO		
**Novel protein (Asilidin7)**	Gene.34206::c9761_g1_i1_VG_Ma_ar_203::g.34206::m.34206	483.10	406.64	YES	YES	U-Asilidin7-Mar1a	
	Gene.117828::c50982_g1_i1_VG_Ma_ar_203::g.117828::m.117828	1.30	5.91	NO	NO		
**Peptidase M12**	Gene.41932::c10499_g1_i1_VG_Ma_ar_203::g.41932::m.41932	2.03	0.78	NO	NO		
	Gene.32494::c9568_g1_i1_VG_Ma_ar_203::g.32494::m.32494	3.01	0.66	YES	NO		
	Gene.32526::c9568_g1_i3_VG_Ma_ar_203::g.32526::m.32526	3.06	0.56	YES	NO		
	Gene.64046::c12048_g1_i1_VG_Ma_ar_203::g.64046::m.64046	3.37	1.54	NO	NO		
	Gene.32514::c9568_g1_i2_VG_Ma_ar_203::g.32514::m.32514	3.18	0.09	NO	NO		
	Gene.41948::c10499_g1_i2_VG_Ma_ar_203::g.41948::m.41948	1.79	0.08	NO	NO		
	Gene.10953::c5561_g1_i2_VG_Ma_ar_203::g.10953::m.10953	2.24	1.33	YES	NO		
	Gene.64060::c12048_g1_i2_VG_Ma_ar_203::g.64060::m.64060	2.93	0.71	NO	NO		
	Gene.74611::c12509_g1_i1_VG_Ma_ar_203::g.74611::m.74611	1.38	0.60	NO	NO		
	Gene.74644::c12509_g1_i5_VG_Ma_ar_203::g.74644::m.74644	1.58	0.38	NO	NO		
	Gene.80467::c13221_g1_i1_VG_Ma_ar_203::g.80467::m.80467	0.57	0.43	NO	NO		
	Gene.117852::c51002_g1_i1_VG_Ma_ar_203::g.117852::m.117852	0.32	0.43	NO	NO		
**Peptidase M13**	Gene.57706::c11672_g1_i1_VG_Ma_ar_203::g.57706::m.57706	452.63	240.37	NO	NO		
	Gene.40742::c10398_g1_i1_VG_Ma_ar_203::g.40742::m.40742	12.47	2.04	NO	NO		
	Gene.40753::c10398_g1_i3_VG_Ma_ar_203::g.40753::m.40753	12.47	2.04	NO	NO		
	Gene.40763::c10398_g1_i4_VG_Ma_ar_203::g.40763::m.40763	12.47	2.04	NO	NO		
	Gene.51637::c11263_g1_i1_VG_Ma_ar_203::g.51637::m.51637	4.84	2.57	NO	NO		

	Gene.22282::c8168_g1_i1_VG_Ma_ar_203::g.22282::m.22282	3.94	1.52	YES	NO		
	Gene.33379::c9668_g1_i1_VG_Ma_ar_203::g.33379::m.33379	3.42	2.16	YES	NO		
	Gene.29053::c9132_g1_i1_VG_Ma_ar_203::g.29053::m.29053	3.62	1.97	YES	NO		
	Gene.24254::c8452_g1_i2_VG_Ma_ar_203::g.24254::m.24254	1.87	1.04	YES	NO		
	Gene.17881::c7373_g1_i1_VG_Ma_ar_203::g.17881::m.17881	3.25	3.39	NO	NO		
	Gene.40774::c10398_g1_i5_VG_Ma_ar_203::g.40774::m.40774	3.66	0.00	NO	NO		
	Gene.1909::c1074_g1_i1_VG_Ma_ar_203::g.1909::m.1909	0.63	0.44	NO	NO		
	Gene.108510::c41473_g1_i1_VG_Ma_ar_203::g.108510::m.108510	0.43	0.26	NO	NO		
	Gene.125690::c59014_g1_i1_VG_Ma_ar_203::g.125690::m.125690	0.66	0.49	NO	NO		
	Gene.80980::c13569_g1_i1_VG_Ma_ar_203::g.80980::m.80980	0.48	0.27	NO	NO		
	Gene.101785::c34397_g1_i1_VG_Ma_ar_203::g.101785::m.101785	0.52	0.34	NO	NO		
	Gene.106580::c39940_g1_i1_VG_Ma_ar_203::g.106580::m.106580	0.62	0.28	NO	NO		
	Gene.109722::c42596_g1_i1_VG_Ma_ar_203::g.109722::m.109722	0.52	0.54	NO	NO		
	Gene.80304::c13096_g1_i1_VG_Ma_ar_203::g.80304::m.80304	0.43	0.44	NO	NO		
	Gene.99226::c32057_g1_i1_VG_Ma_ar_203::g.99226::m.99226	0.38	0.55	NO	NO		
	Gene.445::c214_g1_i1_VG_Ma_ar_203::g.445::m.445	0.44	0.45	NO	NO		
	Gene.100288::c32968_g1_i1_VG_Ma_ar_203::g.100288::m.100288	0.36	0.31	NO	NO		
	Gene.98425::c31492_g1_i1_VG_Ma_ar_203::g.98425::m.98425	0.29	0.24	NO	NO		
	Gene.109140::c42062_g1_i1_VG_Ma_ar_203::g.109140::m.109140	0.34	0.33	NO	NO		
	Gene.83738::c15823_g1_i1_VG_Ma_ar_203::g.83738::m.83738	0.45	0.67	YES	NO		
	Gene.85161::c17175_g1_i1_VG_Ma_ar_203::g.85161::m.85161	0.39	0.00	NO	NO		
	Gene.87714::c20274_g1_i1_VG_Ma_ar_203::g.87714::m.87714	0.33	0.24	NO	NO		
	Gene.116261::c49583_g1_i1_VG_Ma_ar_203::g.116261::m.116261	0.36	0.28	NO	NO		
	Gene.93430::c25834_g1_i1_VG_Ma_ar_203::g.93430::m.93430	0.37	0.23	NO	NO		
	Gene.90723::c23314_g1_i1_VG_Ma_ar_203::g.90723::m.90723	0.38	1.27	NO	NO		
	Gene.120764::c54070_g1_i1_VG_Ma_ar_203::g.120764::m.120764	0.36	0.55	NO	NO		
	Gene.84463::c16497_g1_i1_VG_Ma_ar_203::g.84463::m.84463	0.24	0.15	YES	NO		
	Gene.128121::c61390_g1_i1_VG_Ma_ar_203::g.128121::m.128121	0.29	0.70	NO	NO		
	Gene.2603::c1433_g1_i1_VG_Ma_ar_203::g.2603::m.2603	0.34	0.49	NO	NO		
	Gene.94349::c26824_g1_i1_VG_Ma_ar_203::g.94349::m.94349	0.24	0.00	NO	NO		
	Gene.87683::c20228_g1_i1_VG_Ma_ar_203::g.87683::m.87683	0.24	1.03	NO	NO		
	Gene.88118::c20979_g1_i1_VG_Ma_ar_203::g.88118::m.88118	0.32	0.72	NO	NO		
	Gene.108651::c41604_g1_i1_VG_Ma_ar_203::g.108651::m.108651	0.34	1.63	NO	NO		
	Gene.115761::c49178_g1_i1_VG_Ma_ar_203::g.115761::m.115761	0.25	0.74	NO	NO		
	Gene.127808::c61059_g1_i1_VG_Ma_ar_203::g.127808::m.127808	0.23	0.59	NO	NO		
	Gene.95007::c27539_g1_i1_VG_Ma_ar_203::g.95007::m.95007	0.21	0.39	NO	NO		
	Gene.102092::c34726_g1_i1_VG_Ma_ar_203::g.102092::m.102092	0.26	1.44	NO	NO		
	Gene.115277::c48786_g1_i1_VG_Ma_ar_203::g.115277::m.115277	0.20	0.00	NO	NO		
	Gene.82416::c14711_g1_i1_VG_Ma_ar_203::g.82416::m.82416	0.16	0.00	NO	NO		
	Gene.87957::c20683_g1_i1_VG_Ma_ar_203::g.87957::m.87957	0.18	1.14	NO	NO		
**Peptidase M14**	Gene.52743::c11342_g1_i2_VG_Ma_ar_203::g.52743::m.52743	27.97	11.42	YES	NO		
	Gene.52731::c11342_g1_i1_VG_Ma_ar_203::g.52731::m.52731	26.33	1.78	YES	NO		
	Gene.18855::c7548_g1_i1_VG_Ma_ar_203::g.18855::m.18855	2.07	0.96	YES	NO		
	Gene.81848::c14254_g1_i1_VG_Ma_ar_203::g.81848::m.81848	0.46	0.23	NO	NO		
	Gene.83718::c15804_g1_i1_VG_Ma_ar_203::g.83718::m.83718	0.40	0.12	NO	NO		
	Gene.107924::c40989_g1_i1_VG_Ma_ar_203::g.107924::m.107924	0.82	1.65	NO	NO		
	Gene.92348::c24751_g1_i1_VG_Ma_ar_203::g.92348::m.92348	0.43	0.62	NO	NO		
	Gene.117224::c50433_g1_i1_VG_Ma_ar_203::g.117224::m.117224	0.32	0.65	NO	NO		
	Gene.103088::c35760_g1_i1_VG_Ma_ar_203::g.103088::m.103088	0.46	1.53	NO	NO		
	Gene.100884::c33514_g1_i1_VG_Ma_ar_203::g.100884::m.100884	0.33	1.40	NO	NO		
	Gene.95347::c27944_g1_i1_VG_Ma_ar_203::g.95347::m.95347	0.34	0.00	NO	NO		
	Gene.111775::c44732_g1_i1_VG_Ma_ar_203::g.111775::m.111775	0.26	0.77	NO	NO		
	Gene.129656::c63102_g1_i1_VG_Ma_ar_203::g.129656::m.129656	0.23	0.00	NO	NO		
**Peptidase S10**	Gene.43100::c10605_g1_i1_VG_Ma_ar_203::g.43100::m.43100	44.04	21.15	YES	NO		
	Gene.127482::c60736_g1_i1_VG_Ma_ar_203::g.127482::m.127482	0.85	0.43	NO	NO		
	Gene.91327::c23808_g1_i1_VG_Ma_ar_203::g.91327::m.91327	0.22	0.89	NO	NO		
**Peptidase S1**	Gene.24008::c8418_g1_i1_VG_Ma_ar_203::g.24008::m.24008	25032.39	14354.20	YES	YES		
	Gene.49420::c11103_g1_i1_VG_Ma_ar_203::g.49420::m.49420	9902.62	7107.75	YES	YES		
	Gene.21993::c8116_g1_i1_VG_Ma_ar_203::g.21993::m.21993	7318.34	4550.38	YES	YES		
	Gene.20901::c7927_g1_i1_VG_Ma_ar_203::g.20901::m.20901	7824.18	4699.38	NO	NO		
	Gene.30642::c9342_g1_i1_VG_Ma_ar_203::g.30642::m.30642	1077.82	520.44	YES	NO		
	Gene.33669::c9700_g1_i1_VG_Ma_ar_203::g.33669::m.33669	1347.46	992.35	YES	NO		
	Gene.34279::c9773_g1_i1_VG_Ma_ar_203::g.34279::m.34279	952.75	436.57	YES	YES		
	Gene.34284::c9773_g1_i2_VG_Ma_ar_203::g.34284::m.34284	865.63	165.88	YES	YES		
	Gene.3304::c1909_g1_i1_VG_Ma_ar_203::g.3304::m.3304	825.20	688.64	NO	NO		
	Gene.7767::c4486_g1_i1_VG_Ma_ar_203::g.7767::m.7767	198.47	109.06	NO	NO		
	Gene.29134::c9144_g1_i1_VG_Ma_ar_203::g.29134::m.29134	150.01	77.21	YES	NO		
	Gene.29140::c9144_g1_i2_VG_Ma_ar_203::g.29140::m.29140	146.09	43.37	YES	NO		
	Gene.18172::c7426_g1_i1_VG_Ma_ar_203::g.18172::m.18172	98.78	69.23	NO	NO		
	Gene.32804::c9593_g1_i2_VG_Ma_ar_203::g.32804::m.32804	40.47	24.90	NO	NO		
	Gene.53083::c11368_g1_i1_VG_Ma_ar_203::g.53083::m.53083	16.84	3.47	NO	NO		
	Gene.44359::c10710_g1_i1_VG_Ma_ar_203::g.44359::m.44359	15.79	8.02	YES	NO		
	Gene.37774::c10097_g1_i1_VG_Ma_ar_203::g.37774::m.37774	18.47	10.14	YES	NO		
	Gene.53088::c11368_g1_i2_VG_Ma_ar_203::g.53088::m.53088	31.94	17.91	NO	NO		
	Gene.17508::c7298_g1_i1_VG_Ma_ar_203::g.17508::m.17508	15.82	8.79	YES	NO		
	Gene.21994::c8116_g1_i1_VG_Ma_ar_203::g.21994::m.21994	15.71	9.10	YES	NO		
	Gene.44363::c10710_g1_i2_VG_Ma_ar_203::g.44363::m.44363	9.81	5.18	YES	NO		
	Gene.71236::c12373_g1_i1_VG_Ma_ar_203::g.71236::m.71236	7.72	2.43	NO	NO		
	Gene.71239::c12373_g1_i2_VG_Ma_ar_203::g.71239::m.71239	7.78	1.90	NO	NO		
	Gene.116699::c49966_g1_i1_VG_Ma_ar_203::g.116699::m.116699	8.83	4.12	NO	NO		
	Gene.1514::c880_g1_i1_VG_Ma_ar_203::g.1514::m.1514	8.89	4.27	YES	NO		
	Gene.17550::c7307_g1_i1_VG_Ma_ar_203::g.17550::m.17550	6.25	3.41	NO	NO		
	Gene.53087::c11368_g1_i2_VG_Ma_ar_203::g.53087::m.53087	7.75	1.98	NO	NO		
	Gene.25395::c8629_g1_i1_VG_Ma_ar_203::g.25395::m.25395	5.37	1.68	YES	NO		
	Gene.25397::c8629_g1_i2_VG_Ma_ar_203::g.25397::m.25397	5.37	1.68	YES	NO		
	Gene.81088::c13658_g1_i1_VG_Ma_ar_203::g.81088::m.81088	4.82	2.77	YES	NO		
	Gene.23988::c8416_g1_i1_VG_Ma_ar_203::g.23988::m.23988	3.17	1.33	YES	NO		
	Gene.36312::c9972_g1_i2_VG_Ma_ar_203::g.36312::m.36312	2.53	1.15	YES	NO		
	Gene.7233::c4273_g1_i1_VG_Ma_ar_203::g.7233::m.7233	4.75	2.55	YES	NO		
	Gene.32896::c9607_g1_i1_VG_Ma_ar_203::g.32896::m.32896	4.77	2.58	YES	NO		
	Gene.37977::c10118_g1_i1_VG_Ma_ar_203::g.37977::m.37977	2.79	1.47	YES	NO		
	Gene.36304::c9972_g1_i1_VG_Ma_ar_203::g.36304::m.36304	2.13	0.89	YES	NO		
	Gene.99340::c32155_g1_i1_VG_Ma_ar_203::g.99340::m.99340	3.59	1.71	YES	NO		
	Gene.19467::c7663_g1_i1_VG_Ma_ar_203::g.19467::m.19467	3.28	1.75	YES	NO		
	Gene.20757::c7898_g1_i1_VG_Ma_ar_203::g.20757::m.20757	2.42	1.21	YES	NO		
	Gene.538::c263_g1_i1_VG_Ma_ar_203::g.538::m.538	2.32	1.14	YES	NO		
	Gene.22476::c8197_g1_i1_VG_Ma_ar_203::g.22476::m.22476	1.65	0.85	YES	NO		
	Gene.97292::c30654_g1_i1_VG_Ma_ar_203::g.97292::m.97292	1.64	0.97	YES	NO		
	Gene.8754::c4799_g1_i1_VG_Ma_ar_203::g.8754::m.8754	2.01	1.08	NO	NO		
	Gene.14070::c6525_g1_i1_VG_Ma_ar_203::g.14070::m.14070	1.04	0.48	NO	NO		excluded
	Gene.91176::c23679_g1_i1_VG_Ma_ar_203::g.91176::m.91176	1.94	0.81	NO	NO		
	Gene.14390::c6616_g1_i1_VG_Ma_ar_203::g.14390::m.14390	2.14	1.15	YES	NO		
	Gene.3939::c2275_g1_i1_VG_Ma_ar_203::g.3939::m.3939	2.24	1.18	YES	NO		
	Gene.122596::c56668_g1_i1_VG_Ma_ar_203::g.122596::m.122596	1.35	0.55	NO	NO		
	Gene.9132::c4934_g1_i1_VG_Ma_ar_203::g.9132::m.9132	0.96	0.57	YES	NO		
	Gene.21425::c8027_g1_i1_VG_Ma_ar_203::g.21425::m.21425	1.74	0.97	NO	NO		
	Gene.6600::c3929_g1_i1_VG_Ma_ar_203::g.6600::m.6600	9.82	22.37	NO	NO		
	Gene.11041::c5590_g1_i1_VG_Ma_ar_203::g.11041::m.11041	1.06	0.57	NO	NO		
	Gene.15847::c6958_g2_i1_VG_Ma_ar_203::g.15847::m.15847	0.75	0.47	NO	NO		
	Gene.92146::c24578_g1_i1_VG_Ma_ar_203::g.92146::m.92146	1.70	0.89	YES	NO		
	Gene.22189::c8146_g2_i1_VG_Ma_ar_203::g.22189::m.22189	0.94	0.62	YES	NO		
	Gene.98141::c31272_g1_i1_VG_Ma_ar_203::g.98141::m.98141	5.11	2.60	NO	NO		
	Gene.13246::c6310_g1_i1_VG_Ma_ar_203::g.13246::m.13246	1.17	0.73	YES	NO		
	Gene.5139::c3031_g1_i1_VG_Ma_ar_203::g.5139::m.5139	5.72	20.59	NO	NO		
	Gene.6912::c4142_g1_i1_VG_Ma_ar_203::g.6912::m.6912	1.36	0.90	NO	NO		
	Gene.8769::c4803_g1_i1_VG_Ma_ar_203::g.8769::m.8769	1.03	0.53	YES	NO		
	Gene.7924::c4528_g1_i1_VG_Ma_ar_203::g.7924::m.7924	0.88	0.54	YES	NO		
	Gene.116522::c49813_g1_i1_VG_Ma_ar_203::g.116522::m.116522	4.08	5.72	NO	NO		
	Gene.6224::c3683_g1_i1_VG_Ma_ar_203::g.6224::m.6224	0.88	0.52	NO	NO		
	Gene.116123::c49487_g1_i1_VG_Ma_ar_203::g.116123::m.116123	0.98	0.41	NO	NO		
	Gene.122761::c56769_g1_i1_VG_Ma_ar_203::g.122761::m.122761	1.24	0.54	NO	NO		
	Gene.101437::c34050_g1_i1_VG_Ma_ar_203::g.101437::m.101437	3.72	6.69	NO	NO		
	Gene.81316::c13819_g1_i1_VG_Ma_ar_203::g.81316::m.81316	0.65	0.28	YES	NO		

	Gene.125982::c59271_g1_i1_VG_Ma_ar_203::g.125982::m.125982	0.84	0.57	NO	NO
	Gene.88318::c21487_g1_i1_VG_Ma_ar_203::g.88318::m.88318	0.56	0.32	NO	NO
	Gene.10160::c5290_g1_i1_VG_Ma_ar_203::g.10160::m.10160	1.24	0.85	YES	NO
	Gene.4502::c2602_g1_i1_VG_Ma_ar_203::g.4502::m.4502	0.72	0.49	NO	NO
	Gene.6015::c3551_g1_i1_VG_Ma_ar_203::g.6015::m.6015	0.87	0.69	NO	NO
	Gene.755::c396_g1_i1_VG_Ma_ar_203::g.755::m.755	0.71	0.39	NO	NO
	Gene.2552::c1411_g1_i1_VG_Ma_ar_203::g.2552::m.2552	2.10	4.34	NO	NO
	Gene.85705::c17813_g1_i1_VG_Ma_ar_203::g.85705::m.85705	0.59	0.40	NO	NO
	Gene.116244::c49574_g1_i1_VG_Ma_ar_203::g.116244::m.116244	0.53	0.32	NO	NO
	Gene.80001::c12877_g1_i1_VG_Ma_ar_203::g.80001::m.80001	0.65	0.33	NO	NO
	Gene.98436::c31498_g1_i1_VG_Ma_ar_203::g.98436::m.98436	0.69	0.48	YES	NO
	Gene.90704::c23301_g1_i1_VG_Ma_ar_203::g.90704::m.90704	0.51	0.41	YES	NO
	Gene.97836::c31040_g1_i1_VG_Ma_ar_203::g.97836::m.97836	0.63	0.23	YES	NO
	Gene.4090::c2349_g1_i1_VG_Ma_ar_203::g.4090::m.4090	0.52	0.23	NO	NO
	Gene.88372::c21520_g1_i1_VG_Ma_ar_203::g.88372::m.88372	0.62	0.33	NO	NO
	Gene.113947::c47808_g1_i1_VG_Ma_ar_203::g.113947::m.113947	0.43	0.21	NO	NO
	Gene.101927::c34537_g1_i1_VG_Ma_ar_203::g.101927::m.101927	0.64	0.46	NO	NO
	Gene.126156::c59434_g1_i1_VG_Ma_ar_203::g.126156::m.126156	0.60	0.30	NO	NO
	Gene.84248::c16302_g1_i1_VG_Ma_ar_203::g.84248::m.84248	0.71	0.96	NO	NO
	Gene.98247::c31355_g1_i1_VG_Ma_ar_203::g.98247::m.98247	0.63	0.51	NO	NO
	Gene.100276::c32961_g1_i1_VG_Ma_ar_203::g.100276::m.100276	0.46	0.40	NO	NO
	Gene.98055::c31202_g1_i1_VG_Ma_ar_203::g.98055::m.98055	0.54	0.15	NO	NO
	Gene.83612::c15705_g1_i1_VG_Ma_ar_203::g.83612::m.83612	0.85	0.98	NO	NO
	Gene.3531::c2042_g1_i1_VG_Ma_ar_203::g.3531::m.3531	0.64	0.44	NO	NO
	Gene.82679::c14914_g1_i1_VG_Ma_ar_203::g.82679::m.82679	0.71	0.89	NO	NO
	Gene.102365::c35010_g1_i1_VG_Ma_ar_203::g.102365::m.102365	0.37	0.37	NO	NO
	Gene.107008::c40267_g1_i1_VG_Ma_ar_203::g.107008::m.107008	0.32	0.17	NO	NO
	Gene.122754::c56765_g1_i1_VG_Ma_ar_203::g.122754::m.122754	0.40	0.18	NO	NO
	Gene.127849::c61095_g1_i1_VG_Ma_ar_203::g.127849::m.127849	1.38	2.58	NO	NO
	Gene.107855::c40935_g1_i1_VG_Ma_ar_203::g.107855::m.107855	0.86	2.27	NO	NO
	Gene.127108::c60352_g1_i1_VG_Ma_ar_203::g.127108::m.127108	0.33	0.24	NO	NO
	Gene.6255::c3702_g1_i1_VG_Ma_ar_203::g.6255::m.6255	0.50	0.41	YES	NO
	Gene.126776::c60034_g1_i1_VG_Ma_ar_203::g.126776::m.126776	0.40	0.08	NO	NO
	Gene.82630::c14875_g1_i1_VG_Ma_ar_203::g.82630::m.82630	0.40	0.26	NO	NO
	Gene.84907::c16942_g1_i1_VG_Ma_ar_203::g.84907::m.84907	0.81	0.89	NO	NO
	Gene.92213::c24641_g1_i1_VG_Ma_ar_203::g.92213::m.92213	0.41	0.11	NO	NO
	Gene.102076::c34713_g1_i1_VG_Ma_ar_203::g.102076::m.102076	0.25	0.15	NO	NO
	Gene.103792::c36616_g1_i1_VG_Ma_ar_203::g.103792::m.103792	0.62	1.09	NO	NO
	Gene.81272::c13783_g1_i1_VG_Ma_ar_203::g.81272::m.81272	0.38	0.40	NO	NO
	Gene.92884::c25269_g1_i1_VG_Ma_ar_203::g.92884::m.92884	0.43	0.68	NO	NO
	Gene.114971::c48537_g1_i1_VG_Ma_ar_203::g.114971::m.114971	0.28	0.23	NO	NO
	Gene.23995::c8416_g1_i2_VG_Ma_ar_203::g.23995::m.23995	0.57	1.27	NO	NO
	Gene.82895::c15084_g1_i1_VG_Ma_ar_203::g.82895::m.82895	0.37	0.30	NO	NO
	Gene.117793::c50956_g1_i1_VG_Ma_ar_203::g.117793::m.117793	0.38	0.64	NO	NO
	Gene.119272::c52333_g1_i1_VG_Ma_ar_203::g.119272::m.119272	0.50	0.57	NO	NO
	Gene.127058::c60300_g1_i1_VG_Ma_ar_203::g.127058::m.127058	0.59	1.28	NO	NO
	Gene.127642::c60904_g1_i1_VG_Ma_ar_203::g.127642::m.127642	0.32	0.55	NO	NO
	Gene.89519::c22345_g1_i1_VG_Ma_ar_203::g.89519::m.89519	0.67	3.25	NO	NO
	Gene.10145::c5286_g1_i1_VG_Ma_ar_203::g.10145::m.10145	0.42	0.46	NO	NO
	Gene.124973::c58410_g1_i1_VG_Ma_ar_203::g.124973::m.124973	0.35	0.32	NO	NO
	Gene.125260::c58648_g1_i1_VG_Ma_ar_203::g.125260::m.125260	0.27	0.20	NO	NO
	Gene.127264::c60506_g1_i1_VG_Ma_ar_203::g.127264::m.127264	0.33	0.58	NO	NO
	Gene.85545::c17639_g1_i1_VG_Ma_ar_203::g.85545::m.85545	0.33	0.34	NO	NO
	Gene.94386::c26866_g1_i1_VG_Ma_ar_203::g.94386::m.94386	0.28	0.13	NO	NO
	Gene.10147::c5286_g1_i2_VG_Ma_ar_203::g.10147::m.10147	0.40	0.00	NO	NO
	Gene.129156::c62487_g1_i1_VG_Ma_ar_203::g.129156::m.129156	0.37	0.90	NO	NO
	Gene.85319::c17347_g1_i1_VG_Ma_ar_203::g.85319::m.85319	0.25	0.16	NO	NO
	Gene.90725::c23316_g1_i1_VG_Ma_ar_203::g.90725::m.90725	0.30	0.49	NO	NO
	Gene.90963::c23496_g1_i1_VG_Ma_ar_203::g.90963::m.90963	0.22	0.13	NO	NO
	Gene.94004::c26439_g1_i1_VG_Ma_ar_203::g.94004::m.94004	0.33	0.31	NO	NO
	Gene.116568::c49850_g1_i1_VG_Ma_ar_203::g.116568::m.116568	0.30	0.24	NO	NO
	Gene.117319::c50511_g1_i1_VG_Ma_ar_203::g.117319::m.117319	0.29	0.23	NO	NO
	Gene.128023::c61288_g1_i1_VG_Ma_ar_203::g.128023::m.128023	0.28	0.22	NO	NO
	Gene.79698::c12688_g1_i1_VG_Ma_ar_203::g.79698::m.79698	0.40	1.53	NO	NO
	Gene.80394::c13162_g1_i1_VG_Ma_ar_203::g.80394::m.80394	0.28	0.31	NO	NO
	Gene.100636::c33280_g1_i1_VG_Ma_ar_203::g.100636::m.100636	0.36	1.14	NO	NO
	Gene.104598::c37672_g1_i1_VG_Ma_ar_203::g.104598::m.104598	0.24	0.00	NO	NO
	Gene.118623::c51716_g1_i1_VG_Ma_ar_203::g.118623::m.118623	0.27	0.28	NO	NO
	Gene.119693::c52830_g1_i1_VG_Ma_ar_203::g.119693::m.119693	0.26	0.26	NO	NO
	Gene.119868::c53006_g1_i1_VG_Ma_ar_203::g.119868::m.119868	0.34	0.49	NO	NO
	Gene.122243::c56194_g1_i1_VG_Ma_ar_203::g.122243::m.122243	0.29	0.33	NO	NO
	Gene.81607::c14081_g1_i1_VG_Ma_ar_203::g.81607::m.81607	0.34	0.86	NO	NO
	Gene.83981::c16046_g1_i1_VG_Ma_ar_203::g.83981::m.83981	0.23	0.57	NO	NO
	Gene.84182::c16242_g1_i1_VG_Ma_ar_203::g.84182::m.84182	0.31	0.00	NO	NO
	Gene.95234::c27797_g1_i1_VG_Ma_ar_203::g.95234::m.95234	0.25	0.00	NO	NO
	Gene.95765::c28492_g1_i1_VG_Ma_ar_203::g.95765::m.95765	0.25	0.36	NO	NO
	Gene.97768::c30999_g1_i1_VG_Ma_ar_203::g.97768::m.97768	0.25	0.74	NO	NO
	Gene.98698::c31670_g1_i1_VG_Ma_ar_203::g.98698::m.98698	0.32	0.72	NO	NO
	Gene.100200::c32892_g1_i1_VG_Ma_ar_203::g.100200::m.100200	0.25	0.00	NO	NO
	Gene.103757::c36570_g1_i1_VG_Ma_ar_203::g.103757::m.103757	0.23	0.00	NO	NO
	Gene.109919::c42775_g1_i1_VG_Ma_ar_203::g.109919::m.109919	0.38	1.13	NO	NO
	Gene.128811::c62095_g1_i1_VG_Ma_ar_203::g.128811::m.128811	0.18	0.50	NO	NO
	Gene.81540::c14028_g1_i1_VG_Ma_ar_203::g.81540::m.81540	0.22	0.00	NO	NO
	Gene.85795::c17908_g1_i1_VG_Ma_ar_203::g.85795::m.85795	0.21	0.81	NO	NO
	Gene.86524::c18724_g1_i1_VG_Ma_ar_203::g.86524::m.86524	0.21	1.28	NO	NO
	Gene.88192::c21109_g1_i1_VG_Ma_ar_203::g.88192::m.88192	0.20	0.00	NO	NO
	Gene.89496::c22320_g1_i1_VG_Ma_ar_203::g.89496::m.89496	0.20	1.49	NO	NO
	Gene.92122::c24551_g1_i1_VG_Ma_ar_203::g.92122::m.92122	0.22	0.47	NO	NO
	Gene.93718::c26142_g1_i1_VG_Ma_ar_203::g.93718::m.93718	0.25	0.64	NO	NO
	Gene.101065::c33690_g1_i1_VG_Ma_ar_203::g.101065::m.101065	0.24	0.57	NO	NO
	Gene.6747::c4040_g1_i1_VG_Ma_ar_203::g.6747::m.6747	0.28	2.58	NO	NO
	Gene.114083::c47904_g1_i1_VG_Ma_ar_203::g.114083::m.114083	0.22	0.45	NO	NO
	Gene.121360::c54863_g1_i1_VG_Ma_ar_203::g.121360::m.121360	0.21	0.81	NO	NO
	Gene.127033::c60277_g1_i1_VG_Ma_ar_203::g.127033::m.127033	0.18	0.30	NO	NO
	Gene.82482::c14760_g1_i1_VG_Ma_ar_203::g.82482::m.82482	0.19	1.44	NO	NO
	Gene.88238::c21245_g1_i1_VG_Ma_ar_203::g.88238::m.88238	0.22	0.00	NO	NO
	Gene.90479::c23111_g1_i1_VG_Ma_ar_203::g.90479::m.90479	0.19	0.64	NO	NO
	Gene.91730::c24177_g1_i1_VG_Ma_ar_203::g.91730::m.91730	0.17	0.47	NO	NO
	Gene.96477::c29502_g1_i1_VG_Ma_ar_203::g.96477::m.96477	0.22	1.97	NO	NO
	Gene.98130::c31261_g1_i1_VG_Ma_ar_203::g.98130::m.98130	0.19	1.44	NO	NO
	Gene.99927::c32658_g1_i1_VG_Ma_ar_203::g.99927::m.99927	0.19	2.04	NO	NO
	Gene.104939::c38178_g1_i1_VG_Ma_ar_203::g.104939::m.104939	0.22	0.00	NO	NO
	Gene.112721::c45833_g1_i1_VG_Ma_ar_203::g.112721::m.112721	0.23	0.00	NO	NO
	Gene.112810::c45948_g1_i1_VG_Ma_ar_203::g.112810::m.112810	0.22	1.05	NO	NO
	Gene.113872::c47717_g1_i1_VG_Ma_ar_203::g.113872::m.113872	0.19	0.68	NO	NO
	Gene.114903::c48480_g1_i1_VG_Ma_ar_203::g.114903::m.114903	0.14	0.30	NO	NO
	Gene.119340::c52418_g1_i1_VG_Ma_ar_203::g.119340::m.119340	0.21	0.00	NO	NO
	Gene.85145::c17162_g1_i1_VG_Ma_ar_203::g.85145::m.85145	0.13	1.44	NO	NO
	Gene.89608::c22421_g1_i1_VG_Ma_ar_203::g.89608::m.89608	0.16	0.00	NO	NO
	Gene.101242::c33866_g1_i1_VG_Ma_ar_203::g.101242::m.101242	0.15	0.00	NO	NO
	Gene.107595::c40730_g1_i1_VG_Ma_ar_203::g.107595::m.107595	0.12	0.00	NO	NO
	Gene.116541::c49827_g1_i1_VG_Ma_ar_203::g.116541::m.116541	0.12	0.61	NO	NO
**Phospholipase A2**	Gene.22371::c8182_g2_i1_VG_Ma_ar_203::g.22371::m.22371	2615.27	1738.45	YES	YES
	Gene.25984::c8719_g1_i2_VG_Ma_ar_203::g.25984::m.25984	10.47	5.93	NO	NO
	Gene.25982::c8719_g1_i1_VG_Ma_ar_203::g.25982::m.25982	9.67	5.41	NO	NO
	Gene.23736::c8388_g1_i2_VG_Ma_ar_203::g.23736::m.23736	3.42	1.80	YES	NO
	Gene.1785::c1013_g1_i1_VG_Ma_ar_203::g.1785::m.1785	2.10	1.32	NO	NO
	Gene.22370::c8182_g2_i1_VG_Ma_ar_203::g.22370::m.22370	1.81	1.38	YES	NO
	Gene.111469::c44364_g1_i1_VG_Ma_ar_203::g.111469::m.111469	0.37	0.64	NO	NO
**Pimplin**	Gene.6389::c3792_g1_i1_VG_Ma_ar_203::g.6389::m.6389	0.72	0.24	NO	NO
**Serpin**	Gene.39303::c10250_g1_i1_VG_Ma_ar_203::g.39303::m.39303	12293.05	6538.48	YES	NO
	Gene.23038::c8283_g1_i1_VG_Ma_ar_203::g.23038::m.23038	265.44	112.04	YES	NO
	Gene.23042::c8283_g1_i2_VG_Ma_ar_203::g.23042::m.23042	255.92	26.67	YES	NO

	Gene.18821::c7540_g1_i1_VG_Ma_ar_203::g.18821::m.18821	154.52	82.76	YES	NO		
	Gene.88604::c21663_g1_i1_VG_Ma_ar_203::g.88604::m.88604	1046.99	5398.06	NO	NO		
	Gene.39631::c10286_g1_i1_VG_Ma_ar_203::g.39631::m.39631	50.23	25.82	YES	NO		
	Gene.36691::c10004_g1_i2_VG_Ma_ar_203::g.36691::m.36691	33.09	15.63	NO	NO		
	Gene.36685::c10004_g1_i1_VG_Ma_ar_203::g.36685::m.36685	32.48	2.24	YES	NO		
	Gene.36698::c10004_g1_i3_VG_Ma_ar_203::g.36698::m.36698	31.26	0.38	NO	NO		
	Gene.25783::c8691_g1_i2_VG_Ma_ar_203::g.25783::m.25783	8.73	4.38	YES	NO		
	Gene.25787::c8691_g1_i3_VG_Ma_ar_203::g.25787::m.25787	8.60	0.34	YES	NO		
	Gene.28746::c9089_g1_i2_VG_Ma_ar_203::g.28746::m.28746	2.41	1.34	YES	NO		
	Gene.41224::c10441_g1_i1_VG_Ma_ar_203::g.41224::m.41224	4.10	2.28	YES	NO		
	Gene.18923::c7561_g1_i1_VG_Ma_ar_203::g.18923::m.18923	26.97	52.57	NO	NO		
	Gene.16482::c7092_g1_i1_VG_Ma_ar_203::g.16482::m.16482	3.49	1.90	YES	NO		
	Gene.49092::c11076_g1_i2_VG_Ma_ar_203::g.49092::m.49092	3.15	1.98	YES	NO		
	Gene.41230::c10441_g1_i2_VG_Ma_ar_203::g.41230::m.41230	3.34	1.44	NO	NO		
	Gene.94719::c27227_g1_i1_VG_Ma_ar_203::g.94719::m.94719	10.66	9.83	NO	NO		
	Gene.87312::c19725_g1_i1_VG_Ma_ar_203::g.87312::m.87312	10.90	8.61	NO	NO		
	Gene.8468::c4703_g2_i1_VG_Ma_ar_203::g.8468::m.8468	1.36	0.48	NO	NO		
	Gene.100456::c33123_g1_i1_VG_Ma_ar_203::g.100456::m.100456	9.71	4.53	NO	NO		
	Gene.25790::c8691_g1_i4_VG_Ma_ar_203::g.25790::m.25790	6.18	1.07	NO	NO		
	Gene.117760::c50918_g1_i1_VG_Ma_ar_203::g.117760::m.117760	6.13	29.83	NO	NO		
	Gene.7457::c4377_g1_i1_VG_Ma_ar_203::g.7457::m.7457	0.74	0.48	NO	NO		
	Gene.114524::c48202_g1_i1_VG_Ma_ar_203::g.114524::m.114524	4.12	2.06	NO	NO		
	Gene.15331::c6833_g1_i2_VG_Ma_ar_203::g.15331::m.15331	4.29	10.83	NO	NO		
	Gene.18925::c7561_g1_i2_VG_Ma_ar_203::g.18925::m.18925	3.50	5.42	NO	NO		
	Gene.11985::c5955_g1_i1_VG_Ma_ar_203::g.11985::m.11985	1.99	0.42	NO	NO		
	Gene.11987::c5955_g1_i2_VG_Ma_ar_203::g.11987::m.11987	2.35	5.24	NO	NO		
	Gene.108067::c41091_g1_i1_VG_Ma_ar_203::g.108067::m.108067	0.61	0.30	NO	NO		
	Gene.124771::c58257_g1_i1_VG_Ma_ar_203::g.124771::m.124771	0.45	0.26	NO	NO		
	Gene.2018::c1155_g1_i2_VG_Ma_ar_203::g.2018::m.2018	0.60	0.43	NO	NO		
	Gene.2916::c1661_g1_i1_VG_Ma_ar_203::g.2916::m.2916	2.47	4.07	NO	NO		
	Gene.2917::c1661_g1_i2_VG_Ma_ar_203::g.2917::m.2917	2.47	4.07	NO	NO		
	Gene.2019::c1155_g2_i1_VG_Ma_ar_203::g.2019::m.2019	0.81	0.48	NO	NO		
	Gene.96020::c28833_g1_i1_VG_Ma_ar_203::g.96020::m.96020	2.04	1.63	NO	NO		
	Gene.12363::c6050_g1_i1_VG_Ma_ar_203::g.12363::m.12363	0.85	1.05	NO	NO		
	Gene.123904::c57601_g1_i1_VG_Ma_ar_203::g.123904::m.123904	1.77	6.78	NO	NO		
	Gene.15332::c6833_g1_i3_VG_Ma_ar_203::g.15332::m.15332	1.33	5.43	NO	NO		
	Gene.4413::c2527_g1_i1_VG_Ma_ar_203::g.4413::m.4413	1.24	3.21	NO	NO		
	Gene.4415::c2527_g1_i2_VG_Ma_ar_203::g.4415::m.4415	1.24	3.21	NO	NO		
	Gene.110485::c43311_g1_i1_VG_Ma_ar_203::g.110485::m.110485	0.49	0.24	NO	NO		
	Gene.124519::c58058_g1_i1_VG_Ma_ar_203::g.124519::m.124519	0.63	0.64	NO	NO		
	Gene.81525::c14017_g1_i1_VG_Ma_ar_203::g.81525::m.81525	0.43	0.38	NO	NO		
	Gene.129254::c62591_g1_i1_VG_Ma_ar_203::g.129254::m.129254	0.39	0.41	NO	NO		
	Gene.6478::c3845_g1_i1_VG_Ma_ar_203::g.6478::m.6478	0.84	2.89	NO	NO		
	Gene.858::c465_g1_i1_VG_Ma_ar_203::g.858::m.858	0.81	1.93	NO	NO		
	Gene.860::c465_g1_i2_VG_Ma_ar_203::g.860::m.860	0.62	0.00	NO	NO		
	Gene.104715::c37844_g1_i1_VG_Ma_ar_203::g.104715::m.104715	0.32	0.56	NO	NO		
	Gene.2017::c1155_g1_i1_VG_Ma_ar_203::g.2017::m.2017	0.37	0.00	NO	NO		
	Gene.104253::c37223_g1_i1_VG_Ma_ar_203::g.104253::m.104253	0.26	0.18	NO	NO		
	Gene.111990::c44958_g1_i1_VG_Ma_ar_203::g.111990::m.111990	0.42	2.28	NO	NO		
	Gene.99484::c32288_g1_i1_VG_Ma_ar_203::g.99484::m.99484	0.38	3.39	NO	NO		
	Gene.124790::c58277_g1_i1_VG_Ma_ar_203::g.124790::m.124790	0.28	0.61	NO	NO		
	Gene.36686::c10004_g1_i1_VG_Ma_ar_203::g.36686::m.36686	0.26	0.41	NO	NO		
	Gene.36692::c10004_g1_i2_VG_Ma_ar_203::g.36692::m.36692	0.26	0.41	NO	NO		
	Gene.111536::c44438_g1_i1_VG_Ma_ar_203::g.111536::m.111536	0.24	0.95	NO	NO		
	Gene.12562::c6112_g1_i1_VG_Ma_ar_203::g.12562::m.12562	0.36	1.97	NO	NO		
	Gene.112407::c45435_g1_i1_VG_Ma_ar_203::g.112407::m.112407	0.28	0.86	NO	NO		
	Gene.113559::c47092_g1_i1_VG_Ma_ar_203::g.113559::m.113559	0.28	1.85	NO	NO		
	Gene.130598::c64471_g1_i1_VG_Ma_ar_203::g.130598::m.130598	0.25	1.36	NO	NO		
	Gene.101633::c34223_g1_i1_VG_Ma_ar_203::g.101633::m.101633	0.21	0.86	NO	NO		
	Gene.118107::c51243_g1_i1_VG_Ma_ar_203::g.118107::m.118107	0.19	1.28	NO	NO		
	Gene.82452::c14732_g1_i1_VG_Ma_ar_203::g.82452::m.82452	0.15	1.05	NO	NO		
**Sphingomyelinase**	Gene.49297::c11093_g3_i1_VG_Ma_ar_203::g.49297::m.49297	7.52	1.74	YES	NO		
	Gene.49308::c11093_g3_i2_VG_Ma_ar_203::g.49308::m.49308	7.52	1.74	YES	NO		
	Gene.19930::c7750_g1_i1_VG_Ma_ar_203::g.19930::m.19930	1.94	1.11	YES	NO		
	Gene.97096::c30520_g1_i1_VG_Ma_ar_203::g.97096::m.97096	0.25	0.00	NO	NO		
	Gene.80725::c13388_g1_i1_VG_Ma_ar_203::g.80725::m.80725	0.17	0.00	NO	NO		
	Gene.127133::c60379_g1_i1_VG_Ma_ar_203::g.127133::m.127133	0.20	1.53	NO	NO		
**SVWC-domain 1 (Asilidin8)**	Gene.10561::c5412_g1_i1_VG_Ma_ar_203::g.10561::m.10561	5892.41	5055.18	YES	YES	U-Asilidin8-Mar1a	
	Gene.1996::c1145_g1_i1_VG_Ma_ar_203::g.1996::m.1996	5.59	5.13	YES	NO		
	Gene.6951::c4164_g1_i1_VG_Ma_ar_203::g.6951::m.6951	2.13	1.62	YES	NO		
**SVWC-domain 2 (Asilidin9)**	Gene.19409::c7651_g1_i1_VG_Ma_ar_203::g.19409::m.19409	9419.14	9288.18	YES	YES	U-Asilidin9-Mar1a	
	Gene.1624::c925_g1_i1_VG_Ma_ar_203::g.1624::m.1624	1.32	1.03	YES	NO		
**Venom acid phosphatase-like**	Gene.47513::c10949_g2_i2_VG_Ma_ar_203::g.47513::m.47513	1923.41	796.97	YES	YES		
	Gene.47507::c10949_g2_i1_VG_Ma_ar_203::g.47507::m.47507	1026.94	479.11	YES	YES		
	Gene.47502::c10949_g1_i1_VG_Ma_ar_203::g.47502::m.47502	26.87	12.41	NO	NO		
	Gene.51883::c11286_g1_i1_VG_Ma_ar_203::g.51883::m.51883	11.20	3.07	NO	NO		
	Gene.51890::c11286_g1_i2_VG_Ma_ar_203::g.51890::m.51890	11.20	3.07	NO	NO		
	Gene.51898::c11286_g1_i3_VG_Ma_ar_203::g.51898::m.51898	10.66	0.00	NO	NO		
	Gene.23092::c8291_g1_i1_VG_Ma_ar_203::g.23092::m.23092	11.66	5.82	NO	NO		
	Gene.57502::c11663_g2_i1_VG_Ma_ar_203::g.57502::m.57502	6.18	3.20	YES	NO		
	Gene.21344::c8010_g1_i1_VG_Ma_ar_203::g.21344::m.21344	9.09	6.00	NO	NO		
	Gene.5599::c3273_g1_i1_VG_Ma_ar_203::g.5599::m.5599	5.38	13.57	NO	NO		
