# Supplementary material for: A Dipteran’s Novel Sucker Punch: Evolution of Arthropod Atypical Venom with a Neurotoxic Component in Robber Flies (Asilidae, Diptera)
Source: Toxins (Basel). 2018 Jan 5;10(1):29. doi: 10.3390/toxins10010029 (PMC5793116; doi:10.3390/toxins10010029)
Supplement: Supplementary file 1 [file toxins-10-00029-s001.zip › toxins-254261-supplementary/toxins-254261SupplementaryFile1_Proof.pdf]

Supplementary Materials: A Dipteran's Novel Sucker Punch: Evolution of Arthropod Atypical Venom with a Neurotoxic Component in Robber Flies (Asilidae, Diptera)

Stephan Holger Drukewitz, Nico Fuhrmann, Eivind A. B. Undheim, Alexander Blanke, Julien Giribaldi, Rosanna Mary, Guillaume Laconde, Sébastien Dutertre and Björn Marcus von Reumont

Identified enzymatic components in the asilid venom that were only present in the proteome of one species

Peptidase S1 digests proteins by cleaving their peptide bonds [1]. Proteins of the PS1 family are known to be recruited into venoms convergently multiple times in reptiles, hymenopterans, cephalopods, bats and hematophagouse arthropods [2]. Considering the mode of feeding of asilids it is likely that PS1 are necessary for liquefying the prey, however, we recover peptidase in the proteome only for M. arthriticus.

The venom acidic phosphatase secreted in the thoracic glands of M. arthriticus is known to be one of the major allergens in the venom of Apis melifera [3–6], but is also known from the venom of other hymenopterans like bumblebees or paper wasps [4,6]. The function needs to be evaluated in flies, however, it might be speculated given its broad occurrence in defensive hymenopteran venom that it is deployed also in robber flies as a defensive toxin.

This interpretation would be supported by another enzyme present in M. arthriticus, a Natterin-like protein. Two sequences with a Natterin-like domain known from the venom of the toad fish Thalassophryne nattereri are secreted in the thoracic glands of M. arthriticus, (see Table2, Figure S9). Similar sequences were also identified in the transriptome of E. rufibarbis but are absent in the proteome. Natterin was first described in the venom of Thalassophryne nattereri, later it was also identified in the skin secreting of oriental catfishs (Plotosus lineatus) [7–9] being rather used for defensive purpose. Yet, Natterin-like toxins show a lethal activity in mice and arthropods, whereas arthropods (freshwater crabs) are much more sensitive. This high sensitivity of arthropods to natterin like toxins could imply that assilids may use this primary defensive toxin in an offensive manner to overpower their prey. Currently no study exists that tests differences in offensive and defensive venom compositions of asilids, or if asilids generally use defensive stings.

Phospholipase catalyzes the hydrolysis of phospholipids into fatty acids and other lipophilic substances. One secreted Pospholipase A2 was identified for M.arthriticus, Pla2 proteins are known venom components of cepahlopods, scorpions, ticks, spiders, several insects and reptiles [2]. They are known for several toxic activities, like cytotoxicity, myotoxicity, neurotoxicity or antiplatelet activity [10,11]. We can only speculate about a possible function in robber flies eventually it acts as a neurotoxic component or spreading factor for the third fraction of putative asilid venom which shows neurotoxicity.

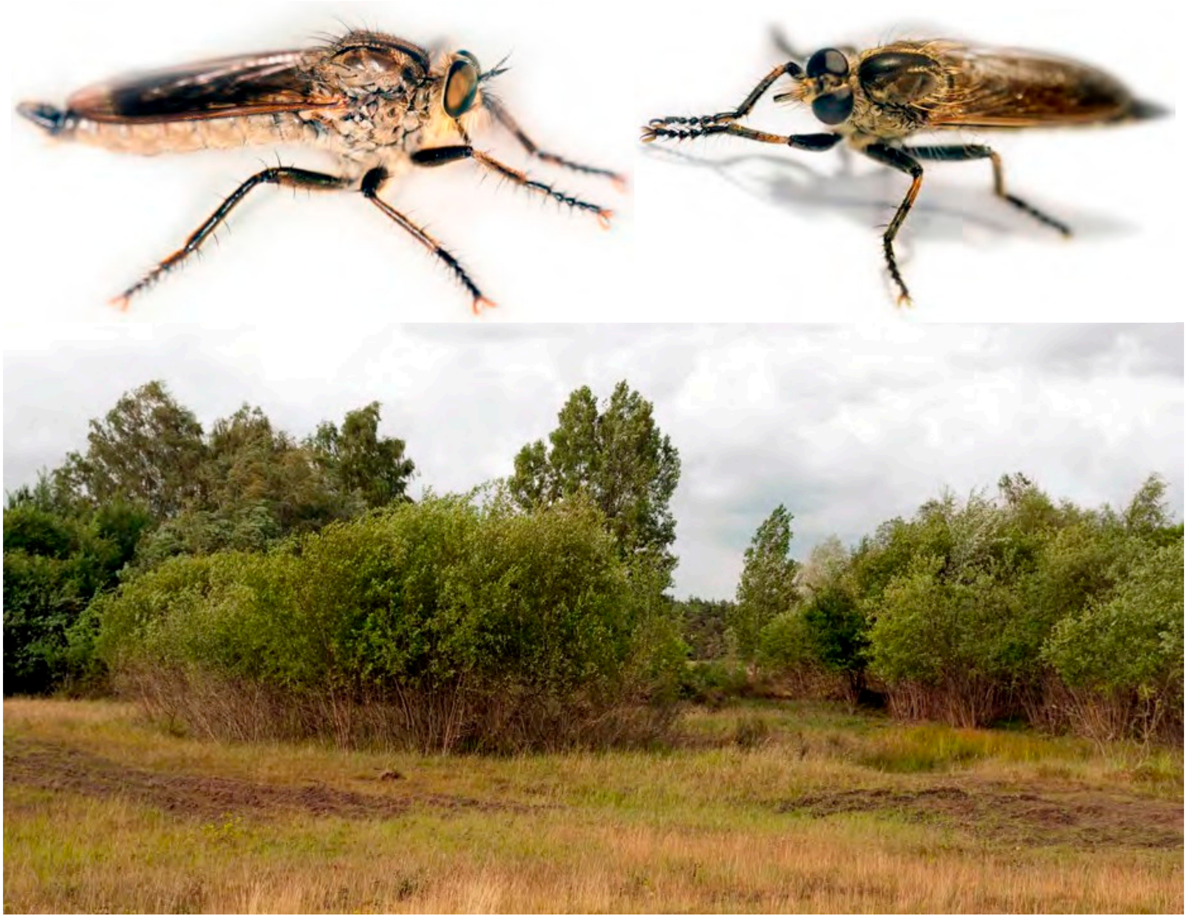

Figure S1. Habitus and habitat of the two studied robber flies. (top left) *Eutolmus rufibarbis*; (top right) *Machimus arthriticus*; (bottom) Heathland habitat where these two species were collected.

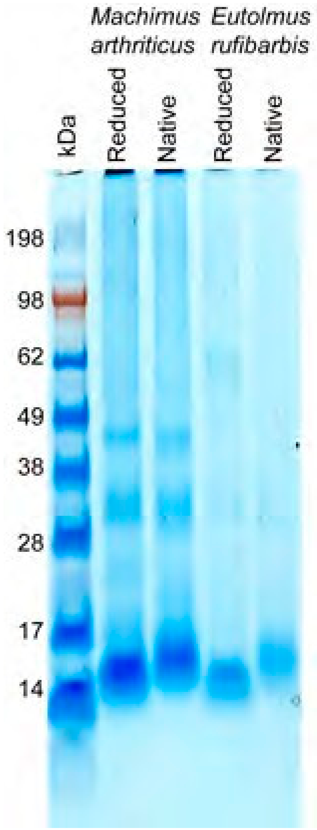

Figure S2. SDS-PAGE gel of crude venom of Machimus arthriticus and Eutolmus rufibarbis stained with Coomassie blue.

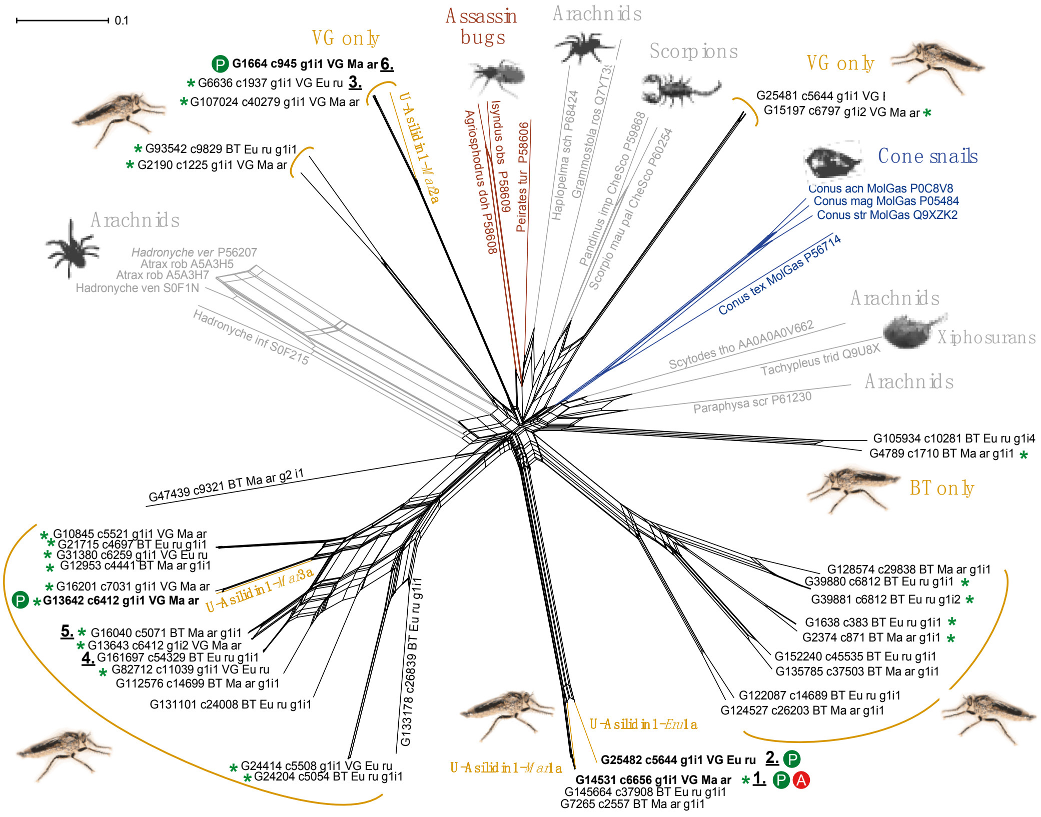

Figure S3. Neighbor network of the asilidin1 sequences identified in *M. athriticus* and *E. rufibarbis* and known cysteine inhibitor knot toxins from cone snails, spiders, scorpions, assassin bugs and xiphosurans. Robber fly sequences that were confirmed in the proteome are marked with a white P in a green circle. The sequence for which activity was tested is marked with a white A in a red circle.

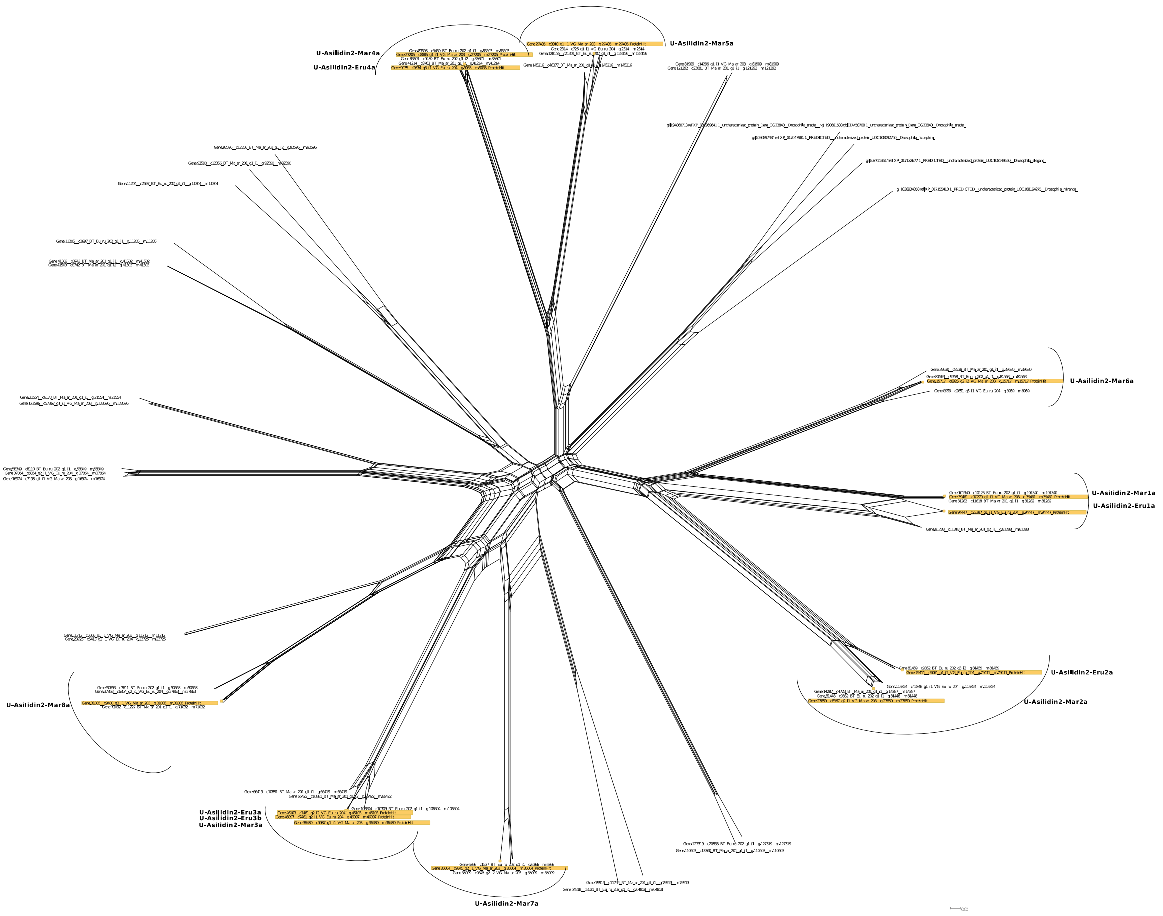

Figure S4. Neighbor network of Asilidin 2, new named protein hits are highlighted.

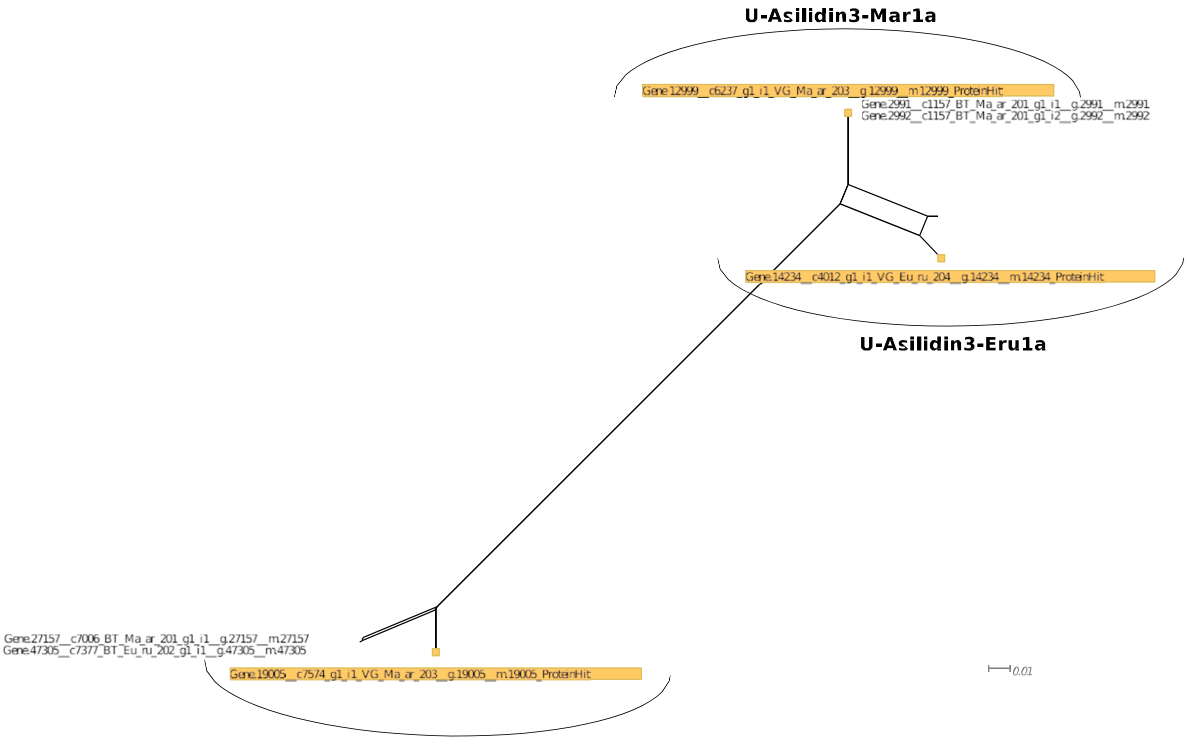

Figure S5. Neighbor network of Asilidin 3, new named protein hits are highlighted.

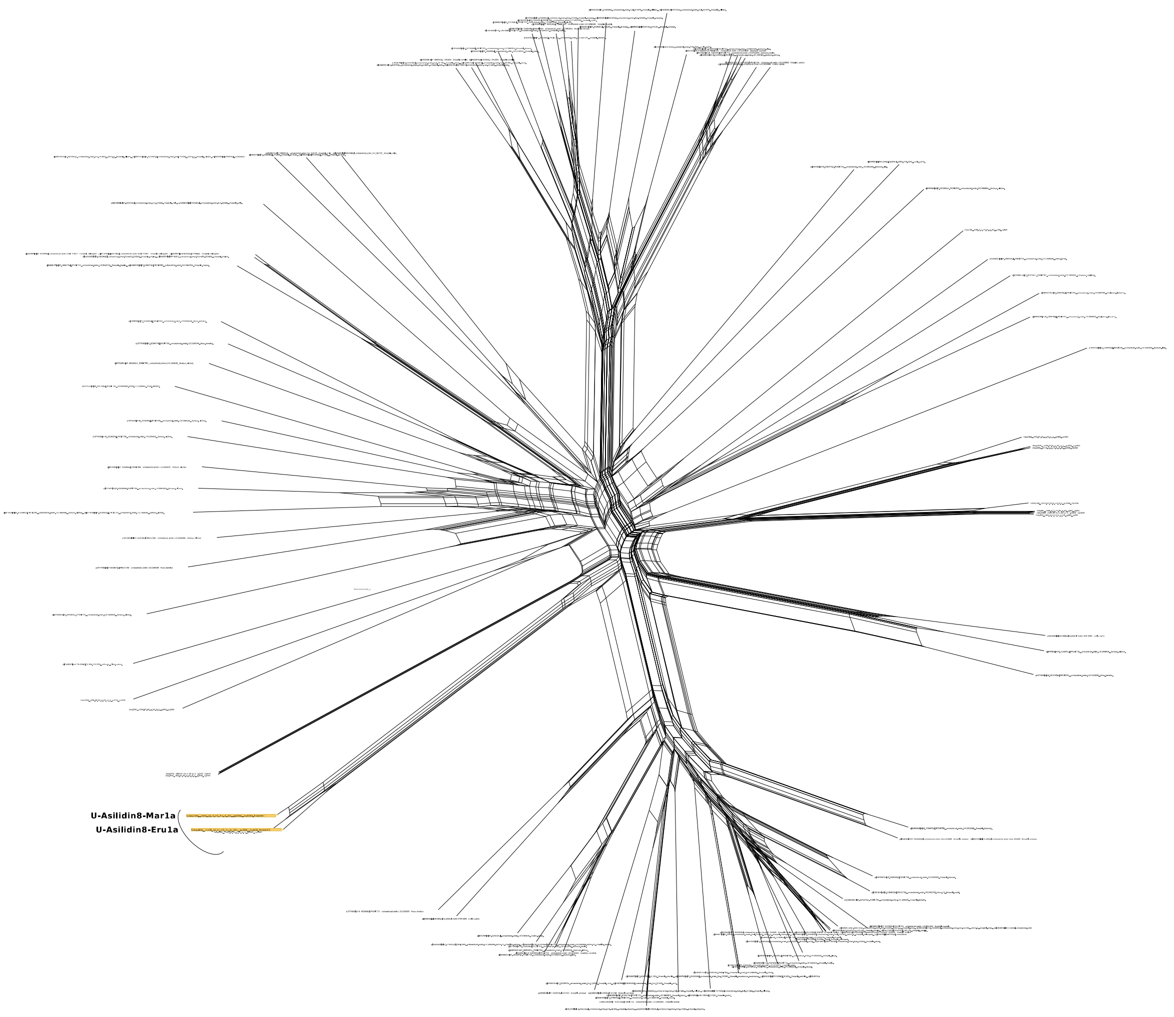

Figure S6. Neighbor network of Asilidin 8, new named protein hits are highlighted.

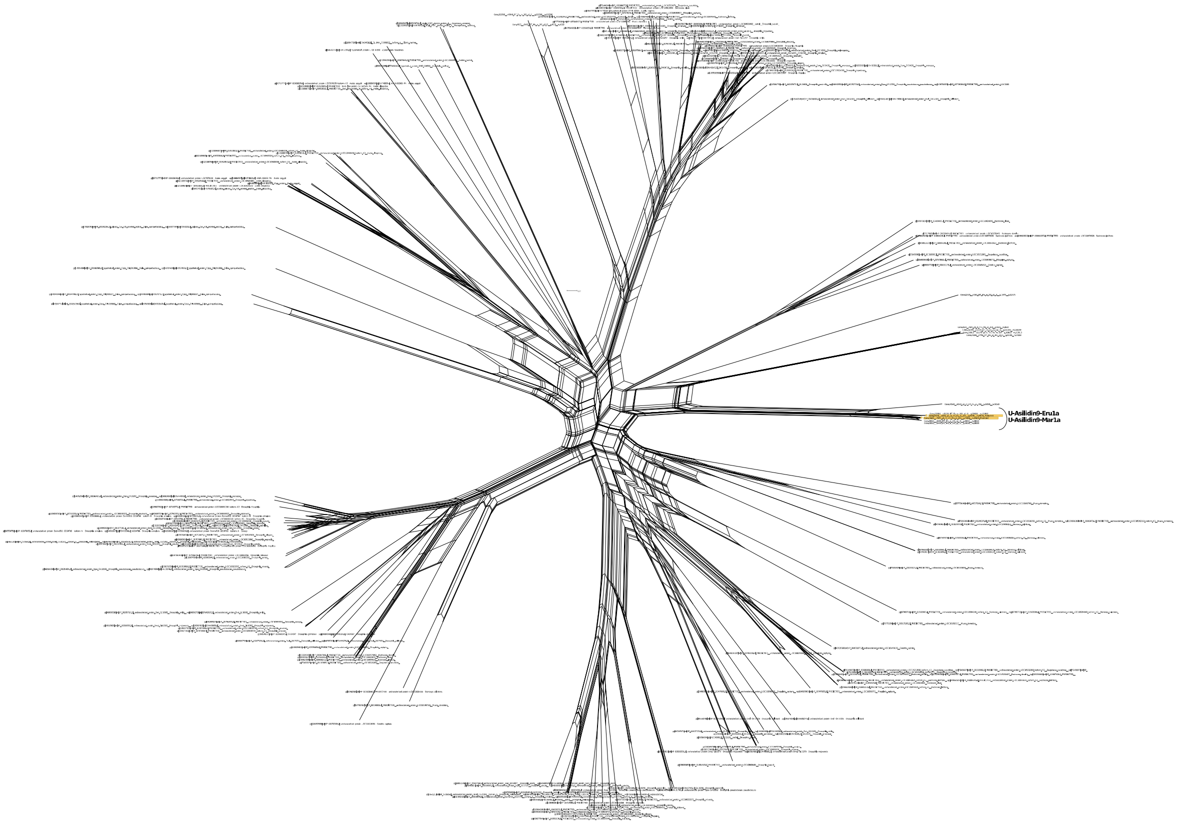

Figure S7. Neighbor network of Asilidin 9, new named protein hits are highlighted.

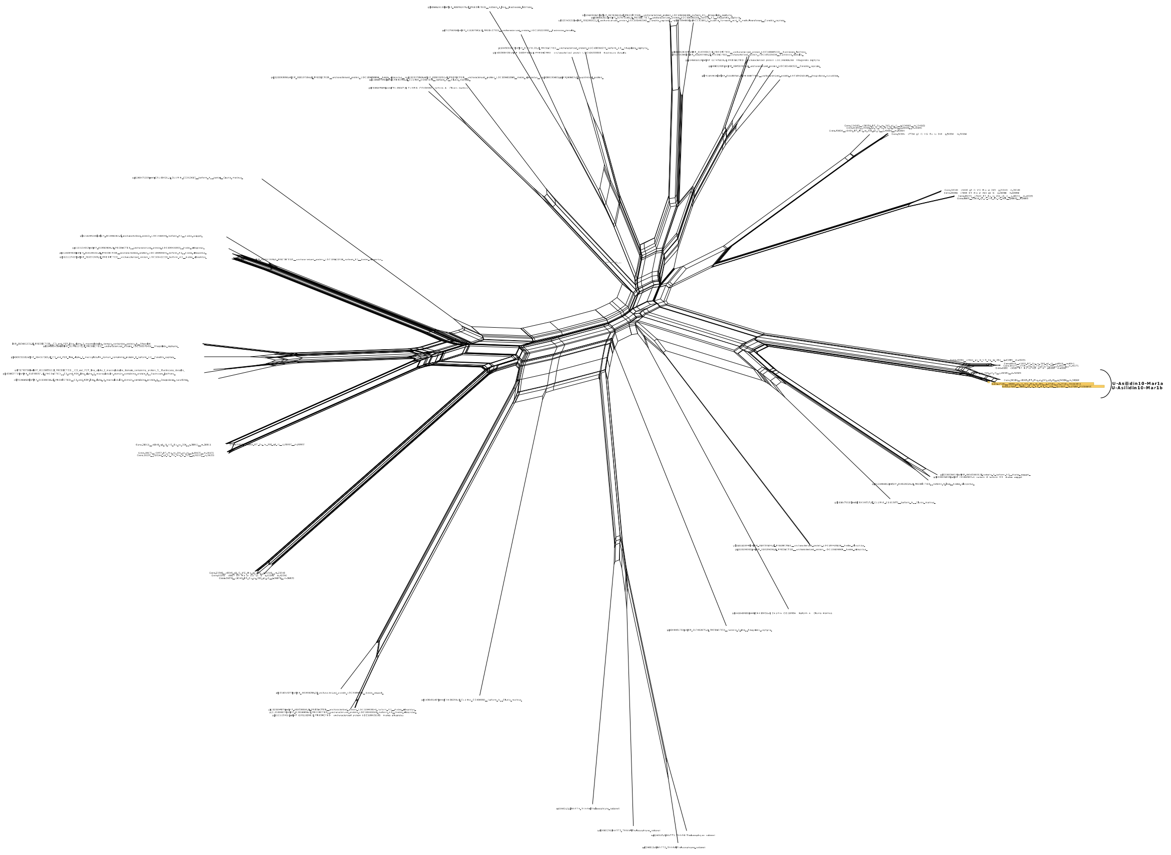

Figure S8. Neighbor network of Asilidin 10, new named protein hits are highlighted.

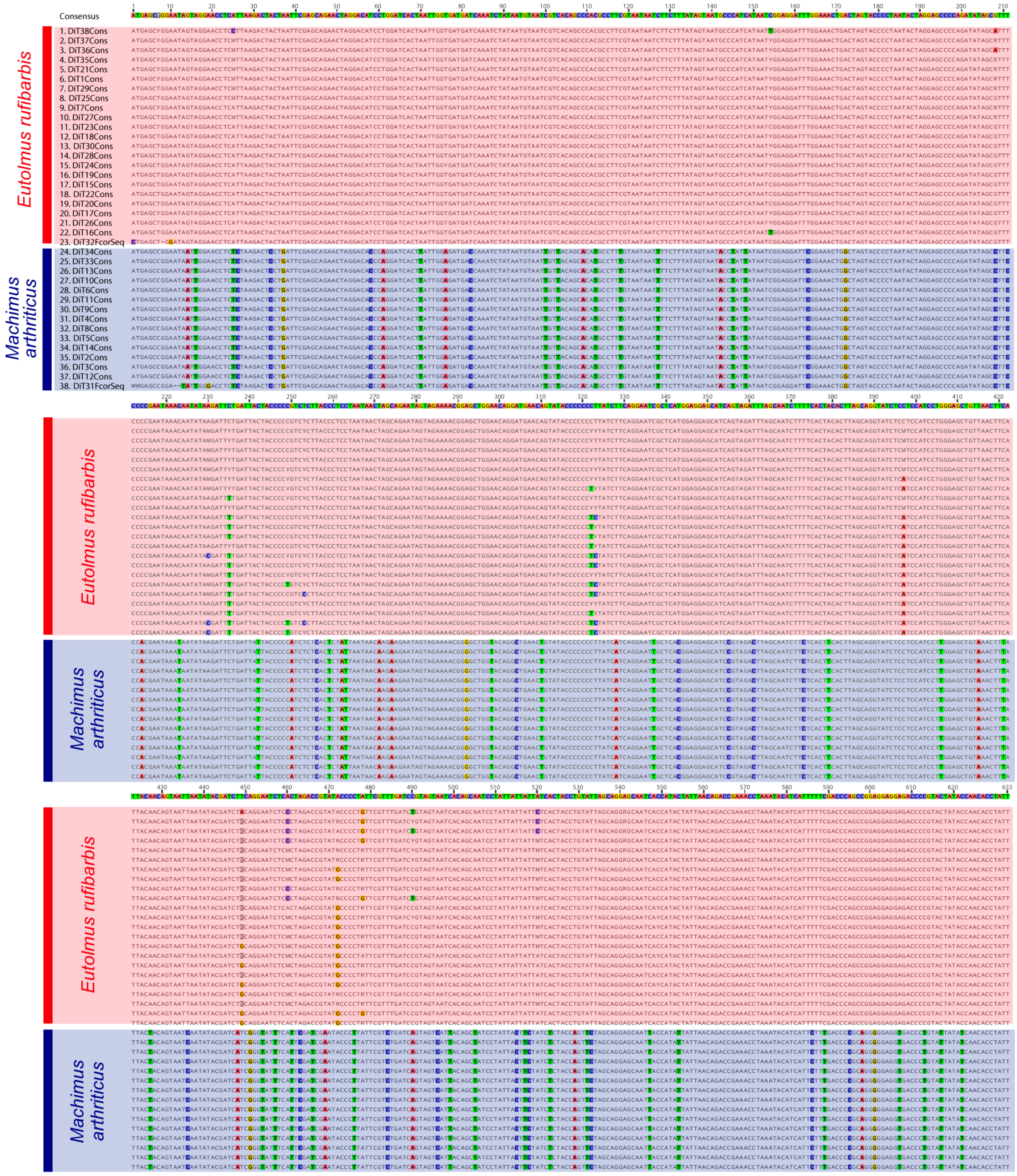

Figure S9. Alignment of CO1 barcode sequences of specimens from *E. rufibarbis* and *M. arthritcus* used for our analyses.

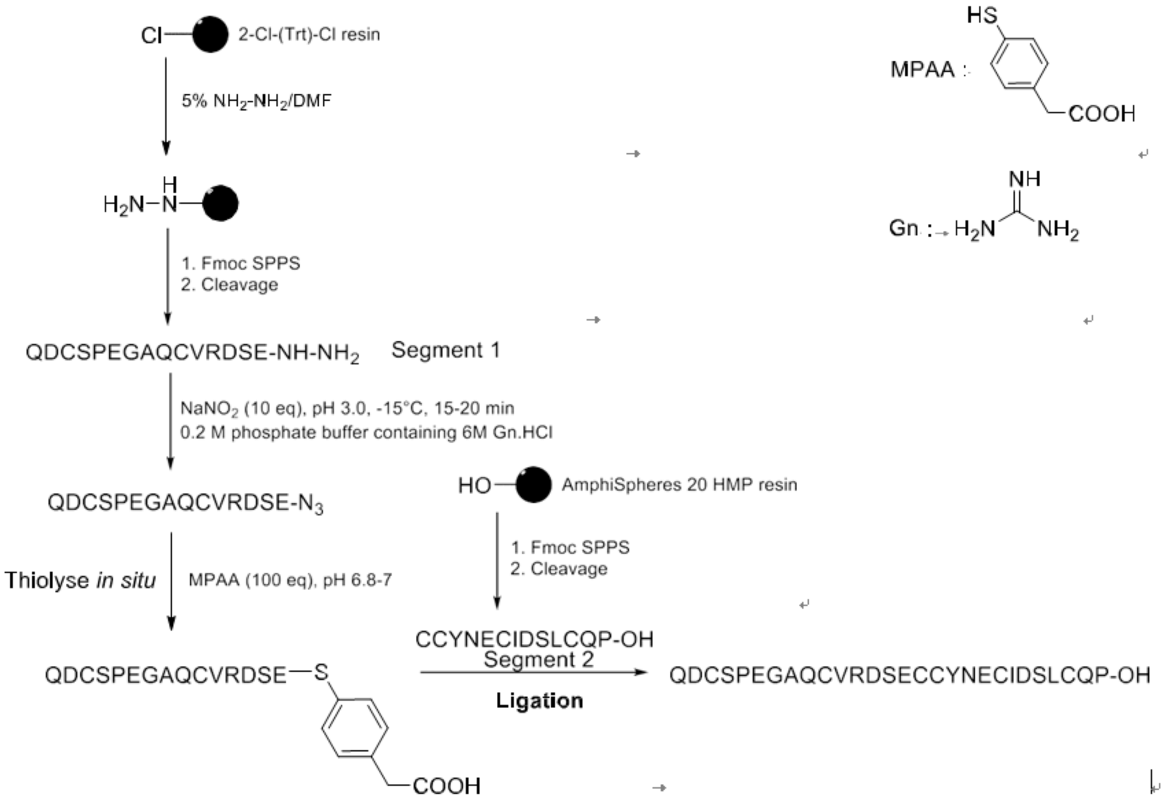

Figure S10. Overall strategy for asilidin1 peptide synthesis.

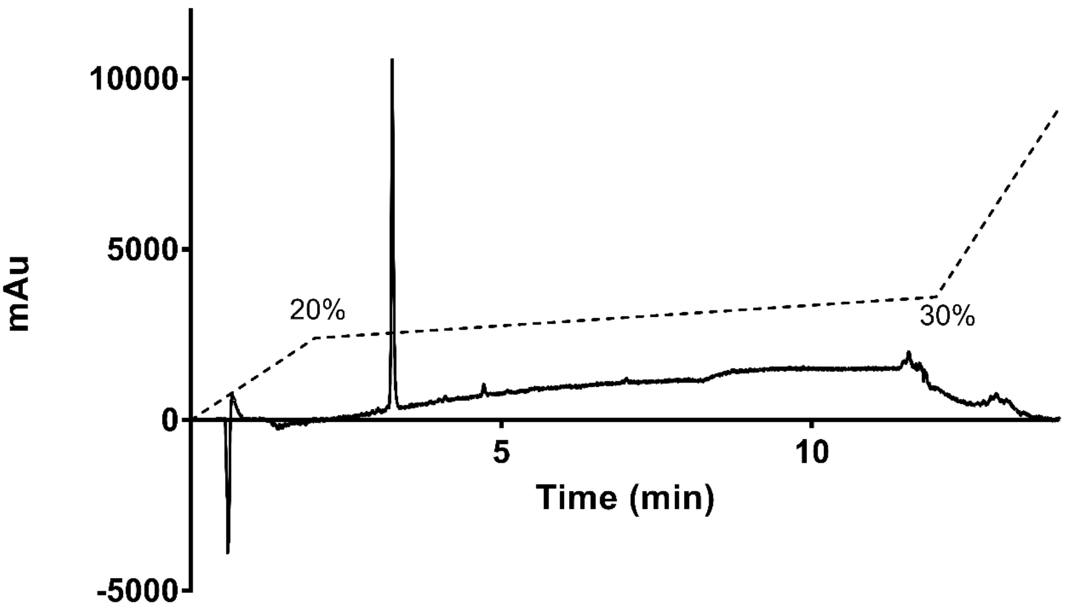

Figure S11. UV chromatogram of linear toxin (dashed line indicates acetonitriles gradient).

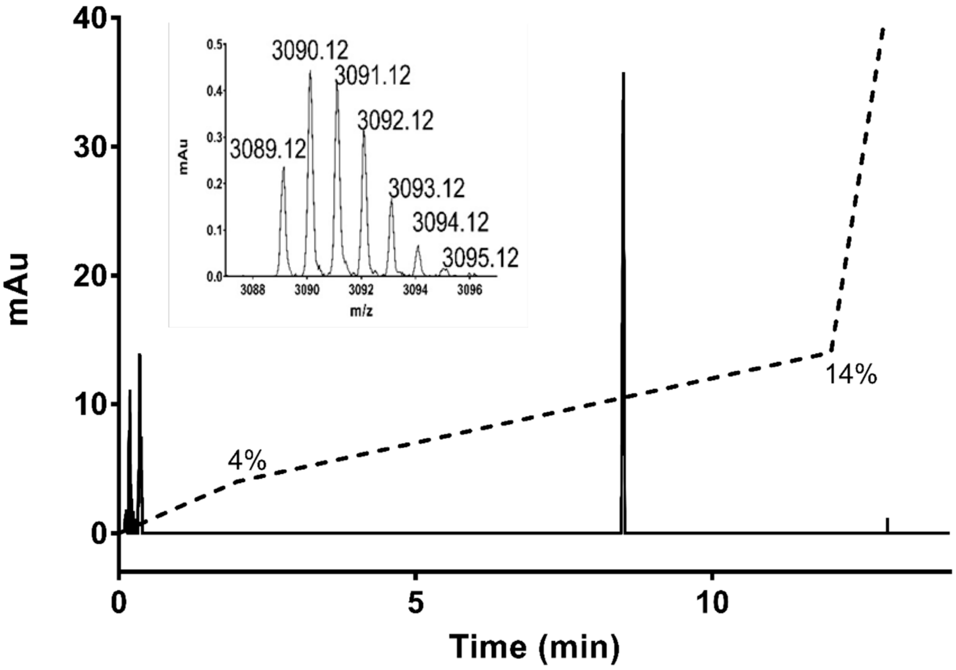

Figure S12. UV chromatogram of folded toxin and MALDI analysis (dashed line indicates acetonitrile gradient).

References

1. Muhlia-Almazán A; Sánchez-Paz A; Garc'\ia-Carreño FL. Invertebrate trypsins: a review. *J. Comp. Physiol. B*. **2008**, *178*, 655–72.
2. Fry BG; Roelants K; Champagne DE; Scheib H; Tyndall JDA; King GF. The Toxicogenomic Multiverse: Convergent Recruitment of Proteins Into Animal Venoms. *Annu. Rev. Genomics Hum. Genet. Annual Reviews*, **2009**, *10*, 483–511.
3. Grunwald T; Bockisch B; Spillner E; Ring J; Bredehorst R; Ollert M. Molecular cloning and expression in insect cells of honeybee venom allergen acid phosphatase (Api m 3). *J. Allergy Clin. Immunol.* **2006**, *117*, 848–54.
4. Zhu J ying; Ye G yin; Hu C. Molecular cloning and characterization of acid phosphatase in venom of the endoparasitoid wasp Pteromalus puparum (Hymenoptera: Pteromalidae). *Toxicon*. **2008,** *51,* 1391–1399.
5. Barboni E; Kemeny DM; Campos S; Vernon CA. The purification of acid phosphatase from honey bee venom (Apis mellifica). *Toxicon*. **1987**, *25*, 1097–103.
6. Hoffman DR. Allergens in bee venom. III. Identification of allergen B of bee venom as an acid phosphatase. *J. Allergy Clin. Immunol.* **1977**, *59*, 364–6.
7. Magalhães GS; Lopes-Ferreira M; Junqueira-de-Azevedo ILM; Spencer PJ; Araújo MS; Portaro FC V. Natterins, a new class of proteins with kininogenase activity characterized from Thalassophryne nattereri fish venom. *Biochimie*. **2005**, *87*, 687–99.
8. Tamura S; Yamakawa M; Shiomi K. Purification, characterization and {cDNA} cloning of two natterin-like toxins from the skin secretion of oriental catfish Plotosus lineatus. *Toxicon*. **2011**, *58*, 430–8.
9. Lopes-Ferreira M; Grund LZ; Lima C. Thalassophryne nattereri fish venom: from the envenoming to the understanding of the immune system. *J. Venom. Anim. Toxins Incl. Trop. Dis.* **2014**, doi:10.1186/1678-9199-20-35. eCollection 2014.
10. Fry BG. From genome to "venome": Molecular origin and evolution of the snake venom proteome inferred from phylogenetic analysis of toxin sequences and related body proteins. *Genome Res. Cold Spring Harbor Laboratory* **2005**, *15*, 403–20.
11. Kini RM. Excitement ahead: structure, function and mechanism of snake venom phospholipase A2 enzymes. *Toxicon*. **2003**, *42*, 827–40.
